# Age-Dependent Variations in Functional Quality and Proteomic Characteristics of Canine (*Canis lupus familiaris*) Epididymal Spermatozoa

**DOI:** 10.3390/ijms23169143

**Published:** 2022-08-15

**Authors:** Anna Zmudzinska, Jerzy Wisniewski, Piotr Mlynarz, Beata Olejnik, Marzena Mogielnicka-Brzozowska

**Affiliations:** 1 Department of Animal Biochemistry and Biotechnology, University of Warmia and Mazury in Olsztyn, Oczapowskiego 5, 10-719 Olsztyn, Poland; 2 Department of Biochemistry, Molecular Biology and Biotechnology, Wroclaw University of Science and Technology, Wyspianskiego 27, 50-370 Wroclaw, Poland; 3 Department of Biochemistry and Immunochemistry, Wroclaw Medical University, Chalubinskiego 10, 50-368 Wroclaw, Poland

**Keywords:** epididymal spermatozoa, aging, proteins, canine

## Abstract

Increased male age is associated with a significant reduction in semen quality. Little is known about the sperm proteome changes resulting from the aging process. This study aimed to investigate the relationship between the functional quality and proteome of epididymal spermatozoa of dogs that were differing in age. The study was conducted on 30 male dogs that were divided into three age groups. G1—12 to 41 months old, G2—42 to 77 months old, and G3—78 to 132 months old. The sperm samples were assessed using a computer-assisted semen analysis (CASA). The epididymal sperm proteins were analyzed using gel electrophoresis (SDS-PAGE), nano-liquid chromatography coupled to quadrupole time of flight mass spectrometry (NanoUPLC-Q-TOF/MS) and bioinformatic tools. The sperm quality parameters were significantly lower in older dogs. NanoUPLC-Q-TOF/MS identification resulted in 865 proteins that were found in the G1, 472 in G2, and 435 in G3. There were seven proteins that were present in all three age groups, and four of them (ACTB, CE10, NPC2, CRISP2) showed significant changes among the studied groups. Age-dependent variations were detected in the sperm proteome composition and were related to important metabolite pathways, which might suggest that several proteins are implicated in sperm maturation and could be potential aging biomarkers.

## 1. Introduction

Canine epididymal semen provides an additional source of gametes to preserve the genetics of valuable breeding dogs [1]. Moreover, the development of a gene bank of epididymal semen would greatly contribute to increasing the genetic diversity in dogs and also in endangered canids [1]. Additionally, the dog already fulfils many of the criteria of a good model of the human epididymis on a molecular level also regarding aging [2,3].

For dog breeders, it is important to know how long they may use semen from old high-quality reproducers without losing the semen quality. Thus, the possible effect of male age on fertility has become increasingly important in animal reproduction. While the influence of female age on fertility is well established, the impact of male age is poorly characterized [4]. It has been observed that age is associated with diminished dog sperm quality [5,6,7]. Age-dependent changes in sperm quality have been reported in the rat [8,9], hamster [10], ferret [11], cat [12], boar [13], and in humans [14]. Many different techniques have been used to assess the reproductive potential of male dogs, but little is still known about the protein composition changes during the aging processes of the epididymis and epididymal spermatozoa. Associations have been shown among age and epididymal and accessory sex gland function in humans according to sperm progressive motility and protein activity such as NAG (neutral α-glucosidase) and PSA (prostate-specific antigen) [15]. The declining sperm motility that is observed in older men might be due to age-dependent changes in epididymal and accessory sex gland function [15]. The epididymis and accessory sex gland function are highly dependent on androgens. The epididymis undergoes morphological changes with aging, which probably affects sperm maturation [10,15]. However, the particular molecular mechanisms of epididymal sperm aging based on changes in the protein structures and function have not yet been determined.

Motility is believed to be one of the most important parameters in evaluating the fertilizing ability of spermatozoa [16]. Sperm motility is the result of a multitude of factors, including post-testicular molecular processes. Following spermatogenesis, the spermatozoa undergo maturation in the epididymis, including gaining their ability to be motile [17]. Protein components that are derived from epididymal ducts play a very important role in these events [2]. Sperm structures and functions are affected by protein degradation and post-translational modifications [18]. Both protein interaction with the sperm plasma membrane and inner protein recompositing play a basic role in the epididymal sperm maturation [2,3]. Many internal and external factors may influence these protein qualities and functions, which are closely related to sperm fertilizing ability [17].

Only a few studies have been conducted to determine the protein-based molecular mechanisms that are responsible for the age-dependent decline in epididymal sperm quality. Most notably, Silva et al. [4] noted increased levels of apoptotic/stress markers: cellular tumor antigen p53 (TP53) and mitogen-activated protein kinases (MAPKs) activity.

This study aimed to investigate the relationship between the functional quality and proteome of epididymal spermatozoa of dogs that were differing in age.

## 2. Results

### 2.1. Epididymal Sperm Quality Assessment

The concentration of epididymal spermatozoa in the studied group of dogs ranged as follows: Group 1 (G1), from 21.4 to 54.9 × 10^8^ spermatozoa/mL (38.2 ± 3.2 × 10^8^ spermatozoa/mL, mean ± SE); Group 2 (G2), from 9.7 to 49.4 × 10^8^ spermatozoa/mL (27.7 ± 3.7 × 10^8^ spermatozoa/mL, mean ± SE); and Group 3 (G3), from 6.8 to 39.6 × 10^8^ spermatozoa/mL (24.8 ± 3.9 × 10^8^ spermatozoa/mL, mean ± SE) (Table 1).

The sperm concentration, VSL, and VCL that were measured in the three age groups differed statistically significantly (*p* < 0.05) between G1 and G3. The results also showed significant differences (*p* < 0.05) in the percentage of TMOT, PMOT, and VAP values between G3 compared with G1 and G2 (Table 1).

A statistically significant negative correlation with dog age group was shown for the following parameters: sperm concentration (r = −0.43; *p* < 0.05), TMOT % (r = −0.55; *p* < 0.005), PMOT % (r = −0.49; *p* < 0.01), VAP (r = −0.54; *p* < 0.005), VCL (r = −0.44; *p* < 0.05), and VSL (r = −0.52; *p* < 0.005).

### 2.2. Epididymal Sperm Morphology Assessment

**Figure 1:**
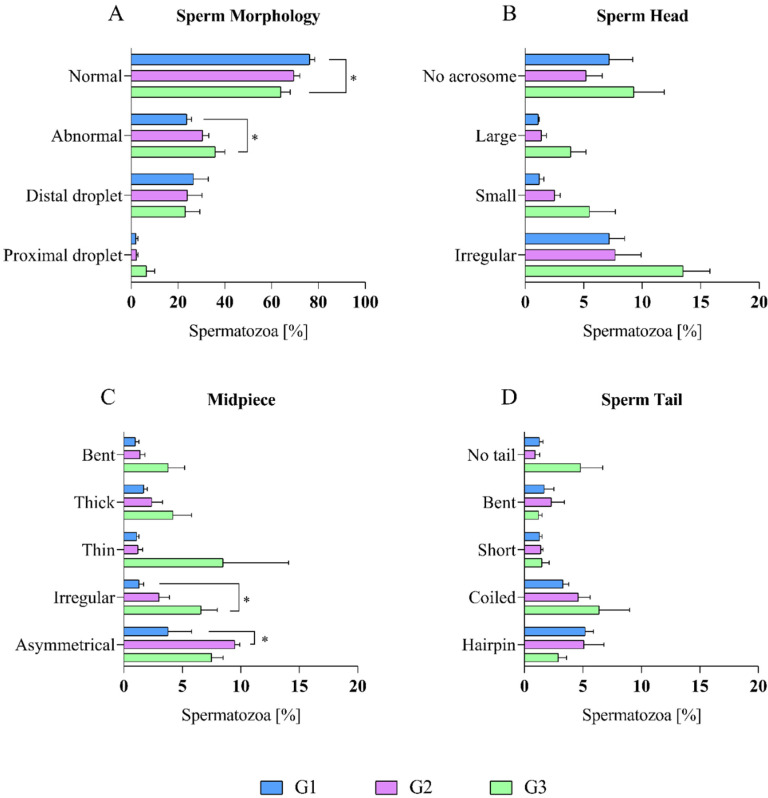
Morphological characteristics of the epididymal spermatozoa of dogs (*Canis lupus familiaris*) in different age groups (G1: 12–41 months old; G2: 42–77 months old; G3: 78–132 months old). The dog epididymal spermatozoa were stained with Spermac Stain™. (**A**) Sperm morphology was analysed according to the World Health Organization guidelines: normal (without primary defects), abnormal (at least one defect), sperm with distal droplet and sperm with proximal droplet. (**B**) Sperm head: no acrosome (sperm head without acrosome), large (sperm with large head), small (sperm with small head), irregular (irregular shape of sperm head). (**C**) Midpiece: bent, thick, thin, irregular (irregular shape of sperm midpiece), asymmetrical (asymmetrical position of sperm midpiece). (**D**) Sperm tail: no tail (sperm without tail), bent, short, coiled, harpin (harpin-like sperm tail). Values were given as the mean ± SE. * Significant at *p* < 0.05.

The percentage of morphological changes that were found in dog epididymal spermatozoa among age groups (G1, G2, and G3) are shown in Figure 1.

When comparing the percentage of morphological defects of the dog epididymal spermatozoa among the three age groups, it was shown that the highest percentage of normal spermatozoa was found in G1 (76.3 ± 2.1%), and the lowest value was in G3 (64.0 ± 4.0%). The differences were statistically significant (*p* < 0.05) between these two groups. G2 characterized the average percentage of normal spermatozoa (69.5 ± 2.6%) (Figure 1A).

There were no significant differences in the percentage of distal and proximal droplets among the age groups (Figure 1A). Also, no significant differences were found in the percentage of sperm head defects among the age groups (Figure 1B).

When comparing morphological defects of the midpiece among the age groups, there were statistical differences (*p* < 0.05) between two of them. The lowest percentage of irregular midpiece was found in G1 (1.3 ± 0.4%), and the highest was in G3 (6.6 ± 1.4%). The highest number of asymmetrical midpieces was found in G2 (9.5 ± 0.4%), and the lowest was in G1 (3.8 ± 2.0%). The average percentage of irregular and asymmetrical midpiece was 3.0 ± 0.9% (in G2) and 7.5 ± 1.0% (G3), respectively. Defects such as a bent, thick, and thin midpiece did not have significant differences among the age groups (Figure 1C).

When comparing morphological defects of the sperm tails among the age groups, there were no statistical differences that were found (Figure 1D).

### 2.3. SDS-PAGE Analysis

**Figure 2:**
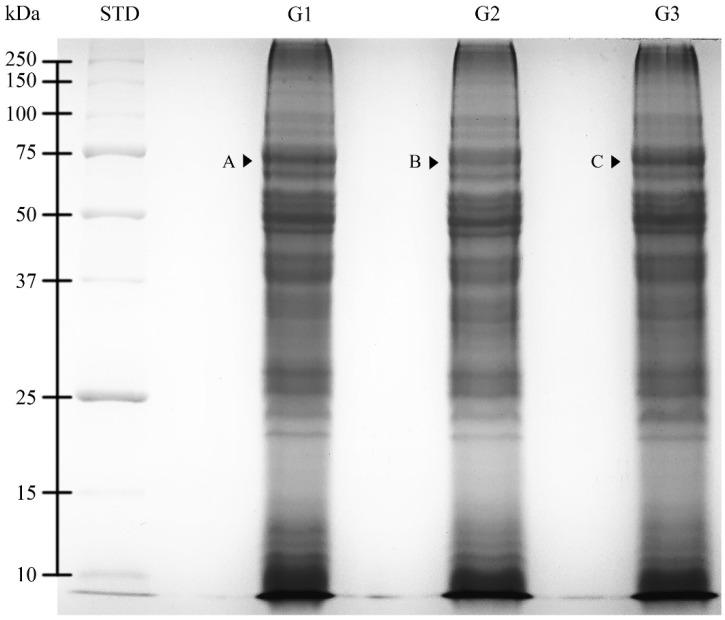
One-dimensional SDS-PAGE (12%) gel image of the cauda epididymal spermatozoa of dogs (*Canis lupus familiaris*) in different age groups (G1: 12–41 months old; G2: 42–77 months old; G3: 78–132 months old). Differentially expressed proteins (DEPs) are marked with letters A, B, and C. STD—molecular weight markers.

The SDS-PAGE protein profiles of dog epididymal spermatozoa extracts were analyzed according to the age group (G1, G2, and G3). The protein profiles for all three age groups were similar and were characterized by the presence of 36 protein fractions (PFs) with molecular weights (MW) ranging from 10.6 to >250.0 kDa (Figure 2).

An optical density (OD) analysis of PFs showed higher protein content (*p* < 0.05) for one PF (68 kDa) when compared with the corresponding (showing the same MW) PFs among the age groups. PFs in particular age groups were marked with letters from A to C (Table 2).

### 2.4. Qualitative and Quantitative Mass Spectrometry Analysis

#### 2.4.1. Qualitative Analysis

Mass spectrometry analysis identified a total of 1772 proteins in all age groups: G1—865 proteins, G2—472 proteins, and G3—435 proteins.

**Figure 3:**
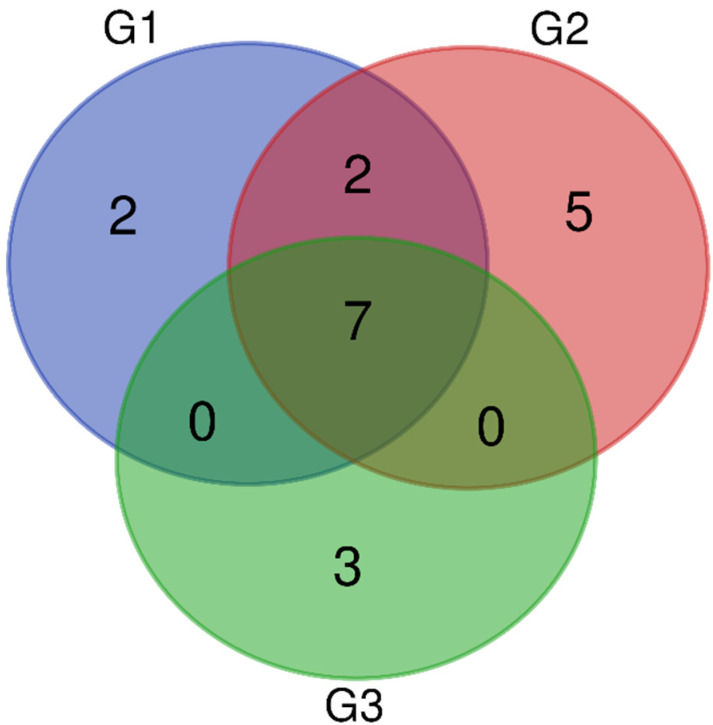
A Venn diagram showing the number of unique proteins (UPs) and common proteins that were identified in the epididymal spermatozoa of dogs (*Canis lupus familiaris*) in different age groups (G1, G2, G3).

UniProt database-supported identification resulted in two unique proteins (UPs) that were identified in G1, five UPs identified in G2, and three Ups that were identified in G3, which were present in a statistically significant manner (when present in 50% + 1 animals) (Figure 3). There were two polypeptides that were identified in G1 as UPs were: glutathione peroxidase (GPX5) and hyaluronoglucosaminidase (CEMIP). A total of five polypeptides that were identified in G2 as UPs were: mannosyl-glycoprotein endo-beta-N-acetylglucosaminidase (ENGASE), inactive ribonuclease-like protein 9 (RNASE9), clusterin (CLU), pleckstrin homolog, MyTH4, and FERM domain-containing H1 (PLEKHH1) and epididymal sperm-binding protein 1 (ELSPBP1). There were three polypeptides that were identified in G3 as UPs were: cystatin domain-containing protein (LOC607874), Family with sequence similarity 135 member A (FAM135A), and abnormal spindle-like microcephaly-associated protein homolog (ASPM).

Protein identification also resulted in seven proteins that were present in all three age groups (G1, G2, G3) and two proteins that were found in G1 and G2 (Figure 3). The seven common proteins in groups G1, G2, and G3 were lactotransferrin (LTF), actin, cytoplasmic 1 (ACTB), prostaglandin-H2 D-isomerase (PTGDS), CE10 protein (CE10), NPC intracellular cholesterol transporter 2 (NPC2), albumin (ALB), and cysteine-rich secretory protein 2 (CRISP2). The two proteins that were detected in both G1 and G2 were WAP domain-containing protein (N/A/WAPdcp) and lipocln_cytosolic_FA-bd_dom domain-containing protein (LCNL1).

#### 2.4.2. Quantitative Analysis

The study also included a semi-quantity analysis of protein content that was based on intensity measurement. When comparing the intensity of seven common proteins (presented in all groups G1, G2, and G3) among the age groups, statistical differences were found in the intensity of four proteins.

**Figure 4:**
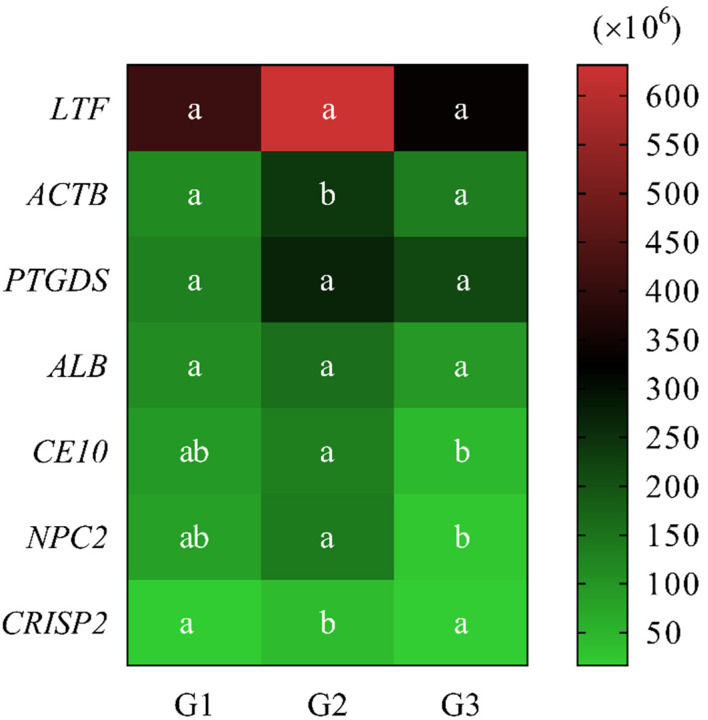
Heatmap showing the common proteins abundance (MS intensity) in epididymal spermatozoa of dogs (*Canis lupus familiaris*) in different age groups (G1, G2, G3). Different superscripts within rows (a, b) means statistically significant differences (*p* < 0.05) among the age groups.

ACTB intensity was low (*p* < 0.05) in G1 and G3 (114.6 ± 16.1 × 10^6^ and 134.7 ± 23.9 × 10^6^, respectively). It was shown that it differed statistically significantly (*p* < 0.05) when compared with G2 (240.2 ± 36.2 × 10^6^) (Figure 4).

When comparing the intensity of CE10 and NPC2 among the age groups, it was noted that the lowest (*p* < 0.05) values of CE10 (45.5 ± 9.5 × 10^6^) and NPC2 (24.8 ± 8.6 × 10^6^) were found in G3. Values differed statistically significantly (*p* < 0.05) when they were compared with G2, 132.8 ± 17.8 × 10^6^ and 140.4 ± 34.1 × 10^6^, CE10 and NPC2, respectively. The intensity of the above-mentioned proteins in G1 showed no statistically significant differences when they were compared to other age groups and were 93.7 ± 25.7 × 10^6^ and 82.9 ± 21.5 × 10^6^, respectively (Figure 4).

The CRISP2 intensity was low in G1 and G3 (17.1 ± 6.3 × 10^6^ and 16.0 ± 5.0 × 10^6^, respectively), and it differed statistically significantly (*p* < 0.05) when it was compared with G2 (40.5 ± 6.8 × 10^6^) (Figure 4).

There were no significant differences in the values among the age groups in the intensity of LTF, PTGDS, and ALB (Figure 4). The intensity in LTF and ALB ranged from 331.6 ± 77.4 × 10^6^ and 94.3 ± 21.2 × 10^6^ (G3) to 631.1 ± 109.6 × 10^6^ and 157.2 ± 92.8 × 10^6^ (G2), respectively, while the PTGDS intensity ranged from 130.2 ± 39.3 × 10^6^ (G1) to 269.9 ± 85.4 × 10^6^ (G2).

### 2.5. Western Blotting Analysis

**Figure 5:**
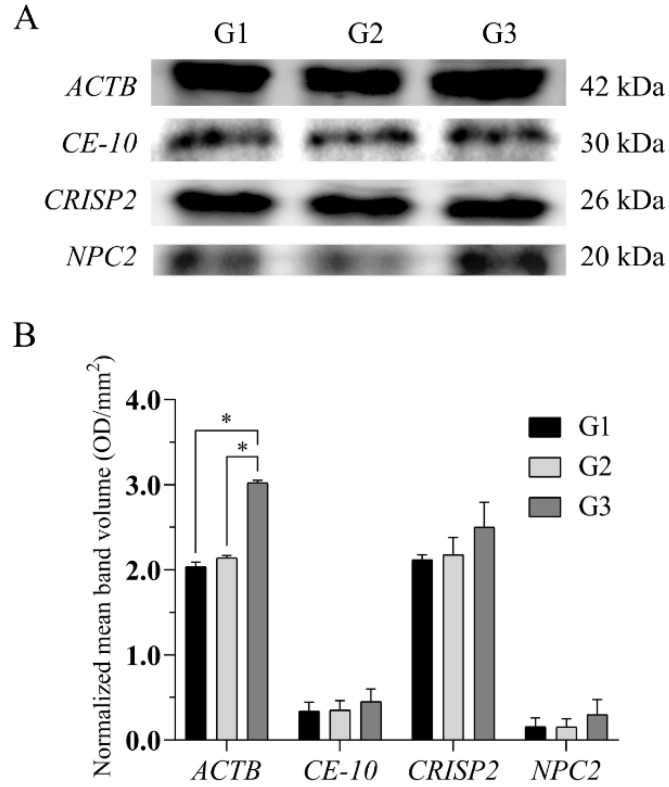
Protein expression levels in the epididymal spermatozoa of dogs (*Canis lupus familiaris*) in different age groups: G1, G2, and G3 blotted with antibodies (**A**). Each column indicates the normalized value (mean ± SE) of protein expression from three replicates. Protein expression represents band optical density (OD) which is significantly different among the age groups. * Significant at *p* < 0.05 (**B**).

Western blotting analysis showed differential protein expression in dog epididymal spermatozoa among the age groups (Figure 5A,B). Significant differences (*p* < 0.05) were found for ACTB in G3 when compared to G1 and G2 in the normalized mean band volume (Figure 5B).

### 2.6. GO Analysis, KEGG Pathways, and Functional Annotation

**Figure 6:**
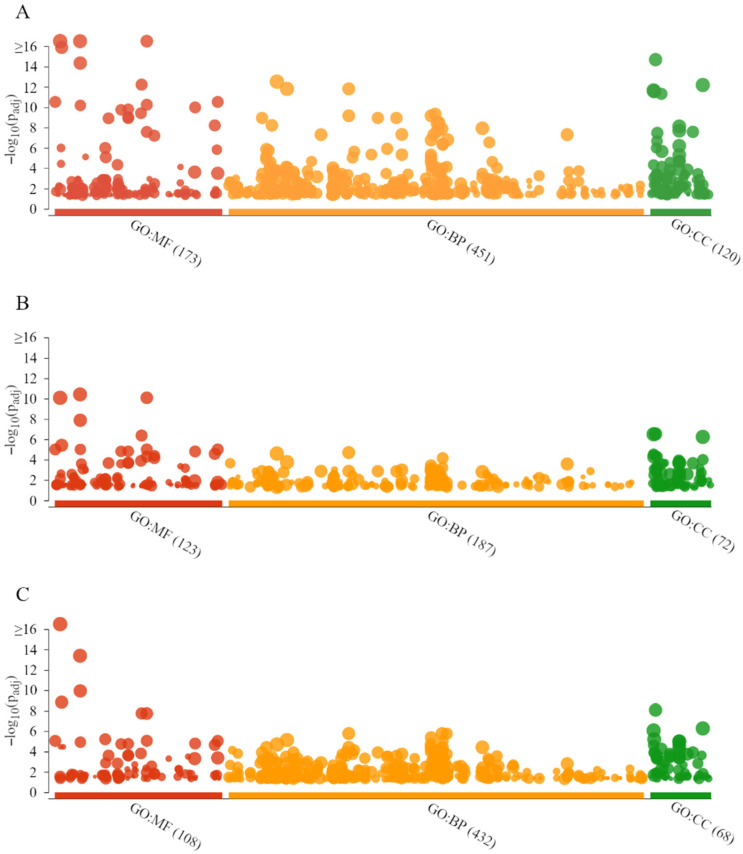
g.GOSt multi-query Manhattan plots showing the functional classification of proteins that were present in the epididymal spermatozoa of dogs (*Canis lupus familiaris*) in different age groups: G1 (**A**), G2 (**B**), and G3 (**C**). The *x*-axis represents Gene Ontology (GO) functional terms, grouped and color-coded by data sources: molecular function (GO:MF, red), biological process (GO:BP, orange), and cellular component (GO:CC, green). The *y*-axis shows the adjusted enrichment *p*-values in negative log10 scale. Circles illustrates the enrichment of GO terms and shows the *p*-value. The circle sizes are in accordance with the corresponding GO term size. The number behind the source name in the *x*-axis labels shows how many significantly enriched GO terms there were from this source.

The g.GOSt multi-query Manhattan plots of the GO analysis and KEGG pathways of G1, G2, and G3 dog epididymal spermatozoa proteins, performed with the KOBAS annotation tool (v.3.0), are shown in Figure 6A–C, respectively.

**Figure 7:**
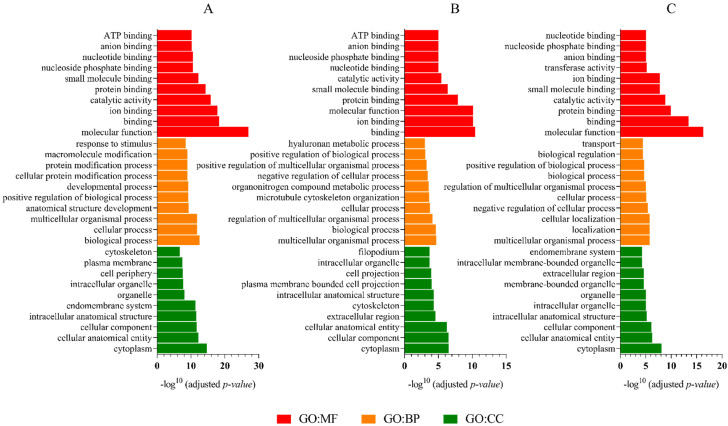
Gene Ontology (GO) of proteins that were present in the epididymal spermatozoa of dogs (*Canis lupus familiaris*) in different age groups: G1 (**A**), G2 (**B**), and G3 (**C**). There are ten highly significant GO terms for molecular function (GO:MF, red), biological process (GO:BP, orange), and cellular component (GO:CC, green) that are presented.

Gene ontology analysis showed similar main molecular functions for all the age groups. For the G1 proteins, the GO:MF terms were dominated by molecular function (LTF, CE10, PTGDS, AREL1), binding (LTF, PTGDS, ALB, SACS), and ion binding (LTF, ALB, SULF2, CYP2E1) (Figure 7A). Furthermore, for the G2 proteins, the GO:MF terms were represented by binding (LTF, PTGDS, ALB, ASPM), ion binding (LTF, ALB, MYO1D, CDC42BPA), and molecular function (LTF, PTGDS, CE10, ALB) (Figure 7B). Whereas for the analysis of the G3 proteins, GO:MF terms were dominated by molecular function (LTF, ASPM, TRPV1, PTGDS), binding (LTF, ASPM, TRPV1, PTGDS), and protein binding (ASPM, TRPV1, CBL, ALB) (Figure 7C).

Gene ontology analysis showed similar biological processes for all the age groups. For the G1 proteins, the GO:BP terms were represented by biological processes (LTF, NPC2, PTGDS, AREL1), cellular processes (LTF, NPC2, PTGDS, AREL1), and multicellular organismal processes (LTF, PTGDS, PLCD3, AFP) (Figure 7A). Furthermore, for the G2 proteins, the GO:BP terms were dominated by multicellular organismal processes (LTF, PTGDS, ASPM, CEMIP), biological processes (LTF, PTGDS, ALB, NPC2), and regulation of multicellular organismal processes (LTF, PTGDS, ASPM, CLU) (Figure 7B). However, for the G3 protein analysis, GO:BP terms were represented by multicellular organismal processes (LTF, ASPM, TRPV1, PTGDS), localization (ASPM, TRPV1, CBL, ALB), and cellular localization (ASPM, TRPV1, ALB, CLU) (Figure 7C).

Gene ontology analysis showed the same main cellular component for all the age groups. For the G1 proteins, the GO:CC terms were dominated by cytoplasm (LTF, AREL1, ALB, SACS), cellular anatomical entity (LTF, CE10, PTGDS, AREL1), and cellular component (LTF, CE10, PTGDS, AREL1) (Figure 7A). Furthermore, for the G2 proteins, the GO:CC terms were represented by cytoplasm (LTF, ALB, ASPM, CEMIP), cellular component (LTF, PTGDS, CE10, ALB), and cellular anatomical entity (LTF, PTGDS, CE10, ALB) (Figure 7B). However, for the G3 protein analysis, GO:CC terms were dominated by cytoplasm (LTF, ASPM, CBL, ALB), cellular anatomical entity (LTF, ASPM, TRPV1, PTGDS), and cellular component (LTF, ASPM, TRPV1, PTGDS) (Figure 7C).

Pathways analysis using the Kyoto Encyclopedia of Genes and Genomes (KEGG) showed mainly protein export for the G1 group (SRP14, SEC61A1, SRPRA), protein processing in the endoplasmic reticulum (TUSC3, SEC24D, MAPK10, RRBP1, SEC61A1, ERN1), and Ras signaling pathway (RGL2, TIAM1, GRB2, MAPK10, ZAP70, ABL1). For the G2 group, the main pathways were: Salmonella infection (NFKB1, TJP1, DYNC2H1), transcriptional misregulation in cancer (MET, MLLT1, NFKB1), and adherens junction (MET, TJP1). For the G3 group, the main metabolic pathways were established as mucin-type O-glycan biosynthesis (GALNT6, GALNT18), porphyrin and chlorophyll metabolism (BLVRA, CP), and cell cycle (SMC1B, ANAPC11, MCM2) (Table 3).

## 3. Discussion

Age-dependent changes in males (e.g., increase in systemic diseases, infections, abnormalities in testis structure, and lower sex hormones) may influence the quality of epididymal sperm [19,20].

This study aimed to verify the possible influence of the aging process on dog epididymal spermatozoa quality and on their proteome characteristics. According to the authors’ knowledge, this is the first study to investigate the influence of dog age on the epididymal sperm proteome.

The aging process in semen has been mostly investigated in humans, and a significantly lower ejaculate volume [20,21,22], a decrease in sperm with progressive motility [21,22], and a higher percentage of sperm DNA fragmentation index [20] were found. In this study, it was shown that the motion parameters of the dog’s epididymal sperm, such as TMOT, PMOT, VAP, VCL, and VSL, decreased with age. These findings are in agreement with the results of Verón et al. [23], who have shown that several sperm quality parameters for men’s ejaculate, such as VSL, VCL, VAP, BCF, and ALH, were also negatively affected by age. Bhanmeechao et al. [7] also have shown that dog age was negatively correlated with epididymal sperm motility, sperm vigor, and viability. Additionally, those authors described a positive correlation between male dog age and the percentage of sperm defects [7]. Similar results were presented in the current study. The highest percentage of morphological damage was noted in the epididymal sperm midpiece. This may indicate disturbances in the ATP production in aging sperm. Negative changes in dog ejaculated sperm morphology followed by male age were also shown by Rijsselaere et al. [5] and Rota et al. [6]. Lechner et al. [24] showed that the percentage of progressively motile, membrane-intact, and morphologically normal spermatozoa was the lowest in 10 and 11-year-old dogs.

The above studies clearly showed that senescence in dogs was associated with a decrease in the functional quality of the epididymal sperm, and consequently, this will be transferred on to the ejaculated sperm quality, which may result in reduced fertility or even infertility.

Much more research is needed on studying sperm proteomics, which would allow a better understanding of the molecular events affecting the biological functions of the reproductive cells [25]. The first step to proteome analysis is usually protein electrophoresis. To date, SDS-PAGE of the dog epididymal spermatozoa proteins according to age has not been shown. In this study, there were no differences that were found in the number of protein fractions or in the range of molecular weights in the gel image presenting epididymal sperm proteins depending on the age of the dog. Only the 68 kDa protein showed changes in their intensity depending on the age group. According to the authors’ earlier results, this might be lactotransferrin or carboxylesterase 5A [26]. Lactotransferrin showed differences in the abundance in epididymal sperm from different age groups, but it was not statistically significant. The authors suggested that LTF that was produced and secreted in the dog epididymis may coat epididymal sperm for protection [26]. This phenomenon may also be age-dependent.

Baker et al. [27] used two-dimensional gel electrophoresis (2DE) to investigate changes in rat sperm proteomes during epididymal maturation, and Asano et al. [28] used iTRAQ mass spectrometry to characterize membrane fractions in murine sperm. The rat and mouse sperm proteomes were characterized by 2DE and LC-MS/MS identification [29,30]. The dog ejaculated spermatozoa proteome was characterized by Araujo et al. [31,32] using mass spectrometry.

Based on the authors’ knowledge, this is also the first study concerning a mass spectrometry analysis of the proteins that are present in dog epididymal spermatozoa according to age, using liquid samples. The epididymal sperm proteome was characterized for different animal species, such as the horse [33], bull [34], swine [35], and mice [36]. In a recent study by the authors of fractionated canine epididymal sperm proteins [26], mass spectrometry identification resulted in a total of 195 proteins that were extracted from the gel. That may be a small number when compared with a liquid sample analysis involving 1772 proteins that were identified in all age groups. As can be noted, an analysis of liquid samples might provide much more information than an analysis of protein samples that were extracted from the protein bands from a gel, even for the same biological material.

Knowledge of the impact of aging processes on male dog fertility is limited. Brito et al. [37] indicated that aging processes have an adverse effect on spermatogenesis and sperm maturation in the epididymis. However, a proteomic study of the influence of aging processes on the sperm proteome in the dog has not yet been conducted. In this study, the whole proteome profiling of the dog’s epididymal spermatozoa divided into age groups showed differences in the total number of peptides that were identified.

A total of four common proteins (ACTB, CE10, NPC2, CRISP2) showed statistically significant different expressions according to the dog’s age. Their presence in the canine epididymal sperm was confirmed using Western blotting. Low levels of ACTB, CE10, NPC2, and CRISP2 in the epididymal sperm may be associated with incomplete maturity or aging of the dogs. However, a large-scale study is needed to confirm the potential of these proteins as epididymal sperm aging markers.

ACTB is responsible for cell volume regulation, and it builds the cytoskeleton of sperm cells [38]. It is localized in the flagellar and acrosomal membrane of the spermatozoa. Its role in sperm motility and capacitation was proposed [39,40,41].

NPC2 intercellular cholesterol transporter 2, also called epididymal secretory protein mRNA was found in high amounts in the epididymal duct epithelium, while the protein was found in the duct lumen [42]. NPC2 form was found on the acrosome and equatorial region of the sperm [43]. It is implicated in cell cholesterol metabolism [44].

CRISP2 is a cysteine-rich secretory protein (CRISP) family member. A decrease in CRISP2 amount in sperm is associated with male infertility [45,46,47]. It is known to be a part of the sperm acrosome and sperm tail [48,49,50,51].

All three proteins (ACTB, NPC2, CRISP2) were earlier found in dog epididymal spermatozoa and described in detail in a previous study by the authors [26].

The pattern of cysteine residues indicates that CE10 is similar to the epididymal CE4 protein [2]. Closely related gene products were abundant in the epididymis of stallions and bulls but not in rodents or men [52]. It was not found in dogs. The described protein may act as extracellular proteinase inhibitor [53].

A qualitative analysis of proteins showed the presence of two proteins that were unique for the group of the youngest dogs. The proteins were glutathione peroxidase (GPx) and hyaluronoglucosaminidase (CEMIP). GPx is a well-known, highly abundant protein in the canine epididymis [54] and was described in detail in a recent study by the authors [26]. CEMIP (also named KIAA1199) is expressed in the human testis [55]. It exerts significant changes in cell morphology and actin cytoskeletal dynamics and mediates its effects through the cooperative regulation of the canonical Wnt and P38/MAPK signaling [56]. The biological role of CEMIP has been studied in cancer biology in humans, but it has not been described in other species including the dog. The expression of CEMIP may be regulated depending on whether the cells are mortal or immortal rather than how old the cells are [55].

A total of five unique proteins were identified in the middle-aged dog group: endo-β-N-acetylglucosaminidase (ENGASE), epididymal sperm-binding protein 1 (ELSPBP1), inactive ribonuclease-like protein 9 (RNASE9), clusterin (CLU), and pleckstrin homolog, MyTH4 and FERM domain-containing H1 (PLEKHH1).

ENGASE is one of the key enzymes in the processing event of free oligosaccharides in the cytosol [57]. Recently reported evidence suggests that the enzyme can also directly act on misfolded N-glycoproteins [58]. The ENGASE gene exhibited a broad tissue distribution [57], but it was not characterized in the reproductive organs or canine spermatozoa.

ELSPBP1 was first described in humans and dogs as a sperm-binding protein of epididymal origin [59]. Since then, orthologs have been identified in horse [60], pig [61,62], and bovine [63] models. ELSPBP1 binds to the spermatozoa during their transit through the epididymis [63]. More recently, ELSPBP1 was shown to negatively correlate with bull fertility [64] and was, in fact, associated with the sperm population that was already dead before ejaculation [65].

RNASE 9 may be synthesized and secreted by principal cells of the epididymis and may bind to spermatozoa when they are passing by. Therefore, human RNASE 9 may be a sperm maturation-related protein [66]. Liu et al. [67] demonstrated that RNASE 9 protein inhibited sperm capacitation and acrosome reaction in humans. Epididymis-specific and androgen-dependent RNASE9 expression was shown in rats [68] and in mice [69]. This enzyme also exhibited antibacterial activity [66]. RNASE 9 presence in the canine epididymal spermatozoa was established for the first time in the current study. It might also act as an antibacterial factor and sperm capacitation inhibitor in canine sperm.

CLU is an extracellular chaperone that is known to be secreted by stallion testes [70] and epididymides [71] in a high amount. It is overexpressed in several human cancers such as prostate cancer [72]. CLU participates in sperm maturation by affecting lipid transport and membrane remodeling [72]. Morphologically defected sperm extensively bind CLU to its plasma membrane [73]. CLU presence in the canine epididymal spermatozoa was established for the first time in the current study.

PLEKHH1 is a protein that is involved in intracellular signaling or as constituents of the cytoskeleton [74]. It can bind phosphatidylinositol lipids within biological membranes. Through these interactions, PLEKHH1 plays a role in recruiting proteins to different membranes. The protein expression was found in human testis, epididymis, seminal vesicles, and prostate [75]. However, although its presence was shown [26], its function in canine epididymal tissues or sperm has not yet been established.

In the oldest dog group (G3), three proteins were identified as unique: Cystatin domain-containing protein (LOC607874), Family with sequence similarity 135 member A (FAM 135A), and abnormal spindle-like microcephaly-associated protein homolog (ASPM). These proteins were not shown in dog tissues or spermatozoa until now.

*Canis lupus familiaris* cystatin-C-like (LOC607874) mRNA was found as recorded in the NCBI. A cystatin-related epididymal-specific (CRES) gene was found in the mouse epididymis, showing homology with those of well-established protein inhibitors (cystatins) [76]. The CRES gene is almost restricted to the epididymis and much less expressed in the testis, may be expressed in spermatids [77].

FAM 135A function is closely related to the regulation of cellular proliferation, differentiation, development, and cellular growth control [78]. The protein expression was found in human tissue: testis, epididymis, prostate, and seminal vesicle [79].

ASPM is expressed in a variety of embryonic and adult tissues and is upregulated in cancer [80]. A lack of a functional ASPM may affect the fidelity of chromosome segregation which leads to a reduced ability of fetal stem cells to produce neurons [80]. ASPM possesses a role in sperm flagellar function [81].

These three above-mentioned proteins may be potentially epididymal sperm aging markers, but a much broader study is needed to confirm these findings.

A gene ontology analysis showed the main function of the analyzed sperm proteins to be binding, independent of age groups. This might underline the importance of epididymal sperm protein affinity to other proteins or ions as one of the sperm function regulating mechanisms. This is a well-documented issue for both animal and human ejaculated sperm [82]. However, less is known about epididymal sperm in this regard. Epididymal sperm protein functions that were found in each of the three age groups were mainly involved in the regulation of biological processes, cellular processes, multicellular organismal processes, and cellular localization. Using a cellular components analysis for all three age groups, it was found that epididymal sperm proteins were derived mainly from the cytoplasm and cellular anatomical entities.

A pathway analysis using KEGG for the youngest dogs mainly showed protein export, protein processing in the endoplasmic reticulum, and the Ras signaling pathway.

Most secretory and membrane-bound proteins are co-translationally translocated. Proteins that reside in the endoplasmic reticulum (ER), Golgi, or endosomes also use the co-translational translocation pathway [83], even though most secretory proteins are co-translationally translocated. In addition, proteins that are targeted to other cellular destinations, such as mitochondria, chloroplasts, or peroxisomes, use specialized post-translational pathways [83]. These processes seem to be intensified in the epididymal spermatozoa of young dogs.

The Ras signaling pathway is one of the main pathways to transduce intracellular signals in response to mitogens controlling cell growth, survival, and anti-apoptotic programs. Ras, a low-molecular-weight GTP-binding protein, plays a key regulatory role in many biochemical processes. The presence of Ras was shown in hamster testicular, caput and cauda epididymal spermatozoa [84]. The interaction of Ras with both PI3-kinase and PKC suggests that Ras may regulate several signaling pathways in spermatozoa [84]. It has been recently reported that Rab 2A and 3A (members of the Ras family) are related to acrosomal exocytosis in spermatozoa, and Rab 2A can be used as a fertility-related biomarker in males [85]. Rab proteins were located in the sperm head and tail. Ras is correlated with various sperm motility patterns and motion kinematics before capacitation [86].

For the middle-aged dogs, the main established pathways were: salmonella infection, transcriptional misregulation in cancer, and adherens junction.

In tumor cells, genes encoding transcription factors (TFs) are often amplified, deleted, rearranged via chromosomal translocation and inversion, or subjected to point mutations that result in a gain or loss of function. Similar processes might be connected with aging in a sperm cell.

Adherens junctional complexes, such as ectoplasmic specializations, facilitate cellular interactions that are critical for both adhesion and signaling between Sertoli cells and germ cells [87]. Previous studies indicated that Sertoli cell-germ cell adherens junctions undergo extensive restructuring to promote germ cell maturation and spermation [87,88].

For the oldest dogs, the main metabolic pathways were established as mucin-type O-glycan biosynthesis, porphyrin and chlorophyll metabolism, and cell cycle.

Mucin-type O-glycosylation is a protein modification that is present on membrane-bound and secreted proteins [89]. Mucins bind bacteria and viruses, and also function as receptors for carbohydrate-binding proteins. Mucin-type O-glycan biosynthesis was intensified in the spermatozoa of obese rats [90].

Metal complexes of porphyrins play important biological roles. A porphyrin- and chlorophyll-metabolism-enriched pathway was found in goat seminal plasma [91]. However, little is known about porphyrin metabolism in canine epididymal sperm.

The cell cycle includes the mitotic and meiotic cell cycle [92]. The meiotic proteins might be remnants of spermatogenesis, with no function in mature sperm [92].

In a study by Silva et al. [4], the activity of 12 proteins in human ejaculated spermatozoa was correlated with male age. Of those, half of them were the main components of the mammalian target of the rapamycin (mTORC1) signaling pathway.

It seems that in different dog ages, different metabolic pathways in epididymal spermatozoa are intensified, which might be connected with the aging processes.

In order to analyze the influence of male age on the epididymal semen, parameters such as sperm motility, membrane condition, antioxidant status, and chromatin status are usually examined. The current study additionally investigated the influence of age on the proteome of the dog’s epididymal sperm and its metabolic pathways, which provides a broader point of view on the aging process.

## 4. Materials and Methods

The study was performed under the guidance of Directive 63/2010/EU and the Journal of Laws of the Republic of Poland (2017) regarding the protection of animals that are used for scientific or educational purposes. The exemption letter was obtained from the Local Ethics Committee for Animal Experimentation, Olsztyn, Poland (LKE/01/2022). The authors have permission to conduct animal experiments according to the Polish Laboratory Animal Science Association (Numbers: 1432/2015; 1508/2015).

### 4.1. Chemicals and Media

All chemicals of the highest purity were purchased from the Sigma Chemical Company (St. Louis, MO, USA) unless otherwise stated.

### 4.2. Animals

The study was performed on 30 mixed-breed dogs that were divided into three age groups according to the study by Ortega-Pacheco et al. [93], with modifications: G1 (young, 12 to 41 months old; n = 10); G2 (adult, 42 to 77 months old, n = 10); and G3 (old, 78 to 132 months old, n = 10) of unknown fertility. The weight of the dogs was from 9 to 28 kg (mean 16.5 kg) in G1, from 15 to 30 kg (mean 20.8 kg) in G2, and from 17 to 30 kg (mean 23.9 kg) in G3. Only dogs for whom the authors were able to prove animal age documented information were taken into consideration. The dogs were fed and kept in the same environmental conditions in the Shelter for Homeless Animals in Tomaryny (Poland). All of the dogs were presented for a routine orchiectomy by a qualified veterinary doctor as a part of a program to prevent animal homelessness and promote adoption. The consent form was achieved from the director of the shelter.

### 4.3. Cauda Epididymal Semen Collection

The materials, i.e., the testis with the epididymis, were placed in sterile plastic containers in 0.9% NaCl solution and then in a thermobox at a temperature of 4 °C and delivered within one hour to the laboratory of the Department of Animal Biochemistry and Biotechnology (University of Warmia and Mazury in Olsztyn, Poland). Immediately after that, the gonads were washed with DPBS (Dulbecco’s Phosphate-Buffered Saline, Gibco, Grand Island, NY, USA). The cauda epididymal tissue was cut carefully with a sterile scalpel to avoid sectioning the blood vessels. The effluent of the epididymal semen was aspirated from the cauda epididymal tissue using an automatic pipette [94] with modification. The samples that were obtained from the cauda epididymis (right and left) of the same animal were pooled.

### 4.4. Spermatozoa Quality Assessment

The epididymal sperm concentration was determined using a Bürker chamber under a light microscope (Olympus BX41TF, Tokyo, Japan).

The sperm samples were subsequently assessed using a computer-assisted semen analysis (CASA-system, HTM-IVOS, 12.3, Hamilton-Thorne Biosciences, Beverly, MA, USA). The procedure was described previously by Mogielnicka-Brzozowska et al. [95]. The following software settings that were recommended by the manufacturer for canine sperm analyses were used: frame acquired—30, frame acquisition rate—60 Hz, minimum cell contrast—75, minimum cell size—6 pixels, straightness threshold—75%, path velocity threshold—100 μm/s, low average path velocity (VAP) cut-off—9.9 μm/s, low straight-line velocity (VSL) cut-off—20 μm/s, static size gates—0.80–4.93, static intensity gates—0.49–1.68, and static elongation gates—22–84. The total motility (TMOT, %), progressive motility (PMOT, %), average path velocity (VAP, µm/s), straight-line velocity (VSL, µm/s), curvilinear velocity (VCL, µm/s), the amplitude of lateral head displacement (ALH, µm), beat cross frequency (BCF, Hz), straightness (STR, %), and linearity coefficient (LIN, %) were analyzed in each epididymal sperm sample.

### 4.5. Morphology Assessment of Epididymal Spermatozoa

The dog epididymal spermatozoa were prepared as smears on glass slides using 10 μL of each sample (1 × 10^8^ spermatozoa) and left to dry on a thermoblock (5 min, 37 °C). Spermac Stain™ (FertiPro, Beernem, Belgium) staining was then performed according to the manufacturer’s recommendations with modifications. The morphological structures in the dog epididymal spermatozoa were examined under bright light microscopy at the magnification of 1000× (Olympus BX41TF). Approximately 200 spermatozoa were counted in each sperm sample. The spermatozoa were classified into two categories: normal (without defects) or damaged (at least one defect), according to the World Health Organization guidelines [96]. During the determination of the sperm head defects, attention was paid to the shape of the acrosome and continuity of the membranes surrounding the sperm nucleus. A normal head was smooth and regularly contoured, the midpiece was slender and regular, and the tail was smooth along its length and thinner than the midpiece. According to the protocol of Spermac Stain™ (FertiPro), the epididymal sperm fragments were stained as follows: the acrosome (dark green), the nucleus (red), the equatorial region (pale green), and the midpiece and the tail (green).

### 4.6. Preliminary Sample Preparation

After the epididymal semen quality assessment, the sperm samples were centrifuged at 800× *g* for 10 min at 4 °C to remove epididymal fluid (EF). The remaining supernatant (EF) was removed, and the sperm pellet was resuspended in 1 mL DPBS (Gibco) and again centrifuged at 800× *g* for 10 min at 4 °C to remove loosely bound proteins [97]. The remaining supernatant was removed and discarded. Each of the sperm samples was split in half. One half was used for the electrophoretic separation, and the other half was stored for 2 weeks at −80 °C for further LC-MS analysis.

#### 4.6.1. Preliminary Sample Preparation for Protein Analysis

The epididymal spermatozoa samples were placed in an ice bath and subjected to sonication using the Omni Sonic Ruptor 250 Ultrasonic Homogenizer (Omni International, Kennesaw, GA, USA) with the following parameters: 150 W for 10 min with a frequency of 60 kHz. Following sonication, the sperm samples were centrifuged (8000× *g* for 10 min at 4 °C). The resulting supernatant containing sperm intracellular proteins (SIPs) was collected into another Eppendorf tube. An aliquot of 1 mL of Radioimmunoprecipitation Assay Buffer (RIPA) containing 50 mM Tris-HCl; 150 mM NaCl; 1% (*v*/*v*) Triton X-100; 0.5% sodium deoxycholate; 0.1% SDS; and ddH2O, pH 7.4 was added to the remaining sperm pellet and the sample was incubated for 5 min at 4 °C and was then vortexed and left overnight in a buffer [95,98] with modifications. Protease Inhibitor Cocktail (Sigma-Aldrich/P8340, St. Louis, MO, USA) was added both to the SIPs and the remaining sperm pellet. The samples were then centrifuged at 8000× *g* for 10 min, 4 °C, to obtain a clear lysate of the sperm membrane-associated protein fraction (SMAPs). The clear lysate was collected into another Eppendorf tube and mixed together with SIPs to get a whole protein set of an epididymal sperm cell (sperm extracts—SE) and was then frozen and kept at −80 °C until further analyses.

#### 4.6.2. Total Protein Content Measurement

The total protein content was measured using Bradford Reagent (Sigma-Aldrich/B6916) in SE.

### 4.7. Polyacrylamide Gel Electrophoresis (SDS-PAGE)

For proteomic analysis, the SE that were isolated from the epididymal spermatozoa of individual dogs were pooled according to age group (G1, G2, G3). Each pool was run in triplicate (technical replicate). Each well was loaded with 50 μg of protein in solution. The SDS-PAGE procedure was previously described by Mogielnicka-Brzozowska et al. [95]. The molecular weight (MW) and the optical density (OD) of the stained protein bands (PB) were determined using MultiAnalyst 1.1 software (BioRad, Laboratories, Hercules, CA, USA). In the current experiment, proteins in the gel were not analyzed by mass spectrometry.

### 4.8. Western Blotting Analysis

Sperm extract protein samples containing exactly 50 μg were separated by 12% SDS-PAGE and transferred to Immobilon-P polyvinylidene fluoride (PVDF) membranes (Millipore, Bedford, MA, USA). Electroblotting was carried out for 1 h at 300 mA, according to the previously described method [99].

After blocking non-specific binding sites with 5% nonfat milk in Tris-buffered saline, TBS (1 M Tris, 5 M NaCl, pH 8.0), containing 0.05% (*v*/*v*) Tween 20, TBST (MP Biomedicals LLC, Santa-Ana, CA, USA), the blots were incubated with one of the following primary antibodies (Thermo Fisher Scientific, Waltham, MA, USA): beta Actin rabbit polyclonal antibody (PA5-85271; 1:500), Bcl-10 rabbit polyclonal antibody (PA5-85359; 1:250), NPC2 rabbit polyclonal antibody (PA5-51463; 1:500), and CRISP rabbit polyclonal antibody (PA5-97621; 1:1000). The gene name Bcl-10 was used as the alias name for CE10. According to the manufacturer’s information, it is an antibody that recognizes CE10. Following incubation with the primary antibodies overnight at 4 °C, the membranes were washed and incubated for 1 h at room temperature with Peroxidase AffiniPure Goat Anti-Rabbit secondary antibody (111-035-003; 1:20,000; Jackson ImmunoResearch, Baltimore Pike, PA, USA), developed with enhanced chemiluminescence ServaLight CL EOS Substrate kit (Serva, Heidelberg, Germany), and scanned with the ChemiDoc™ Touch Imaging System (BioRad Laboratories). The molecular weights of the protein were determined using the molecular weight standard (PageRuler™ Prestained Protein Ladde, Catalog Number 26,617, Thermo Fisher Scientific). Protein bands from the scanned images were quantified using MultiAnalyst 1.1 software (BioRad Laboratories). The signal intensities were normalized to the total protein by staining membranes with Coomassie Blue. The values were expressed as the total signal intensity inside the boundary of a band that was measured in pixel intensity units/mm^2^—optical density (OD).

### 4.9. Identification of Proteins in Liquid Samples by Mass Spectrometry

#### 4.9.1. Sample Preparation for LC-MS Analysis

Sperm cell samples were extracted as follows. The frozen samples were put on ice, and 800 µL of MTBE: MeOH mixture (3:1) was added to each Eppendorf tube. The extraction mixture was pre-cooled to −18 °C and dispensed as quick as possible. Next, the samples were sonicated in an ice-water-filled sonic bath for 15 min. To separate the polar and non-polar metabolites, 400 µL of water and a MeOH mixture was added and was well vortexed. The extracted samples were placed in a pre-cooled centrifuge (4 °C) and spun for 7 min at 21,000× *g*. The upper non-polar phase for analysis of lipids was collected into a new tube. The interphase containing residual upper and some of the lower phase was discarded. After a short centrifugation (5 min), the lower methanol-water phase was transferred into a new tube. Finally, the residual methanol-water phase was discarded, and the pellets were frozen at −80 °C. The collected liquid extracts were dried in a speed-vac and stored at −80 °C prior to the LC-MS analysis. The MTBE: MeOH mixture contained 12.5 µM D7-Arginine and 100 ng/mL D70-Phosphatidylcholine (36:0) as internal standards for the polar and non-polar phases, respectively.

#### 4.9.2. In Solution Digestion

The protein pellets were dissolved in 10 µL 6M Guanidine-HCl in a 25 mM bicarbonate ammonium solution (pH 8.0). A total of 1 µL of 200 mM DTT in 25 mM bicarbonate ammonium solution (pH 8.0) was then added to each sample and incubated for 30 min at 37 °C. After protein reduction, 10 µL of 200 mM iodoacetamide in 25 mM bicarbonate ammonium solution (pH 8.0) was added for alkylation. Each sample was gently vortexed and incubated in the dark for 1 h at room temperature. A total of 25 mM bicarbonate ammonium solution (pH 8.0) was then added in order to reduce the guanidine-HCl concentration to 0.6 M. Protein digestion was performed by adding a trypsin solution with a final ratio of 1:25 (trypsin:protein), and the samples were incubated overnight in 37 °C with gentle vortexing. After trypsinolysis, 1% formic acid was added to adjust the pH to 3–4. The samples were then desalted on Pierce™ C18 Spin Columns (Thermo Fisher Scientific), vacuum-dried, and resuspended in acetonitrile.

#### 4.9.3. NanoUPLC-Q-TOF/MS Analysis

Waters Acquity liquid chromatography M-Class system (Waters Corp., Milford, MA, USA) equipped with a Peptide BEH C18 analytical column (150 mm × 75 µm; 1.7 µm, Waters Corp.), and Symmetry C18 precolumn (180 µm x 20 mm; 1.7 µm, Waters Corp.) was performed to separate the digested samples.

Each sample was injected into the precolumn and then washed with 99% solvent A (0.1% formic acid in water) at a flow rate of 5 µL/min for 5 min. After washing, the peptides were transferred to an analytical column and separated. The flow rate of the mobile phase was 300 nL/min. The total run time of the analytical gradient, including the column equilibration step, was set at 75 min. The elution gradient steps were as follows: from 0 to 2 min, 5% B (0.1% formic acid in acetonitrile); from 2 to 15 min, 5% to 30% B; from 15 to 45 min, 30% to 60% B; from 45 to 48 min, 60% to 85% B; 10 min, 85% B; and from 58 to 58.5 min, the B concentration dropped from 85% to 5%.

A mass spectrometry (MS) analysis was performed using Synapt G2-Si (Waters Corp., Milford, MA, USA) with a nano-electrospray ionization (nESI) source, operating under a positive ion mode. The capillary voltage was set at 3.0 kV, and the cone voltage was set at 40 V. The cone gas flow was set at 40 L/h, and the source temperature was set at 100 °C. The nanoflow gas flow was set at 0.2 Bar. Data were acquired for m/z 70 to m/z 1800 using data-independent mode (MSE). Leucine enkephalin (m/z 556.2771) was used as a Lockspray. The lock mass was acquired every 45 s, and a mass correction was applied automatically during acquisition.

Raw chromatography files were analyzed with Byonic software (Protein Metrics, Cupertino, CA, USA). The following settings were used for peak picking and identification: trypsin digestion, max. two miss-cleavages, max. three charges, possible modifications: carbamidomethylated Cys; oxidation of Met and Trp; dioxidation of Trp; pyro-Glu; de-carbamidomethylated Cys; oxidation of Pro; phosphorylation of Ser, Tyr, Thr; (di)methylation of Lys and Arg; acetylation of Lys; trimethylation at Lys; sulfation of Cys, Ser, Thr, Tyr. The detected peptides were compared to the SWISSPROT dog proteome (CANLF)—that was downloaded April 2021. False identifications were limited by comparison with common contaminants and decoys that were obtained by reverse amino acid sequencing in silico-cleavage peptide models. The total intensity was a sum of all the peak intensities over all MS/MS spectra. The protein *p*-value is the likelihood of the peptide-spectrum matches (PSMs) to this protein (or protein group) arising by random chance, according to a simple probabilistic model. A log *p*-value of −3.0 corresponds to a protein *p*-value of 0.001, or one chance in a thousand.

### 4.10. Gene Ontology and Functional Annotation

The functional enrichment of proteins that were present in the dog (*Canis lupus familiaris*) epididymal spermatozoa according to age groups (G1, G2, G3) in Gene Ontology (GO) categories: molecular function (GO:MF), biological process (GO:BP), and cellular component (GO:CC), was performed with the g:Profiler online tool, (https://biit.cs.ut.ee/gprofiler/gost, accessed on 26 April 2022). Annotations were performed with the *Canis lupus familiaris* database, using the false discovery rate (FDR) of the Benjamini–Hochberg (BH) method for the significance threshold. The user threshold was 0.05. The significance of the dog (*Canis lupus familiaris*) epididymal spermatozoa proteins were analyzed in groups (G1, G2, G3) with different male ages in the Kyoto Encyclopedia of Genes and Genomes (KEGG) pathway database and the KOBAS 3.0 (http://kobas.cbi.pku.edu, accessed on 10 February 2022) protein functional annotation tool [100]. A Venn diagram was constructed using a web tool (http://bioinformatics.psb.uugent.be/webtools/Venn, accessed on 23 November 2021).

Heatmaps, GO plots, and morphological characteristic plots were performed using GraphPad Prism software (GraphPad Prism v.9.2.0. for Windows, GraphPad Prism software, San Diego, CA, USA).

### 4.11. Statistical Analysis

The data analysis was carried out using Statistica version 13.1 (StatSoft, TIBCO Software Inc., Palo Alto, CA, USA). The results are presented as the means and standard error (mean ± SE). The sperm motility, morphology, protein intensity, and comparison of the OD values were analyzed with Tukey’s HSD test to detect the significant differences among the age groups. The values were considered to differ significantly at *p* < 0.05.

## 5. Conclusions

In conclusion, the present study demonstrated that aging in dogs was associated with diminished functional quality of the cauda epididymal spermatozoa. Differences in the sperm proteome composition were shown for the young, middle-age, and old dogs, which was followed by changes in the sperm metabolic pathways, which might influence the sperm fertilizing ability. The findings of the current study involving maturation and aging protein markers may be applicable to clinical andrology and to improving canine reproductive technologies.

## Figures and Tables

**Table 1:** Motion parameters of the epididymal spermatozoa of dogs (*Canis lupus familiaris*) in different age groups (G1: 12–41 months old; G2: 42–77 months old; G3: 78–132 months old).

Sperm Parameters	G1 (n = 10)	G2 (n = 10)	G3 (n = 10)	*p*-Value
Concentration (×10^8^/mL)	38.2 ± 3.2 ^a^	27.7 ± 3.7 ^ab^	24.8 ± 3.9 ^b^	0.018
Total motility (TMOT, %)	92.8 ± 0.3 ^a^	89.1 ± 0.9 ^a^	81.3 ± 2.4 ^b^	0.002
Progressive motility (PMOT, %)	57.7 ± 2.7 ^a^	53.8 ± 2.3 ^a^	38.5 ± 5.8 ^b^	0.007
Average path velocity (VAP, µm/s)	137.7 ± 3.4 ^a^	132.5 ± 3.0 ^a^	113.5 ± 7.3 ^b^	0.002
Straight line velocity (VSL, µm/s)	114.0 ± 3.5 ^a^	110.1 ± 2.5 ^ab^	93.0 ± 7.5 ^b^	0.003
Curvilinear velocity (VCL, µm/s)	206.6 ± 5.9 ^a^	198.0 ± 8.0 ^ab^	174.2 ± 9.3 ^b^	0.014
Amplitude of lateral head displacement (ALH, µm)	7.1 ± 0.3 ^a^	6.8 ± 0.3 ^a^	6.5 ± 0.4 ^a^	0.430
Beat cross frequency (BCF, Hz)	16.2 ± 1.0 ^a^	17.2 ± 1.4 ^a^	19.8 ± 2.0 ^a^	0.115
Straightness (STR, %)	81.9 ± 1.3 ^a^	82.1 ± 1.1 ^a^	80.5 ± 2.2 ^a^	0.354
Linearity (LIN, %)	57.8 ± 2.3 ^a^	58.2 ± 2.2 ^a^	54.5 ± 3.4 ^a^	0.234

Values are represented as the mean ± SE. Different superscripts (a, b) with the same row indicate significant differences at *p* < 0.05.

**Table 2:** Average optical density (OD) values (mean ± SE) of differentially expressed proteins (DEPs) of the cauda epididymal spermatozoa of dogs (*Canis lupus familiaris*) in different age groups (G1: 12–41 months old; G2: 42–77 months old; G3: 78–132 months old). Different superscripts (a, b) within the same column indicate significant differences (*p* ≤ 0.05) among the fractions. DEPs are marked with letters from A to C. MW—average molecular weight.

Age Groups	Protein Bands	MW (kDa)	OD ± SE
G1	A	68.48	0.32 ± 0.01 ^a^
G2	B	68.37	0.25 ± 0.01 ^b^
G3	C	68.07	0.33 ± 0.02 ^a^

**Table 3:** The KEGG (Kyoto Encyclopedia of Genes and Genomes) pathway analysis of the epididymal spermatozoa of dogs (*Canis lupus familiaris*) in different age groups (G1: 12–41 months old; G2: 42–77 months old; G3: 78–132 months old). *p* ≤ 0.01. Gene name abbreviations were explained in Tables S1–S3.

**G1**				
**ID**	**Pathway Name**	**Protein Counts**	**−Log^10^** **(Adjusted *p*-Value)**	**Protein Names**
cfa03060	Protein export	3	5.63 × 10^−4^	SRP14|SEC61A1|SRPRA
cfa04141	Protein processing in endoplasmic reticulum	6	6.22 × 10^−4^	TUSC3|SEC24D|MAPK10| RRBP1|SEC61A1|ERN1
cfa04014	Ras signaling pathway	6	3.58 × 10^−3^	RGL2|TIAM1|GRB2| MAPK10|ZAP70|ABL1
cfa04930	Type II diabetes mellitus	3	3.59 × 10^−3^	ABCC8|HK1|MAPK10
cfa00330	Arginine and proline metabolism	3	3.80 × 10^−3^	MAOB|PRODH2|NOS3
cfa04979	Cholesterol metabolism	3	4.02 × 10^−3^	LIPA|ABCG8|ABCA1
cfa02010	ABC transporters	3	4.02 × 10^−3^	ABCG8|ABCC8|ABCA1
cfa04530	Tight junction	5	5.10 × 10^−3^	TIAM1|SLC9A3R1|MAPK10| RAB13|ROCK2
cfa04360	Axon guidance	5	6.14 × 10^−3^	PLXNC1|ABL1|ENAH| ROCK2|ABLIM2
cfa00360	Phenylalanine metabolism	2	6.20 × 10^−3^	MAOB|IL4I1
cfa05135	Yersinia infection	4	7.34 × 10^−3^	ROCK2|MAPK10|ZAP70|NLRP3
**G2**				
**ID**	**Pathway Name**	**Protein Counts**	**−Log^10^** **(Adjusted *p*-Value)**	**Protein Names**
cfa05132	Salmonella infection	3	5.55 × 10^−4^	NFKB1|TJP1|DYNC2H1
cfa05202	Transcriptional misregulation in cancer	3	4.54 × 10^−3^	MET|MLLT1|NFKB1
cfa04520	Adherens junction	2	8.47 × 10^−3^	MET|TJP1
**G3**				
**ID**	**Pathway Name**	**Protein Counts**	**−Log^10^** **(Adjusted *p*-Value)**	**Protein Names**
cfa00512	Mucin-type O-glycan biosynthesis	2	3.37 × 10^−3^	GALNT6|GALNT18
cfa00860	Porphyrin and chlorophyll metabolism	2	3.79 × 10^−3^	BLVRA|CP
cfa04110	Cell cycle	3	4.43 × 10^−3^	SMC1B|ANAPC11|MCM2
cfa04120	Ubiquitin mediated proteolysis	3	6.18 × 10^−3^	ANAPC11|MGRN1|UBR5
cfa02010	ABC transporters	2	7.61 × 10^−3^	ABCA12|ABCC1
cfa05225	Hepatocellular carcinoma	3	9.79 × 10^−3^	PLCG2|TERT|WNT6

## Data Availability

The data that are presented in this study are available on request from the corresponding author.

## Supplementary Materials

The following supporting information can be downloaded at: https://www.mdpi.com/article/10.3390/ijms23169143/s1.
