# Supplementary material for: Age-Dependent Variations in Functional Quality and Proteomic Characteristics of Canine (Canis lupus familiaris) Epididymal Spermatozoa
Source: Int J Mol Sci. 2022 Aug 15;23(16):9143. doi: 10.3390/ijms23169143 (PMC9409041; doi:10.3390/ijms23169143)
Supplement: Supplementary file 1 [file ijms-23-09143-s001.zip › Supplementary Table S1.pdf]

Table S1 Supplementary. Proteins of Group 1 (12 to 41 months old) dog (Canis lupus familiaris) epididymal spermatozoa evaluated by mass spectrometry (NanoUPLC-Q-TOF/MS).

Description	|Log Prob|	Best |Log Prob|	Best score	Total Intensity	# of spectra	# of unique peptides	# of mod peptides	Coverage %	# AA's in protein	Protein DB number
>tr|F1PR54|F1PR54_CANLF Lactotransferrin OS=Canis lupus familiaris OX=9615 GN=LTF PE=3 SV=1	19.74	4.25	327.40	1267254089.0	84	13	1	18.22	708	40436
>tr|F1PR54|F1PR54_CANLF Lactotransferrin OS=Canis lupus familiaris OX=9615 GN=LTF PE=3 SV=1	18.94	3.80	339.40	1215051051.0	72	11	1	16.67	708	40436
>tr|F1P920|F1P920_CANLF Apoptosis resistant E3 ubiquitin protein ligase 1 OS=Canis lupus familiaris OX=9615 GN=AREL1 PE=4 SV=3	0.10	0.00	71.90	1167711957.3	15	1	0	0.88	793	14252
>tr|A0A5F4BVF3|A0A5F4BVF3_CANLF Lactotransferrin OS=Canis lupus familiaris OX=9615 GN=LTF PE=3 SV=1	6.26	2.91	378.90	949659431.2	67	9	0	15.97	626	32850
>tr|A0A5F4BVF3|A0A5F4BVF3_CANLF Lactotransferrin OS=Canis lupus familiaris OX=9615 GN=LTF PE=3 SV=1	11.06	3.33	380.50	806283865.7	76	14	1	21.09	626	32850
>sp|Q28895|NPC2_CANLF NPC intracellular cholesterol transporter 2 OS=Canis lupus familiaris OX=9615 GN=NPC2 PE=2 SV=1	18.28	5.26	587.30	788208902.3	68	4	0	36.91	149	153
>tr|F1P920|F1P920_CANLF Apoptosis resistant E3 ubiquitin protein ligase 1 OS=Canis lupus familiaris OX=9615 GN=AREL1 PE=4 SV=3	0.23	0.03	59.60	785239591.8	11	1	0	0.88	793	14252
>tr|F1PMI1|F1PMI1_CANLF Sidekick cell adhesion molecule 2 OS=Canis lupus familiaris OX=9615 GN=SDK2 PE=4 SV=3	0.18	0.02	21.40	670721164.1	9	1	0	0.28	2120	44885
>tr|A0A5F4DD58|A0A5F4DD58_CANLF Phosphoinositide phospholipase C OS=Canis lupus familiaris OX=9615 GN=PLCD3 PE=4 SV=1	0.24	0.02	146.60	656412058.5	83	4	1	1.48	741	3088
>tr|F2Z4Q6|F2Z4Q6_CANLF Alpha fetoprotein OS=Canis lupus familiaris OX=9615 GN=AFP PE=4 SV=2	20.91	4.01	308.90	651306681.1	130	17	2	33.28	637	24990
>tr|A0A5F4BVF3|A0A5F4BVF3_CANLF Lactotransferrin OS=Canis lupus familiaris OX=9615 GN=LTF PE=3 SV=1	12.50	3.24	332.00	628759365.5	76	10	0	11.50	626	32850
>sp|P49822|ALBU_CANLF Albumin OS=Canis lupus familiaris OX=9615 GN=ALB PE=1 SV=3	10.77	4.02	295.20	624251171.4	36	7	0	14.80	608	490
>tr|F1PR54|F1PR54_CANLF Lactotransferrin OS=Canis lupus familiaris OX=9615 GN=LTF PE=3 SV=1	21.93	4.03	351.30	622958392.6	69	11	0	14.41	708	40436
>tr|J9P6V7|J9P6V7_CANLF HTH CENPB-type domain-containing protein OS=Canis lupus familiaris OX=9615 GN=LOC102153482 PE=3 SV=2	0.67	0.53	28.10	620016126.3	8	1	0	0.85	702	13682
>tr|E2RCT1|E2RCT1_CANLF WAP domain-containing protein OS=Canis lupus familiaris OX=9615 PE=4 SV=2	6.73	3.20	315.30	602951558.5	30	2	0	9.48	116	21717
>tr|E2RCT1|E2RCT1_CANLF WAP domain-containing protein OS=Canis lupus familiaris OX=9615 PE=4 SV=2	5.40	2.72	289.30	557446362.7	36	2	0	9.48	116	21717
>sp|P49822|ALBU_CANLF Albumin OS=Canis lupus familiaris OX=9615 GN=ALB PE=1 SV=3	10.39	3.28	337.00	543988868.7	25	7	0	12.99	608	490
>tr|E2QRT5|E2QRT5_CANLF Structural maintenance of chromosomes protein OS=Canis lupus familiaris OX=9615 GN=SMC1B PE=3 SV=1	0.86	0.44	64.40	496319666.9	22	1	0	0.49	1235	2380
>sp|Q9XS65|PTGDS_CANLF Prostaglandin-H2 D-isomerase OS=Canis lupus familiaris OX=9615 GN=PTGDS PE=2 SV=1	7.98	2.36	377.40	474370331.8	47	5	1	14.14	191	165
>sp|E2RKA8|RL32_CANLF 60S ribosomal protein L32 OS=Canis lupus familiaris OX=9615 GN=RPL32 PE=1 SV=1	1.24	0.02	111.40	460727872.2	89	2	0	4.44	135	275

>tr|F1PR54|F1PR54_CANLF Lactotransferrin OS=Canis lupus familiaris OX=9615 GN=LTF PE=3 SV=1	17.10	3.60	337.30	435790428.3	70	11	0	14.41	708	40436
>tr|Q9XSV4|Q9XSV4_CANLF CE10 protein OS=Canis lupus familiaris OX=9615 GN=ce10 PE=2 SV=1	5.15	3.99	327.80	408366088.7	35	3	0	12.73	110	41542
>sp|P62286|ASPM_CANLF Abnormal spindle-like microcephaly-associated protein homolog OS=Canis lupus familiaris OX=9615 GN=ASPM PE=2 SV=2	0.60	0.49	150.80	394855697.9	87	3	0	0.23	3469	677
>tr|Q9XSV4|Q9XSV4_CANLF CE10 protein OS=Canis lupus familiaris OX=9615 GN=ce10 PE=2 SV=1	3.23	2.66	238.70	383807575.7	28	3	0	12.73	110	41542
>tr|A0A5F4CUD4|A0A5F4CUD4_CANLF Transcription initiation factor TFIID subunit OS=Canis lupus familiaris OX=9615 GN=TAF1 PE=3 SV=1	0.61	0.12	101.20	379105437.2	33	2	0	0.53	1897	4265
>sp|Q28895|NPC2_CANLF NPC intracellular cholesterol transporter 2 OS=Canis lupus familiaris OX=9615 GN=NPC2 PE=2 SV=1	4.98	4.24	362.30	373918670.2	28	2	0	12.75	149	153
>sp|Q9XS65|PTGDS_CANLF Prostaglandin-H2 D-isomerase OS=Canis lupus familiaris OX=9615 GN=PTGDS PE=2 SV=1	3.45	1.81	308.70	368463965.4	17	2	1	10.47	191	165
>sp|E2RKA8|RL32_CANLF 60S ribosomal protein L32 OS=Canis lupus familiaris OX=9615 GN=RPL32 PE=1 SV=1	1.38	0.04	94.00	358661369.7	68	2	0	4.44	135	275
>sp|E2RKA8|RL32_CANLF 60S ribosomal protein L32 OS=Canis lupus familiaris OX=9615 GN=RPL32 PE=1 SV=1	1.90	0.15	167.30	351563558.8	87	2	0	4.44	135	275
>tr|A0A5F4D6L9|A0A5F4D6L9_CANLF Sacsin molecular chaperone OS=Canis lupus familiaris OX=9615 GN=SACS PE=4 SV=1	0.10	0.01	75.30	331955034.6	43	1	0	0.09	4500	1444
>tr|A0A5F4C1S8|A0A5F4C1S8_CANLF E3 ubiquitin-protein ligase CBL OS=Canis lupus familiaris OX=9615 GN=CBL PE=4 SV=1	1.50	0.48	198.40	330371188.7	50	2	0	0.91	773	1308
>tr|F1PR54|F1PR54_CANLF Lactotransferrin OS=Canis lupus familiaris OX=9615 GN=LTF PE=3 SV=1	9.42	3.49	352.00	328762076.3	51	9	1	11.02	708	40436
>tr|F6Y6X6|F6Y6X6_CANLF IQ motif containing GTPase activating protein 3 OS=Canis lupus familiaris OX=9615 GN=IQGAP3 PE=4 SV=2	0.18	0.06	91.10	326817609.0	19	3	1	0.95	1693	7673
>tr|F1PJ71|F1PJ71_CANLF Glutathione peroxidase OS=Canis lupus familiaris OX=9615 GN=GPX5 PE=3 SV=2	6.50	2.91	302.20	318253471.2	21	6	1	29.86	221	19009
>tr|Q9XSV4|Q9XSV4_CANLF CE10 protein OS=Canis lupus familiaris OX=9615 GN=ce10 PE=2 SV=1	6.60	3.56	400.20	310914392.6	43	2	0	9.09	110	41542
>tr|Q9XSV4|Q9XSV4_CANLF CE10 protein OS=Canis lupus familiaris OX=9615 GN=ce10 PE=2 SV=1	6.23	3.27	380.10	309017826.8	38	2	0	9.09	110	41542
>tr|A0A5F4D6L9|A0A5F4D6L9_CANLF Sacsin molecular chaperone OS=Canis lupus familiaris OX=9615 GN=SACS PE=4 SV=1	1.31	0.38	241.30	305031485.1	27	4	0	0.53	4500	1444
>tr|A0A5F4CHL0|A0A5F4CHL0_CANLF Anoctamin OS=Canis lupus familiaris OX=9615 GN=ANO9 PE=3 SV=1	0.65	0.39	176.50	302257152.0	14	1	0	1.82	824	37323
>sp|Q9XS65|PTGDS_CANLF Prostaglandin-H2 D-isomerase OS=Canis lupus familiaris OX=9615 GN=PTGDS PE=2 SV=1	3.47	1.98	327.80	295884114.0	14	2	1	10.47	191	165
>sp|E2RKA8|RL32_CANLF 60S ribosomal protein L32 OS=Canis lupus familiaris OX=9615 GN=RPL32 PE=1 SV=1	0.10	0.00	57.60	284373062.5	39	1	0	2.22	135	275
>tr|F1PJ71|F1PJ71_CANLF Glutathione peroxidase OS=Canis lupus familiaris OX=9615 GN=GPX5 PE=3 SV=2	6.21	3.02	260.80	279752926.7	21	5	1	24.89	221	19009
>tr|A0A5F4BVF3|A0A5F4BVF3_CANLF Lactotransferrin OS=Canis lupus familiaris OX=9615 GN=LTF PE=3 SV=1	3.85	1.27	312.30	273240796.5	28	5	0	8.15	626	32850

>tr|F1PR54|F1PR54_CANLF Lactotransferrin OS=Canis lupus familiaris OX=9615 GN=LTF PE=3 SV=1	21.66	3.33	323.80	266657122.3	49	12	1	16.38	708	40436
>tr|F1PGF6|F1PGF6_CANLF Sprouty related EVH1 domain containing 1 OS=Canis lupus familiaris OX=9615 GN=SPRED1 PE=4 SV=2	0.24	0.02	107.10	258992144.8	20	1	0	0.90	443	10620
>tr|A0A5F4D6L9|A0A5F4D6L9_CANLF Sacsin molecular chaperone OS=Canis lupus familiaris OX=9615 GN=SACS PE=4 SV=1	1.31	0.36	238.10	258707327.3	51	4	0	0.53	4500	1444
>tr|J9PAQ2|J9PAQ2_CANLF Cyclin N-terminal domain-containing protein OS=Canis lupus familiaris OX=9615 PE=3 SV=1	0.62	0.38	54.10	255892985.7	13	1	0	1.25	400	2385
>tr|A0A5F4CR89|A0A5F4CR89_CANLF Voltage-dependent R-type calcium channel subunit alpha OS=Canis lupus familiaris OX=9615 GN=CACNA1E PE=3 SV=1	0.61	0.38	54.10	255892985.7	13	1	0	0.19	2688	1145
>tr|F1P6X5|F1P6X5_CANLF ST6 N-acetylgalactosaminide alpha-2,6-sialyltransferase 5 OS=Canis lupus familiaris OX=9615 GN=ST6GALNAC5 PE=3 SV=3	0.22	0.02	84.50	246113203.8	19	1	0	1.18	338	4902
>sp|O18840|ACTB_CANLF Actin, cytoplasmic 1 OS=Canis lupus familiaris OX=9615 GN=ACTB PE=2 SV=3	1.78	1.56	240.90	226071178.5	12	2	0	7.73	375	642
>tr|Q9XSV4|Q9XSV4_CANLF CE10 protein OS=Canis lupus familiaris OX=9615 GN=ce10 PE=2 SV=1	5.37	3.36	320.20	223705143.2	44	3	0	12.73	110	41542
>tr|A0A5F4BVF3|A0A5F4BVF3_CANLF Lactotransferrin OS=Canis lupus familiaris OX=9615 GN=LTF PE=3 SV=1	3.73	1.41	237.30	221525134.1	30	3	0	3.99	626	32850
>tr|Q9XSV4|Q9XSV4_CANLF CE10 protein OS=Canis lupus familiaris OX=9615 GN=ce10 PE=2 SV=1	1.65	0.82	333.90	215728728.2	23	3	0	14.55	110	41542
>sp|O18840|ACTB_CANLF Actin, cytoplasmic 1 OS=Canis lupus familiaris OX=9615 GN=ACTB PE=2 SV=3	6.01	3.93	316.40	215049909.7	16	3	0	10.13	375	642
>tr|F1PJH2|F1PJH2_CANLF FAT atypical cadherin 3 OS=Canis lupus familiaris OX=9615 GN=FAT3 PE=4 SV=3	0.10	0.00	50.40	214399990.1	30	2	2	0.11	4557	3984
>sp|O18840|ACTB_CANLF Actin, cytoplasmic 1 OS=Canis lupus familiaris OX=9615 GN=ACTB PE=2 SV=3	4.29	3.13	343.40	208868607.2	18	2	0	5.33	375	642
>tr|Q9XSV4|Q9XSV4_CANLF CE10 protein OS=Canis lupus familiaris OX=9615 GN=ce10 PE=2 SV=1	5.67	3.77	288.00	205217653.1	30	3	0	12.73	110	41542
>tr|J9PAQ2|J9PAQ2_CANLF Cyclin N-terminal domain-containing protein OS=Canis lupus familiaris OX=9615 PE=3 SV=1	0.10	0.01	58.20	195241904.1	11	1	0	1.25	400	2385
>tr|A0A5F4CR89|A0A5F4CR89_CANLF Voltage-dependent R-type calcium channel subunit alpha OS=Canis lupus familiaris OX=9615 GN=CACNA1E PE=3 SV=1	0.10	0.01	58.20	195241904.1	11	1	0	0.19	2688	1145
>tr|A0A5F4BVF3|A0A5F4BVF3_CANLF Lactotransferrin OS=Canis lupus familiaris OX=9615 GN=LTF PE=3 SV=1	8.76	3.38	331.90	192363156.0	39	6	0	9.42	626	32850
>sp|Q28895|NPC2_CANLF NPC intracellular cholesterol transporter 2 OS=Canis lupus familiaris OX=9615 GN=NPC2 PE=2 SV=1	10.26	4.49	381.70	191672362.0	21	4	0	30.20	149	153
>sp|Q28895|NPC2_CANLF NPC intracellular cholesterol transporter 2 OS=Canis lupus familiaris OX=9615 GN=NPC2 PE=2 SV=1	8.08	3.25	414.00	187319398.5	27	4	0	30.20	149	153
>tr|A0A5F4D9S5|A0A5F4D9S5_CANLF Hyaluronoglucosaminidase OS=Canis lupus familiaris OX=9615 GN=CEMIP PE=3 SV=1	0.81	0.57	198.20	187265362.7	13	1	0	0.24	1684	9775
>tr|J9P0B4|J9P0B4_CANLF Tudor domain containing 15 OS=Canis lupus familiaris OX=9615 GN=TDRD15 PE=4 SV=2	1.59	1.43	231.60	186749628.5	9	1	0	0.57	2105	4188
>tr|A0A5F4CCF4|A0A5F4CCF4_CANLF Activated leukocyte cell adhesion molecule OS=Canis lupus familiaris OX=9615 GN=ALCAM PE=4 SV=1	0.10	0.00	19.40	183809298.5	17	1	1	0.74	542	12753

>tr|A0A5F4DC90|A0A5F4DC90_CANLF Marker of proliferation Ki-67 OS=Canis lupus familiaris OX=9615 GN=MKI67 PE=4 SV=1	0.10	0.00	19.40	183809298.5	17	1	1	0.16	2529	1622
>sp|Q28895|NPC2_CANLF NPC intracellular cholesterol transporter 2 OS=Canis lupus familiaris OX=9615 GN=NPC2 PE=2 SV=1	11.28	5.59	422.90	182362786.5	25	4	0	30.20	149	153
>sp|O18840|ACTB_CANLF Actin, cytoplasmic 1 OS=Canis lupus familiaris OX=9615 GN=ACTB PE=2 SV=3	2.31	1.21	193.70	178525809.1	10	2	0	7.73	375	642
>tr|A0A5F4C5Q4|A0A5F4C5Q4_CANLF Protein MCM10 homolog OS=Canis lupus familiaris OX=9615 GN=MCM10 PE=3 SV=1	0.10	0.00	14.70	176286212.2	15	1	0	0.47	856	2094
>tr|A0A5F4BU36|A0A5F4BU36_CANLF Titin OS=Canis lupus familiaris OX=9615 GN=TTN PE=3 SV=1	0.23	0.03	38.30	175131771.3	15	2	0	0.04	27097	33785
>tr|A0A5F4D9S5|A0A5F4D9S5_CANLF Hyaluronoglucosaminidase OS=Canis lupus familiaris OX=9615 GN=CEMIP PE=3 SV=1	0.27	0.04	187.80	169746454.8	13	1	0	0.24	1684	9775
>sp|Q2PQH8|GDE_CANLF Glycogen debranching enzyme OS=Canis lupus familiaris OX=9615 GN=AGL PE=2 SV=1	0.70	0.34	78.10	168347768.2	13	2	0	0.26	1533	23
>tr|F1PBU5|F1PBU5_CANLF Non-specific serine/threonine protein kinase OS=Canis lupus familiaris OX=9615 GN=SMG1 PE=3 SV=3	0.95	0.39	163.60	167474779.0	11	2	0	0.17	3634	6898
>tr|A0A5K1V0D8|A0A5K1V0D8_CANLF Sulfatase 2 OS=Canis lupus familiaris OX=9615 GN=SULF2 PE=3 SV=1	0.42	0.17	181.60	157954121.7	15	2	0	0.81	859	1192
>tr|A0A5F4DHJ7|A0A5F4DHJ7_CANLF APC-binding protein EB1 OS=Canis lupus familiaris OX=9615 PE=3 SV=1	0.10	0.00	33.60	156720911.0	19	2	2	1.67	300	38718
>tr|A0A5F4BVF3|A0A5F4BVF3_CANLF Lactotransferrin OS=Canis lupus familiaris OX=9615 GN=LTF PE=3 SV=1	3.63	2.38	321.00	153715851.7	31	4	0	5.91	626	32850
>sp|Q9XS65|PTGDS_CANLF Prostaglandin-H2 D-isomerase OS=Canis lupus familiaris OX=9615 GN=PTGDS PE=2 SV=1	2.19	1.42	297.60	149308819.0	12	2	0	10.47	191	165
>sp|O18840|ACTB_CANLF Actin, cytoplasmic 1 OS=Canis lupus familiaris OX=9615 GN=ACTB PE=2 SV=3	7.57	4.92	325.70	148633565.9	22	2	0	5.33	375	642
>tr|F1PR54|F1PR54_CANLF Lactotransferrin OS=Canis lupus familiaris OX=9615 GN=LTF PE=3 SV=1	2.12	0.75	244.50	148619287.0	21	4	0	5.23	708	40436
>tr|F1PJY1|F1PJY1_CANLF Mannosyl-glycoprotein endo-beta-N-acetylglucosaminidase OS=Canis lupus familiaris OX=9615 GN=ENGASE PE=3 SV=3	1.06	0.39	154.00	146672909.4	17	3	2	1.74	690	32761
>sp|O18840|ACTB_CANLF Actin, cytoplasmic 1 OS=Canis lupus familiaris OX=9615 GN=ACTB PE=2 SV=3	3.78	1.84	297.70	143880969.5	18	3	0	9.87	375	642
>tr|J9P6V7|J9P6V7_CANLF HTH CENPB-type domain-containing protein OS=Canis lupus familiaris OX=9615 GN=LOC102153482 PE=3 SV=2	0.10	0.06	20.10	143181095.0	2	1	0	0.85	702	13682
>sp|O18840|ACTB_CANLF Actin, cytoplasmic 1 OS=Canis lupus familiaris OX=9615 GN=ACTB PE=2 SV=3	5.00	2.91	354.90	142626884.3	14	3	0	10.13	375	642
>sp|P49822|ALBU_CANLF Albumin OS=Canis lupus familiaris OX=9615 GN=ALB PE=1 SV=3	1.54	1.18	254.90	141176435.5	17	5	2	11.02	608	490
>tr|Q30KS5|Q30KS5_CANLF Beta-defensin 129 OS=Canis lupus familiaris OX=9615 GN=DEFB129 PE=2 SV=1	2.65	2.49	340.70	137028954.2	9	1	0	4.22	166	41730
>sp|Q28895|NPC2_CANLF NPC intracellular cholesterol transporter 2 OS=Canis lupus familiaris OX=9615 GN=NPC2 PE=2 SV=1	5.75	4.15	358.30	136510608.5	18	2	0	21.48	149	153
>tr|E2QRT5|E2QRT5_CANLF Structural maintenance of chromosomes protein OS=Canis lupus familiaris OX=9615 GN=SMC1B PE=3 SV=1	0.23	0.11	53.50	135883701.4	7	1	0	0.49	1235	2380

>tr|F1PCW0|F1PCW0_CANLF Golgin A4 OS=Canis lupus familiaris OX=9615 GN=GOLGA4 PE=4 SV=3	0.18	0.02	35.60	134233498.2	13	2	0	0.45	2239	2331
>tr|J9P9B1|J9P9B1_CANLF Mitochondrial translational initiation factor 2 OS=Canis lupus familiaris OX=9615 GN=MTIF2 PE=4 SV=2	0.20	0.02	43.10	132212898.4	12	1	0	0.75	668	1755
>tr|E2RRF5|E2RRF5_CANLF RNA binding motif protein 19 OS=Canis lupus familiaris OX=9615 GN=RBM19 PE=4 SV=3	0.10	0.01	101.40	131832452.8	44	2	0	0.72	970	905
>sp|Q28895|NPC2_CANLF NPC intracellular cholesterol transporter 2 OS=Canis lupus familiaris OX=9615 GN=NPC2 PE=2 SV=1	10.34	3.97	448.10	131596555.2	39	5	0	30.20	149	153
>tr|F1PR54|F1PR54_CANLF Lactotransferrin OS=Canis lupus familiaris OX=9615 GN=LTF PE=3 SV=1	5.24	1.63	235.10	127971923.9	27	8	0	12.71	708	40436
>sp|Q9XS65|PTGDS_CANLF Prostaglandin-H2 D-isomerase OS=Canis lupus familiaris OX=9615 GN=PTGDS PE=2 SV=1	4.20	2.78	330.70	123756320.5	18	3	1	13.61	191	165
>tr|Q9XSV4|Q9XSV4_CANLF CE10 protein OS=Canis lupus familiaris OX=9615 GN=ce10 PE=2 SV=1	3.99	2.29	391.70	122646525.0	23	2	0	9.09	110	41542
>tr|F1PJ71|F1PJ71_CANLF Glutathione peroxidase OS=Canis lupus familiaris OX=9615 GN=GPX5 PE=3 SV=2	1.47	0.58	271.10	122645604.4	14	4	1	15.84	221	19009
>sp|O46607|GPX5_CANLF Epididymal secretory glutathione peroxidase OS=Canis lupus familiaris OX=9615 GN=GPX5 PE=2 SV=1	2.25	1.76	287.00	122228480.8	8	2	0	4.52	221	564
>sp|O46669|SCNAA_CANLF Sodium channel protein type 10 subunit alpha OS=Canis lupus familiaris OX=9615 GN=SCN10A PE=2 SV=1	1.83	0.75	139.20	121295373.1	22	6	0	1.22	1962	90
>tr|A0A5F4CQE4|A0A5F4CQE4_CANLF LARGE xylosyl- and glucuronyltransferase 1 OS=Canis lupus familiaris OX=9615 GN=LARGE1 PE=4 SV=1	0.10	0.00	91.90	119276057.0	15	1	0	0.70	714	7538
>sp|P49822|ALBU_CANLF Albumin OS=Canis lupus familiaris OX=9615 GN=ALB PE=1 SV=3	3.89	1.45	241.20	118117499.8	16	5	2	8.55	608	490
>sp|Q9XS65|PTGDS_CANLF Prostaglandin-H2 D-isomerase OS=Canis lupus familiaris OX=9615 GN=PTGDS PE=2 SV=1	4.00	1.74	334.90	117715935.6	19	4	1	13.61	191	165
>sp|Q9XSU7|RL27_CANLF 60S ribosomal protein L27 OS=Canis lupus familiaris OX=9615 GN=RPL27 PE=2 SV=3	0.62	0.02	60.20	117000186.3	78	1	0	3.68	136	314
>tr|A0A5F4CQE4|A0A5F4CQE4_CANLF LARGE xylosyl- and glucuronyltransferase 1 OS=Canis lupus familiaris OX=9615 GN=LARGE1 PE=4 SV=1	0.45	0.27	41.00	114868641.5	12	1	0	0.70	714	7538
>tr|J9NS29|J9NS29_CANLF Cystatin domain-containing protein OS=Canis lupus familiaris OX=9615 GN=LOC607874 PE=4 SV=2	4.61	2.46	289.60	114468559.1	10	3	0	16.29	313	30016
>tr|A0A5F4CAK8|A0A5F4CAK8_CANLF Ankyrin repeat and sterile alpha motif domain containing 1B OS=Canis lupus familiaris OX=9615 GN=ANKS1B PE=4 SV=1	0.10	0.00	49.50	114257500.4	13	2	2	0.86	1281	31927
>tr|F1PPN1|F1PPN1_CANLF Scaffold attachment factor B2 OS=Canis lupus familiaris OX=9615 GN=SAFB2 PE=4 SV=3	0.10	0.00	69.70	113741557.9	6	1	0	0.63	954	22763
>sp|O18840|ACTB_CANLF Actin, cytoplasmic 1 OS=Canis lupus familiaris OX=9615 GN=ACTB PE=2 SV=3	5.84	4.16	281.40	112762253.4	20	2	0	5.33	375	642
>tr|F1P884|F1P884_CANLF PiggyBac transposable element derived 5 OS=Canis lupus familiaris OX=9615 GN=PGBD5 PE=4 SV=3	0.24	0.12	141.60	107595841.1	7	1	0	2.40	458	28672
>tr|F1PJ71|F1PJ71_CANLF Glutathione peroxidase OS=Canis lupus familiaris OX=9615 GN=GPX5 PE=3 SV=2	2.53	2.00	304.60	106948021.5	16	4	0	25.34	221	19009
>sp|Q28895|NPC2_CANLF NPC intracellular cholesterol transporter 2 OS=Canis lupus familiaris OX=9615 GN=NPC2 PE=2 SV=1	5.37	1.81	343.30	104984185.1	14	4	0	30.20	149	153

>tr|A0A5F4BZW4|A0A5F4BZW4_CANLF Malonyl-CoA decarboxylase OS=Canis lupus familiaris OX=9615 GN=MLYCD PE=4 SV=1	0.48	0.34	187.00	103394674.3	8	1	0	1.30	461	4809
>sp|O18840|ACTB_CANLF Actin, cytoplasmic 1 OS=Canis lupus familiaris OX=9615 GN=ACTB PE=2 SV=3	1.94	1.72	238.10	103375480.6	12	1	0	2.93	375	642
>sp|Q9MZY0|CP2E1_CANLF Cytochrome P450 2E1 OS=Canis lupus familiaris OX=9615 GN=CYP2E1 PE=2 SV=1	0.10	0.03	165.90	103304842.3	6	3	2	4.66	494	522
>tr|A0A5F4C7Q7|A0A5F4C7Q7_CANLF IQ motif and Sec7 domain ArfGEF 1 OS=Canis lupus familiaris OX=9615 GN=IQSEC1 PE=3 SV=1	0.17	0.02	83.80	101797261.8	10	1	0	0.51	971	4269
>tr|J9NS29|J9NS29_CANLF Cystatin domain-containing protein OS=Canis lupus familiaris OX=9615 GN=LOC607874 PE=4 SV=2	1.32	1.27	245.20	101142675.0	7	1	0	6.39	313	30016
>tr|E2R186|E2R186_CANLF Fibroblast growth factor receptor OS=Canis lupus familiaris OX=9615 GN=FGFR1 PE=3 SV=3	0.12	0.02	62.60	100670644.0	13	1	0	0.59	853	9797
>sp|O18840|ACTB_CANLF Actin, cytoplasmic 1 OS=Canis lupus familiaris OX=9615 GN=ACTB PE=2 SV=3	6.15	4.10	411.00	99965065.4	11	2	0	5.33	375	642
>tr|E2RA54|E2RA54_CANLF Bromodomain and WD repeat domain containing 3 OS=Canis lupus familiaris OX=9615 GN=BRWD3 PE=4 SV=3	0.67	0.56	187.20	97944614.4	11	2	0	0.80	1750	4294
>sp|Q28894|WFDC2_CANLF WAP four-disulfide core domain protein 2 OS=Canis lupus familiaris OX=9615 GN=WFDC2 PE=2 SV=1	3.06	2.68	393.10	96221301.8	20	1	0	6.45	124	53
>tr|F1PI09|F1PI09_CANLF Aldehyde oxidase OS=Canis lupus familiaris OX=9615 GN=AOX2 PE=3 SV=3	2.39	2.29	228.50	96041029.3	6	1	0	0.67	1347	21650
>sp|F1PTE3|RAB13_CANLF Ras-related protein Rab-13 OS=Canis lupus familiaris OX=9615 GN=RAB13 PE=1 SV=2	0.50	0.23	142.90	95584726.0	6	2	0	2.46	203	147
>tr|Q30KS5|Q30KS5_CANLF Beta-defensin 129 OS=Canis lupus familiaris OX=9615 GN=DEFB129 PE=2 SV=1	1.47	1.35	326.80	93102732.0	7	1	0	4.22	166	41730
>tr|A0A5F4D6L9|A0A5F4D6L9_CANLF Sacsin molecular chaperone OS=Canis lupus familiaris OX=9615 GN=SACS PE=4 SV=1	1.02	0.85	229.70	92992927.0	17	4	1	0.33	4500	1444
>sp|Q2PQH8|GDE_CANLF Glycogen debranching enzyme OS=Canis lupus familiaris OX=9615 GN=AGL PE=2 SV=1	0.42	0.09	104.80	92985336.0	15	2	0	0.26	1533	23
>tr|F1PPN1|F1PPN1_CANLF Scaffold attachment factor B2 OS=Canis lupus familiaris OX=9615 GN=SAFB2 PE=4 SV=3	0.15	0.07	69.40	92373389.0	5	1	0	0.63	954	22763
>tr|Q9XSV4|Q9XSV4_CANLF CE10 protein OS=Canis lupus familiaris OX=9615 GN=ce10 PE=2 SV=1	4.13	2.46	333.50	92001608.0	28	2	0	9.09	110	41542
>sp|Q2PQH8|GDE_CANLF Glycogen debranching enzyme OS=Canis lupus familiaris OX=9615 GN=AGL PE=2 SV=1	0.45	0.18	130.60	90954014.0	14	2	0	0.26	1533	23
>sp|Q9MZY0|CP2E1_CANLF Cytochrome P450 2E1 OS=Canis lupus familiaris OX=9615 GN=CYP2E1 PE=2 SV=1	0.10	0.02	92.20	90796602.3	9	1	1	4.05	494	522
>sp|O18840|ACTB_CANLF Actin, cytoplasmic 1 OS=Canis lupus familiaris OX=9615 GN=ACTB PE=2 SV=3	2.55	1.19	236.40	90298869.2	14	3	0	9.87	375	642
>sp|Q28895|NPC2_CANLF NPC intracellular cholesterol transporter 2 OS=Canis lupus familiaris OX=9615 GN=NPC2 PE=2 SV=1	5.01	3.36	264.20	90121962.8	29	3	0	21.48	149	153
>sp|Q9XS65|PTGDS_CANLF Prostaglandin-H2 D-isomerase OS=Canis lupus familiaris OX=9615 GN=PTGDS PE=2 SV=1	2.23	1.28	283.50	89686197.0	13	3	1	10.47	191	165
>tr|J9P5T2|J9P5T2_CANLF Non-specific serine/threonine protein kinase OS=Canis lupus familiaris OX=9615 GN=WNK3 PE=4 SV=2	0.10	0.00	45.10	89526200.9	11	1	0	0.22	2294	5229

>tr|F1PGF9|F1PGF9_CANLF Rho guanine nucleotide exchange factor 26 OS=Canis lupus familiaris OX=9615 GN=ARHGEF26 PE=4 SV=3	1.39	1.29	320.30	87856755.1	6	1	0	0.84	594	22876
>tr|A0A5F4C535|A0A5F4C535_CANLF ADAM metallopeptidase with thrombospondin type 1 motif 13 OS=Canis lupus familiaris OX=9615 GN=ADAMTS13 PE=4 SV=1	0.10	0.02	84.50	87599235.6	7	1	0	0.90	444	2664
>sp|Q9XS65|PTGDS_CANLF Prostaglandin-H2 D-isomerase OS=Canis lupus familiaris OX=9615 GN=PTGDS PE=2 SV=1	2.01	1.55	288.50	85454601.9	11	2	1	10.47	191	165
>tr|A0A5F4CDY8|A0A5F4CDY8_CANLF Kinesin family member 16B OS=Canis lupus familiaris OX=9615 GN=KIF16B PE=3 SV=1	0.95	0.02	85.80	85362610.9	61	1	1	0.96	1245	3757
>tr|A0A5F4DK55|A0A5F4DK55_CANLF Reverse transcriptase domain-containing protein OS=Canis lupus familiaris OX=9615 PE=4 SV=1	0.50	0.23	180.50	81982953.1	7	2	0	0.55	1275	1651
>tr|A0A5F4BVF3|A0A5F4BVF3_CANLF Lactotransferrin OS=Canis lupus familiaris OX=9615 GN=LTF PE=3 SV=1	1.05	0.97	275.10	81961926.1	13	3	0	3.99	626	32850
>tr|F1Q0P9|F1Q0P9_CANLF AT-hook containing transcription factor 1 OS=Canis lupus familiaris OX=9615 GN=AHCTF1 PE=4 SV=3	0.23	0.03	93.20	81588408.8	13	2	0	0.26	2321	1521
>tr|F1PI09|F1PI09_CANLF Aldehyde oxidase OS=Canis lupus familiaris OX=9615 GN=AOX2 PE=3 SV=3	2.47	2.39	213.10	81422792.8	5	1	0	0.67	1347	21650
>tr|F1P7F0|F1P7F0_CANLF DENN domain containing 2A OS=Canis lupus familiaris OX=9615 GN=DENND2A PE=4 SV=3	0.10	0.01	34.30	80662709.3	7	1	0	0.45	1104	2294
>tr|E2R5H9|E2R5H9_CANLF Blood vessel epicardial substance OS=Canis lupus familiaris OX=9615 GN=BVES PE=3 SV=1	0.50	0.44	67.50	77690368.2	4	1	0	1.67	360	8594
>tr|F1PGF9|F1PGF9_CANLF Rho guanine nucleotide exchange factor 26 OS=Canis lupus familiaris OX=9615 GN=ARHGEF26 PE=4 SV=3	0.57	0.53	266.90	76427460.5	3	1	0	0.84	594	22876
>sp|O18840|ACTB_CANLF Actin, cytoplasmic 1 OS=Canis lupus familiaris OX=9615 GN=ACTB PE=2 SV=3	2.49	1.58	266.20	76102799.8	11	2	0	5.07	375	642
>tr|A0A5F4CCD0|A0A5F4CCD0_CANLF Cysteine rich secretory protein 2 OS=Canis lupus familiaris OX=9615 GN=CRISP2 PE=3 SV=1	1.32	1.26	213.50	75936743.2	5	2	0	7.07	311	11017
>tr|A0A5F4DHE4|A0A5F4DHE4_CANLF Protein tyrosine phosphatase non-receptor type 22 OS=Canis lupus familiaris OX=9615 GN=PTPN22 PE=4 SV=1	0.54	0.51	173.30	75764927.7	4	2	1	2.42	784	5054
>tr|J9JHT4|J9JHT4_CANLF Unc-79 homolog, NALCN channel complex subunit OS=Canis lupus familiaris OX=9615 GN=UNC79 PE=4 SV=2	0.36	0.27	38.30	74913627.1	6	1	0	0.19	2622	39915
>tr|E2RCT1|E2RCT1_CANLF WAP domain-containing protein OS=Canis lupus familiaris OX=9615 PE=4 SV=2	3.06	1.64	242.50	74730589.2	12	2	0	9.48	116	21717
>tr|J9NTF0|J9NTF0_CANLF PTPRF interacting protein alpha 1 OS=Canis lupus familiaris OX=9615 GN=PPFIA1 PE=3 SV=2	0.10	0.00	6.70	74343383.3	5	1	1	0.47	1288	2190
>tr|F1PEX6|F1PEX6_CANLF Tyrosine-protein kinase OS=Canis lupus familiaris OX=9615 GN=ABL1 PE=3 SV=2	0.17	0.02	219.40	74134182.7	11	1	1	0.70	1150	1051
>sp|P49822|ALBU_CANLF Albumin OS=Canis lupus familiaris OX=9615 GN=ALB PE=1 SV=3	3.43	2.97	251.90	72775789.7	7	3	0	6.91	608	490
>tr|A0A5F4D850|A0A5F4D850_CANLF Structural maintenance of chromosomes protein OS=Canis lupus familiaris OX=9615 GN=SMC3 PE=3 SV=1	0.10	0.02	40.70	72513150.7	3	1	0	0.41	1219	7467
>tr|A0A5F4C7X0|A0A5F4C7X0_CANLF Centriolin OS=Canis lupus familiaris OX=9615 GN=CNTRL PE=4 SV=1	0.10	0.02	40.70	72513150.7	3	1	0	0.20	2506	3658
>tr|J9NZY7|J9NZY7_CANLF Midasin OS=Canis lupus familiaris OX=9615 GN=MDN1 PE=3 SV=2	0.10	0.02	40.70	72513150.7	3	1	0	0.09	5558	2169

>sp|Q28894|WFDC2_CANLF WAP four-disulfide core domain protein 2 OS=Canis lupus familiaris OX=9615 GN=WFDC2 PE=2 SV=1	2.29	2.07	427.50	71379011.9	12	1	0	6.45	124	53
>tr|A0A5F4BVF3|A0A5F4BVF3_CANLF Lactotransferrin OS=Canis lupus familiaris OX=9615 GN=LTF PE=3 SV=1	2.55	2.36	294.00	71122865.2	10	3	0	3.99	626	32850
>tr|A0A5F4CB08|A0A5F4CB08_CANLF Glutathione transferase OS=Canis lupus familiaris OX=9615 GN=LOC100856518 PE=3 SV=1	0.58	0.56	199.40	71054194.6	4	1	0	3.08	292	4491
>sp|Q8WN22|PRKDC_CANLF DNA-dependent protein kinase catalytic subunit OS=Canis lupus familiaris OX=9615 GN=PRKDC PE=2 SV=1	1.07	0.72	83.40	70510292.8	9	3	0	0.19	4144	338
>tr|A0A5F4DGF5|A0A5F4DGF5_CANLF Alkaline phosphatase OS=Canis lupus familiaris OX=9615 GN=ALPL PE=3 SV=1	5.03	3.46	347.90	69987456.6	7	3	0	5.77	572	6357
>tr|A0A5F4CHL0|A0A5F4CHL0_CANLF Anoctamin OS=Canis lupus familiaris OX=9615 GN=ANO9 PE=3 SV=1	0.44	0.38	139.70	68736909.1	4	1	0	1.82	824	37323
>tr|J9NTK2|J9NTK2_CANLF J domain-containing protein OS=Canis lupus familiaris OX=9615 GN=DNAJC12 PE=4 SV=2	0.27	0.03	202.00	68153747.8	13	1	0	4.72	106	2310
>tr|E2RIK1|E2RIK1_CANLF Phosphatidylinositol-4-phosphate 3-kinase OS=Canis lupus familiaris OX=9615 GN=PIK3C2G PE=3 SV=3	0.10	0.01	86.40	67553350.1	3	1	0	0.42	1445	5924
>sp|Q6AW47|EST5A_CANLF Carboxylesterase 5A OS=Canis lupus familiaris OX=9615 GN=CES5A PE=2 SV=1	3.04	1.47	266.70	66636742.9	11	4	0	5.22	575	629
>tr|F1PJ71|F1PJ71_CANLF Glutathione peroxidase OS=Canis lupus familiaris OX=9615 GN=GPX5 PE=3 SV=2	0.86	0.60	231.50	66067554.5	10	4	0	25.34	221	19009
>tr|F1P8L2|F1P8L2_CANLF Thymine DNA glycosylase OS=Canis lupus familiaris OX=9615 GN=TDG PE=4 SV=2	0.10	0.05	18.60	65118195.8	4	1	0	1.97	406	37574
>tr|E2R6E0|E2R6E0_CANLF Lipocln_cytosolic_FA-bd_dom domain-containing protein OS=Canis lupus familiaris OX=9615 GN=LCNL1 PE=3 SV=2	2.28	2.18	228.10	64505297.6	6	1	0	3.01	299	1932
>tr|A0A5F4CNT4|A0A5F4CNT4_CANLF Microtubule actin crosslinking factor 1 OS=Canis lupus familiaris OX=9615 GN=MACF1 PE=4 SV=1	0.85	0.71	153.50	64378332.7	8	1	0	0.11	7352	1251
>tr|F1PDQ3|F1PDQ3_CANLF Beta-nerve growth factor OS=Canis lupus familiaris OX=9615 GN=NGF PE=3 SV=3	0.10	0.07	37.20	63003806.8	9	3	2	2.08	240	6254
>tr|J9P436|J9P436_CANLF G protein-coupled receptor 45 OS=Canis lupus familiaris OX=9615 GN=GPR45 PE=4 SV=2	0.10	0.07	37.20	63003806.8	9	2	1	1.34	373	7720
>sp|O18840|ACTB_CANLF Actin, cytoplasmic 1 OS=Canis lupus familiaris OX=9615 GN=ACTB PE=2 SV=3	1.91	1.73	225.70	62856604.7	10	1	0	2.93	375	642
>tr|A0A5F4CL05|A0A5F4CL05_CANLF Chloride channel protein OS=Canis lupus familiaris OX=9615 GN=CLCN7 PE=3 SV=1	0.35	0.27	38.30	62752256.0	5	1	0	0.59	844	10399
>sp|Q5TJE5|RGL2_CANLF Ral guanine nucleotide dissociation stimulator-like 2 OS=Canis lupus familiaris OX=9615 GN=RGL2 PE=3 SV=1	1.32	0.93	101.00	61436340.9	4	2	0	0.64	780	266
>sp|Q28298|RRBP1_CANLF Ribosome-binding protein 1 OS=Canis lupus familiaris OX=9615 GN=RRBP1 PE=2 SV=1	0.48	0.27	99.90	61403971.8	14	3	0	0.46	1534	61
>sp|Q2PQH8|GDE_CANLF Glycogen debranching enzyme OS=Canis lupus familiaris OX=9615 GN=AGL PE=2 SV=1	0.98	0.39	106.20	60463153.8	13	5	0	0.65	1533	23
>tr|E2RIK1|E2RIK1_CANLF Phosphatidylinositol-4-phosphate 3-kinase OS=Canis lupus familiaris OX=9615 GN=PIK3C2G PE=3 SV=3	0.48	0.44	55.60	59105479.1	3	1	0	0.42	1445	5924
>tr|F1PJY1|F1PJY1_CANLF Mannosyl-glycoprotein endo-beta-N-acetylglucosaminidase OS=Canis lupus familiaris OX=9615 GN=ENGASE PE=3 SV=3	0.25	0.02	183.30	58690567.6	13	2	2	1.74	690	32761

>tr|F1PBU5|F1PBU5_CANLF Non-specific serine/threonine protein kinase OS=Canis lupus familiaris OX=9615 GN=SMG1 PE=3 SV=3	0.80	0.49	201.10	58165901.4	13	2	0	0.17	3634	6898
>tr|E2RQN7|E2RQN7_CANLF RNA helicase OS=Canis lupus familiaris OX=9615 GN=DHX35 PE=4 SV=3	0.10	0.02	37.30	57891199.6	4	1	0	2.95	679	32613
>sp|P62286|ASPM_CANLF Abnormal spindle-like microcephaly-associated protein homolog OS=Canis lupus familiaris OX=9615 GN=ASPM PE=2 SV=2	0.94	0.36	101.20	57685539.8	6	4	0	0.32	3469	677
>tr|A0A5F4CCD0|A0A5F4CCD0_CANLF Cysteine rich secretory protein 2 OS=Canis lupus familiaris OX=9615 GN=CRISP2 PE=3 SV=1	0.83	0.79	174.80	57143240.6	4	2	0	7.07	311	11017
>tr|A0A5K1V0D8|A0A5K1V0D8_CANLF Sulfatase 2 OS=Canis lupus familiaris OX=9615 GN=SULF2 PE=3 SV=1	0.46	0.36	168.00	56781003.6	6	1	0	0.35	859	1192
>tr|F1PCW0|F1PCW0_CANLF Golgin A4 OS=Canis lupus familiaris OX=9615 GN=GOLGA4 PE=4 SV=3	0.10	0.00	14.70	56622341.0	8	1	0	0.22	2239	2331
>tr|A0A5F4D9S5|A0A5F4D9S5_CANLF Hyaluronoglucosaminidase OS=Canis lupus familiaris OX=9615 GN=CEMIP PE=3 SV=1	0.35	0.21	133.40	56062704.2	8	1	0	0.24	1684	9775
>tr|E2R856|E2R856_CANLF Myeloid derived growth factor OS=Canis lupus familiaris OX=9615 GN=MYDGF PE=4 SV=3	0.31	0.27	29.00	56022807.2	3	1	0	3.47	173	26349
>tr|A0A5F4CTQ6|A0A5F4CTQ6_CANLF SLC9A3 regulator 2 OS=Canis lupus familiaris OX=9615 GN=SLC9A3R2 PE=4 SV=1	0.31	0.27	29.00	56022807.2	3	1	0	1.90	315	18010
>tr|E2RKA1|E2RKA1_CANLF Tyrosine-protein kinase receptor OS=Canis lupus familiaris OX=9615 GN=NTRK2 PE=3 SV=2	0.31	0.27	29.00	56022807.2	3	1	0	0.73	822	20603
>tr|F1PEA8|F1PEA8_CANLF Phosphatidylinositol-3,4,5-trisphosphate 5-phosphatase OS=Canis lupus familiaris OX=9615 GN=INPP5D PE=3 SV=3	0.31	0.27	29.00	56022807.2	3	1	0	0.43	1399	17627
>tr|E2QX33|E2QX33_CANLF Coiled-coil and C2 domain containing 1A OS=Canis lupus familiaris OX=9615 GN=CC2D1A PE=3 SV=1	0.10	0.03	148.20	55979391.6	5	1	1	0.84	951	10961
>tr|A0A5F4BSC2|A0A5F4BSC2_CANLF Galectin OS=Canis lupus familiaris OX=9615 GN=LGALS4 PE=4 SV=1	0.10	0.00	74.20	55878627.6	6	1	0	1.62	309	19997
>tr|A0A5F4CM35|A0A5F4CM35_CANLF Glycerophosphodiester phosphodiesterase domain containing 2 OS=Canis lupus familiaris OX=9615 GN=GDPD2 PE=3 SV=1	0.10	0.00	24.40	55805501.5	9	1	1	0.65	614	16708
>sp|O18840|ACTB_CANLF Actin, cytoplasmic 1 OS=Canis lupus familiaris OX=9615 GN=ACTB PE=2 SV=3	1.44	1.30	287.50	55329329.6	10	2	0	5.07	375	642
>sp|Q9MZY0|CP2E1_CANLF Cytochrome P450 2E1 OS=Canis lupus familiaris OX=9615 GN=CYP2E1 PE=2 SV=1	1.57	1.04	129.10	55324154.9	8	2	1	4.66	494	522
>tr|E2RCT1|E2RCT1_CANLF WAP domain-containing protein OS=Canis lupus familiaris OX=9615 PE=4 SV=2	4.69	3.76	349.40	54634292.4	8	2	0	14.66	116	21717
>tr|E2R6E0|E2R6E0_CANLF Lipocln_cytosolic_FA-bd_dom domain-containing protein OS=Canis lupus familiaris OX=9615 GN=LCNL1 PE=3 SV=2	2.40	2.36	135.40	54394337.6	3	1	0	3.01	299	1932
>tr|A0A5F4CLI1|A0A5F4CLI1_CANLF Histone deacetylase 6 OS=Canis lupus familiaris OX=9615 GN=HDAC6 PE=4 SV=1	0.49	0.45	172.70	54364173.4	3	1	0	0.43	1175	4057
>tr|E2RKA1|E2RKA1_CANLF Tyrosine-protein kinase receptor OS=Canis lupus familiaris OX=9615 GN=NTRK2 PE=3 SV=2	0.20	0.18	58.00	54282136.4	2	1	0	0.73	822	20603
>tr|F1PBJ1|F1PBJ1_CANLF Methylcytosine dioxygenase TET OS=Canis lupus familiaris OX=9615 GN=TET3 PE=3 SV=2	0.10	0.00	86.80	53409123.6	5	1	0	0.28	1795	1529
>sp|P25473|CLUS_CANLF Clusterin OS=Canis lupus familiaris OX=9615 GN=CLU PE=2 SV=1	0.38	0.34	218.40	53142113.9	3	1	0	0.90	445	725

>sp|F1PRN2|MYO1D_CANLF Unconventional myosin-Id OS=Canis lupus familiaris OX=9615 GN=MYO1D PE=1 SV=2	1.09	0.57	160.80	52902342.3	9	2	0	0.60	1006	763
>tr|E2RE16|E2RE16_CANLF Non-specific serine/threonine protein kinase OS=Canis lupus familiaris OX=9615 GN=PAK4 PE=4 SV=1	0.34	0.08	95.30	52479900.2	41	1	0	0.84	592	12735
>tr|E2RIK1|E2RIK1_CANLF Phosphatidylinositol-4-phosphate 3-kinase OS=Canis lupus familiaris OX=9615 GN=PIK3C2G PE=3 SV=3	0.46	0.44	67.00	52439169.9	2	1	0	0.42	1445	5924
>tr|E2RCT1|E2RCT1_CANLF WAP domain-containing protein OS=Canis lupus familiaris OX=9615 PE=4 SV=2	5.07	3.13	393.10	50869292.0	8	2	0	14.66	116	21717
>sp|O18840|ACTB_CANLF Actin, cytoplasmic 1 OS=Canis lupus familiaris OX=9615 GN=ACTB PE=2 SV=3	0.61	0.47	273.20	50476927.3	8	2	0	5.07	375	642
>tr|J9P7Y2|J9P7Y2_CANLF Angiotensin-converting enzyme OS=Canis lupus familiaris OX=9615 GN=ACE2 PE=3 SV=1	0.10	0.02	90.80	49895948.3	3	1	0	1.62	804	1041
>tr|E2RN16|E2RN16_CANLF Mitogen-activated protein kinase kinase kinase 2 OS=Canis lupus familiaris OX=9615 GN=MAP3K2 PE=4 SV=2	0.40	0.34	188.30	49798235.8	4	1	0	0.97	620	34325
>tr|A0A5F4BYT5|A0A5F4BYT5_CANLF Tyrosine-protein kinase OS=Canis lupus familiaris OX=9615 GN=ZAP70 PE=3 SV=1	0.43	0.35	99.40	47983643.3	5	1	0	3.72	592	15350
>tr|A0A5F4DIZ5|A0A5F4DIZ5_CANLF Kinesin-like protein OS=Canis lupus familiaris OX=9615 GN=KIF9 PE=3 SV=1	0.14	0.06	174.80	47784525.6	5	1	0	1.50	732	7709
>tr|A0A5F4C730|A0A5F4C730_CANLF Semaphorin 4D OS=Canis lupus familiaris OX=9615 GN=SEMA4D PE=3 SV=1	0.23	0.15	124.20	47685286.2	5	1	0	0.28	1067	1802
>tr|Q9XSV4|Q9XSV4_CANLF CE10 protein OS=Canis lupus familiaris OX=9615 GN=ce10 PE=2 SV=1	1.04	0.40	187.20	47663466.3	15	3	0	12.73	110	41542
>sp|Q6AW47|EST5A_CANLF Carboxylesterase 5A OS=Canis lupus familiaris OX=9615 GN=CES5A PE=2 SV=1	1.74	0.82	255.20	47577384.0	8	3	0	3.65	575	629
>tr|A0A5F4DD58|A0A5F4DD58_CANLF Phosphoinositide phospholipase C OS=Canis lupus familiaris OX=9615 GN=PLCD3 PE=4 SV=1	0.17	0.01	52.90	46589241.4	54	2	0	1.08	741	3088
>tr|A0A5F4C615|A0A5F4C615_CANLF Glucocorticoid receptor OS=Canis lupus familiaris OX=9615 GN=NR3C1 PE=3 SV=1	0.10	0.06	106.90	45155646.5	3	2	1	2.76	725	7049
>tr|F1PLV2|F1PLV2_CANLF Peptidyl-prolyl cis-trans isomerase OS=Canis lupus familiaris OX=9615 GN=CSNK1G1 PE=3 SV=3	0.67	0.65	193.00	45151630.6	3	1	0	5.35	243	4290
>sp|Q9XSU7|RL27_CANLF 60S ribosomal protein L27 OS=Canis lupus familiaris OX=9615 GN=RPL27 PE=2 SV=3	0.54	0.02	42.80	44466788.4	74	1	0	3.68	136	314
>tr|F1P8J6|F1P8J6_CANLF RNA helicase OS=Canis lupus familiaris OX=9615 GN=DDX55 PE=3 SV=3	0.28	0.24	147.60	44211263.8	3	1	0	1.58	568	8934
>tr|E2R186|E2R186_CANLF Fibroblast growth factor receptor OS=Canis lupus familiaris OX=9615 GN=FGFR1 PE=3 SV=3	0.10	0.00	116.70	43892865.4	8	1	0	0.59	853	9797
>sp|P06625|SRPRA_CANLF Signal recognition particle receptor subunit alpha OS=Canis lupus familiaris OX=9615 GN=SRPRA PE=2 SV=2	0.14	0.05	179.10	43747251.5	5	3	0	2.82	638	369
>tr|F1P7Z8|F1P7Z8_CANLF Coiled-coil domain-containing protein 93 OS=Canis lupus familiaris OX=9615 GN=CCDC93 PE=3 SV=2	0.10	0.03	34.80	42083001.7	5	1	0	0.63	631	1813
>tr|E2R6E0|E2R6E0_CANLF Lipocln_cytosolic_FA-bd_dom domain-containing protein OS=Canis lupus familiaris OX=9615 GN=LCNL1 PE=3 SV=2	2.14	2.06	224.10	42056053.9	5	1	0	3.01	299	1932
>tr|A0A5F4BXA7|A0A5F4BXA7_CANLF Leucine rich repeat containing 7 OS=Canis lupus familiaris OX=9615 GN=LRRC7 PE=3 SV=1	0.13	0.05	130.20	41644681.3	5	1	0	0.39	1542	2603

>tr|F1PGK9|F1PGK9_CANLF ADAM metallopeptidase with thrombospondin type 1 motif 5 OS=Canis lupus familiaris OX=9615 GN=ADAMTS5 PE=4 SV=3	0.12	0.02	75.80	41480860.2	27	1	0	0.59	845	11956
>tr|F1PM73|F1PM73_CANLF Palmitoyltransferase OS=Canis lupus familiaris OX=9615 GN=ZDHHC23 PE=3 SV=3	0.24	0.16	55.50	41316872.2	5	1	0	3.98	427	22460
>tr|A0A5F4CM35|A0A5F4CM35_CANLF Glycerophosphodiester phosphodiesterase domain containing 2 OS=Canis lupus familiaris OX=9615 GN=GDPD2 PE=3 SV=1	0.10	0.01	64.70	41155352.5	4	1	1	0.65	614	16708
>tr|F1PP47|F1PP47_CANLF RE1 silencing transcription factor OS=Canis lupus familiaris OX=9615 GN=REST PE=4 SV=2	0.10	0.03	107.10	41062721.9	5	2	1	0.90	996	17823
>tr|F1PBU5|F1PBU5_CANLF Non-specific serine/threonine protein kinase OS=Canis lupus familiaris OX=9615 GN=SMG1 PE=3 SV=3	1.51	0.81	177.20	40793140.9	10	2	0	0.17	3634	6898
>tr|F1PRU0|F1PRU0_CANLF WD_REPEATS_REGION domain-containing protein OS=Canis lupus familiaris OX=9615 GN=TLE7 PE=3 SV=2	0.33	0.05	31.10	40601505.3	15	1	0	1.16	431	22849
>tr|E2RCT1|E2RCT1_CANLF WAP domain-containing protein OS=Canis lupus familiaris OX=9615 PE=4 SV=2	1.47	1.40	135.90	40419871.7	3	2	0	9.48	116	21717
>tr|E2R6E0|E2R6E0_CANLF Lipocln_cytosolic_FA-bd_dom domain-containing protein OS=Canis lupus familiaris OX=9615 GN=LCNL1 PE=3 SV=2	8.13	2.94	152.90	40061195.9	5	3	0	9.36	299	1932
>tr|A0A5F4DGX9|A0A5F4DGX9_CANLF Target of myb1 membrane trafficking protein OS=Canis lupus familiaris OX=9615 GN=TOM1 PE=3 SV=1	0.16	0.08	164.80	39625191.0	5	1	0	3.35	477	40074
>tr|A0A5F4CRP9|A0A5F4CRP9_CANLF Tumor suppressor candidate 3 OS=Canis lupus familiaris OX=9615 GN=TUSC3 PE=3 SV=1	0.42	0.30	84.90	38762611.3	7	1	0	3.18	314	6225
>sp|Q9XS65|PTGDS_CANLF Prostaglandin-H2 D-isomerase OS=Canis lupus familiaris OX=9615 GN=PTGDS PE=2 SV=1	9.62	2.91	344.50	38730905.4	74	4	1	19.37	191	165
>tr|F1P9J6|F1P9J6_CANLF Zinc finger protein 16 OS=Canis lupus familiaris OX=9615 GN=ZNF16 PE=4 SV=3	0.38	0.36	158.40	38657674.5	2	1	0	1.47	680	39751
>tr|A0A5F4DGF5|A0A5F4DGF5_CANLF Alkaline phosphatase OS=Canis lupus familiaris OX=9615 GN=ALPL PE=3 SV=1	0.87	0.77	206.80	37988761.9	3	2	0	5.77	572	6357
>tr|A0A5F4BXD8|A0A5F4BXD8_CANLF Matrix metallopeptidase 16 OS=Canis lupus familiaris OX=9615 GN=MMP16 PE=3 SV=1	0.45	0.43	183.90	37714034.6	2	1	0	2.65	566	6476
>tr|E2R6E0|E2R6E0_CANLF Lipocln_cytosolic_FA-bd_dom domain-containing protein OS=Canis lupus familiaris OX=9615 GN=LCNL1 PE=3 SV=2	1.71	1.55	189.00	37651996.4	9	1	0	3.68	299	1932
>sp|Q6AW47|EST5A_CANLF Carboxylesterase 5A OS=Canis lupus familiaris OX=9615 GN=CES5A PE=2 SV=1	0.59	0.53	164.10	37613594.5	3	2	0	3.83	575	629
>sp|P49822|ALBU_CANLF Albumin OS=Canis lupus familiaris OX=9615 GN=ALB PE=1 SV=3	1.27	0.75	97.60	37265884.2	6	2	0	4.77	608	490
>tr|A0A5K1V0D8|A0A5K1V0D8_CANLF Sulfatase 2 OS=Canis lupus familiaris OX=9615 GN=SULF2 PE=3 SV=1	0.29	0.13	207.90	37025926.2	9	1	0	0.35	859	1192
>tr|A0A5F4BSC2|A0A5F4BSC2_CANLF Galectin OS=Canis lupus familiaris OX=9615 GN=LGALS4 PE=4 SV=1	0.10	0.02	6.00	36912888.7	3	1	0	1.62	309	19997
>sp|Q6AW47|EST5A_CANLF Carboxylesterase 5A OS=Canis lupus familiaris OX=9615 GN=CES5A PE=2 SV=1	0.70	0.36	189.80	36484739.2	2	2	0	1.57	575	629
>sp|Q2PQH8|GDE_CANLF Glycogen debranching enzyme OS=Canis lupus familiaris OX=9615 GN=AGL PE=2 SV=1	0.51	0.50	81.40	36251439.8	2	1	0	0.13	1533	23
>tr|A0A5F4CQE4|A0A5F4CQE4_CANLF LARGE xylosyl- and glucuronyltransferase 1 OS=Canis lupus familiaris OX=9615 GN=LARGE1 PE=4 SV=1	0.10	0.00	17.90	36057021.7	5	1	0	0.70	714	7538

>tr|A0A5F4CYM0|A0A5F4CYM0_CANLF Quiescin sulfhydryl oxidase 2 OS=Canis lupus familiaris OX=9615 GN=QSOX2 PE=4 SV=1	0.10	0.02	76.90	35835400.1	10	1	0	2.52	636	1464
>tr|F1PQZ1|F1PQZ1_CANLF Hyaluronan binding protein 4 OS=Canis lupus familiaris OX=9615 GN=HABP4 PE=4 SV=2	0.15	0.09	161.80	35234505.3	4	1	0	7.91	215	39227
>tr|J9NTK2|J9NTK2_CANLF J domain-containing protein OS=Canis lupus familiaris OX=9615 GN=DNAJC12 PE=4 SV=2	0.10	0.03	121.30	34933309.7	2	1	0	4.72	106	2310
>tr|A0A5F4BZW4|A0A5F4BZW4_CANLF Malonyl-CoA decarboxylase OS=Canis lupus familiaris OX=9615 GN=MLYCD PE=4 SV=1	0.25	0.17	204.20	34887633.1	5	1	0	1.30	461	4809
>tr|A0A5F4D0B3|A0A5F4D0B3_CANLF Bromodomain containing 1 OS=Canis lupus familiaris OX=9615 GN=BRD1 PE=4 SV=1	0.19	0.17	154.30	34688350.5	2	1	0	0.81	1112	1580
>tr|J9NS28|J9NS28_CANLF RBR-type E3 ubiquitin transferase OS=Canis lupus familiaris OX=9615 GN=ANKIB1 PE=4 SV=2	0.23	0.19	37.00	34454786.8	3	1	0	2.33	988	26345
>tr|J9P3V5|J9P3V5_CANLF FAM75 domain-containing protein OS=Canis lupus familiaris OX=9615 PE=4 SV=2	0.10	0.08	173.80	34311408.5	2	1	0	0.36	1370	32063
>tr|A0A5F4CT98|A0A5F4CT98_CANLF [Heparan sulfate]-glucosamine N-sulfotransferase OS=Canis lupus familiaris OX=9615 GN=NDST1 PE=3 SV=1	0.14	0.14	177.60	34027788.6	5	2	1	2.04	932	9786
>tr|J9P7Y2|J9P7Y2_CANLF Angiotensin-converting enzyme OS=Canis lupus familiaris OX=9615 GN=ACE2 PE=3 SV=1	0.10	0.02	134.00	33954793.5	4	1	0	1.62	804	1041
>sp|P49822|ALBU_CANLF Albumin OS=Canis lupus familiaris OX=9615 GN=ALB PE=1 SV=3	0.30	0.25	178.50	33756533.6	7	2	1	2.30	608	490
>tr|E2R4F0|E2R4F0_CANLF Cadherin EGF LAG seven-pass G-type receptor 2 OS=Canis lupus familiaris OX=9615 GN=CELSR2 PE=3 SV=2	0.14	0.04	72.20	33750901.4	6	3	3	0.65	2919	12898
>tr|F1PBU5|F1PBU5_CANLF Non-specific serine/threonine protein kinase OS=Canis lupus familiaris OX=9615 GN=SMG1 PE=3 SV=3	0.36	0.31	167.70	33714348.1	4	1	0	0.08	3634	6898
>tr|A0A5F4C9Z9|A0A5F4C9Z9_CANLF UTP20 small subunit processome component OS=Canis lupus familiaris OX=9615 GN=UTP20 PE=4 SV=1	0.15	0.15	72.60	33460592.3	2	2	1	0.44	2758	1779
>tr|E2QXW6|E2QXW6_CANLF Patatin like phospholipase domain containing 7 OS=Canis lupus familiaris OX=9615 GN=PNPLA7 PE=3 SV=2	0.10	0.06	35.90	33412088.3	3	1	1	1.58	1456	31798
>tr|E2RGH9|E2RGH9_CANLF HECT-type E3 ubiquitin transferase OS=Canis lupus familiaris OX=9615 GN=HECTD1 PE=3 SV=2	0.10	0.06	147.40	33389415.2	2	1	0	0.31	2609	36968
>tr|F1PSM3|F1PSM3_CANLF Symplekin OS=Canis lupus familiaris OX=9615 GN=SYMPK PE=4 SV=2	0.10	0.00	29.80	33109043.8	4	2	2	0.39	1275	9214
>tr|E2QUV0|E2QUV0_CANLF Nipped-B protein OS=Canis lupus familiaris OX=9615 GN=NIPBL PE=3 SV=3	0.10	0.00	29.80	33109043.8	4	2	2	0.18	2722	14722
>tr|A0A5F4DFY1|A0A5F4DFY1_CANLF SHH signaling and ciliogenesis regulator SDCCAG8 OS=Canis lupus familiaris OX=9615 GN=SDCCAG8 PE=4 SV=1	0.40	0.08	31.50	33068258.0	17	1	1	0.59	673	17765
>tr|A0A5F4D7Y5|A0A5F4D7Y5_CANLF Pleckstrin homology, MyTH4 and FERM domain containing H1 OS=Canis lupus familiaris OX=9615 GN=PLEKHH1 PE=4 SV=1	1.08	1.02	260.40	33039458.3	5	1	0	0.30	1342	5979
>tr|A0A5F4DD58|A0A5F4DD58_CANLF Phosphoinositide phospholipase C OS=Canis lupus familiaris OX=9615 GN=PLCD3 PE=4 SV=1	0.90	0.80	205.90	33023756.5	6	1	0	0.40	741	3088
>tr|E2RN56|E2RN56_CANLF Zinc finger CCCH-type containing 13 OS=Canis lupus familiaris OX=9615 GN=ZC3H13 PE=4 SV=3	0.58	0.54	228.50	32881674.4	3	1	0	0.24	1660	1446
>tr|L7MTP6|L7MTP6_CANLF Myosin heavy chain 3 OS=Canis lupus familiaris OX=9615 GN=MYH3 PE=3 SV=1	0.10	0.03	35.90	32077979.8	3	1	0	0.26	1940	17787

>tr|E2R8U5|E2R8U5_CANLF Collagen type XXIV alpha 1 chain OS=Canis lupus familiaris OX=9615 GN=COL24A1 PE=4 SV=3	0.10	0.10	124.20	31867195.8	2	2	1	0.82	1709	25162
>tr|F1P9I2|F1P9I2_CANLF Cdk5 and Abl enzyme substrate 1 OS=Canis lupus familiaris OX=9615 GN=CABLES1 PE=4 SV=3	0.10	0.03	139.60	31695250.6	2	1	0	0.92	757	13186
>tr|F1PJY1|F1PJY1_CANLF Mannosyl-glycoprotein endo-beta-N-acetylglucosaminidase OS=Canis lupus familiaris OX=9615 GN=ENGASE PE=3 SV=3	0.10	0.02	117.80	31614381.6	4	1	1	1.74	690	32761
>sp|Q9XS65|PTGDS_CANLF Prostaglandin-H2 D-isomerase OS=Canis lupus familiaris OX=9615 GN=PTGDS PE=2 SV=1	1.34	0.77	257.70	31592600.2	9	2	1	10.47	191	165
>sp|Q9XS65|PTGDS_CANLF Prostaglandin-H2 D-isomerase OS=Canis lupus familiaris OX=9615 GN=PTGDS PE=2 SV=1	0.24	0.18	119.40	31259064.7	5	2	1	10.47	191	165
>tr|J9NTK2|J9NTK2_CANLF J domain-containing protein OS=Canis lupus familiaris OX=9615 GN=DNAJC12 PE=4 SV=2	0.57	0.53	161.40	31149079.9	3	1	0	4.72	106	2310
>tr|A0A5F4D6L9|A0A5F4D6L9_CANLF Sacsin molecular chaperone OS=Canis lupus familiaris OX=9615 GN=SACS PE=4 SV=1	0.20	0.14	226.80	30959033.6	9	4	0	0.58	4500	1444
>tr|A0A5F4DIG1|A0A5F4DIG1_CANLF Interleukin 4 induced 1 OS=Canis lupus familiaris OX=9615 GN=IL4I1 PE=4 SV=1	0.53	0.45	189.40	30842373.8	5	3	0	5.08	571	21105
>tr|E2RRP1|E2RRP1_CANLF Butyryl-CoA dehydrogenase OS=Canis lupus familiaris OX=9615 GN=IVD PE=3 SV=2	0.12	0.08	95.50	30783500.3	4	1	0	3.99	426	10739
>tr|J9P432|J9P432_CANLF Glutamine--fructose-6-phosphate transaminase (isomerizing) OS=Canis lupus familiaris OX=9615 GN=GFPT1 PE=4 SV=2	0.33	0.19	113.50	30126452.3	8	1	0	1.18	677	7191
>tr|A0A5F4DDL6|A0A5F4DDL6_CANLF Molybdenum cofactor sulfurase OS=Canis lupus familiaris OX=9615 GN=MOCOS PE=3 SV=1	0.10	0.05	129.20	30055326.6	3	1	0	0.57	876	14262
>sp|F1PRN2|MYO1D_CANLF Unconventional myosin-Id OS=Canis lupus familiaris OX=9615 GN=MYO1D PE=1 SV=2	0.51	0.49	158.60	29831028.2	2	1	0	0.30	1006	763
>sp|E2RED8|AP4M1_CANLF AP-4 complex subunit mu-1 OS=Canis lupus familiaris OX=9615 GN=AP4M1 PE=3 SV=2	0.77	0.61	208.80	29709839.5	9	1	0	0.66	452	634
>tr|A0A5F4DCA4|A0A5F4DCA4_CANLF Reverse transcriptase domain-containing protein OS=Canis lupus familiaris OX=9615 PE=4 SV=1	0.51	0.49	144.60	29591909.8	2	1	0	0.31	978	860
>sp|P62286|ASPM_CANLF Abnormal spindle-like microcephaly-associated protein homolog OS=Canis lupus familiaris OX=9615 GN=ASPM PE=2 SV=2	1.03	0.69	74.70	29560318.9	6	3	0	0.23	3469	677
>tr|F1PZB6|F1PZB6_CANLF Alkaline ceramidase OS=Canis lupus familiaris OX=9615 GN=ACER1 PE=3 SV=3	0.59	0.42	66.80	29094250.6	13	2	2	4.18	263	35976
>tr|A0A5F4DDT5|A0A5F4DDT5_CANLF Tousled like kinase 1 OS=Canis lupus familiaris OX=9615 GN=TLK1 PE=4 SV=1	0.15	0.09	157.80	29089524.1	5	1	0	0.68	734	7453
>tr|A0A5F4CJI0|A0A5F4CJI0_CANLF Folate_rec domain-containing protein OS=Canis lupus familiaris OX=9615 GN=FOLR1 PE=3 SV=1	0.61	0.57	231.20	28756277.6	3	1	0	3.14	255	10075
>sp|A2IBY8|MIP_CANLF Lens fiber major intrinsic protein OS=Canis lupus familiaris OX=9615 GN=MIP PE=2 SV=1	0.12	0.08	85.30	28513209.4	13	1	0	1.90	263	112
>tr|J9NW72|J9NW72_CANLF Sperm associated antigen 8 OS=Canis lupus familiaris OX=9615 GN=SPAG8 PE=4 SV=1	0.79	0.69	103.30	28470734.0	6	1	0	4.07	442	10371
>tr|E2RG75|E2RG75_CANLF Inactive ribonuclease-like protein 9 OS=Canis lupus familiaris OX=9615 GN=RNASE9 PE=3 SV=2	3.36	3.18	276.50	28260980.6	7	2	1	8.08	198	41734
>tr|E2RCT1|E2RCT1_CANLF WAP domain-containing protein OS=Canis lupus familiaris OX=9615 PE=4 SV=2	3.82	2.59	249.00	28068068.5	5	2	0	14.66	116	21717

>tr|J9NS28|J9NS28_CANLF RBR-type E3 ubiquitin transferase OS=Canis lupus familiaris OX=9615 GN=ANKIB1 PE=4 SV=2	0.23	0.17	62.70	27866905.8	4	1	0	2.33	988	26345
>tr|A0A5F4DIG1|A0A5F4DIG1_CANLF Interleukin 4 induced 1 OS=Canis lupus familiaris OX=9615 GN=IL4I1 PE=4 SV=1	0.78	0.70	229.40	27633961.0	4	3	0	5.08	571	21105
>sp|P49822|ALBU_CANLF Albumin OS=Canis lupus familiaris OX=9615 GN=ALB PE=1 SV=3	0.42	0.30	120.40	27519003.0	5	2	1	2.30	608	490
>tr|F1P9X0|F1P9X0_CANLF Dolichol phosphate-mannose biosynthesis regulatory protein OS=Canis lupus familiaris OX=9615 GN=DPM2 PE=3 SV=3	0.10	0.07	18.60	27294111.0	7	1	1	5.95	84	12958
>tr|A0A5F4CYM0|A0A5F4CYM0_CANLF Quiescin sulfhydryl oxidase 2 OS=Canis lupus familiaris OX=9615 GN=QSOX2 PE=4 SV=1	0.18	0.03	137.60	27257639.1	10	1	0	2.52	636	1464
>tr|Q9XSV4|Q9XSV4_CANLF CE10 protein OS=Canis lupus familiaris OX=9615 GN=ce10 PE=2 SV=1	1.99	1.83	273.70	27127890.7	7	2	0	9.09	110	41542
>tr|A0A5F4BTI7|A0A5F4BTI7_CANLF THUMP domain containing 2 OS=Canis lupus familiaris OX=9615 GN=THUMPD2 PE=3 SV=1	0.10	0.00	29.80	26970400.6	3	2	2	1.05	478	12577
>tr|E2RJ91|E2RJ91_CANLF BTB domain containing 7 OS=Canis lupus familiaris OX=9615 GN=BTBD7 PE=4 SV=1	0.10	0.00	29.80	26970400.6	3	2	2	0.44	1126	21940
>sp|O18840|ACTB_CANLF Actin, cytoplasmic 1 OS=Canis lupus familiaris OX=9615 GN=ACTB PE=2 SV=3	0.10	0.06	190.60	26426006.5	3	1	0	2.93	375	642
>tr|J9NTK2|J9NTK2_CANLF J domain-containing protein OS=Canis lupus familiaris OX=9615 GN=DNAJC12 PE=4 SV=2	0.23	0.18	162.80	26409014.4	5	1	0	4.72	106	2310
>tr|A0A5F4C887|A0A5F4C887_CANLF O-acyltransferase OS=Canis lupus familiaris OX=9615 GN=DGAT1 PE=3 SV=1	0.10	0.02	51.20	26301544.2	2	1	0	1.93	623	22414
>tr|A0A5F4D848|A0A5F4D848_CANLF Lipase I OS=Canis lupus familiaris OX=9615 GN=LIPI PE=3 SV=1	0.17	0.15	170.30	26274176.5	2	1	0	2.09	431	1169
>tr|A0A5F4C7W7|A0A5F4C7W7_CANLF Phosphatidylinositol-4,5-bisphosphate 3-kinase OS=Canis lupus familiaris OX=9615 GN=PIK3CD PE=3 SV=1	0.46	0.45	159.40	26123041.8	3	1	0	1.52	1051	18533
>tr|A0A5F4D430|A0A5F4D430_CANLF Transcription factor AP-2 gamma OS=Canis lupus familiaris OX=9615 GN=TFAP2C PE=3 SV=1	0.66	0.60	167.20	25996456.6	4	1	0	1.38	650	9642
>sp|P21842|CMA1_CANLF Chymase OS=Canis lupus familiaris OX=9615 GN=CMA1 PE=1 SV=1	0.21	0.11	89.80	25921456.0	6	1	0	0.80	249	34
>tr|F1PBJ1|F1PBJ1_CANLF Methylcytosine dioxygenase TET OS=Canis lupus familiaris OX=9615 GN=TET3 PE=3 SV=2	0.10	0.02	32.50	25839500.3	2	1	0	0.28	1795	1529
>sp|P83509|RHG35_CANLF Rho GTPase-activating protein 35 OS=Canis lupus familiaris OX=9615 GN=ARHGAP35 PE=2 SV=1	0.55	0.47	115.20	25749029.1	5	1	1	0.73	1500	323
>sp|P49822|ALBU_CANLF Albumin OS=Canis lupus familiaris OX=9615 GN=ALB PE=1 SV=3	0.11	0.05	208.20	25738417.0	4	1	0	2.30	608	490
>sp|Q28298|RRBP1_CANLF Ribosome-binding protein 1 OS=Canis lupus familiaris OX=9615 GN=RRBP1 PE=2 SV=1	0.48	0.38	83.10	25559518.3	6	1	0	0.13	1534	61
>tr|F1P704|F1P704_CANLF Zona pellucida binding protein OS=Canis lupus familiaris OX=9615 GN=ZPBP PE=3 SV=3	0.57	0.48	252.70	25536808.4	4	2	0	7.73	362	12422
>tr|E2R531|E2R531_CANLF Zinc finger protein 462 OS=Canis lupus familiaris OX=9615 GN=ZNF462 PE=4 SV=3	0.57	0.57	198.60	25529808.8	6	2	1	0.58	2398	22062
>tr|A0A5F4CJI1|A0A5F4CJI1_CANLF Kelch like family member 26 OS=Canis lupus familiaris OX=9615 GN=KLHL26 PE=4 SV=1	0.53	0.51	191.00	25251348.5	3	1	0	1.81	662	13157

>tr|F1PGF9|F1PGF9_CANLF Rho guanine nucleotide exchange factor 26 OS=Canis lupus familiaris OX=9615 GN=ARHGEF26 PE=4 SV=3	1.24	1.20	296.70	25220664.0	3	1	0	0.84	594	22876
>tr|A0A5F4D3J0|A0A5F4D3J0_CANLF Dymeclin OS=Canis lupus familiaris OX=9615 GN=DYM PE=3 SV=1	0.46	0.35	122.80	25111507.9	2	2	2	1.54	712	10307
>tr|A0A5F4BZW4|A0A5F4BZW4_CANLF Malonyl-CoA decarboxylase OS=Canis lupus familiaris OX=9615 GN=MLYCD PE=4 SV=1	0.33	0.27	199.70	24861305.6	4	1	0	1.30	461	4809
>tr|F1PM73|F1PM73_CANLF Palmitoyltransferase OS=Canis lupus familiaris OX=9615 GN=ZDHHC23 PE=3 SV=3	0.84	0.82	42.40	24749138.0	2	1	0	3.98	427	22460
>tr|A0A5F4D6L9|A0A5F4D6L9_CANLF Sacsin molecular chaperone OS=Canis lupus familiaris OX=9615 GN=SACS PE=4 SV=1	0.59	0.27	253.90	24582394.0	7	3	0	0.58	4500	1444
>tr|A0A5F4BZB3|A0A5F4BZB3_CANLF Bassoon presynaptic cytomatrix protein OS=Canis lupus familiaris OX=9615 GN=BSN PE=4 SV=1	0.35	0.35	164.40	24432488.5	4	1	0	0.44	3905	17263
>tr|F6XAM4|F6XAM4_CANLF Peptidylprolyl isomerase OS=Canis lupus familiaris OX=9615 GN=SCRN1 PE=4 SV=2	0.20	0.20	122.10	24306589.2	1	1	0	7.05	241	38880
>tr|F1PUR2|F1PUR2_CANLF CHK1 checkpoint homolog OS=Canis lupus familiaris OX=9615 GN=CHEK1 PE=3 SV=3	0.10	0.00	49.30	23535628.7	6	1	0	0.88	454	4485
>sp|Q30DN6|KDM5D_CANLF Lysine-specific demethylase 5D OS=Canis lupus familiaris OX=9615 GN=KDM5D PE=2 SV=1	0.91	0.85	187.00	23222062.1	4	1	0	0.26	1545	213
>tr|A0A5F4D9Z7|A0A5F4D9Z7_CANLF Zinc finger FYVE-type containing 19 OS=Canis lupus familiaris OX=9615 GN=ZFYVE19 PE=4 SV=1	0.10	0.07	93.40	23186453.4	2	1	0	1.53	392	13875
>tr|A0A5F4BQW4|A0A5F4BQW4_CANLF Zinc finger protein 654 OS=Canis lupus familiaris OX=9615 GN=ZNF654 PE=4 SV=1	0.11	0.10	203.50	23135482.6	4	1	1	1.03	1170	6910
>tr|A0A5F4CPU3|A0A5F4CPU3_CANLF SEC24 homolog D, COPII coat complex component OS=Canis lupus familiaris OX=9615 GN=SEC24D PE=3 SV=1	0.15	0.02	89.30	23044839.7	24	1	0	0.53	946	2125
>sp|Q28895|NPC2_CANLF NPC intracellular cholesterol transporter 2 OS=Canis lupus familiaris OX=9615 GN=NPC2 PE=2 SV=1	1.49	1.40	311.00	23018796.9	3	2	0	14.77	149	153
>tr|A0A5F4DCA4|A0A5F4DCA4_CANLF Reverse transcriptase domain-containing protein OS=Canis lupus familiaris OX=9615 PE=4 SV=1	0.54	0.48	167.20	22970793.4	4	1	0	0.31	978	860
>tr|Q2A652|Q2A652_CANLF G-protein coupled receptor OS=Canis lupus familiaris OX=9615 GN=PTGDR2 PE=2 SV=1	0.10	0.00	131.20	22483401.3	3	1	0	1.25	400	41469
>tr|F1PHQ0|F1PHQ0_CANLF Clathrin heavy chain OS=Canis lupus familiaris OX=9615 GN=CLTC PE=3 SV=3	0.16	0.14	159.40	22360460.1	2	1	1	0.65	1682	24646
>tr|Q9XSV4|Q9XSV4_CANLF CE10 protein OS=Canis lupus familiaris OX=9615 GN=ce10 PE=2 SV=1	2.93	1.90	312.30	22146957.7	9	2	0	9.09	110	41542
>tr|A0A5F4CD39|A0A5F4CD39_CANLF Centromere protein U OS=Canis lupus familiaris OX=9615 GN=CENPU PE=3 SV=1	0.10	0.03	137.40	21905303.1	3	1	1	2.68	448	33164
>tr|J9PAZ6|J9PAZ6_CANLF Hyperpolarization activated cyclic nucleotide gated potassium channel 4 OS=Canis lupus familiaris OX=9615 GN=HCN4 PE=4 SV=2	0.21	0.17	103.40	21839012.4	3	1	1	4.91	530	19838
>tr|A0A5F4CCD0|A0A5F4CCD0_CANLF Cysteine rich secretory protein 2 OS=Canis lupus familiaris OX=9615 GN=CRISP2 PE=3 SV=1	1.54	1.48	112.20	21777312.0	4	1	0	4.82	311	11017
>tr|Q9XSV4|Q9XSV4_CANLF CE10 protein OS=Canis lupus familiaris OX=9615 GN=ce10 PE=2 SV=1	0.89	0.80	185.00	21685230.8	6	2	0	9.09	110	41542
>tr|A0A5F4CVL6|A0A5F4CVL6_CANLF RB transcriptional corepressor 1 OS=Canis lupus familiaris OX=9615 GN=RB1 PE=3 SV=1	0.10	0.02	34.70	21674741.6	18	1	1	0.43	927	1490

>tr|A0A5F4D430|A0A5F4D430_CANLF Transcription factor AP-2 gamma OS=Canis lupus familiaris OX=9615 GN=TFAP2C PE=3 SV=1	0.79	0.75	173.00	21557633.4	3	1	0	1.38	650	9642
>tr|F1PLT8|F1PLT8_CANLF Sulfhydryl oxidase OS=Canis lupus familiaris OX=9615 GN=QSOX1 PE=3 SV=3	0.11	0.09	148.90	21541774.5	2	1	0	2.64	568	33056
>tr|E2R6E0|E2R6E0_CANLF Lipocln_cytosolic_FA-bd_dom domain-containing protein OS=Canis lupus familiaris OX=9615 GN=LCNL1 PE=3 SV=2	1.25	1.19	126.50	21536887.8	4	1	0	3.01	299	1932
>tr|A0A5K1V0D8|A0A5K1V0D8_CANLF Sulfatase 2 OS=Canis lupus familiaris OX=9615 GN=SULF2 PE=3 SV=1	0.10	0.00	180.00	21470834.1	4	1	0	0.35	859	1192
>tr|Q30KS5|Q30KS5_CANLF Beta-defensin 129 OS=Canis lupus familiaris OX=9615 GN=DEFB129 PE=2 SV=1	1.00	0.96	279.00	21465779.2	3	1	0	4.22	166	41730
>sp|E2RED8|AP4M1_CANLF AP-4 complex subunit mu-1 OS=Canis lupus familiaris OX=9615 GN=AP4M1 PE=3 SV=2	0.30	0.23	209.40	21406036.1	5	1	0	0.66	452	634
>tr|F6PLX8|F6PLX8_CANLF Beta-defensin OS=Canis lupus familiaris OX=9615 GN=DEFB118 PE=3 SV=1	0.31	0.27	235.50	21401548.6	3	1	0	5.85	171	34287
>sp|Q8HYV8|ASB17_CANLF Ankyrin repeat and SOCS box protein 17 OS=Canis lupus familiaris OX=9615 GN=ASB17 PE=2 SV=1	0.10	0.00	172.90	21245230.7	4	1	0	1.02	295	404
>tr|A0A5F4DDS5|A0A5F4DDS5_CANLF CD86 molecule OS=Canis lupus familiaris OX=9615 GN=CD86 PE=4 SV=1	0.49	0.47	164.60	20983189.6	2	1	0	2.22	315	17545
>tr|A0A5F4CQW0|A0A5F4CQW0_CANLF Polybromo 1 OS=Canis lupus familiaris OX=9615 GN=PBRM1 PE=4 SV=1	0.21	0.19	140.20	20883191.9	2	1	0	0.79	1510	2204
>tr|A0A5F4DCB2|A0A5F4DCB2_CANLF NOP2/Sun RNA methyltransferase 5 OS=Canis lupus familiaris OX=9615 GN=NSUN5 PE=3 SV=1	0.10	0.05	45.70	20836011.4	1	1	0	1.49	469	2010
>tr|E2RCT1|E2RCT1_CANLF WAP domain-containing protein OS=Canis lupus familiaris OX=9615 PE=4 SV=2	0.27	0.21	154.10	20742543.1	4	1	0	8.62	116	21717
>tr|A0A5F4C0S7|A0A5F4C0S7_CANLF HEAT repeat containing 5A OS=Canis lupus familiaris OX=9615 GN=HEATR5A PE=3 SV=1	0.10	0.02	93.80	20628990.4	15	1	0	0.25	1995	1753
>tr|A0A5F4CNP4|A0A5F4CNP4_CANLF ADP ribosylation factor GTPase activating protein 3 OS=Canis lupus familiaris OX=9615 GN=ARFGAP3 PE=4 SV=1	0.41	0.37	183.20	20553248.6	3	1	0	1.12	714	964
>tr|E2RE16|E2RE16_CANLF Non-specific serine/threonine protein kinase OS=Canis lupus familiaris OX=9615 GN=PAK4 PE=4 SV=1	0.29	0.12	101.60	20491308.1	10	1	0	0.84	592	12735
>tr|A0A5F4CCD0|A0A5F4CCD0_CANLF Cysteine rich secretory protein 2 OS=Canis lupus familiaris OX=9615 GN=CRISP2 PE=3 SV=1	1.05	1.01	160.70	20009199.8	3	1	0	4.82	311	11017
>tr|Q9XSV4|Q9XSV4_CANLF CE10 protein OS=Canis lupus familiaris OX=9615 GN=ce10 PE=2 SV=1	1.38	1.12	233.10	19989969.8	6	3	0	14.55	110	41542
>sp|Q5QQ50|XYLT2_CANLF Xylosyltransferase 2 OS=Canis lupus familiaris OX=9615 GN=XYLT2 PE=2 SV=1	0.20	0.18	99.80	19874604.6	2	1	0	0.35	865	388
>tr|F1PBJ1|F1PBJ1_CANLF Methylcytosine dioxygenase TET OS=Canis lupus familiaris OX=9615 GN=TET3 PE=3 SV=2	0.16	0.02	78.50	19810784.1	8	1	0	0.28	1795	1529
>tr|A0A5F4C840|A0A5F4C840_CANLF KIAA1109 OS=Canis lupus familiaris OX=9615 GN=KIAA1109 PE=4 SV=1	0.47	0.46	176.10	19808731.3	2	1	0	0.16	5041	21640
>tr|A0A5F4BXD8|A0A5F4BXD8_CANLF Matrix metallopeptidase 16 OS=Canis lupus familiaris OX=9615 GN=MMP16 PE=3 SV=1	0.26	0.26	169.00	19760675.6	1	1	0	2.65	566	6476
>sp|O46607|GPX5_CANLF Epididymal secretory glutathione peroxidase OS=Canis lupus familiaris OX=9615 GN=GPX5 PE=2 SV=1	0.73	0.71	76.00	19747744.3	2	1	0	6.79	221	564

>sp|P01002|IPSG_CANLF Double-headed protease inhibitor, submandibular gland OS=Canis lupus familiaris OX=9615 PE=1 SV=1	1.05	1.03	209.10	19708370.2	2	1	0	7.83	115	434
>tr|E2RRP1|E2RRP1_CANLF Butyryl-CoA dehydrogenase OS=Canis lupus familiaris OX=9615 GN=IVD PE=3 SV=2	0.14	0.10	80.90	19542226.8	3	1	0	3.99	426	10739
>tr|A0A5F4CCE2|A0A5F4CCE2_CANLF Pappalysin 2 OS=Canis lupus familiaris OX=9615 GN=PAPPA2 PE=3 SV=1	1.63	1.63	155.30	19488411.9	1	1	0	0.81	1722	20443
>tr|A0A5F4C2N8|A0A5F4C2N8_CANLF Sperm protamine P1 OS=Canis lupus familiaris OX=9615 PE=3 SV=1	0.75	0.71	219.00	19469318.1	3	1	0	10.00	50	7114
>tr|E2R856|E2R856_CANLF Myeloid derived growth factor OS=Canis lupus familiaris OX=9615 GN=MYDGF PE=4 SV=3	0.53	0.47	24.40	19417306.0	4	1	0	3.47	173	26349
>tr|A0A5F4CTQ6|A0A5F4CTQ6_CANLF SLC9A3 regulator 2 OS=Canis lupus familiaris OX=9615 GN=SLC9A3R2 PE=4 SV=1	0.53	0.47	24.40	19417306.0	4	1	0	1.90	315	18010
>tr|E2RKA1|E2RKA1_CANLF Tyrosine-protein kinase receptor OS=Canis lupus familiaris OX=9615 GN=NTRK2 PE=3 SV=2	0.53	0.47	24.40	19417306.0	4	1	0	0.73	822	20603
>tr|F1PEA8|F1PEA8_CANLF Phosphatidylinositol-3,4,5-trisphosphate 5-phosphatase OS=Canis lupus familiaris OX=9615 GN=INPP5D PE=3 SV=3	0.53	0.47	24.40	19417306.0	4	1	0	0.43	1399	17627
>tr|E2R079|E2R079_CANLF Serpin family B member 2 OS=Canis lupus familiaris OX=9615 GN=SERPINB2 PE=3 SV=2	0.20	0.20	150.10	19354015.4	1	1	0	1.92	416	21385
>tr|F1PR54|F1PR54_CANLF Lactotransferrin OS=Canis lupus familiaris OX=9615 GN=LTF PE=3 SV=1	15.13	3.21	309.60	19326649.7	43	9	0	14.41	708	40436
>tr|A0A5F4CHN1|A0A5F4CHN1_CANLF Serine/arginine repetitive matrix 2 OS=Canis lupus familiaris OX=9615 GN=SRRM2 PE=4 SV=1	0.66	0.66	197.10	19302627.6	1	1	0	0.16	2564	3180
>tr|J9P0B4|J9P0B4_CANLF Tudor domain containing 15 OS=Canis lupus familiaris OX=9615 GN=TDRD15 PE=4 SV=2	0.10	0.01	151.50	19087152.4	9	1	0	0.57	2105	4188
>tr|A0A5F4CIW5|A0A5F4CIW5_CANLF Hexokinase OS=Canis lupus familiaris OX=9615 GN=HK1 PE=3 SV=1	0.70	0.66	153.20	18902666.7	3	1	0	0.48	839	1598
>tr|Q9XSV4|Q9XSV4_CANLF CE10 protein OS=Canis lupus familiaris OX=9615 GN=ce10 PE=2 SV=1	2.04	1.77	213.50	18885638.8	7	3	0	14.55	110	41542
>sp|Q30DN6|KDM5D_CANLF Lysine-specific demethylase 5D OS=Canis lupus familiaris OX=9615 GN=KDM5D PE=2 SV=1	0.75	0.71	167.90	18831626.3	3	1	0	0.26	1545	213
>tr|E2R5M1|E2R5M1_CANLF Filamin binding LIM protein 1 OS=Canis lupus familiaris OX=9615 GN=FBLIM1 PE=4 SV=3	0.17	0.17	11.10	18544122.8	2	1	1	4.90	388	12864
>tr|E2RLU6|E2RLU6_CANLF G-protein coupled receptor 139 OS=Canis lupus familiaris OX=9615 GN=GPR139 PE=2 SV=1	0.35	0.35	52.40	18492944.3	1	1	0	6.82	352	41386
>tr|J9NTK2|J9NTK2_CANLF J domain-containing protein OS=Canis lupus familiaris OX=9615 GN=DNAJC12 PE=4 SV=2	0.39	0.35	152.50	18410976.8	3	1	0	4.72	106	2310
>sp|P49822|ALBU_CANLF Albumin OS=Canis lupus familiaris OX=9615 GN=ALB PE=1 SV=3	13.42	2.87	336.30	18325290.9	58	12	1	23.85	608	490
>tr|A0A5F4DIQ5|A0A5F4DIQ5_CANLF Helicase with zinc finger 2 OS=Canis lupus familiaris OX=9615 GN=HELZ2 PE=4 SV=1	0.48	0.43	165.00	18310220.9	4	2	0	0.85	2929	1253
>tr|A0A5F4C7W7|A0A5F4C7W7_CANLF Phosphatidylinositol-4,5-bisphosphate 3-kinase OS=Canis lupus familiaris OX=9615 GN=PIK3CD PE=3 SV=1	0.47	0.41	22.60	18206838.5	4	1	0	0.57	1051	18533
>tr|J9P0B4|J9P0B4_CANLF Tudor domain containing 15 OS=Canis lupus familiaris OX=9615 GN=TDRD15 PE=4 SV=2	0.69	0.59	215.90	18195964.5	6	1	0	0.57	2105	4188

>tr|J9NS28|J9NS28_CANLF RBR-type E3 ubiquitin transferase OS=Canis lupus familiaris OX=9615 GN=ANKIB1 PE=4 SV=2	0.10	0.06	47.20	18191412.1	1	1	0	2.33	988	26345
>tr|A0A5F4BZ61|A0A5F4BZ61_CANLF G_PROTEIN_RECEP_F1_2 domain-containing protein OS=Canis lupus familiaris OX=9615 GN=OR5D13 PE=4 SV=1	0.10	0.00	57.10	18121586.8	2	1	0	1.70	294	37537
>tr|A0A5F4BUA6|A0A5F4BUA6_CANLF NCK associated protein 5 OS=Canis lupus familiaris OX=9615 GN=NCKAP5 PE=4 SV=1	0.13	0.14	84.30	18095631.5	1	1	0	0.66	1977	5785
>sp|F1PRN2|MYO1D_CANLF Unconventional myosin-Id OS=Canis lupus familiaris OX=9615 GN=MYO1D PE=1 SV=2	0.82	0.74	171.10	18091275.2	5	1	0	0.30	1006	763
>sp|P23685|NAC1_CANLF Sodium/calcium exchanger 1 OS=Canis lupus familiaris OX=9615 GN=SLC8A1 PE=1 SV=1	0.13	0.09	151.40	18090485.6	3	1	0	1.03	970	764
>tr|E2RCT1|E2RCT1_CANLF WAP domain-containing protein OS=Canis lupus familiaris OX=9615 PE=4 SV=2	0.43	0.40	186.10	17984233.1	3	1	0	14.66	116	21717
>tr|E2R6E0|E2R6E0_CANLF Lipocln_cytosolic_FA-bd_dom domain-containing protein OS=Canis lupus familiaris OX=9615 GN=LCNL1 PE=3 SV=2	2.36	1.74	95.80	17961992.8	3	2	0	7.69	299	1932
>tr|E2RIH1|E2RIH1_CANLF DOP1 leucine zipper like protein B OS=Canis lupus familiaris OX=9615 GN=DOP1B PE=3 SV=3	1.18	1.18	47.30	17933746.1	1	1	0	1.20	2259	7751
>tr|A0A5F4C7P9|A0A5F4C7P9_CANLF Beta-2-microglobulin OS=Canis lupus familiaris OX=9615 GN=B2M PE=4 SV=1	0.40	0.37	107.20	17663412.7	3	1	0	12.15	107	10611
>tr|E2RH77|E2RH77_CANLF Ferric oxidoreductase domain-containing protein OS=Canis lupus familiaris OX=9615 GN=STEAP1 PE=4 SV=2	0.10	0.02	86.20	17584984.2	1	1	0	2.18	596	22587
>tr|F1P8J6|F1P8J6_CANLF RNA helicase OS=Canis lupus familiaris OX=9615 GN=DDX55 PE=3 SV=3	0.10	0.06	116.00	17441535.0	1	1	0	1.58	568	8934
>sp|Q6AW47|EST5A_CANLF Carboxylesterase 5A OS=Canis lupus familiaris OX=9615 GN=CES5A PE=2 SV=1	0.42	0.38	237.10	17411428.4	3	1	0	0.87	575	629
>sp|Q9TU53|CUBN_CANLF Cubilin OS=Canis lupus familiaris OX=9615 GN=CUBN PE=1 SV=1	0.10	0.05	188.70	17410876.3	3	1	0	0.08	3620	406
>tr|Q30KS5|Q30KS5_CANLF Beta-defensin 129 OS=Canis lupus familiaris OX=9615 GN=DEFB129 PE=2 SV=1	3.20	3.18	273.50	17335003.1	2	1	0	4.22	166	41730
>tr|F1Q064|F1Q064_CANLF WSC domain containing 2 OS=Canis lupus familiaris OX=9615 GN=WSCD2 PE=3 SV=2	1.20	0.81	197.70	17328528.2	3	2	0	2.66	564	5749
>tr|cc|E2RSV7_CANLF Nuclear pore complex protein Nup93 OS=Canis lupus familiaris OX=9615 GN=NUP93 PE=3 SV=2	0.43	0.36	151.30	17288988.2	5	2	1	2.16	925	13752
>tr|A0A5F4BTW9|A0A5F4BTW9_CANLF Adhesion G protein-coupled receptor L2 OS=Canis lupus familiaris OX=9615 GN=ADGRL2 PE=4 SV=1	0.19	0.19	128.40	17274808.2	1	1	0	0.47	1474	2238
>tr|A0A5F4CTH2|A0A5F4CTH2_CANLF FA complementation group I OS=Canis lupus familiaris OX=9615 GN=FANCI PE=4 SV=1	0.35	0.35	120.80	17259280.1	1	1	0	0.85	1404	5579
>tr|F1P6B8|F1P6B8_CANLF Intraflagellar transport protein 57 homolog OS=Canis lupus familiaris OX=9615 GN=IFT57 PE=3 SV=3	0.10	0.03	197.60	17239961.0	3	1	0	0.62	482	4757
>sp|Q7YRB7|AOFB_CANLF Amine oxidase [flavin-containing] B OS=Canis lupus familiaris OX=9615 GN=MAOB PE=2 SV=3	0.45	0.45	128.50	17226955.8	1	1	0	0.77	520	646
>tr|E2RDS7|E2RDS7_CANLF Zinc finger protein 398 OS=Canis lupus familiaris OX=9615 GN=ZNF398 PE=4 SV=2	0.16	0.12	157.40	17209915.4	3	1	0	1.40	642	44996
>sp|Q9TU69|GHR_CANLF Growth hormone receptor OS=Canis lupus familiaris OX=9615 GN=GHR PE=2 SV=1	0.12	0.10	140.50	17128942.5	2	1	0	1.72	638	541

>tr|A0A5F4C546|A0A5F4C546_CANLF Solute carrier family 25 member 17 OS=Canis lupus familiaris OX=9615 GN=SLC25A17 PE=3 SV=1	0.38	0.38	97.60	17096286.3	1	1	0	5.83	223	39398
>tr|A0A5F4DD58|A0A5F4DD58_CANLF Phosphoinositide phospholipase C OS=Canis lupus familiaris OX=9615 GN=PLCD3 PE=4 SV=1	0.42	0.36	144.70	16940464.9	5	2	0	0.94	741	3088
>tr|E2QUM4|E2QUM4_CANLF Pseudopodium enriched atypical kinase 1 OS=Canis lupus familiaris OX=9615 GN=PEAK1 PE=4 SV=3	0.52	0.52	126.80	16893351.7	3	2	2	1.73	1674	5482
>tr|F1PTB2|F1PTB2_CANLF Interleukin 6 signal transducer OS=Canis lupus familiaris OX=9615 GN=IL6ST PE=3 SV=3	0.44	0.42	174.90	16803218.6	2	1	0	0.35	867	4860
>sp|Q5TJE1|DAXX_CANLF Death domain-associated protein 6 OS=Canis lupus familiaris OX=9615 GN=DAXX PE=3 SV=1	0.95	0.91	215.20	16793805.3	3	1	0	0.54	737	429
>tr|E2RSW3|E2RSW3_CANLF Ferric chelate reductase 1 OS=Canis lupus familiaris OX=9615 GN=FRRS1 PE=3 SV=3	0.10	0.02	63.70	16726431.7	2	1	0	0.66	605	40434
>tr|A0A5F4BQE1|A0A5F4BQE1_CANLF Vacuolar fusion protein MON1 homolog OS=Canis lupus familiaris OX=9615 GN=MON1B PE=3 SV=1	0.67	0.67	145.60	16642869.2	1	1	0	2.32	561	9974
>tr|Q9XSV4|Q9XSV4_CANLF CE10 protein OS=Canis lupus familiaris OX=9615 GN=ce10 PE=2 SV=1	4.37	2.16	334.50	16557162.0	53	2	0	9.09	110	41542
>tr|F1PGL2|F1PGL2_CANLF RNA helicase OS=Canis lupus familiaris OX=9615 GN=DHX16 PE=4 SV=2	0.15	0.13	40.10	16524257.0	2	1	0	1.73	1042	43089
>tr|J9P4Q5|J9P4Q5_CANLF HECT-type E3 ubiquitin transferase OS=Canis lupus familiaris OX=9615 GN=HECW1 PE=4 SV=2	0.10	0.03	105.10	16497583.2	1	1	0	1.26	1587	3937
>tr|A0A5F4D8I6|A0A5F4D8I6_CANLF Phospholipase A2 receptor 1 OS=Canis lupus familiaris OX=9615 GN=PLA2R1 PE=4 SV=1	0.53	0.41	121.60	16419922.9	7	1	0	0.50	1394	6796
>sp|Q9TU69|GHR_CANLF Growth hormone receptor OS=Canis lupus familiaris OX=9615 GN=GHR PE=2 SV=1	0.21	0.19	112.60	16417944.7	2	1	0	1.72	638	541
>sp|Q9XSU7|RL27_CANLF 60S ribosomal protein L27 OS=Canis lupus familiaris OX=9615 GN=RPL27 PE=2 SV=3	0.10	0.00	52.70	16415776.7	38	1	0	3.68	136	314
>tr|J9NZH4|J9NZH4_CANLF NTR domain-containing protein OS=Canis lupus familiaris OX=9615 GN=LOC102154527 PE=3 SV=2	0.27	0.25	188.50	16404036.1	2	1	0	8.14	221	39505
>tr|F6XCF8|F6XCF8_CANLF Vacuolar protein sorting-associated protein 54 OS=Canis lupus familiaris OX=9615 GN=VPS54 PE=3 SV=1	0.10	0.05	34.80	16350850.2	3	1	0	2.38	965	34050
>tr|A0A5F4D430|A0A5F4D430_CANLF Transcription factor AP-2 gamma OS=Canis lupus familiaris OX=9615 GN=TFAP2C PE=3 SV=1	0.34	0.34	165.20	16336580.4	1	1	0	1.38	650	9642
>tr|A0A5F4C7E7|A0A5F4C7E7_CANLF 3-beta-hydroxysterol Delta (14)-reductase OS=Canis lupus familiaris OX=9615 GN=LBR PE=3 SV=1	0.10	0.06	140.90	16271171.0	1	1	0	1.04	576	1034
>tr|A0A5F4DKM7|A0A5F4DKM7_CANLF Terminal uridylyl transferase 4 OS=Canis lupus familiaris OX=9615 GN=TUT4 PE=4 SV=1	0.10	0.05	77.60	16269702.7	3	1	0	0.19	1611	941
>tr|A0A5F4CJI0|A0A5F4CJI0_CANLF Folate_rec domain-containing protein OS=Canis lupus familiaris OX=9615 GN=FOLR1 PE=3 SV=1	0.35	0.33	203.80	16184156.6	2	1	0	3.14	255	10075
>sp|Q5TJE1|DAXX_CANLF Death domain-associated protein 6 OS=Canis lupus familiaris OX=9615 GN=DAXX PE=3 SV=1	0.49	0.47	196.80	16176258.2	3	1	0	0.54	737	429
>tr|A0A5F4DJM5|A0A5F4DJM5_CANLF Tyrosine-protein phosphatase non-receptor type OS=Canis lupus familiaris OX=9615 GN=PTPN21 PE=3 SV=1	0.32	0.28	165.80	16149442.2	3	1	0	0.55	1096	5460
>tr|A0A5F4D9Z7|A0A5F4D9Z7_CANLF Zinc finger FYVE-type containing 19 OS=Canis lupus familiaris OX=9615 GN=ZFYVE19 PE=4 SV=1	0.74	0.75	131.70	16130682.1	1	1	0	1.53	392	13875

>tr|A0A5F4D6G2|A0A5F4D6G2_CANLF SMG7 nonsense mediated mRNA decay factor OS=Canis lupus familiaris OX=9615 GN=SMG7 PE=4 SV=1	0.47	0.45	267.80	16124928.9	2	1	0	0.43	1175	2075
>tr|A0A5F4D2W3|A0A5F4D2W3_CANLF Growth factor receptor bound protein 2 OS=Canis lupus familiaris OX=9615 GN=GRB2 PE=4 SV=1	0.15	0.13	123.40	15860300.0	2	2	2	4.95	222	13178
>tr|A0A5F4BSQ1|A0A5F4BSQ1_CANLF Dystonin OS=Canis lupus familiaris OX=9615 GN=DST PE=4 SV=1	0.17	0.18	114.10	15848542.5	1	1	0	0.17	7514	9739
>tr|A0A5F4CK18|A0A5F4CK18_CANLF Transcription elongation factor spt6 OS=Canis lupus familiaris OX=9615 GN=SUPT6H PE=3 SV=1	0.10	0.08	153.00	15837347.8	1	1	0	0.44	1602	3373
>tr|E2RIK1|E2RIK1_CANLF Phosphatidylinositol-4-phosphate 3-kinase OS=Canis lupus familiaris OX=9615 GN=PIK3C2G PE=3 SV=3	0.57	0.53	38.70	15731759.4	3	1	0	0.42	1445	5924
>tr|F1PGF9|F1PGF9_CANLF Rho guanine nucleotide exchange factor 26 OS=Canis lupus familiaris OX=9615 GN=ARHGEF26 PE=4 SV=3	0.62	0.60	275.30	15678307.1	2	1	0	0.84	594	22876
>sp|P49822|ALBU_CANLF Albumin OS=Canis lupus familiaris OX=9615 GN=ALB PE=1 SV=3	0.14	0.14	69.70	15672286.1	1	1	0	1.97	608	490
>tr|A0A5F4D952|A0A5F4D952_CANLF FAT atypical cadherin 1 OS=Canis lupus familiaris OX=9615 GN=FAT1 PE=4 SV=1	0.42	0.43	163.90	15585287.4	1	1	0	0.26	4614	14224
>tr|E2RHG5|E2RHG5_CANLF Nudix hydrolase 3 OS=Canis lupus familiaris OX=9615 GN=NUDT3 PE=4 SV=1	0.45	0.39	136.00	15525663.2	4	1	0	6.40	172	21894
>tr|A0A5F4CLU1|A0A5F4CLU1_CANLF Superoxide dismutase [Cu-Zn] OS=Canis lupus familiaris OX=9615 GN=SOD1 PE=3 SV=1	0.95	0.91	185.70	15423358.4	3	1	0	7.09	141	25417
>tr|F1PVA2|F1PVA2_CANLF Adhesion G protein-coupled receptor V1 OS=Canis lupus familiaris OX=9615 GN=ADGRV1 PE=4 SV=3	0.32	0.33	173.40	15282101.3	1	1	0	0.08	6300	19804
>tr|F1PBJ1|F1PBJ1_CANLF Methylcytosine dioxygenase TET OS=Canis lupus familiaris OX=9615 GN=TET3 PE=3 SV=2	0.10	0.00	74.20	15265630.4	10	1	0	0.28	1795	1529
>tr|A0A5F4CI67|A0A5F4CI67_CANLF Mannanase OS=Canis lupus familiaris OX=9615 GN=MANBA PE=4 SV=1	0.64	0.62	197.50	15157777.5	2	1	0	1.45	828	37800
>tr|A0A5F4CQZ1|A0A5F4CQZ1_CANLF LIM domain 7 OS=Canis lupus familiaris OX=9615 GN=LMO7 PE=4 SV=1	0.10	0.00	89.10	15154154.7	4	1	1	0.28	1806	10523
>sp|O46607|GPX5_CANLF Epididymal secretory glutathione peroxidase OS=Canis lupus familiaris OX=9615 GN=GPX5 PE=2 SV=1	1.01	0.85	233.10	15096457.5	4	2	0	8.60	221	564
>tr|E2RHP1|E2RHP1_CANLF T cell activation inhibitor, mitochondrial OS=Canis lupus familiaris OX=9615 GN=TCAIM PE=4 SV=1	0.24	0.20	84.40	14970549.4	3	1	0	2.01	497	25418
>tr|E2QUV3|E2QUV3_CANLF Alpha-2-HS-glycoprotein OS=Canis lupus familiaris OX=9615 GN=AHSG PE=4 SV=2	0.70	0.66	59.90	14914548.0	3	1	0	5.48	365	20747
>tr|A0A5F4DKA1|A0A5F4DKA1_CANLF Zinc finger protein 335 OS=Canis lupus familiaris OX=9615 GN=ZNF335 PE=4 SV=1	0.14	0.12	172.80	14857466.3	3	2	0	1.09	1380	32203
>tr|F6PLX8|F6PLX8_CANLF Beta-defensin OS=Canis lupus familiaris OX=9615 GN=DEFB118 PE=3 SV=1	0.10	0.03	199.60	14835226.8	2	1	0	5.85	171	34287
>tr|E2RE16|E2RE16_CANLF Non-specific serine/threonine protein kinase OS=Canis lupus familiaris OX=9615 GN=PAK4 PE=4 SV=1	0.10	0.00	57.00	14718612.4	34	1	0	0.84	592	12735
>tr|A0A5F4BT89|A0A5F4BT89_CANLF Olfactory receptor OS=Canis lupus familiaris OX=9615 GN=OR5W6 PE=3 SV=1	0.10	0.02	111.40	14625569.8	1	1	1	6.95	302	29923
>tr|F1PFZ5|F1PFZ5_CANLF Milk fat globule EGF and factor V/VIII domain containing OS=Canis lupus familiaris OX=9615 GN=MFGE8 PE=4 SV=3	0.82	0.80	236.70	14614665.7	3	2	1	8.88	428	7079

>tr|A0A5F4D4R7|A0A5F4D4R7_CANLF TIAM Rac1 associated GEF 1 OS=Canis lupus familiaris OX=9615 GN=TIAM1 PE=4 SV=1	0.35	0.33	82.90	14458396.2	2	1	0	1.06	1601	3447
>tr|F1PGX9|F1PGX9_CANLF Solute carrier family 2 member 13 OS=Canis lupus familiaris OX=9615 GN=SLC2A13 PE=3 SV=3	0.13	0.13	157.40	14454989.0	1	1	0	0.96	624	37617
>tr|J9P199|J9P199_CANLF tRNA (guanine(37)-N1)-methyltransferase OS=Canis lupus familiaris OX=9615 GN=TRMT5 PE=3 SV=2	0.16	0.14	130.30	14347364.2	2	1	0	2.02	495	29429
>tr|A0A5F4D9S5|A0A5F4D9S5_CANLF Hyaluronoglucosaminidase OS=Canis lupus familiaris OX=9615 GN=CEMIP PE=3 SV=1	1.79	1.77	182.50	14236079.5	2	1	0	0.24	1684	9775
>tr|A0A5F4D430|A0A5F4D430_CANLF Transcription factor AP-2 gamma OS=Canis lupus familiaris OX=9615 GN=TFAP2C PE=3 SV=1	1.07	1.05	175.10	14213859.7	2	1	0	1.38	650	9642
>tr|E2RT65|E2RT65_CANLF Phosphoglycerate mutase OS=Canis lupus familiaris OX=9615 GN=PI4K2A PE=3 SV=2	0.56	0.52	216.80	14213607.7	3	1	0	4.33	254	15155
>sp|Q2PQH8|GDE_CANLF Glycogen debranching enzyme OS=Canis lupus familiaris OX=9615 GN=AGL PE=2 SV=1	0.36	0.36	102.20	14211650.4	1	1	0	0.13	1533	23
>tr|A0A5F4DCA4|A0A5F4DCA4_CANLF Reverse transcriptase domain-containing protein OS=Canis lupus familiaris OX=9615 PE=4 SV=1	0.47	0.43	157.00	14021031.5	3	1	0	0.31	978	860
>tr|A0A5F4CGB9|A0A5F4CGB9_CANLF Decapping mRNA 1B OS=Canis lupus familiaris OX=9615 GN=DCP1B PE=3 SV=1	0.10	0.03	74.50	13938089.4	4	1	0	0.36	1115	990
>sp|Q28894|WFDC2_CANLF WAP four-disulfide core domain protein 2 OS=Canis lupus familiaris OX=9615 GN=WFDC2 PE=2 SV=1	3.64	3.62	426.70	13881147.4	2	1	0	6.45	124	53
>tr|E2R6E0|E2R6E0_CANLF Lipocln_cytosolic_FA-bd_dom domain-containing protein OS=Canis lupus familiaris OX=9615 GN=LCNL1 PE=3 SV=2	1.64	1.60	207.90	13803649.9	3	1	0	3.68	299	1932
>sp|Q8WMU5|FZD6_CANLF Frizzled-6 OS=Canis lupus familiaris OX=9615 GN=FZD6 PE=2 SV=1	0.45	0.43	137.10	13681349.2	2	1	0	0.42	712	593
>tr|A0A5F4CCD0|A0A5F4CCD0_CANLF Cysteine rich secretory protein 2 OS=Canis lupus familiaris OX=9615 GN=CRISP2 PE=3 SV=1	0.74	0.72	107.80	13629738.6	2	1	0	4.82	311	11017
>tr|J9NYC7|J9NYC7_CANLF Dynein axonemal heavy chain 12 OS=Canis lupus familiaris OX=9615 GN=DNAH12 PE=3 SV=1	0.23	0.19	122.10	13622353.3	3	1	0	0.33	3960	15992
>sp|Q28895|NPC2_CANLF NPC intracellular cholesterol transporter 2 OS=Canis lupus familiaris OX=9615 GN=NPC2 PE=2 SV=1	0.10	0.02	137.50	13609297.8	2	2	0	21.48	149	153
>tr|J9NSS6|J9NSS6_CANLF DNA helicase OS=Canis lupus familiaris OX=9615 GN=CHD2 PE=4 SV=2	0.10	0.00	46.00	13315861.0	30	1	0	0.28	1780	1264
>tr|A0A5F4D9S5|A0A5F4D9S5_CANLF Hyaluronoglucosaminidase OS=Canis lupus familiaris OX=9615 GN=CEMIP PE=3 SV=1	0.48	0.46	220.70	13297319.1	2	1	0	0.24	1684	9775
>tr|A0A5F4BQ68|A0A5F4BQ68_CANLF Interferon-induced GTP-binding protein Mx1 OS=Canis lupus familiaris OX=9615 GN=MX2 PE=3 SV=1	0.53	0.51	114.80	13272317.3	2	1	1	2.47	729	13816
>sp|P01002|IPSG_CANLF Double-headed protease inhibitor, submandibular gland OS=Canis lupus familiaris OX=9615 PE=1 SV=1	0.80	0.78	183.80	13232528.2	2	1	0	7.83	115	434
>tr|J9P2T7|J9P2T7_CANLF 26S proteasome non-ATPase regulatory subunit 5 OS=Canis lupus familiaris OX=9615 PE=4 SV=1	0.89	0.89	76.80	13195937.6	1	1	0	1.95	461	23870
>tr|F1PA52|F1PA52_CANLF Hyperpolarization activated cyclic nucleotide gated potassium and sodium channel 2 OS=Canis lupus familiaris OX=9615 GN=HCN2 PE=3 SV=3	0.54	0.54	191.20	13158030.9	1	1	0	0.73	821	34579
>tr|A0A5F4CCD0|A0A5F4CCD0_CANLF Cysteine rich secretory protein 2 OS=Canis lupus familiaris OX=9615 GN=CRISP2 PE=3 SV=1	0.13	0.13	174.10	13099870.7	1	1	0	2.25	311	11017

>tr|J9NS29|J9NS29_CANLF Cystatin domain-containing protein OS=Canis lupus familiaris OX=9615 GN=LOC607874 PE=4 SV=2	0.77	0.75	186.80	13060868.3	2	1	0	6.39	313	30016
>tr|A0A5F4DCZ9|A0A5F4DCZ9_CANLF Kinase D interacting substrate 220 OS=Canis lupus familiaris OX=9615 GN=KIDINS220 PE=4 SV=1	0.16	0.16	101.20	12938678.3	1	1	0	0.60	1678	1923
>sp|F1PRN2|MYO1D_CANLF Unconventional myosin-Id OS=Canis lupus familiaris OX=9615 GN=MYO1D PE=1 SV=2	0.37	0.37	102.70	12925775.1	1	1	0	0.30	1006	763
>tr|A0A5F4DHH0|A0A5F4DHH0_CANLF ATP binding cassette subfamily A member 1 OS=Canis lupus familiaris OX=9615 GN=ABCA1 PE=4 SV=1	0.10	0.03	41.20	12918943.9	1	1	0	0.23	2175	3709
>tr|F1PIS0|F1PIS0_CANLF Ryanodine receptor 1 OS=Canis lupus familiaris OX=9615 GN=RYR1 PE=4 SV=3	0.10	0.03	41.20	12918943.9	1	1	0	0.10	5038	8330
>tr|E2RC02|E2RC02_CANLF GDP-D-mannose dehydratase OS=Canis lupus familiaris OX=9615 GN=GMDS PE=3 SV=3	0.10	0.01	15.90	12892457.3	11	1	1	1.15	349	5434
>tr|E2RHG5|E2RHG5_CANLF Nudix hydrolase 3 OS=Canis lupus familiaris OX=9615 GN=NUDT3 PE=4 SV=1	0.26	0.20	133.00	12886224.2	4	1	0	6.40	172	21894
>tr|J9P6S2|J9P6S2_CANLF Solute carrier family 12 member 4 OS=Canis lupus familiaris OX=9615 GN=SLC12A4 PE=3 SV=2	1.05	1.05	153.30	12860021.1	2	2	1	1.33	1055	3880
>tr|E2R6E0|E2R6E0_CANLF Lipocln_cytosolic_FA-bd_dom domain-containing protein OS=Canis lupus familiaris OX=9615 GN=LCNL1 PE=3 SV=2	2.66	1.39	209.50	12838532.2	3	2	0	6.69	299	1932
>sp|Q5TJE1|DAXX_CANLF Death domain-associated protein 6 OS=Canis lupus familiaris OX=9615 GN=DAXX PE=3 SV=1	0.34	0.33	171.30	12837981.6	2	1	0	0.41	737	429
>tr|J9NS28|J9NS28_CANLF RBR-type E3 ubiquitin transferase OS=Canis lupus familiaris OX=9615 GN=ANKIB1 PE=4 SV=2	0.45	0.45	71.40	12776431.9	1	1	0	2.33	988	26345
>tr|A0A5F4BPM5|A0A5F4BPM5_CANLF ILK associated serine/threonine phosphatase OS=Canis lupus familiaris OX=9615 GN=ILKAP PE=3 SV=1	1.51	1.45	218.50	12669300.9	4	1	1	2.79	359	7963
>tr|J9NSK0|J9NSK0_CANLF Basic helix-loop-helix family member b9 OS=Canis lupus familiaris OX=9615 GN=BHLHB9 PE=3 SV=1	0.39	0.37	67.30	12627010.9	2	1	0	1.09	550	35937
>sp|Q9TU53|CUBN_CANLF Cubilin OS=Canis lupus familiaris OX=9615 GN=CUBN PE=1 SV=1	0.10	0.08	32.90	12500960.0	2	1	0	0.14	3620	406
>tr|J9NYC7|J9NYC7_CANLF Dynein axonemal heavy chain 12 OS=Canis lupus familiaris OX=9615 GN=DNAH12 PE=3 SV=1	0.34	0.32	141.90	12376983.9	2	1	0	0.33	3960	15992
>tr|F1P704|F1P704_CANLF Zona pellucida binding protein OS=Canis lupus familiaris OX=9615 GN=ZPBP PE=3 SV=3	0.10	0.04	220.30	12326937.1	2	1	0	2.76	362	12422
>tr|A0A5F4CCF5|A0A5F4CCF5_CANLF Interleukin 6 signal transducer OS=Canis lupus familiaris OX=9615 GN=IL6ST PE=4 SV=1	0.10	0.02	83.60	12292416.5	2	1	1	34.85	66	3968
>tr|F1PFZ5|F1PFZ5_CANLF Milk fat globule EGF and factor V/VIII domain containing OS=Canis lupus familiaris OX=9615 GN=MFGE8 PE=4 SV=3	1.63	1.59	272.90	12209066.7	3	1	0	2.57	428	7079
>tr|A0A5F4D020|A0A5F4D020_CANLF VWFA domain-containing protein OS=Canis lupus familiaris OX=9615 PE=3 SV=1	0.10	0.00	150.60	12192369.0	2	1	0	1.00	798	27451
>tr|F1PBU5|F1PBU5_CANLF Non-specific serine/threonine protein kinase OS=Canis lupus familiaris OX=9615 GN=SMG1 PE=3 SV=3	0.15	0.15	159.40	12097641.9	1	1	0	0.08	3634	6898
>tr|A0A5F4CJD2|A0A5F4CJD2_CANLF Chloride channel protein OS=Canis lupus familiaris OX=9615 GN=CLCN7 PE=3 SV=1	0.10	0.00	16.80	12089698.2	1	1	0	0.64	785	6893
>sp|P23685|NAC1_CANLF Sodium/calcium exchanger 1 OS=Canis lupus familiaris OX=9615 GN=SLC8A1 PE=1 SV=1	0.35	0.36	147.30	12080140.7	1	1	0	1.03	970	764

>tr|J9P9K7|J9P9K7_CANLF Glycylpeptide N-tetradecanoyltransferase OS=Canis lupus familiaris OX=9615 GN=NMT2 PE=3 SV=2	0.31	0.29	76.10	12076877.6	2	1	0	3.16	507	19368
>tr|A0A5F4CGN3|A0A5F4CGN3_CANLF Mitogen-activated protein kinase OS=Canis lupus familiaris OX=9615 GN=MAPK10 PE=3 SV=1	0.45	0.43	121.60	12033344.4	2	1	0	0.86	464	863
>tr|A0A5F4CHN1|A0A5F4CHN1_CANLF Serine/arginine repetitive matrix 2 OS=Canis lupus familiaris OX=9615 GN=SRRM2 PE=4 SV=1	1.55	1.24	197.70	11997739.7	6	2	0	0.27	2564	3180
>tr|A0A5F4D0W4|A0A5F4D0W4_CANLF Glutathione-dependent dehydroascorbate reductase OS=Canis lupus familiaris OX=9615 GN=GSTO2 PE=3 SV=1	0.17	0.17	87.70	11986125.6	1	1	0	4.15	337	26941
>tr|A0A5F4BNS6|A0A5F4BNS6_CANLF Transcription activation suppressor family member 2 OS=Canis lupus familiaris OX=9615 GN=TASOR2 PE=3 SV=1	0.19	0.17	57.80	11952164.7	2	1	0	0.70	2724	1014
>tr|F1PS80|F1PS80_CANLF Protein phosphatase 4 regulatory subunit 1 OS=Canis lupus familiaris OX=9615 GN=PPP4R1 PE=4 SV=3	0.10	0.00	17.90	11836060.3	1	1	0	0.56	887	2666
>tr|F1Q455|F1Q455_CANLF PKHD1 like 1 OS=Canis lupus familiaris OX=9615 GN=PKHD1L1 PE=4 SV=3	0.10	0.05	198.30	11758293.5	2	1	0	0.07	4263	3461
>tr|A0A5F4D9S5|A0A5F4D9S5_CANLF Hyaluronoglucosaminidase OS=Canis lupus familiaris OX=9615 GN=CEMIP PE=3 SV=1	0.46	0.44	191.60	11703568.0	2	1	0	0.24	1684	9775
>tr|A0A5F4CQE4|A0A5F4CQE4_CANLF LARGE xylosyl- and glucuronyltransferase 1 OS=Canis lupus familiaris OX=9615 GN=LARGE1 PE=4 SV=1	0.10	0.02	24.30	11686654.1	9	1	0	0.70	714	7538
>tr|E2R4A0|E2R4A0_CANLF Actin-like protein 7B OS=Canis lupus familiaris OX=9615 GN=ACTL7B PE=3 SV=2	0.33	0.31	150.80	11667250.0	2	1	1	2.42	454	3066
>tr|A0A5F4DIQ5|A0A5F4DIQ5_CANLF Helicase with zinc finger 2 OS=Canis lupus familiaris OX=9615 GN=HELZ2 PE=4 SV=1	0.10	0.03	231.40	11625071.6	2	1	0	0.14	2929	1253
>tr|Q9XSV4|Q9XSV4_CANLF CE10 protein OS=Canis lupus familiaris OX=9615 GN=ce10 PE=2 SV=1	0.68	0.56	153.40	11582539.1	7	1	0	8.18	110	41542
>sp|O46607|GPX5_CANLF Epididymal secretory glutathione peroxidase OS=Canis lupus familiaris OX=9615 GN=GPX5 PE=2 SV=1	0.43	0.43	85.50	11558729.4	1	1	0	6.79	221	564
>tr|E2RHS5|E2RHS5_CANLF tRNA (guanine-N(7)-)-methyltransferase non-catalytic subunit WDR4 OS=Canis lupus familiaris OX=9615 GN=WDR4 PE=3 SV=1	0.50	0.36	112.10	11547415.2	8	1	0	1.72	406	22738
>tr|F1PHG5|F1PHG5_CANLF RING-type E3 ubiquitin transferase OS=Canis lupus familiaris OX=9615 GN=RNF138 PE=4 SV=3	0.10	0.02	145.20	11526376.8	2	1	0	2.43	411	14298
>tr|E2R6E0|E2R6E0_CANLF Lipocln_cytosolic_FA-bd_dom domain-containing protein OS=Canis lupus familiaris OX=9615 GN=LCNL1 PE=3 SV=2	0.39	0.37	164.50	11428515.8	2	1	0	3.01	299	1932
>tr|E2R824|E2R824_CANLF Zinc finger protein 518B OS=Canis lupus familiaris OX=9615 GN=ZNF518B PE=4 SV=3	0.13	0.11	121.00	11390595.8	2	1	0	0.80	1000	24468
>tr|J9NS29|J9NS29_CANLF Cystatin domain-containing protein OS=Canis lupus familiaris OX=9615 GN=LOC607874 PE=4 SV=2	1.39	1.37	145.10	11309808.6	2	1	0	6.39	313	30016
>tr|F1PZ46|F1PZ46_CANLF Tudor domain containing 12 OS=Canis lupus familiaris OX=9615 GN=TDRD12 PE=4 SV=3	0.10	0.00	159.60	11262139.3	2	1	0	0.23	1320	3323
>tr|J9NRV0|J9NRV0_CANLF Histone H4 OS=Canis lupus familiaris OX=9615 GN=H4C11 PE=3 SV=2	0.19	0.17	150.10	11236927.7	2	1	1	21.78	101	7932
>tr|J9P0B4|J9P0B4_CANLF Tudor domain containing 15 OS=Canis lupus familiaris OX=9615 GN=TDRD15 PE=4 SV=2	1.48	0.89	105.10	11191609.5	3	2	0	1.85	2105	4188
>tr|F1Q0U7|F1Q0U7_CANLF ENAH actin regulator OS=Canis lupus familiaris OX=9615 GN=ENAH PE=3 SV=3	0.13	0.13	161.30	11109991.5	1	1	0	1.60	810	976

>tr|E2R6E0|E2R6E0_CANLF Lipocln_cytosolic_FA-bd_dom domain-containing protein OS=Canis lupus familiaris OX=9615 GN=LCNL1 PE=3 SV=2	2.02	1.98	238.10	11038584.9	3	1	0	3.68	299	1932
>sp|B8K1W2|ABCBB_CANLF Bile salt export pump OS=Canis lupus familiaris OX=9615 GN=Abcb11e PE=1 SV=1	0.85	0.81	211.00	11024350.0	3	1	0	0.30	1325	527
>tr|A0A5F4DFY1|A0A5F4DFY1_CANLF SHH signaling and ciliogenesis regulator SDCCAG8 OS=Canis lupus familiaris OX=9615 GN=SDCCAG8 PE=4 SV=1	0.10	0.01	39.30	10965869.4	11	1	1	0.59	673	17765
>tr|F1Q075|F1Q075_CANLF Nuclear receptor subfamily 1 group I member 2 OS=Canis lupus familiaris OX=9615 GN=NR1I2 PE=3 SV=3	0.28	0.26	109.70	10900461.8	2	1	1	2.12	471	44187
>tr|A0A5F4BZW4|A0A5F4BZW4_CANLF Malonyl-CoA decarboxylase OS=Canis lupus familiaris OX=9615 GN=MLYCD PE=4 SV=1	0.44	0.42	166.50	10873514.6	2	1	0	1.30	461	4809
>tr|E2RBL7|E2RBL7_CANLF DNA polymerase OS=Canis lupus familiaris OX=9615 GN=POLL PE=3 SV=2	0.32	0.30	143.60	10826999.5	2	1	0	1.60	625	22831
>tr|A0A5F4C3H5|A0A5F4C3H5_CANLF Purine rich element binding protein G OS=Canis lupus familiaris OX=9615 GN=PURG PE=3 SV=1	0.29	0.27	129.30	10817997.1	2	1	0	2.82	319	1070
>tr|E2RK34|E2RK34_CANLF Cytochrome P450 family 2 subfamily S member 1 OS=Canis lupus familiaris OX=9615 GN=CYP2S1 PE=3 SV=3	0.43	0.43	107.30	10797590.7	1	1	0	1.35	669	7130
>tr|A0A5F4C951|A0A5F4C951_CANLF F-actin monooxygenase OS=Canis lupus familiaris OX=9615 GN=MICAL3 PE=3 SV=1	0.23	0.23	102.80	10785253.8	1	1	1	0.38	2113	4569
>tr|F1PKS5|F1PKS5_CANLF IRF tryptophan pentad repeat domain-containing protein OS=Canis lupus familiaris OX=9615 GN=LOC609817 PE=4 SV=3	0.14	0.12	76.90	10753371.3	2	1	1	3.17	441	4897
>tr|A0A5F4CZE8|A0A5F4CZE8_CANLF FAD dependent oxidoreductase domain containing 2 OS=Canis lupus familiaris OX=9615 GN=FOXRED2 PE=4 SV=1	0.19	0.17	34.40	10730748.4	2	1	1	4.84	723	4094
>tr|J9P3D0|J9P3D0_CANLF Solute carrier family 4 member 9 OS=Canis lupus familiaris OX=9615 GN=SLC4A9 PE=3 SV=2	0.36	0.35	114.20	10719768.4	2	1	0	1.12	893	31921
>tr|A0A5F4DCA4|A0A5F4DCA4_CANLF Reverse transcriptase domain-containing protein OS=Canis lupus familiaris OX=9615 PE=4 SV=1	0.71	0.54	137.40	10686770.4	3	2	0	0.61	978	860
>sp|Q6AW47|EST5A_CANLF Carboxylesterase 5A OS=Canis lupus familiaris OX=9615 GN=CES5A PE=2 SV=1	0.52	0.52	190.20	10672246.8	1	1	0	0.87	575	629
>tr|A0A5F4C9V7|A0A5F4C9V7_CANLF Roundabout guidance receptor 3 OS=Canis lupus familiaris OX=9615 GN=ROBO3 PE=4 SV=1	0.22	0.22	184.30	10638326.1	1	1	0	0.42	1421	3563
>tr|E2QY55|E2QY55_CANLF Isocitrate dehydrogenase [NAD] subunit, mitochondrial OS=Canis lupus familiaris OX=9615 GN=IDH3G PE=3 SV=3	0.15	0.15	134.60	10546175.6	1	1	0	2.30	392	21772
>tr|J9NSS6|J9NSS6_CANLF DNA helicase OS=Canis lupus familiaris OX=9615 GN=CHD2 PE=4 SV=2	0.15	0.12	29.60	10468514.3	3	1	0	0.28	1780	1264
>tr|A0A5F4CPU3|A0A5F4CPU3_CANLF SEC24 homolog D, COPII coat complex component OS=Canis lupus familiaris OX=9615 GN=SEC24D PE=3 SV=1	0.10	0.02	17.10	10412460.3	1	1	0	0.53	946	2125
>tr|F1PSM3|F1PSM3_CANLF Symplekin OS=Canis lupus familiaris OX=9615 GN=SYMPK PE=4 SV=2	0.10	0.02	17.10	10412460.3	1	1	0	0.39	1275	9214
>tr|A0A5F4C2X8|A0A5F4C2X8_CANLF Fibrosin like 1 OS=Canis lupus familiaris OX=9615 GN=FBRSL1 PE=4 SV=1	0.10	0.00	149.50	10397764.3	2	1	0	0.71	985	1104
>tr|F1P8E3|F1P8E3_CANLF Glutamate metabotropic receptor 6 OS=Canis lupus familiaris OX=9615 GN=GRM6 PE=3 SV=3	0.16	0.16	130.20	10389980.0	1	1	0	0.68	876	36035
>tr|F1Q4I7|F1Q4I7_CANLF Beta-1,4-N-acetylgalactosaminyltransferase OS=Canis lupus familiaris OX=9615 GN=B4GALNT3 PE=3 SV=3	0.38	0.38	183.50	10351897.6	1	1	0	0.40	1003	6004

>sp|O46669|SCNAA_CANLF Sodium channel protein type 10 subunit alpha OS=Canis lupus familiaris OX=9615 GN=SCN10A PE=2 SV=1	0.35	0.36	111.50	10351897.6	1	1	0	0.15	1962	90
>tr|E2RCT1|E2RCT1_CANLF WAP domain-containing protein OS=Canis lupus familiaris OX=9615 PE=4 SV=2	1.69	1.38	135.60	10298958.6	2	2	0	23.28	116	21717
>tr|E2RCT1|E2RCT1_CANLF WAP domain-containing protein OS=Canis lupus familiaris OX=9615 PE=4 SV=2	0.73	0.69	208.70	10288892.1	3	1	0	14.66	116	21717
>tr|J9P801|J9P801_CANLF Jade family PHD finger 2 OS=Canis lupus familiaris OX=9615 GN=JADE2 PE=4 SV=1	0.24	0.24	50.10	10235919.7	2	1	1	0.64	781	4296
>tr|E2R6E0|E2R6E0_CANLF Lipocln_cytosolic_FA-bd_dom domain-containing protein OS=Canis lupus familiaris OX=9615 GN=LCNL1 PE=3 SV=2	2.41	2.35	243.70	10222630.6	4	1	0	3.68	299	1932
>tr|J9P2T7|J9P2T7_CANLF 26S proteasome non-ATPase regulatory subunit 5 OS=Canis lupus familiaris OX=9615 PE=4 SV=1	0.21	0.19	111.70	10219823.1	2	1	0	1.95	461	23870
>tr|A0A5F4CSE7|A0A5F4CSE7_CANLF TYR_PHOSPHATASE_2 domain-containing protein OS=Canis lupus familiaris OX=9615 GN=PTP4A1 PE=4 SV=1	0.10	0.01	57.80	10216523.6	1	1	1	5.20	173	25182
>tr|E2RRP1|E2RRP1_CANLF Butyryl-CoA dehydrogenase OS=Canis lupus familiaris OX=9615 GN=IVD PE=3 SV=2	0.10	0.06	87.50	10207941.6	2	1	0	3.99	426	10739
>tr|A0A5F4CLU1|A0A5F4CLU1_CANLF Superoxide dismutase [Cu-Zn] OS=Canis lupus familiaris OX=9615 GN=SOD1 PE=3 SV=1	0.10	0.06	174.10	10138293.1	2	1	0	7.09	141	25417
>tr|A0A5F4D9Z7|A0A5F4D9Z7_CANLF Zinc finger FYVE-type containing 19 OS=Canis lupus familiaris OX=9615 GN=ZFYVE19 PE=4 SV=1	0.58	0.58	127.50	10137454.4	1	1	0	1.53	392	13875
>tr|A0A5F4D7Y5|A0A5F4D7Y5_CANLF Pleckstrin homology, MyTH4 and FERM domain containing H1 OS=Canis lupus familiaris OX=9615 GN=PLEKHH1 PE=4 SV=1	0.34	0.33	194.40	10122263.9	2	1	0	0.30	1342	5979
>sp|P83509|RHG35_CANLF Rho GTPase-activating protein 35 OS=Canis lupus familiaris OX=9615 GN=ARHGAP35 PE=2 SV=1	0.42	0.40	98.10	10086811.8	2	1	1	0.73	1500	323
>tr|F1P7G9|F1P7G9_CANLF Endoribonuclease OS=Canis lupus familiaris OX=9615 GN=ERN1 PE=4 SV=3	0.32	0.32	99.50	10005410.1	1	1	0	1.74	975	20005
>tr|A0A5F4CAH2|A0A5F4CAH2_CANLF RNA polymerase II subunit A C-terminal domain phosphatase OS=Canis lupus familiaris OX=9615 GN=CTDP1 PE=4 SV=1	0.30	0.30	102.70	10002869.5	2	1	0	0.74	945	14396
>tr|A0A5F4CQ96|A0A5F4CQ96_CANLF ATP binding cassette subfamily G member 8 OS=Canis lupus familiaris OX=9615 GN=ABCG8 PE=3 SV=1	0.19	0.17	185.90	9998253.4	2	1	0	1.84	707	17201
>tr|A0A5F4D6L9|A0A5F4D6L9_CANLF Sacsin molecular chaperone OS=Canis lupus familiaris OX=9615 GN=SACS PE=4 SV=1	0.10	0.00	16.40	9968752.9	6	2	0	0.16	4500	1444
>tr|F1PLU0|F1PLU0_CANLF Histone-lysine N-methyltransferase OS=Canis lupus familiaris OX=9615 GN=KMT2A PE=3 SV=3	0.13	0.12	95.30	9933961.8	2	1	1	0.34	3822	11664
>tr|J9NS28|J9NS28_CANLF RBR-type E3 ubiquitin transferase OS=Canis lupus familiaris OX=9615 GN=ANKIB1 PE=4 SV=2	0.45	0.43	26.50	9933373.8	2	1	0	2.33	988	26345
>tr|E2R7J0|E2R7J0_CANLF FRA10A associated CGG repeat 1 OS=Canis lupus familiaris OX=9615 GN=FRA10AC1 PE=4 SV=2	0.13	0.13	117.30	9912961.9	1	1	0	2.24	313	9020
>tr|A0A5F4D963|A0A5F4D963_CANLF Microtubule-associated protein OS=Canis lupus familiaris OX=9615 GN=MAP4 PE=4 SV=1	0.10	0.03	150.30	9839670.6	2	1	0	0.23	2214	10459
>tr|E2RG75|E2RG75_CANLF Inactive ribonuclease-like protein 9 OS=Canis lupus familiaris OX=9615 GN=RNASE9 PE=3 SV=2	2.11	2.05	300.50	9687786.3	4	1	1	4.55	198	41734
>tr|A0A5F4D6G2|A0A5F4D6G2_CANLF SMG7 nonsense mediated mRNA decay factor OS=Canis lupus familiaris OX=9615 GN=SMG7 PE=4 SV=1	0.14	0.14	228.60	9672062.0	1	1	0	0.43	1175	2075

>tr|A0A5F4DI92|A0A5F4DI92_CANLF DNA helicase OS=Canis lupus familiaris OX=9615 GN=CHD6 PE=3 SV=1	0.16	0.14	152.60	9635686.3	2	1	0	0.26	2685	3470
>sp|Q28895|NPC2_CANLF NPC intracellular cholesterol transporter 2 OS=Canis lupus familiaris OX=9615 GN=NPC2 PE=2 SV=1	2.30	1.36	230.90	9613375.0	4	2	0	14.77	149	153
>sp|O46669|SCNAA_CANLF Sodium channel protein type 10 subunit alpha OS=Canis lupus familiaris OX=9615 GN=SCN10A PE=2 SV=1	0.84	0.56	143.90	9602299.5	2	2	0	0.31	1962	90
>tr|J9JHJ0|J9JHJ0_CANLF Testis expressed 50 OS=Canis lupus familiaris OX=9615 GN=TEX50 PE=4 SV=2	0.12	0.10	162.50	9541580.5	2	1	1	7.34	177	3785
>tr|F1Q2M4|F1Q2M4_CANLF DNA topoisomerase 2 OS=Canis lupus familiaris OX=9615 GN=TOP2A PE=3 SV=3	0.35	0.32	158.80	9534871.7	2	2	0	0.52	1532	1808
>tr|F1Q0P9|F1Q0P9_CANLF AT-hook containing transcription factor 1 OS=Canis lupus familiaris OX=9615 GN=AHCTF1 PE=4 SV=3	0.78	0.76	218.80	9531480.1	2	1	0	0.39	2321	1521
>tr|A0A5F4BNS6|A0A5F4BNS6_CANLF Transcription activation suppressor family member 2 OS=Canis lupus familiaris OX=9615 GN=TASOR2 PE=3 SV=1	0.12	0.11	24.70	9503267.6	2	1	0	0.70	2724	1014
>sp|Q9GL25|ESPB1_CANLF Epididymal sperm-binding protein 1 OS=Canis lupus familiaris OX=9615 GN=ELSPBP1 PE=1 SV=1	1.22	1.22	194.20	9429510.7	1	1	0	6.94	245	36
>tr|E2RSE8|E2RSE8_CANLF Vacuolar protein sorting-associated protein 11 homolog OS=Canis lupus familiaris OX=9615 GN=VPS11 PE=3 SV=1	0.46	0.44	164.10	9428095.3	2	1	0	0.85	941	17796
>tr|A0A5F4CHN1|A0A5F4CHN1_CANLF Serine/arginine repetitive matrix 2 OS=Canis lupus familiaris OX=9615 GN=SRRM2 PE=4 SV=1	0.30	0.28	147.90	9418999.7	2	1	0	0.12	2564	3180
>sp|P21842|CMA1_CANLF Chymase OS=Canis lupus familiaris OX=9615 GN=CMA1 PE=1 SV=1	0.19	0.17	104.40	9168112.1	2	1	0	0.80	249	34
>sp|Q9XS65|PTGDS_CANLF Prostaglandin-H2 D-isomerase OS=Canis lupus familiaris OX=9615 GN=PTGDS PE=2 SV=1	0.30	0.28	174.80	9131226.7	2	1	0	3.14	191	165
>tr|A0A5F4CGE0|A0A5F4CGE0_CANLF Ubiquitin protein ligase E3C OS=Canis lupus familiaris OX=9615 GN=UBE3C PE=4 SV=1	0.36	0.36	137.20	9093807.6	1	1	0	0.97	1238	9018
>tr|A0A5F4BX72|A0A5F4BX72_CANLF UV-stimulated scaffold protein A OS=Canis lupus familiaris OX=9615 GN=UVSSA PE=3 SV=1	0.10	0.06	16.80	9093807.6	1	1	0	3.09	679	16191
>tr|A0A5F4CWD8|A0A5F4CWD8_CANLF Replication factor C subunit 4 OS=Canis lupus familiaris OX=9615 GN=RFC4 PE=3 SV=1	0.33	0.33	130.30	9078300.7	1	1	0	2.68	336	34365
>tr|E2RRP3|E2RRP3_CANLF LIM homeobox 5 OS=Canis lupus familiaris OX=9615 GN=LHX5 PE=4 SV=1	0.12	0.12	136.30	9045969.1	1	1	0	2.24	402	19607
>tr|F1PQ34|F1PQ34_CANLF RCC1 and BTB domain containing protein 2 OS=Canis lupus familiaris OX=9615 GN=RCBTB2 PE=4 SV=3	0.10	0.02	63.70	9027790.8	1	1	0	1.23	326	6990
>tr|J9NSK0|J9NSK0_CANLF Basic helix-loop-helix family member b9 OS=Canis lupus familiaris OX=9615 GN=BHLHB9 PE=3 SV=1	0.45	0.45	73.10	9024477.8	1	1	0	1.09	550	35937
>tr|E2QWH7|E2QWH7_CANLF Coiled-coil domain containing 127 OS=Canis lupus familiaris OX=9615 GN=CCDC127 PE=4 SV=3	0.36	0.36	7.90	8996840.8	1	1	0	3.34	479	28521
>tr|J9JH98|J9JH98_CANLF Olfactory receptor family 1 subfamily L member 6 OS=Canis lupus familiaris OX=9615 GN=OR1L6 PE=4 SV=1	0.11	0.11	82.60	8909188.5	1	1	1	6.77	310	3096
>tr|E2R269|E2R269_CANLF Ubiquitinyl hydrolase 1 OS=Canis lupus familiaris OX=9615 GN=VCPIP1 PE=4 SV=1	0.99	0.95	87.70	8805102.3	3	1	0	1.39	1220	40849
>tr|F1PTW3|F1PTW3_CANLF KIAA0753 OS=Canis lupus familiaris OX=9615 GN=KIAA0753 PE=4 SV=3	0.41	0.37	155.90	8765844.2	3	1	0	0.88	905	33899

>tr|J9P3H8|J9P3H8_CANLF ATM interactor OS=Canis lupus familiaris OX=9615 GN=ATMIN PE=4 SV=2	0.10	0.02	58.30	8764056.9	9	1	0	0.58	863	882
>tr|E2R4A0|E2R4A0_CANLF Actin-like protein 7B OS=Canis lupus familiaris OX=9615 GN=ACTL7B PE=3 SV=2	0.25	0.23	122.50	8745609.6	2	1	1	2.42	454	3066
>tr|Q9XSV4|Q9XSV4_CANLF CE10 protein OS=Canis lupus familiaris OX=9615 GN=ce10 PE=2 SV=1	4.79	2.30	363.30	8673546.3	75	2	0	9.09	110	41542
>tr|E2RDT3|E2RDT3_CANLF Protein Mdm4 OS=Canis lupus familiaris OX=9615 GN=MDM4 PE=3 SV=3	0.10	0.03	61.10	8668513.4	1	1	0	4.48	491	11032
>sp|Q8WN22|PRKDC_CANLF DNA-dependent protein kinase catalytic subunit OS=Canis lupus familiaris OX=9615 GN=PRKDC PE=2 SV=1	1.85	1.39	101.60	8643845.6	3	2	0	0.12	4144	338
>tr|A0A5F4CPE1|A0A5F4CPE1_CANLF Major facilitator superfamily domain containing 14B OS=Canis lupus familiaris OX=9615 GN=MFSD14B PE=4 SV=1	0.38	0.38	38.30	8631560.7	1	1	0	3.71	485	28321
>tr|A0A5F4D3Q2|A0A5F4D3Q2_CANLF Non-specific serine/threonine protein kinase OS=Canis lupus familiaris OX=9615 GN=ATR PE=3 SV=1	0.47	0.45	151.30	8534283.6	3	1	0	0.35	2583	7077
>tr|E2RQR2|E2RQR2_CANLF Olfactory receptor family 13 subfamily P member 3 OS=Canis lupus familiaris OX=9615 GN=OR13P3 PE=4 SV=2	0.30	0.30	54.50	8458140.2	1	1	0	6.73	312	15310
>sp|B8K1W2|ABCBB_CANLF Bile salt export pump OS=Canis lupus familiaris OX=9615 GN=Abcb11e PE=1 SV=1	0.58	0.54	238.80	8447881.1	3	1	0	0.30	1325	527
>tr|A0A5F4D824|A0A5F4D824_CANLF Zinc finger RANBP2-type containing 3 OS=Canis lupus familiaris OX=9615 GN=ZRANB3 PE=4 SV=1	0.37	0.37	108.80	8389955.8	1	1	0	0.53	1128	1130
>tr|E2RN16|E2RN16_CANLF Mitogen-activated protein kinase kinase kinase 2 OS=Canis lupus familiaris OX=9615 GN=MAP3K2 PE=4 SV=2	0.10	0.01	157.80	8039775.1	1	1	0	0.97	620	34325
>tr|F6XW36|F6XW36_CANLF SH2 domain containing 4B OS=Canis lupus familiaris OX=9615 GN=SH2D4B PE=4 SV=1	0.35	0.35	87.70	8005068.1	1	1	0	2.31	433	13011
>tr|A0A5F4D961|A0A5F4D961_CANLF GLIPR1 like 2 OS=Canis lupus familiaris OX=9615 GN=GLIPR1L2 PE=4 SV=1	0.10	0.00	49.60	7958313.5	10	1	0	2.40	167	10571
>tr|A0A5K1V0D8|A0A5K1V0D8_CANLF Sulfatase 2 OS=Canis lupus familiaris OX=9615 GN=SULF2 PE=3 SV=1	0.82	0.80	179.30	7953313.6	2	1	0	0.35	859	1192
>tr|E2RTL2|E2RTL2_CANLF Tubulin tyrosine ligase like 6 OS=Canis lupus familiaris OX=9615 GN=TTLL6 PE=4 SV=3	0.10	0.07	37.30	7928933.2	1	1	0	0.60	827	2703
>tr|Q9XSV4|Q9XSV4_CANLF CE10 protein OS=Canis lupus familiaris OX=9615 GN=ce10 PE=2 SV=1	1.04	0.96	286.80	7912428.8	5	1	0	8.18	110	41542
>tr|Q2A652|Q2A652_CANLF G-protein coupled receptor OS=Canis lupus familiaris OX=9615 GN=PTGDR2 PE=2 SV=1	0.83	0.83	131.50	7904084.5	1	1	0	1.25	400	41469
>tr|J9P5T2|J9P5T2_CANLF Non-specific serine/threonine protein kinase OS=Canis lupus familiaris OX=9615 GN=WNK3 PE=4 SV=2	0.10	0.00	65.70	7615324.0	6	1	0	0.22	2294	5229
>tr|A0A5F4BT89|A0A5F4BT89_CANLF Olfactory receptor OS=Canis lupus familiaris OX=9615 GN=OR5W6 PE=3 SV=1	0.64	0.64	86.50	7580025.7	1	1	1	6.95	302	29923
>tr|J9P2Y5|J9P2Y5_CANLF MAK16 homolog OS=Canis lupus familiaris OX=9615 GN=MAK16 PE=3 SV=2	0.55	0.55	189.80	7568316.9	2	2	1	1.34	599	17345
>tr|F1PH76|F1PH76_CANLF Olfactory receptor OS=Canis lupus familiaris OX=9615 GN=OR4H12 PE=3 SV=2	0.12	0.12	94.60	7559293.4	1	1	0	2.91	309	20031
>tr|E2R4F0|E2R4F0_CANLF Cadherin EGF LAG seven-pass G-type receptor 2 OS=Canis lupus familiaris OX=9615 GN=CELSR2 PE=3 SV=2	0.21	0.22	46.30	7548313.9	1	1	1	0.65	2919	12898

>tr|F1PTB2|F1PTB2_CANLF Interleukin 6 signal transducer OS=Canis lupus familiaris OX=9615 GN=IL6ST PE=3 SV=3	0.26	0.27	105.80	7534976.6	1	1	0	0.35	867	4860
>tr|A0A5F4CPU3|A0A5F4CPU3_CANLF SEC24 homolog D, COPII coat complex component OS=Canis lupus familiaris OX=9615 GN=SEC24D PE=3 SV=1	0.54	0.54	5.70	7527381.6	1	1	0	0.53	946	2125
>tr|F1PSM3|F1PSM3_CANLF Symplekin OS=Canis lupus familiaris OX=9615 GN=SYMPK PE=4 SV=2	0.54	0.54	5.70	7527381.6	1	1	0	0.39	1275	9214
>tr|E2R186|E2R186_CANLF Fibroblast growth factor receptor OS=Canis lupus familiaris OX=9615 GN=FGFR1 PE=3 SV=3	0.16	0.03	95.30	7524778.9	9	1	0	0.59	853	9797
>tr|A0A5F4CGN3|A0A5F4CGN3_CANLF Mitogen-activated protein kinase OS=Canis lupus familiaris OX=9615 GN=MAPK10 PE=3 SV=1	1.14	1.14	132.70	7406255.4	1	1	0	0.86	464	863
>tr|F1PEE8|F1PEE8_CANLF Ryanodine receptor 2 OS=Canis lupus familiaris OX=9615 GN=RYR2 PE=4 SV=3	0.16	0.17	95.00	7402159.8	1	1	0	0.22	4903	13641
>tr|E2RG96|E2RG96_CANLF Glutamate receptor OS=Canis lupus familiaris OX=9615 GN=GRIN2C PE=3 SV=3	0.33	0.33	44.70	7335816.2	1	1	0	1.90	1212	31148
>tr|A0A5F4CPY7|A0A5F4CPY7_CANLF von Willebrand factor A domain containing 5A OS=Canis lupus familiaris OX=9615 GN=VWA5A PE=4 SV=1	0.45	0.45	208.90	7332917.7	1	1	0	0.75	803	1309
>tr|F1PPP9|F1PPP9_CANLF Family with sequence similarity 135 member A OS=Canis lupus familiaris OX=9615 GN=FAM135A PE=3 SV=3	0.84	0.78	111.30	7288820.4	4	1	0	1.22	1399	6815
>sp|P38377|S61A1_CANLF Protein transport protein Sec61 subunit alpha isoform 1 OS=Canis lupus familiaris OX=9615 GN=SEC61A1 PE=1 SV=3	0.82	0.80	182.90	7286196.4	2	1	0	0.84	476	358
>tr|A0A5F4D2X6|A0A5F4D2X6_CANLF AF4/FMR2 family member 3 OS=Canis lupus familiaris OX=9615 GN=AFF3 PE=3 SV=1	1.11	1.11	242.40	7260522.1	1	1	0	0.41	1213	3298
>sp|B8K1W2|ABCBB_CANLF Bile salt export pump OS=Canis lupus familiaris OX=9615 GN=Abcb11e PE=1 SV=1	0.56	0.54	178.50	7250369.8	2	1	0	0.30	1325	527
>tr|A0A5F4C2N8|A0A5F4C2N8_CANLF Sperm protamine P1 OS=Canis lupus familiaris OX=9615 PE=3 SV=1	0.28	0.28	204.10	7228673.5	1	1	0	10.00	50	7114
>tr|E2QXS1|E2QXS1_CANLF Lipase A, lysosomal acid type OS=Canis lupus familiaris OX=9615 GN=LIPA PE=4 SV=3	0.51	0.51	70.20	7158421.9	1	1	1	3.60	500	2185
>tr|A0A5F4BPM5|A0A5F4BPM5_CANLF ILK associated serine/threonine phosphatase OS=Canis lupus familiaris OX=9615 GN=ILKAP PE=3 SV=1	0.76	0.74	209.90	7113276.4	2	1	1	3.06	359	7963
>tr|E2RG75|E2RG75_CANLF Inactive ribonuclease-like protein 9 OS=Canis lupus familiaris OX=9615 GN=RNASE9 PE=3 SV=2	2.36	1.63	372.30	7097027.3	4	2	1	4.55	198	41734
>tr|J9P7Y2|J9P7Y2_CANLF Angiotensin-converting enzyme OS=Canis lupus familiaris OX=9615 GN=ACE2 PE=3 SV=1	0.34	0.34	55.10	7049933.8	1	1	0	1.62	804	1041
>tr|F6V7I1|F6V7I1_CANLF Dynein axonemal heavy chain 2 OS=Canis lupus familiaris OX=9615 GN=DNAH2 PE=3 SV=2	0.41	0.41	230.90	7039118.5	1	1	0	0.14	4378	7034
>tr|E2RHB9|E2RHB9_CANLF Squalene monooxygenase OS=Canis lupus familiaris OX=9615 GN=SQLE PE=3 SV=1	0.35	0.35	16.90	6939911.0	1	1	1	3.32	573	31440
>tr|A0A5F4CLU1|A0A5F4CLU1_CANLF Superoxide dismutase [Cu-Zn] OS=Canis lupus familiaris OX=9615 GN=SOD1 PE=3 SV=1	0.26	0.26	69.10	6925232.1	1	1	0	7.09	141	25417
>tr|E2QY94|E2QY94_CANLF Sphingomyelin synthase 2 OS=Canis lupus familiaris OX=9615 GN=SGMS2 PE=3 SV=1	0.17	0.17	60.00	6833113.2	1	1	1	4.93	365	18937
>tr|F1PR54|F1PR54_CANLF Lactotransferrin OS=Canis lupus familiaris OX=9615 GN=LTF PE=3 SV=1	4.28	1.90	329.20	6821944.9	33	4	0	7.49	708	40436

>tr|J9NZH4|J9NZH4_CANLF NTR domain-containing protein OS=Canis lupus familiaris OX=9615 GN=LOC102154527 PE=3 SV=2	1.27	1.27	96.30	6813856.5	1	1	0	8.60	221	39505
>tr|J9P9F3|J9P9F3_CANLF Protein tyrosine phosphatase domain containing 1 OS=Canis lupus familiaris OX=9615 GN=PTPDC1 PE=4 SV=1	0.50	0.50	145.40	6775985.8	1	1	0	1.47	748	24460
>tr|A0A5F4CAN7|A0A5F4CAN7_CANLF RBR-type E3 ubiquitin transferase OS=Canis lupus familiaris OX=9615 GN=RNF144A PE=4 SV=1	0.10	0.07	59.20	6725146.3	1	1	1	2.31	347	23811
>tr|F1PFZ5|F1PFZ5_CANLF Milk fat globule EGF and factor V/VIII domain containing OS=Canis lupus familiaris OX=9615 GN=MFGE8 PE=4 SV=3	0.25	0.25	147.50	6719153.7	1	1	0	1.87	428	7079
>tr|A0A5F4D7Y5|A0A5F4D7Y5_CANLF Pleckstrin homology, MyTH4 and FERM domain containing H1 OS=Canis lupus familiaris OX=9615 GN=PLEKHH1 PE=4 SV=1	0.16	0.16	203.30	6699243.3	1	1	0	0.30	1342	5979
>sp|Q9GKQ8|DSG1_CANLF Desmoglein-1 OS=Canis lupus familiaris OX=9615 GN=DSG1 PE=2 SV=1	0.41	0.41	201.70	6644458.8	1	1	0	0.47	1054	524
>tr|A0A5F4DI20|A0A5F4DI20_CANLF Aryl hydrocarbon receptor nuclear translocator OS=Canis lupus familiaris OX=9615 GN=ARNT PE=4 SV=1	0.10	0.03	169.30	6591554.4	1	1	0	0.40	753	1214
>tr|A0A5F4C1S8|A0A5F4C1S8_CANLF E3 ubiquitin-protein ligase CBL OS=Canis lupus familiaris OX=9615 GN=CBL PE=4 SV=1	0.19	0.17	144.90	6581470.8	2	1	0	0.39	773	1308
>tr|A0A5F4CMF9|A0A5F4CMF9_CANLF Cilia and flagella associated protein 65 OS=Canis lupus familiaris OX=9615 GN=CFAP65 PE=4 SV=1	0.36	0.36	139.30	6548062.7	1	1	0	0.17	1789	4175
>tr|F1P914|F1P914_CANLF Major vault protein OS=Canis lupus familiaris OX=9615 GN=MVP PE=4 SV=3	0.31	0.31	75.10	6542288.9	1	1	1	2.38	923	6261
>tr|A0A5F4D4Z3|A0A5F4D4Z3_CANLF Myosin IXA OS=Canis lupus familiaris OX=9615 GN=MYO9A PE=3 SV=1	0.90	0.91	175.60	6472391.0	1	1	0	0.31	2619	12945
>tr|F1PHA9|F1PHA9_CANLF Motile sperm domain containing 2 OS=Canis lupus familiaris OX=9615 GN=MOSPD2 PE=4 SV=3	0.13	0.14	123.60	6466169.2	1	1	0	0.97	518	10201
>sp|O18840|ACTB_CANLF Actin, cytoplasmic 1 OS=Canis lupus familiaris OX=9615 GN=ACTB PE=2 SV=3	2.78	1.43	346.50	6383688.9	12	2	0	7.73	375	642
>tr|A0A5F4DIZ9|A0A5F4DIZ9_CANLF Dynein axonemal heavy chain 3 OS=Canis lupus familiaris OX=9615 GN=DNAH3 PE=3 SV=1	0.19	0.17	136.30	6342296.9	3	2	1	0.37	3229	26729
>tr|J9NW72|J9NW72_CANLF Sperm associated antigen 8 OS=Canis lupus familiaris OX=9615 GN=SPAG8 PE=4 SV=1	0.58	0.58	91.70	6338436.5	1	1	0	4.07	442	10371
>tr|E2RDB0|E2RDB0_CANLF Phosphate regulating endopeptidase homolog X-linked OS=Canis lupus familiaris OX=9615 GN=PHEX PE=4 SV=2	0.10	0.07	135.90	6320761.7	1	1	1	1.34	749	2968
>tr|E2RRP3|E2RRP3_CANLF LIM homeobox 5 OS=Canis lupus familiaris OX=9615 GN=LHX5 PE=4 SV=1	0.10	0.00	154.40	6313101.1	1	1	0	2.24	402	19607
>tr|F1P6B8|F1P6B8_CANLF Intraflagellar transport protein 57 homolog OS=Canis lupus familiaris OX=9615 GN=IFT57 PE=3 SV=3	0.15	0.15	103.70	6262225.7	1	1	0	0.83	482	4757
>tr|A0A5F4BZS6|A0A5F4BZS6_CANLF Solute carrier family 41 member 3 OS=Canis lupus familiaris OX=9615 GN=SLC41A3 PE=3 SV=1	0.26	0.26	54.40	6216044.8	1	1	1	4.36	711	8952
>tr|A0A5F4CI07|A0A5F4CI07_CANLF RAB3 GTPase activating non-catalytic protein subunit 2 OS=Canis lupus familiaris OX=9615 GN=RAB3GAP2 PE=3 SV=1	0.13	0.13	113.70	6213974.0	1	1	0	0.79	1515	5216
>tr|F6V7I1|F6V7I1_CANLF Dynein axonemal heavy chain 2 OS=Canis lupus familiaris OX=9615 GN=DNAH2 PE=3 SV=2	0.44	0.44	193.50	6180024.1	1	1	0	0.14	4378	7034
>tr|J9NVV6|J9NVV6_CANLF Cytochrome P450 family 4 subfamily X member 1 OS=Canis lupus familiaris OX=9615 GN=CYP4X1 PE=3 SV=1	0.51	0.47	138.70	6162076.2	3	1	0	3.43	495	17759

>sp|P21842|CMA1_CANLF Chymase OS=Canis lupus familiaris OX=9615 GN=CMA1 PE=1 SV=1	0.64	0.60	88.20	6108504.4	3	1	0	0.80	249	34
>tr|A0A5F4C0S7|A0A5F4C0S7_CANLF HEAT repeat containing 5A OS=Canis lupus familiaris OX=9615 GN=HEATR5A PE=3 SV=1	0.22	0.12	108.60	6105671.2	6	1	0	0.25	1995	1753
>tr|E2RPK8|E2RPK8_CANLF Phosphatidylethanolamine binding protein 4 OS=Canis lupus familiaris OX=9615 GN=PEBP4 PE=3 SV=2	0.30	0.30	223.50	6067453.6	1	1	0	2.02	247	4725
>tr|A0A5F4DDT5|A0A5F4DDT5_CANLF Tousled like kinase 1 OS=Canis lupus familiaris OX=9615 GN=TLK1 PE=4 SV=1	0.36	0.36	162.40	6061049.9	1	1	0	0.68	734	7453
>tr|A0A5F4D020|A0A5F4D020_CANLF VWFA domain-containing protein OS=Canis lupus familiaris OX=9615 PE=3 SV=1	0.10	0.00	145.40	5985029.3	1	1	0	1.00	798	27451
>tr|F1P8J6|F1P8J6_CANLF RNA helicase OS=Canis lupus familiaris OX=9615 GN=DDX55 PE=3 SV=3	0.10	0.00	141.70	5985029.3	1	1	0	1.58	568	8934
>sp|Q9TUX8|NOS3_CANLF Nitric oxide synthase, endothelial OS=Canis lupus familiaris OX=9615 GN=NOS3 PE=2 SV=1	0.10	0.03	216.90	5952588.6	1	1	0	0.25	1205	117
>sp|P49822|ALBU_CANLF Albumin OS=Canis lupus familiaris OX=9615 GN=ALB PE=1 SV=3	0.35	0.35	182.50	5910507.1	1	1	0	1.64	608	490
>tr|A0A5F4D9L3|A0A5F4D9L3_CANLF Plexin C1 OS=Canis lupus familiaris OX=9615 GN=PLXNC1 PE=3 SV=1	0.10	0.00	172.80	5907173.8	1	1	0	0.19	1580	1260
>tr|A0A5F4CY61|A0A5F4CY61_CANLF 2-phospho-D-glycerate hydro-lyase OS=Canis lupus familiaris OX=9615 PE=3 SV=1	0.10	0.04	181.90	5887641.8	1	1	0	6.50	123	9061
>tr|A0A5F4CCD0|A0A5F4CCD0_CANLF Cysteine rich secretory protein 2 OS=Canis lupus familiaris OX=9615 GN=CRISP2 PE=3 SV=1	0.10	0.07	152.90	5872668.4	1	1	0	2.25	311	11017
>tr|A0A5F4CZ62|A0A5F4CZ62_CANLF Dynein cytoplasmic 1 heavy chain 1 OS=Canis lupus familiaris OX=9615 GN=DYNC1H1 PE=3 SV=1	0.10	0.03	206.60	5836740.3	1	1	0	0.07	4329	3271
>tr|A0A5F4CUI6|A0A5F4CUI6_CANLF NLR family pyrin domain containing 3 OS=Canis lupus familiaris OX=9615 GN=NLRP3 PE=3 SV=1	0.24	0.25	76.20	5814371.9	1	1	1	0.89	1007	9702
>tr|E2RIV7|E2RIV7_CANLF Syntrophin alpha 1 OS=Canis lupus familiaris OX=9615 GN=SNTA1 PE=3 SV=3	0.10	0.07	36.50	5810887.0	8	1	1	1.03	486	34454
>tr|E2R6E0|E2R6E0_CANLF Lipocln_cytosolic_FA-bd_dom domain-containing protein OS=Canis lupus familiaris OX=9615 GN=LCNL1 PE=3 SV=2	0.55	0.55	152.00	5795482.5	1	1	0	3.01	299	1932
>tr|E2RFH9|E2RFH9_CANLF RNA exonuclease 5 OS=Canis lupus familiaris OX=9615 GN=REXO5 PE=4 SV=3	0.71	0.71	95.30	5782692.1	1	1	1	2.17	782	3811
>tr|A0A5F4D7Y5|A0A5F4D7Y5_CANLF Pleckstrin homology, MyTH4 and FERM domain containing H1 OS=Canis lupus familiaris OX=9615 GN=PLEKHH1 PE=4 SV=1	0.52	0.53	191.20	5769906.8	1	1	0	0.30	1342	5979
>tr|F1PHA9|F1PHA9_CANLF Motile sperm domain containing 2 OS=Canis lupus familiaris OX=9615 GN=MOSPD2 PE=4 SV=3	0.36	0.36	212.00	5751740.5	1	1	0	0.97	518	10201
>tr|A0A5F4C604|A0A5F4C604_CANLF Signal recognition particle 14 kDa protein OS=Canis lupus familiaris OX=9615 GN=SRP14 PE=3 SV=1	0.29	0.29	137.10	5740770.1	1	1	0	5.50	109	19629
>tr|A0A5F4CWK7|A0A5F4CWK7_CANLF Ig-like domain-containing protein OS=Canis lupus familiaris OX=9615 PE=4 SV=1	2.46	2.42	346.40	5731538.2	3	1	1	17.00	100	33283
>tr|E2RPK8|E2RPK8_CANLF Phosphatidylethanolamine binding protein 4 OS=Canis lupus familiaris OX=9615 GN=PEBP4 PE=3 SV=2	0.10	0.00	164.10	5710200.2	1	1	0	2.02	247	4725
>sp|P21842|CMA1_CANLF Chymase OS=Canis lupus familiaris OX=9615 GN=CMA1 PE=1 SV=1	0.54	0.52	79.90	5638895.1	2	1	0	0.80	249	34

>tr|E2RG75|E2RG75_CANLF Inactive ribonuclease-like protein 9 OS=Canis lupus familiaris OX=9615 GN=RNASE9 PE=3 SV=2	2.93	1.97	361.40	5556511.4	2	2	1	4.55	198	41734
>tr|A0A5F4CQ96|A0A5F4CQ96_CANLF ATP binding cassette subfamily G member 8 OS=Canis lupus familiaris OX=9615 GN=ABCG8 PE=3 SV=1	0.10	0.03	163.80	5491250.2	1	1	0	1.84	707	17201
>tr|F1Q419|F1Q419_CANLF URB1 ribosome biogenesis homolog OS=Canis lupus familiaris OX=9615 GN=URB1 PE=4 SV=2	0.99	1.00	215.00	5484906.9	1	1	0	0.40	2277	6033
>tr|F1PLW8|F1PLW8_CANLF CUB and Sushi multiple domains 3 OS=Canis lupus familiaris OX=9615 GN=CSMD3 PE=4 SV=2	0.10	0.00	118.50	5474559.1	1	1	1	0.24	3707	17762
>tr|A0A5F4DHH0|A0A5F4DHH0_CANLF ATP binding cassette subfamily A member 1 OS=Canis lupus familiaris OX=9615 GN=ABCA1 PE=4 SV=1	0.48	0.46	73.20	5472409.6	2	1	0	0.23	2175	3709
>tr|A0A5F4DGF5|A0A5F4DGF5_CANLF Alkaline phosphatase OS=Canis lupus familiaris OX=9615 GN=ALPL PE=3 SV=1	1.54	1.54	195.00	5468783.8	1	1	0	2.45	572	6357
>tr|A0A5F4BXB8|A0A5F4BXB8_CANLF Vacuolar protein sorting 13 homolog A OS=Canis lupus familiaris OX=9615 GN=VPS13A PE=3 SV=1	0.12	0.13	85.20	5464980.8	1	1	1	0.38	2859	6548
>tr|E2RSI6|E2RSI6_CANLF Ezrin OS=Canis lupus familiaris OX=9615 GN=EZR PE=4 SV=1	0.10	0.00	152.80	5439995.0	1	1	0	1.54	586	15650
>tr|E2RN16|E2RN16_CANLF Mitogen-activated protein kinase kinase kinase 2 OS=Canis lupus familiaris OX=9615 GN=MAP3K2 PE=4 SV=2	0.28	0.28	139.30	5439652.6	1	1	0	0.97	620	34325
>tr|A0A5F4C3M5|A0A5F4C3M5_CANLF IQ motif containing GTPase activating protein 2 OS=Canis lupus familiaris OX=9615 GN=IQGAP2 PE=4 SV=1	0.53	0.53	36.10	5414616.0	1	1	0	0.38	1577	1056
>tr|A0A5F4BT89|A0A5F4BT89_CANLF Olfactory receptor OS=Canis lupus familiaris OX=9615 GN=OR5W6 PE=3 SV=1	0.10	0.00	163.90	5372311.7	1	1	1	6.95	302	29923
>sp|Q28895|NPC2_CANLF NPC intracellular cholesterol transporter 2 OS=Canis lupus familiaris OX=9615 GN=NPC2 PE=2 SV=1	10.08	5.44	477.30	5371042.8	36	3	0	30.20	149	153
>tr|E2RS98|E2RS98_CANLF Kinesin-like protein OS=Canis lupus familiaris OX=9615 GN=KIF18B PE=3 SV=3	0.10	0.03	42.30	5370355.7	1	1	0	0.70	856	29529
>tr|F1PB79|F1PB79_CANLF Abhydrolase domain containing 12B OS=Canis lupus familiaris OX=9615 GN=ABHD12B PE=4 SV=3	0.10	0.02	224.10	5354930.4	1	1	0	2.87	349	30573
>tr|A0A5F4DJM5|A0A5F4DJM5_CANLF Tyrosine-protein phosphatase non-receptor type OS=Canis lupus familiaris OX=9615 GN=PTPN21 PE=3 SV=1	0.10	0.03	151.90	5333927.0	1	1	0	0.55	1096	5460
>tr|A0A5F4D6G2|A0A5F4D6G2_CANLF SMG7 nonsense mediated mRNA decay factor OS=Canis lupus familiaris OX=9615 GN=SMG7 PE=4 SV=1	0.76	0.76	235.50	5319387.2	1	1	0	0.43	1175	2075
>tr|F1PRU0|F1PRU0_CANLF WD_REPEATS_REGION domain-containing protein OS=Canis lupus familiaris OX=9615 GN=TLE7 PE=3 SV=2	0.14	0.12	58.00	5298983.1	2	1	0	1.16	431	22849
>tr|F1P721|F1P721_CANLF Kinase suppressor of ras 2 OS=Canis lupus familiaris OX=9615 GN=KSR2 PE=4 SV=3	0.14	0.12	58.00	5298983.1	2	1	0	0.53	950	2276
>tr|F6PKZ1|F6PKZ1_CANLF Arylsulfatase A OS=Canis lupus familiaris OX=9615 GN=ARSA PE=3 SV=2	0.10	0.00	144.60	5287670.8	1	1	0	2.26	487	20206
>tr|F1PBU5|F1PBU5_CANLF Non-specific serine/threonine protein kinase OS=Canis lupus familiaris OX=9615 GN=SMG1 PE=3 SV=3	0.63	0.63	114.80	5257953.9	1	1	0	0.08	3634	6898
>tr|A0A5F4C2X8|A0A5F4C2X8_CANLF Fibrosin like 1 OS=Canis lupus familiaris OX=9615 GN=FBRSL1 PE=4 SV=1	0.10	0.00	142.50	5229332.9	1	1	0	0.71	985	1104
>tr|F1PD69|F1PD69_CANLF RING-type E3 ubiquitin transferase OS=Canis lupus familiaris OX=9615 GN=ZNRF3 PE=3 SV=2	0.10	0.03	214.50	5227219.4	1	1	0	1.21	827	33223
>sp|Q5I2M8|TLR9_CANLF Toll-like receptor 9 OS=Canis lupus familiaris OX=9615 GN=TLR9 PE=2 SV=1	0.43	0.43	140.20	5220431.7	1	1	0	1.16	1032	382

>tr|A0A5F4CCF5|A0A5F4CCF5_CANLF Interleukin 6 signal transducer OS=Canis lupus familiaris OX=9615 GN=IL6ST PE=4 SV=1	0.10	0.00	40.10	5178907.0	1	1	0	34.85	66	3968
>tr|F1PGK9|F1PGK9_CANLF ADAM metallopeptidase with thrombospondin type 1 motif 5 OS=Canis lupus familiaris OX=9615 GN=ADAMTS5 PE=4 SV=3	0.10	0.06	50.60	5178752.8	3	1	0	0.59	845	11956
>tr|E2R8N9|E2R8N9_CANLF WD repeat domain 87 OS=Canis lupus familiaris OX=9615 GN=WDR87 PE=4 SV=3	0.10	0.06	155.60	5147049.2	1	1	0	0.32	2806	14637
>tr|F1PS54|F1PS54_CANLF MFS domain-containing protein OS=Canis lupus familiaris OX=9615 GN=SLC17A3 PE=4 SV=3	0.75	0.75	97.00	5127813.2	1	1	1	7.71	493	988
>tr|A0A5F4DJV1|A0A5F4DJV1_CANLF ATP synthase subunit beta OS=Canis lupus familiaris OX=9615 GN=ATP5F1B PE=3 SV=1	0.10	0.00	106.50	5126973.0	1	1	0	2.42	619	6667
>tr|Q9XSV4|Q9XSV4_CANLF CE10 protein OS=Canis lupus familiaris OX=9615 GN=ce10 PE=2 SV=1	2.03	1.26	210.30	5124275.7	30	2	0	9.09	110	41542
>sp|P49822|ALBU_CANLF Albumin OS=Canis lupus familiaris OX=9615 GN=ALB PE=1 SV=3	0.10	0.00	145.50	5102735.0	1	1	0	1.64	608	490
>tr|A0A5F4CUD4|A0A5F4CUD4_CANLF Transcription initiation factor TFIID subunit OS=Canis lupus familiaris OX=9615 GN=TAF1 PE=3 SV=1	0.10	0.06	24.70	5096037.9	1	1	0	0.26	1897	4265
>tr|J9P870|J9P870_CANLF Insulin like growth factor binding protein 6 OS=Canis lupus familiaris OX=9615 GN=IGFBP6 PE=4 SV=2	0.16	0.16	98.30	5066264.0	1	1	0	4.29	303	29295
>tr|E2RT65|E2RT65_CANLF Phosphoglycerate mutase OS=Canis lupus familiaris OX=9615 GN=PI4K2A PE=3 SV=2	0.10	0.03	230.30	5052692.6	1	1	0	4.33	254	15155
>tr|F1PX00|F1PX00_CANLF Transmembrane and coiled-coil domain family 3 OS=Canis lupus familiaris OX=9615 GN=TMCC3 PE=3 SV=2	0.12	0.12	76.50	5051932.0	1	1	0	4.43	451	8778
>tr|E2R1V3|E2R1V3_CANLF U2 snRNP associated SURP domain containing OS=Canis lupus familiaris OX=9615 GN=U2SURP PE=4 SV=3	0.12	0.12	146.00	5019941.6	1	1	0	0.78	1029	12656
>tr|E2RE16|E2RE16_CANLF Non-specific serine/threonine protein kinase OS=Canis lupus familiaris OX=9615 GN=PAK4 PE=4 SV=1	0.16	0.02	48.50	5005744.2	22	1	0	0.84	592	12735
>tr|A0A5F4CW57|A0A5F4CW57_CANLF Alsin Rho guanine nucleotide exchange factor ALS2 OS=Canis lupus familiaris OX=9615 GN=ALS2 PE=4 SV=1	0.10	0.02	125.00	4998044.8	1	1	0	1.31	1523	1985
>tr|J9NTP3|J9NTP3_CANLF Protein tyrosine phosphatase mitochondrial 1 OS=Canis lupus familiaris OX=9615 GN=PTPMT1 PE=4 SV=1	0.35	0.35	132.30	4991333.5	1	1	0	1.82	275	3704
>tr|A0A5F4C0S7|A0A5F4C0S7_CANLF HEAT repeat containing 5A OS=Canis lupus familiaris OX=9615 GN=HEATR5A PE=3 SV=1	0.38	0.38	151.20	4982510.0	1	1	0	0.30	1995	1753
>tr|A0A5F4CQH1|A0A5F4CQH1_CANLF Na(+)/H(+) exchange regulatory cofactor NHE-RF OS=Canis lupus familiaris OX=9615 GN=SLC9A3R1 PE=4 SV=1	0.10	0.01	163.60	4945695.2	1	1	0	2.23	359	1876
>tr|A0A5F4CT13|A0A5F4CT13_CANLF Anion exchange protein OS=Canis lupus familiaris OX=9615 GN=SLC4A2 PE=3 SV=1	0.10	0.00	97.30	4850691.2	1	1	0	1.50	1263	4624
>tr|A0A5F4D3E3|A0A5F4D3E3_CANLF Poly [ADP-ribose] polymerase OS=Canis lupus familiaris OX=9615 GN=PARP2 PE=4 SV=1	0.10	0.03	56.30	4850691.2	1	1	0	2.16	602	5219
>tr|A0A5F4DJ83|A0A5F4DJ83_CANLF DExH-box helicase 57 OS=Canis lupus familiaris OX=9615 GN=DHX57 PE=4 SV=1	1.09	1.07	168.40	4849595.7	2	1	0	0.64	1411	5166
>tr|A0A5F4D9N8|A0A5F4D9N8_CANLF Procollagen-lysine,2-oxoglutarate 5-dioxygenase 3 OS=Canis lupus familiaris OX=9615 GN=PLOD3 PE=4 SV=1	0.16	0.16	53.30	4849194.1	1	1	0	2.17	785	1958
>tr|E2RIV7|E2RIV7_CANLF Syntrophin alpha 1 OS=Canis lupus familiaris OX=9615 GN=SNTA1 PE=3 SV=3	0.10	0.02	41.30	4832783.8	13	1	1	1.03	486	34454

>tr|A0A5F4CCD0|A0A5F4CCD0_CANLF Cysteine rich secretory protein 2 OS=Canis lupus familiaris OX=9615 GN=CRISP2 PE=3 SV=1	0.30	0.30	170.40	4780781.6	1	1	0	2.25	311	11017
>tr|A0A5F4CDD9|A0A5F4CDD9_CANLF Proline dehydrogenase OS=Canis lupus familiaris OX=9615 GN=PRODH2 PE=3 SV=1	0.10	0.01	183.80	4763870.7	1	1	1	1.69	415	14972
>sp|Q9XS65|PTGDS_CANLF Prostaglandin-H2 D-isomerase OS=Canis lupus familiaris OX=9615 GN=PTGDS PE=2 SV=1	0.42	0.42	169.80	4721122.2	1	1	0	3.14	191	165
>tr|A0A5F4BT89|A0A5F4BT89_CANLF Olfactory receptor OS=Canis lupus familiaris OX=9615 GN=OR5W6 PE=3 SV=1	0.10	0.02	175.30	4715258.3	1	1	1	6.95	302	29923
>tr|J9P434|J9P434_CANLF Myotubularin related protein 14 OS=Canis lupus familiaris OX=9615 GN=MTMR14 PE=4 SV=2	0.10	0.00	140.50	4695683.5	1	1	0	1.51	596	17413
>tr|A0A5F4D6G2|A0A5F4D6G2_CANLF SMG7 nonsense mediated mRNA decay factor OS=Canis lupus familiaris OX=9615 GN=SMG7 PE=4 SV=1	0.19	0.19	193.40	4676067.7	1	1	0	0.43	1175	2075
>tr|E2R6E0|E2R6E0_CANLF Lipocln_cytosolic_FA-bd_dom domain-containing protein OS=Canis lupus familiaris OX=9615 GN=LCNL1 PE=3 SV=2	0.89	0.89	125.80	4617242.5	1	1	0	3.01	299	1932
>tr|J9NSS6|J9NSS6_CANLF DNA helicase OS=Canis lupus familiaris OX=9615 GN=CHD2 PE=4 SV=2	0.10	0.08	70.00	4569948.7	6	1	0	0.28	1780	1264
>tr|J9NSS6|J9NSS6_CANLF DNA helicase OS=Canis lupus familiaris OX=9615 GN=CHD2 PE=4 SV=2	0.25	0.02	65.60	4531674.9	21	1	0	0.28	1780	1264
>sp|Q28279|CNGA1_CANLF cGMP-gated cation channel alpha-1 OS=Canis lupus familiaris OX=9615 GN=CNGA1 PE=2 SV=1	0.36	0.34	164.90	4503656.9	2	1	0	0.58	691	455
>tr|F1PJ71|F1PJ71_CANLF Glutathione peroxidase OS=Canis lupus familiaris OX=9615 GN=GPX5 PE=3 SV=2	0.63	0.57	215.90	4480646.2	4	1	1	6.33	221	19009
>tr|J9PAF1|J9PAF1_CANLF Actin binding LIM protein family member 2 OS=Canis lupus familiaris OX=9615 GN=ABLIM2 PE=4 SV=2	0.50	0.50	119.60	4474940.0	1	1	0	1.71	645	4331
>tr|A0A5F4D9S5|A0A5F4D9S5_CANLF Hyaluronoglucosaminidase OS=Canis lupus familiaris OX=9615 GN=CEMIP PE=3 SV=1	0.55	0.55	187.30	4458486.7	1	1	0	0.24	1684	9775
>tr|F1PI09|F1PI09_CANLF Aldehyde oxidase OS=Canis lupus familiaris OX=9615 GN=AOX2 PE=3 SV=3	0.41	0.41	174.50	4453249.7	1	1	0	0.67	1347	21650
>tr|J9P7Y2|J9P7Y2_CANLF Angiotensin-converting enzyme OS=Canis lupus familiaris OX=9615 GN=ACE2 PE=3 SV=1	0.80	0.80	53.60	4424277.3	1	1	0	1.62	804	1041
>tr|J9NRV0|J9NRV0_CANLF Histone H4 OS=Canis lupus familiaris OX=9615 GN=H4C11 PE=3 SV=2	0.14	0.14	123.30	4411893.1	1	1	1	21.78	101	7932
>tr|A0A5F4DCA4|A0A5F4DCA4_CANLF Reverse transcriptase domain-containing protein OS=Canis lupus familiaris OX=9615 PE=4 SV=1	0.28	0.28	149.70	4405126.8	1	1	0	0.31	978	860
>tr|A0A5F4CAT6|A0A5F4CAT6_CANLF Centromere protein I OS=Canis lupus familiaris OX=9615 GN=CENPI PE=3 SV=1	0.17	0.17	86.80	4393225.8	1	1	1	1.29	699	20654
>tr|F1PL17|F1PL17_CANLF TNF receptor superfamily member 13C OS=Canis lupus familiaris OX=9615 GN=TNFRSF13C PE=4 SV=3	0.55	0.55	93.70	4386567.6	1	1	0	3.15	286	13040
>tr|A0A5F4D7J3|A0A5F4D7J3_CANLF Non-specific serine/threonine protein kinase OS=Canis lupus familiaris OX=9615 GN=CDC42BPA PE=3 SV=1	0.80	0.80	183.60	4334414.5	1	1	0	0.22	1794	1069
>tr|F1PKW7|F1PKW7_CANLF Tyrosine 3-monooxygenase/tryptophan 5-monooxygenase activation protein beta OS=Canis lupus familiaris OX=9615 GN=YWHAB PE=3 SV=3	0.10	0.02	205.20	4326583.6	1	1	0	2.03	246	2992
>tr|F1PJA4|F1PJA4_CANLF Phospholipid-transporting ATPase OS=Canis lupus familiaris OX=9615 GN=ATP8B4 PE=3 SV=3	0.27	0.27	130.50	4266179.6	1	1	0	0.50	1191	2739

>sp|Q9GL25|ESPB1_CANLF Epididymal sperm-binding protein 1 OS=Canis lupus familiaris OX=9615 GN=ELSPBP1 PE=1 SV=1	1.76	1.74	182.30	4263954.6	2	1	0	4.49	245	36
>tr|F1PTL9|F1PTL9_CANLF ATP binding cassette subfamily C member 8 OS=Canis lupus familiaris OX=9615 GN=ABCC8 PE=3 SV=3	0.10	0.02	191.90	4114660.3	1	1	0	0.28	1454	4249
>tr|F1PLU6|F1PLU6_CANLF Collagen type IX alpha 3 chain OS=Canis lupus familiaris OX=9615 GN=COL9A3 PE=4 SV=3	0.43	0.43	13.00	4063872.4	1	1	0	6.01	416	18705
>tr|A0A5F4DCW5|A0A5F4DCW5_CANLF Fibrosin like 1 OS=Canis lupus familiaris OX=9615 GN=FBRSL1 PE=4 SV=1	0.53	0.52	43.80	3992072.6	2	2	2	2.30	566	32781
>tr|A0A5F4DGF5|A0A5F4DGF5_CANLF Alkaline phosphatase OS=Canis lupus familiaris OX=9615 GN=ALPL PE=3 SV=1	0.36	0.36	142.30	3954869.0	1	1	0	2.45	572	6357
>tr|A0A5F4C1S8|A0A5F4C1S8_CANLF E3 ubiquitin-protein ligase CBL OS=Canis lupus familiaris OX=9615 GN=CBL PE=4 SV=1	0.66	0.67	88.50	3945452.0	1	1	0	0.39	773	1308
>tr|A0A5F4BX58|A0A5F4BX58_CANLF Transcription termination factor 1 OS=Canis lupus familiaris OX=9615 GN=TTF1 PE=4 SV=1	0.76	0.74	214.70	3896328.8	2	1	0	0.59	677	12089
>tr|A0A5F4DL64|A0A5F4DL64_CANLF Tyrosine-protein kinase receptor OS=Canis lupus familiaris OX=9615 GN=ROS1 PE=3 SV=1	0.10	0.00	143.30	3787080.6	1	1	0	0.22	2272	14391
>tr|F1PJY1|F1PJY1_CANLF Mannosyl-glycoprotein endo-beta-N-acetylglucosaminidase OS=Canis lupus familiaris OX=9615 GN=ENGASE PE=3 SV=3	0.10	0.04	137.40	3758465.7	2	1	1	1.74	690	32761
>tr|A0A5F4CQ96|A0A5F4CQ96_CANLF ATP binding cassette subfamily G member 8 OS=Canis lupus familiaris OX=9615 GN=ABCG8 PE=3 SV=1	0.18	0.18	173.10	3723762.9	1	1	0	1.84	707	17201
>tr|A0A5F4CNP4|A0A5F4CNP4_CANLF ADP ribosylation factor GTPase activating protein 3 OS=Canis lupus familiaris OX=9615 GN=ARFGAP3 PE=4 SV=1	0.57	0.57	188.60	3707457.0	1	1	0	1.12	714	964
>tr|J9P432|J9P432_CANLF Glutamine--fructose-6-phosphate transaminase (isomerizing) OS=Canis lupus familiaris OX=9615 GN=GFPT1 PE=4 SV=2	0.46	0.44	89.40	3651746.4	2	1	0	1.18	677	7191
>tr|A0A5F4D9S5|A0A5F4D9S5_CANLF Hyaluronoglucosaminidase OS=Canis lupus familiaris OX=9615 GN=CEMIP PE=3 SV=1	0.97	0.97	187.80	3577467.9	1	1	0	0.24	1684	9775
>tr|A0A5F4DCA4|A0A5F4DCA4_CANLF Reverse transcriptase domain-containing protein OS=Canis lupus familiaris OX=9615 PE=4 SV=1	0.54	0.54	128.70	3560066.5	1	1	0	0.31	978	860
>sp|Q9XSU7|RL27_CANLF 60S ribosomal protein L27 OS=Canis lupus familiaris OX=9615 GN=RPL27 PE=2 SV=3	0.10	0.04	16.80	3548887.2	1	1	0	3.68	136	314
>tr|F1PJ71|F1PJ71_CANLF Glutathione peroxidase OS=Canis lupus familiaris OX=9615 GN=GPX5 PE=3 SV=2	0.43	0.42	167.10	3548106.6	3	1	1	6.33	221	19009
>tr|A0A5F4CK78|A0A5F4CK78_CANLF NIMA related kinase 9 OS=Canis lupus familiaris OX=9615 GN=NEK9 PE=3 SV=1	0.17	0.15	114.30	3538761.4	2	1	0	1.25	1040	34954
>tr|A0A5F4DKH0|A0A5F4DKH0_CANLF Multifunctional fusion protein OS=Canis lupus familiaris OX=9615 GN=PARP6 PE=3 SV=1	0.82	0.80	125.30	3513288.8	2	1	0	0.45	1112	1413
>tr|E2RHC5|E2RHC5_CANLF Baculoviral IAP repeat containing 7 OS=Canis lupus familiaris OX=9615 GN=BIRC7 PE=3 SV=3	0.10	0.06	139.80	3491016.8	2	1	0	2.95	271	10900
>tr|F1PGK9|F1PGK9_CANLF ADAM metallopeptidase with thrombospondin type 1 motif 5 OS=Canis lupus familiaris OX=9615 GN=ADAMTS5 PE=4 SV=3	0.10	0.02	41.00	3440320.3	9	1	0	0.59	845	11956
>tr|E2RTL2|E2RTL2_CANLF Tubulin tyrosine ligase like 6 OS=Canis lupus familiaris OX=9615 GN=TTLL6 PE=4 SV=3	0.17	0.17	50.00	3278214.6	1	1	0	0.60	827	2703
>tr|F1PIW3|F1PIW3_CANLF Dynein axonemal heavy chain 14 OS=Canis lupus familiaris OX=9615 GN=DNAH14 PE=3 SV=3	0.11	0.10	69.60	3274474.5	2	1	1	0.16	4491	30490

>tr|A0A5F4C289|A0A5F4C289_CANLF Netrin receptor UNC5 OS=Canis lupus familiaris OX=9615 GN=UNC5D PE=3 SV=1	0.14	0.14	43.70	3257952.1	1	1	1	1.80	945	14524
>tr|J9NUT9|J9NUT9_CANLF Pericentrin OS=Canis lupus familiaris OX=9615 GN=PCNT PE=4 SV=2	0.74	0.74	196.70	3227068.8	1	1	0	0.19	3208	18609
>tr|E2RA12|E2RA12_CANLF PH domain and leucine rich repeat protein phosphatase 2 OS=Canis lupus familiaris OX=9615 GN=PHLPP2 PE=4 SV=3	0.16	0.16	123.80	3110773.8	1	1	1	0.44	1351	11085
>tr|A0A5F4DBL7|A0A5F4DBL7_CANLF LDL receptor related protein 1 OS=Canis lupus familiaris OX=9615 GN=LRP1 PE=3 SV=1	0.42	0.42	32.60	3070857.8	1	1	0	0.22	4133	14011
>tr|E2RRF5|E2RRF5_CANLF RNA binding motif protein 19 OS=Canis lupus familiaris OX=9615 GN=RBM19 PE=4 SV=3	0.13	0.11	43.30	3068568.4	2	1	0	0.41	970	905
>tr|A0A5F4CEM0|A0A5F4CEM0_CANLF Proline rich coiled-coil 2B OS=Canis lupus familiaris OX=9615 GN=PRRC2B PE=4 SV=1	0.10	0.02	142.50	2893515.9	7	1	1	0.58	2235	37089
>tr|J9P432|J9P432_CANLF Glutamine--fructose-6-phosphate transaminase (isomerizing) OS=Canis lupus familiaris OX=9615 GN=GFPT1 PE=4 SV=2	0.71	0.71	67.80	2882998.9	1	1	0	1.18	677	7191
>tr|F1PBU5|F1PBU5_CANLF Non-specific serine/threonine protein kinase OS=Canis lupus familiaris OX=9615 GN=SMG1 PE=3 SV=3	0.23	0.23	171.60	2865115.3	1	1	0	0.08	3634	6898
>tr|A0A5F4CCY0|A0A5F4CCY0_CANLF Programmed cell death 11 OS=Canis lupus familiaris OX=9615 GN=PDCD11 PE=4 SV=1	0.60	0.50	65.10	2834526.4	7	1	0	0.16	1829	1406
>sp|Q9XS65|PTGDS_CANLF Prostaglandin-H2 D-isomerase OS=Canis lupus familiaris OX=9615 GN=PTGDS PE=2 SV=1	1.68	1.10	124.50	2819070.1	7	2	1	16.23	191	165
>tr|A0A5F4D7Y5|A0A5F4D7Y5_CANLF Pleckstrin homology, MyTH4 and FERM domain containing H1 OS=Canis lupus familiaris OX=9615 GN=PLEKHH1 PE=4 SV=1	0.10	0.06	128.30	2760122.0	1	1	0	0.30	1342	5979
>tr|F1PPP9|F1PPP9_CANLF Family with sequence similarity 135 member A OS=Canis lupus familiaris OX=9615 GN=FAM135A PE=3 SV=3	0.58	0.59	34.40	2655858.4	1	1	0	1.22	1399	6815
>tr|J9P3H8|J9P3H8_CANLF ATM interactor OS=Canis lupus familiaris OX=9615 GN=ATMIN PE=4 SV=2	0.16	0.02	49.40	2616877.9	12	1	0	0.58	863	882
>tr|A0A5F4D430|A0A5F4D430_CANLF Transcription factor AP-2 gamma OS=Canis lupus familiaris OX=9615 GN=TFAP2C PE=3 SV=1	0.60	0.61	162.00	2499781.8	1	1	0	1.38	650	9642
>tr|A0A5F4D3Q2|A0A5F4D3Q2_CANLF Non-specific serine/threonine protein kinase OS=Canis lupus familiaris OX=9615 GN=ATR PE=3 SV=1	0.10	0.03	112.80	2446716.1	1	1	0	0.35	2583	7077
>tr|E2R3M8|E2R3M8_CANLF Unc-93 homolog B1, TLR signaling regulator OS=Canis lupus familiaris OX=9615 GN=UNC93B1 PE=4 SV=2	1.02	1.02	217.70	2435177.9	1	1	0	1.08	369	5718
>tr|A0A5F4DHH0|A0A5F4DHH0_CANLF ATP binding cassette subfamily A member 1 OS=Canis lupus familiaris OX=9615 GN=ABCA1 PE=4 SV=1	0.10	0.02	39.00	2381312.6	7	1	0	0.23	2175	3709
>tr|A0A5F4CLU1|A0A5F4CLU1_CANLF Superoxide dismutase [Cu-Zn] OS=Canis lupus familiaris OX=9615 GN=SOD1 PE=3 SV=1	0.35	0.35	143.80	2338204.7	1	1	0	7.09	141	25417
>tr|A0A5F4C1D8|A0A5F4C1D8_CANLF Acrosin OS=Canis lupus familiaris OX=9615 GN=ACR PE=3 SV=1	0.44	0.44	175.10	2328870.8	2	1	0	2.84	423	43346
>tr|F1PI09|F1PI09_CANLF Aldehyde oxidase OS=Canis lupus familiaris OX=9615 GN=AOX2 PE=3 SV=3	0.41	0.37	147.40	2327470.8	3	1	0	0.67	1347	21650
>sp|Q28894|WFDC2_CANLF WAP four-disulfide core domain protein 2 OS=Canis lupus familiaris OX=9615 GN=WFDC2 PE=2 SV=1	1.79	1.77	348.70	2311834.0	2	1	0	6.45	124	53
>tr|A0A5F4CTR9|A0A5F4CTR9_CANLF Semaphorin 3A OS=Canis lupus familiaris OX=9615 GN=SEMA3A PE=3 SV=1	0.27	0.27	14.40	2298305.7	1	1	0	0.68	732	2791

>tr|F1PJ45|F1PJ45_CANLF Adhesion G protein-coupled receptor L3 OS=Canis lupus familiaris OX=9615 GN=ADGRL3 PE=4 SV=2	0.27	0.27	14.40	2298305.7	1	1	0	0.33	1528	2066
>tr|A0A5F4D8X3|A0A5F4D8X3_CANLF Solute carrier family 36 member 4 OS=Canis lupus familiaris OX=9615 GN=SLC36A4 PE=4 SV=1	0.58	0.58	17.70	2293612.5	1	1	0	3.70	459	15767
>tr|E2RG75|E2RG75_CANLF Inactive ribonuclease-like protein 9 OS=Canis lupus familiaris OX=9615 GN=RNASE9 PE=3 SV=2	0.84	0.82	353.00	2240387.0	2	1	1	4.55	198	41734
>tr|F1PPP9|F1PPP9_CANLF Family with sequence similarity 135 member A OS=Canis lupus familiaris OX=9615 GN=FAM135A PE=3 SV=3	0.72	0.70	99.10	2148188.2	2	1	0	1.22	1399	6815
>tr|A0A5F4C1D8|A0A5F4C1D8_CANLF Acrosin OS=Canis lupus familiaris OX=9615 GN=ACR PE=3 SV=1	0.45	0.43	254.50	2133176.8	2	1	0	2.84	423	43346
>sp|Q28279|CNGA1_CANLF cGMP-gated cation channel alpha-1 OS=Canis lupus familiaris OX=9615 GN=CNGA1 PE=2 SV=1	0.89	0.89	213.50	2084821.0	1	1	0	0.58	691	455
>tr|F1PR54|F1PR54_CANLF Lactotransferrin OS=Canis lupus familiaris OX=9615 GN=LTF PE=3 SV=1	3.02	1.60	295.20	2081618.8	18	3	0	5.37	708	40436
>sp|P25473|CLUS_CANLF Clusterin OS=Canis lupus familiaris OX=9615 GN=CLU PE=2 SV=1	0.98	0.98	240.00	2016485.8	1	1	0	0.90	445	725
>tr|A0A5F4DCA4|A0A5F4DCA4_CANLF Reverse transcriptase domain-containing protein OS=Canis lupus familiaris OX=9615 PE=4 SV=1	1.26	1.26	176.40	1998374.1	1	1	0	0.31	978	860
>tr|A0A5F4CF26|A0A5F4CF26_CANLF S1 RNA binding domain 1 OS=Canis lupus familiaris OX=9615 GN=SRBD1 PE=4 SV=1	0.10	0.00	40.00	1975157.3	2	1	0	0.50	1003	6216
>tr|A0A5F4CUE8|A0A5F4CUE8_CANLF Senataxin OS=Canis lupus familiaris OX=9615 GN=SETX PE=4 SV=1	0.11	0.11	133.20	1946556.2	1	1	0	0.26	2645	1796
>sp|Q9XT60|SRY_CANLF Sex-determining region Y protein OS=Canis lupus familiaris OX=9615 GN=SRY PE=3 SV=1	0.29	0.29	89.50	1904460.7	1	1	0	3.18	220	347
>tr|A0A5F4CMY6|A0A5F4CMY6_CANLF Rho-associated protein kinase 2 OS=Canis lupus familiaris OX=9615 GN=ROCK2 PE=3 SV=1	0.10	0.00	82.40	1903807.5	2	1	0	0.35	1145	1527
>tr|A0A5F4C7Q7|A0A5F4C7Q7_CANLF IQ motif and Sec7 domain ArfGEF 1 OS=Canis lupus familiaris OX=9615 GN=IQSEC1 PE=3 SV=1	0.11	0.12	45.50	1862384.0	1	1	0	0.51	971	4269
>tr|A0A5F4DHH0|A0A5F4DHH0_CANLF ATP binding cassette subfamily A member 1 OS=Canis lupus familiaris OX=9615 GN=ABCA1 PE=4 SV=1	0.27	0.25	34.80	1829194.8	2	1	0	0.23	2175	3709
>sp|Q863Z4|MTPN_CANLF Myotrophin OS=Canis lupus familiaris OX=9615 GN=MTPN PE=3 SV=3	0.43	0.43	70.80	1791867.0	2	1	0	4.24	118	229
>tr|F1PI09|F1PI09_CANLF Aldehyde oxidase OS=Canis lupus familiaris OX=9615 GN=AOX2 PE=3 SV=3	0.13	0.13	88.30	1775376.8	1	1	0	0.67	1347	21650
>tr|J9PA70|J9PA70_CANLF Tigger transposable element derived 7 OS=Canis lupus familiaris OX=9615 GN=TIGD7 PE=3 SV=2	0.67	0.67	101.10	1765648.6	1	1	0	1.46	548	5607
>tr|F1Q2B6|F1Q2B6_CANLF Coiled-coil domain containing 57 OS=Canis lupus familiaris OX=9615 GN=CCDC57 PE=4 SV=3	0.37	0.37	149.50	1719110.0	1	1	1	1.10	727	9706
>tr|F1PIJ6|F1PIJ6_CANLF Inorganic diphosphatase OS=Canis lupus familiaris OX=9615 GN=PPA1 PE=3 SV=3	0.10	0.00	12.70	1613397.0	3	1	1	1.77	283	2594
>sp|Q9XT60|SRY_CANLF Sex-determining region Y protein OS=Canis lupus familiaris OX=9615 GN=SRY PE=3 SV=1	0.27	0.27	11.70	1590369.1	1	1	0	2.27	220	347
>tr|J9P9J4|J9P9J4_CANLF Aldehyde dehydrogenase 1 family member A1 OS=Canis lupus familiaris OX=9615 GN=ALDH1A1 PE=3 SV=1	0.36	0.36	275.30	1566400.8	1	1	1	2.07	484	13765

>tr|E2RHV3|E2RHV3_CANLF Tripartite motif containing 23 OS=Canis lupus familiaris OX=9615 GN=TRIM23 PE=4 SV=3	0.10	0.00	61.90	1484135.6	1	1	0	0.87	574	1124
>sp|A2IBY8|MIP_CANLF Lens fiber major intrinsic protein OS=Canis lupus familiaris OX=9615 GN=MIP PE=2 SV=1	0.20	0.20	44.90	1412721.4	1	1	0	1.90	263	112
>tr|E2RI36|E2RI36_CANLF Nuclear transcription factor, X-box binding 1 OS=Canis lupus familiaris OX=9615 GN=NFX1 PE=3 SV=3	0.38	0.35	37.10	1382735.2	3	1	1	0.98	1118	13894
>tr|A0A5F4C5B9|A0A5F4C5B9_CANLF Glutathione S-transferase OS=Canis lupus familiaris OX=9615 GN=LOC481841 PE=3 SV=1	0.10	0.02	35.80	1151128.8	4	1	1	1.83	218	22818
>sp|O18840|ACTB_CANLF Actin, cytoplasmic 1 OS=Canis lupus familiaris OX=9615 GN=ACTB PE=2 SV=3	2.10	1.13	223.60	1148831.5	6	3	0	12.00	375	642
>tr|A0A5F4C2M0|A0A5F4C2M0_CANLF Rotatin OS=Canis lupus familiaris OX=9615 GN=RTTN PE=4 SV=1	0.20	0.20	75.60	1028978.0	1	1	0	0.18	2202	1797
>sp|P41148|ENPL_CANLF Endoplasmin OS=Canis lupus familiaris OX=9615 GN=HSP90B1 PE=1 SV=1	0.10	0.00	8.10	1005822.4	1	1	1	0.50	804	787
>tr|A0A5F4D0U7|A0A5F4D0U7_CANLF Structural maintenance of chromosomes flexible hinge domain containing 1 OS=Canis lupus familiaris OX=9615 GN=SMCHD1 PE=4 SV=1	0.35	0.35	84.80	977352.5	1	1	0	0.16	3051	9634
>tr|E2R9Y2|E2R9Y2_CANLF GB1/RHD3-type G domain-containing protein OS=Canis lupus familiaris OX=9615 GN=GBP1 PE=3 SV=3	0.39	0.35	70.40	955029.0	3	1	0	1.52	591	24472
>tr|E2RE16|E2RE16_CANLF Non-specific serine/threonine protein kinase OS=Canis lupus familiaris OX=9615 GN=PAK4 PE=4 SV=1	0.17	0.17	58.70	954967.4	1	1	0	0.84	592	12735
>tr|F1PPP9|F1PPP9_CANLF Family with sequence similarity 135 member A OS=Canis lupus familiaris OX=9615 GN=FAM135A PE=3 SV=3	0.49	0.50	114.10	933504.9	1	1	0	1.22	1399	6815
>tr|A0A5F4CEQ5|A0A5F4CEQ5_CANLF Steroid 11-beta-monooxygenase OS=Canis lupus familiaris OX=9615 GN=CYP11B2 PE=3 SV=1	0.57	0.57	10.50	915904.4	1	1	0	5.09	530	26732
>tr|A0A5F4DI80|A0A5F4DI80_CANLF Zinc finger protein Gfi-1 OS=Canis lupus familiaris OX=9615 GN=GFI1 PE=4 SV=1	1.14	1.12	115.40	907642.9	2	1	1	0.99	912	9754
>tr|J9P3H8|J9P3H8_CANLF ATM interactor OS=Canis lupus familiaris OX=9615 GN=ATMIN PE=4 SV=2	0.25	0.25	18.90	861906.8	1	1	0	0.58	863	882
>tr|J9NT31|J9NT31_CANLF Thymocyte selection associated family member 2 OS=Canis lupus familiaris OX=9615 GN=THEMIS2 PE=3 SV=1	0.10	0.01	42.00	830275.7	1	1	0	0.78	642	9120
>tr|J9NXS4|J9NXS4_CANLF SIN3 transcription regulator family member B OS=Canis lupus familiaris OX=9615 GN=SIN3B PE=4 SV=2	0.29	0.29	23.90	821092.8	2	1	1	1.24	1125	28078
>tr|E2RCT1|E2RCT1_CANLF WAP domain-containing protein OS=Canis lupus familiaris OX=9615 PE=4 SV=2	1.08	1.02	127.80	784291.3	4	1	0	14.66	116	21717
>tr|A0A5F4CEY4|A0A5F4CEY4_CANLF ELL associated factor 2 OS=Canis lupus familiaris OX=9615 GN=EAF2 PE=3 SV=1	0.10	0.02	35.60	721015.9	1	1	0	0.74	543	6686
>tr|F1Q3S8|F1Q3S8_CANLF Transmembrane protease serine OS=Canis lupus familiaris OX=9615 GN=TMPRSS11A PE=3 SV=3	0.27	0.27	79.60	710362.8	1	1	1	2.12	424	1890
>tr|E2RRM8|E2RRM8_CANLF Fer-1 like family member 6 OS=Canis lupus familiaris OX=9615 GN=FER1L6 PE=4 SV=3	0.14	0.14	42.00	631823.0	1	1	1	0.86	1868	6562
>tr|A0A5F4CRU1|A0A5F4CRU1_CANLF Rab interacting lysosomal protein like 1 OS=Canis lupus familiaris OX=9615 GN=RILPL1 PE=4 SV=1	0.10	0.01	12.70	610615.1	2	1	1	1.04	385	6326
>tr|E2RCT1|E2RCT1_CANLF WAP domain-containing protein OS=Canis lupus familiaris OX=9615 PE=4 SV=2	1.33	1.31	221.50	548065.3	2	1	0	14.66	116	21717

>sp|Q28895|NPC2_CANLF NPC intracellular cholesterol transporter 2 OS=Canis lupus familiaris OX=9615 GN=NPC2 PE=2 SV=1	2.24	1.19	354.10	542889.1	5	2	0	14.77	149	153
>tr|E2R459|E2R459_CANLF SET domain containing 9 OS=Canis lupus familiaris OX=9615 GN=SETD9 PE=4 SV=1	0.33	0.33	14.50	537266.0	1	1	0	7.02	299	34855
>tr|E2QV77|E2QV77_CANLF SPRY domain containing 4 OS=Canis lupus familiaris OX=9615 GN=SPRYD4 PE=4 SV=2	0.55	0.55	71.00	511164.3	1	1	0	3.94	254	8010
>tr|A0A5F4DCW2|A0A5F4DCW2_CANLF F-box and leucine rich repeat protein 18 OS=Canis lupus familiaris OX=9615 GN=FBXL18 PE=4 SV=1	0.24	0.24	122.50	483286.5	1	1	1	1.46	756	5739
>tr|E2R868|E2R868_CANLF [histone H4]-N-methyl-L-lysine20 N-methyltransferase KMT5B OS=Canis lupus familiaris OX=9615 GN=KMT5B PE=4 SV=3	0.10	0.01	23.90	477199.3	2	2	1	1.13	885	7704
>sp|Q28895|NPC2_CANLF NPC intracellular cholesterol transporter 2 OS=Canis lupus familiaris OX=9615 GN=NPC2 PE=2 SV=1	0.79	0.68	102.40	460040.4	2	2	0	14.77	149	153
>tr|E2RI36|E2RI36_CANLF Nuclear transcription factor, X-box binding 1 OS=Canis lupus familiaris OX=9615 GN=NFX1 PE=3 SV=3	0.15	0.15	6.10	429618.9	1	1	1	0.98	1118	13894
>tr|A0A5F4BQA2|A0A5F4BQA2_CANLF Dermatan sulfate epimerase OS=Canis lupus familiaris OX=9615 GN=DSE PE=3 SV=1	0.46	0.44	73.90	420173.8	2	1	0	2.60	308	8924
>tr|J9NW59|J9NW59_CANLF Ubiquitin carboxyl-terminal hydrolase OS=Canis lupus familiaris OX=9615 GN=USP33 PE=3 SV=1	0.10	0.00	14.10	393650.1	1	1	1	0.55	912	4415
>tr|E2RPZ0|E2RPZ0_CANLF Opsin 5 OS=Canis lupus familiaris OX=9615 GN=OPN5 PE=3 SV=3	0.10	0.07	13.10	385548.4	2	2	2	1.32	380	875
>tr|A0A5F4D105|A0A5F4D105_CANLF Amyloid beta precursor protein binding family A member 1 OS=Canis lupus familiaris OX=9615 GN=APBA1 PE=4 SV=1	0.10	0.10	50.40	358932.2	1	1	0	0.56	889	3602
>tr|A0A5F4BZW4|A0A5F4BZW4_CANLF Malonyl-CoA decarboxylase OS=Canis lupus familiaris OX=9615 GN=MLYCD PE=4 SV=1	0.19	0.19	40.90	355115.0	1	1	1	5.42	461	4809
>tr|A0A5F4CCD0|A0A5F4CCD0_CANLF Cysteine rich secretory protein 2 OS=Canis lupus familiaris OX=9615 GN=CRISP2 PE=3 SV=1	0.89	0.84	177.50	351994.1	4	1	0	4.82	311	11017
>tr|A0A5F4CBG5|A0A5F4CBG5_CANLF Ubiquitin specific peptidase 47 OS=Canis lupus familiaris OX=9615 GN=USP47 PE=4 SV=1	0.58	0.54	102.80	350029.6	3	1	0	1.62	1299	1284
>tr|A0A5F4D9S5|A0A5F4D9S5_CANLF Hyaluronoglucosaminidase OS=Canis lupus familiaris OX=9615 GN=CEMIP PE=3 SV=1	0.59	0.59	158.60	335957.8	1	1	0	0.24	1684	9775
>tr|F1P9Y3|F1P9Y3_CANLF Complex I-30kD OS=Canis lupus familiaris OX=9615 GN=NDUFS3 PE=3 SV=2	0.40	0.40	135.20	328949.1	1	1	0	4.04	322	18622
>tr|A0A5F4C854|A0A5F4C854_CANLF Proline-serine-threonine phosphatase interacting protein 1 OS=Canis lupus familiaris OX=9615 GN=PSTPIP1 PE=4 SV=1	0.21	0.21	55.20	319542.4	1	1	0	3.55	141	9178
>sp|E2QRY6|NNRE_CANLF NAD(P)H-hydrate epimerase OS=Canis lupus familiaris OX=9615 GN=NAXE PE=3 SV=1	0.55	0.55	42.40	310792.8	1	1	0	1.74	288	159
>tr|A0A5F4CW80|A0A5F4CW80_CANLF Sorbin and SH3 domain containing 1 OS=Canis lupus familiaris OX=9615 GN=SORBS1 PE=4 SV=1	0.26	0.26	19.40	307126.6	1	1	1	1.15	1303	30514
>tr|A0A5F4BUH7|A0A5F4BUH7_CANLF NADPH:adrenodoxin oxidoreductase, mitochondrial OS=Canis lupus familiaris OX=9615 GN=FDXR PE=3 SV=1	0.10	0.00	17.60	301631.0	3	1	1	0.89	451	5995
>tr|A0A5F4DHQ7|A0A5F4DHQ7_CANLF UDP-glucuronate decarboxylase 1 OS=Canis lupus familiaris OX=9615 GN=UXS1 PE=3 SV=1	0.21	0.19	25.40	281323.2	2	2	2	7.73	401	22885
>tr|A0A5F4BU36|A0A5F4BU36_CANLF Titin OS=Canis lupus familiaris OX=9615 GN=TTN PE=3 SV=1	0.52	0.54	42.20	273602.7	1	1	0	0.08	27097	33785

>tr|A0A5F4DA89|A0A5F4DA89_CANLF Sorting nexin 25 OS=Canis lupus familiaris OX=9615 GN=SNX25 PE=3 SV=1	0.40	0.41	70.90	267500.8	1	1	1	1.37	950	8417
>tr|E2RRM5|E2RRM5_CANLF Ras interacting protein 1 OS=Canis lupus familiaris OX=9615 GN=RASIP1 PE=4 SV=3	0.16	0.16	33.90	265549.4	1	1	0	0.62	1137	25466
>tr|J9P6K3|J9P6K3_CANLF PHD finger protein 2 OS=Canis lupus familiaris OX=9615 GN=PHF2 PE=4 SV=1	0.20	0.16	97.70	265542.8	3	1	1	1.28	1096	24614
>tr|E2QT82|E2QT82_CANLF Proteasome inhibitor PI31 subunit OS=Canis lupus familiaris OX=9615 GN=PSMF1 PE=3 SV=1	0.19	0.19	11.10	248517.3	1	1	1	8.86	271	11472
>tr|E2RFA3|E2RFA3_CANLF RNA helicase OS=Canis lupus familiaris OX=9615 GN=DDX18 PE=3 SV=2	0.82	0.82	112.10	245821.5	1	1	0	3.44	669	32049
>tr|A0A5F4CNT4|A0A5F4CNT4_CANLF Microtubule actin crosslinking factor 1 OS=Canis lupus familiaris OX=9615 GN=MACF1 PE=4 SV=1	0.11	0.12	59.50	242137.8	1	1	0	0.19	7352	1251
>tr|F1PDT8|F1PDT8_CANLF WAP four-disulfide core domain protein 2 OS=Canis lupus familiaris OX=9615 GN=WFDC2 PE=4 SV=3	0.90	0.90	58.00	239930.0	1	1	0	29.09	110	33116
>tr|E2QUV3|E2QUV3_CANLF Alpha-2-HS-glycoprotein OS=Canis lupus familiaris OX=9615 GN=AHSG PE=4 SV=2	1.19	1.17	201.70	235428.2	2	1	0	5.48	365	20747
>tr|A0A5F4DJ73|A0A5F4DJ73_CANLF Proline and serine rich coiled-coil 1 OS=Canis lupus familiaris OX=9615 GN=PSRC1 PE=4 SV=1	0.38	0.38	22.10	226854.1	1	1	0	4.93	345	1871
>tr|J9P2M6|J9P2M6_CANLF MSS51 mitochondrial translational activator OS=Canis lupus familiaris OX=9615 GN=MSS51 PE=4 SV=2	0.18	0.16	20.80	223042.7	2	1	1	2.41	457	41081
>tr|A0A5F4C739|A0A5F4C739_CANLF Rho guanine nucleotide exchange factor 15 OS=Canis lupus familiaris OX=9615 GN=ARHGEF15 PE=4 SV=1	0.23	0.23	6.30	218234.1	1	1	0	2.37	885	14702
>tr|J9P3D0|J9P3D0_CANLF Solute carrier family 4 member 9 OS=Canis lupus familiaris OX=9615 GN=SLC4A9 PE=3 SV=2	0.47	0.47	76.40	177288.7	1	1	0	1.12	893	31921
>sp|E2QY99|MPP5_CANLF MAGUK p55 subfamily member 5 OS=Canis lupus familiaris OX=9615 GN=MPP5 PE=1 SV=1	0.19	0.19	64.50	175268.7	1	1	1	2.22	675	246
>tr|A0A5F4DG16|A0A5F4DG16_CANLF Myosin XVIIIA OS=Canis lupus familiaris OX=9615 GN=MYO18A PE=3 SV=1	0.41	0.41	74.30	162216.5	1	1	0	0.45	2426	21190
>tr|A0A5F4CLN6|A0A5F4CLN6_CANLF DLG associated protein 4 OS=Canis lupus familiaris OX=9615 GN=DLGAP4 PE=3 SV=1	0.43	0.43	69.40	139918.9	1	1	0	3.04	461	7940
>tr|F1PGQ6|F1PGQ6_CANLF Septin OS=Canis lupus familiaris OX=9615 GN=SEPTIN1 PE=3 SV=3	0.42	0.42	20.70	134677.7	1	1	1	4.34	369	22386
>tr|J9P5T2|J9P5T2_CANLF Non-specific serine/threonine protein kinase OS=Canis lupus familiaris OX=9615 GN=WNK3 PE=4 SV=2	0.10	0.00	9.10	127769.0	1	1	0	0.22	2294	5229
>tr|A0A5F4DDF1|A0A5F4DDF1_CANLF DDB1- and CUL4-associated factor 10 OS=Canis lupus familiaris OX=9615 GN=DCAF10 PE=3 SV=1	0.88	0.88	49.90	122484.2	1	1	0	2.66	526	2777
>tr|E2R0D7|E2R0D7_CANLF Collagen type VI alpha 6 chain OS=Canis lupus familiaris OX=9615 GN=COL6A6 PE=4 SV=2	0.23	0.24	46.80	122484.2	1	1	0	0.62	2268	14682
>tr|A0A5F4CCD0|A0A5F4CCD0_CANLF Cysteine rich secretory protein 2 OS=Canis lupus familiaris OX=9615 GN=CRISP2 PE=3 SV=1	0.64	0.64	109.90	117327.5	1	1	0	4.82	311	11017
>tr|F1Q3Q6|F1Q3Q6_CANLF Secernin 1 OS=Canis lupus familiaris OX=9615 GN=SCRN1 PE=3 SV=2	0.10	0.01	55.90	108714.0	1	1	1	3.65	438	38892
>tr|J9P816|J9P816_CANLF E3 ubiquitin-protein transferase MAEA OS=Canis lupus familiaris OX=9615 GN=MAEA PE=4 SV=2	0.10	0.00	31.30	104483.4	1	1	1	1.41	355	1538

>tr|A0A5F4CB90|A0A5F4CB90_CANLF Nuclear cap-binding protein subunit 2 OS=Canis lupus familiaris OX=9615 PE=3 SV=1	0.58	0.58	48.10	102484.5	1	1	0	4.52	155	44031
>tr|F1PA94|F1PA94_CANLF FRY like transcription coactivator OS=Canis lupus familiaris OX=9615 GN=FRYL PE=4 SV=3	1.05	1.05	61.20	97525.8	1	1	0	0.30	3014	11447
>tr|J9P494|J9P494_CANLF SH3 domain-containing protein OS=Canis lupus familiaris OX=9615 GN=NEB PE=4 SV=2	0.28	0.29	12.00	90598.5	1	1	0	0.17	6526	2878
>tr|A0A5F4D463|A0A5F4D463_CANLF Roundabout guidance receptor 1 OS=Canis lupus familiaris OX=9615 GN=ROBO1 PE=4 SV=1	0.22	0.22	37.70	89484.8	1	1	0	1.42	1691	5706
>tr|A0A5F4C4P0|A0A5F4C4P0_CANLF Zinc finger FYVE domain-containing protein 26 OS=Canis lupus familiaris OX=9615 GN=ZFYVE26 PE=4 SV=1	0.24	0.24	37.20	88482.3	1	1	0	0.43	2339	3561
>tr|F1PFD6|F1PFD6_CANLF Teneurin transmembrane protein 4 OS=Canis lupus familiaris OX=9615 GN=TENM4 PE=3 SV=3	0.55	0.55	34.80	74097.8	1	1	0	0.32	2852	5565
>tr|E2R6E0|E2R6E0_CANLF Lipocln_cytosolic_FA-bd_dom domain-containing protein OS=Canis lupus familiaris OX=9615 GN=LCNL1 PE=3 SV=2	1.03	1.03	113.50	73419.6	1	1	0	3.34	299	1932
>tr|A0A5F4C7I9|A0A5F4C7I9_CANLF Ubiquitin specific peptidase 34 OS=Canis lupus familiaris OX=9615 GN=USP34 PE=4 SV=1	0.10	0.00	25.80	73019.5	1	1	1	0.14	3682	1536
