# Supplementary material for: Age-Dependent Variations in Functional Quality and Proteomic Characteristics of Canine (Canis lupus familiaris) Epididymal Spermatozoa
Source: Int J Mol Sci. 2022 Aug 15;23(16):9143. doi: 10.3390/ijms23169143 (PMC9409041; doi:10.3390/ijms23169143)
Supplement: Supplementary file 1 [file ijms-23-09143-s001.zip › Supplementary Table S2.pdf]

**Table S2** Supplementary. Proteins of Group 2 (42 to 77 months old) dog (*Canis lupus familiaris*) epididymal spermatozoa evaluated by mass spectrometry (NanoUPLC-Q-TOF/MS).

| Description | \|Log Prob\| | Best \|Log Prob\| | Best score | Total Intensity | # of spectra | # of unique peptides | # of mod peptides | Coverage % | # AA's in protein | Protein DB number |
|---|---|---|---|---|---|---|---|---|---|---|
| >tr\|F1PR54\|F1PR54_CANLF Lactotransferrin OS=Canis lupus familiaris OX=9615 GN=LTF PE=3 SV=1 | 17.90 | 4.31 | 326.60 | 1652321844.4 | 108 | 12 | 3 | 15.68 | 708 | 40436 |
| >tr\|F1PR54\|F1PR54_CANLF Lactotransferrin OS=Canis lupus familiaris OX=9615 GN=LTF PE=3 SV=1 | 18.80 | 4.55 | 338.80 | 1552918775.9 | 114 | 13 | 3 | 17.94 | 708 | 40436 |
| >tr\|A0A5F4BVF3\|A0A5F4BVF3_CANLF Lactotransferrin OS=Canis lupus familiaris OX=9615 GN=LTF PE=3 SV=1 | 17.82 | 3.11 | 340.00 | 862957897.6 | 85 | 11 | 1 | 18.69 | 626 | 32850 |
| >tr\|F1PR54\|F1PR54_CANLF Lactotransferrin OS=Canis lupus familiaris OX=9615 GN=LTF PE=3 SV=1 | 19.96 | 3.73 | 446.30 | 852673699.4 | 110 | 15 | 3 | 19.77 | 708 | 40436 |
| >tr\|F1PR54\|F1PR54_CANLF Lactotransferrin OS=Canis lupus familiaris OX=9615 GN=LTF PE=3 SV=1 | 12.05 | 2.61 | 394.20 | 820397119.9 | 79 | 12 | 2 | 15.68 | 708 | 40436 |
| >sp\|Q9XS65\|PTGDS_CANLF Prostaglandin-H2 D-isomerase OS=Canis lupus familiaris OX=9615 GN=PTGDS PE=2 SV=1 | 3.52 | 0.70 | 387.70 | 794551556.1 | 49 | 4 | 0 | 15.18 | 191 | 165 |
| >sp\|Q9XS65\|PTGDS_CANLF Prostaglandin-H2 D-isomerase OS=Canis lupus familiaris OX=9615 GN=PTGDS PE=2 SV=1 | 7.49 | 3.22 | 345.70 | 780863794.3 | 40 | 4 | 1 | 13.09 | 191 | 165 |
| >tr\|A0A5F4BVF3\|A0A5F4BVF3_CANLF Lactotransferrin OS=Canis lupus familiaris OX=9615 GN=LTF PE=3 SV=1 | 18.60 | 4.25 | 308.60 | 762179291.5 | 78 | 11 | 1 | 18.53 | 626 | 32850 |
| >tr\|A0A5F4BVF3\|A0A5F4BVF3_CANLF Lactotransferrin OS=Canis lupus familiaris OX=9615 GN=LTF PE=3 SV=1 | 8.01 | 2.43 | 352.20 | 759899559.8 | 60 | 10 | 0 | 13.90 | 626 | 32850 |
| >tr\|F1PR54\|F1PR54_CANLF Lactotransferrin OS=Canis lupus familiaris OX=9615 GN=LTF PE=3 SV=1 | 13.38 | 3.85 | 333.50 | 716046838.0 | 57 | 11 | 0 | 15.11 | 708 | 40436 |
| >sp\|Q9XS65\|PTGDS_CANLF Prostaglandin-H2 D-isomerase OS=Canis lupus familiaris OX=9615 GN=PTGDS PE=2 SV=1 | 5.24 | 2.10 | 365.40 | 669269929.8 | 55 | 5 | 1 | 15.18 | 191 | 165 |
| >sp\|P49822\|ALBU_CANLF Albumin OS=Canis lupus familiaris OX=9615 GN=ALB PE=1 SV=3 | 15.98 | 4.19 | 403.10 | 566233765.5 | 36 | 8 | 0 | 16.61 | 608 | 490 |
| >sp\|P49822\|ALBU_CANLF Albumin OS=Canis lupus familiaris OX=9615 GN=ALB PE=1 SV=3 | 17.51 | 4.64 | 399.80 | 526339247.1 | 37 | 8 | 0 | 16.61 | 608 | 490 |
| >sp\|Q9XS65\|PTGDS_CANLF Prostaglandin-H2 D-isomerase OS=Canis lupus familiaris OX=9615 GN=PTGDS PE=2 SV=1 | 3.65 | 2.06 | 362.80 | 515715047.0 | 31 | 3 | 1 | 13.09 | 191 | 165 |
| >tr\|F1PJ71\|F1PJ71_CANLF Glutathione peroxidase OS=Canis lupus familiaris OX=9615 GN=GPX5 PE=3 SV=2 | 4.67 | 2.08 | 360.00 | 436043750.2 | 29 | 6 | 1 | 27.60 | 221 | 19009 |
| >sp\|O18840\|ACTB_CANLF Actin, cytoplasmic 1 OS=Canis lupus familiaris OX=9615 GN=ACTB PE=2 SV=3 | 8.05 | 3.92 | 296.40 | 390446920.9 | 21 | 4 | 0 | 13.07 | 375 | 642 |
| >sp\|O18840\|ACTB_CANLF Actin, cytoplasmic 1 OS=Canis lupus familiaris OX=9615 GN=ACTB PE=2 SV=3 | 4.31 | 2.86 | 356.40 | 386319672.7 | 26 | 3 | 0 | 5.60 | 375 | 642 |
| >sp\|O18840\|ACTB_CANLF Actin, cytoplasmic 1 OS=Canis lupus familiaris OX=9615 GN=ACTB PE=2 SV=3 | 5.26 | 3.70 | 332.70 | 377724865.9 | 20 | 3 | 0 | 5.60 | 375 | 642 |
| >sp\|O18840\|ACTB_CANLF Actin, cytoplasmic 1 OS=Canis lupus familiaris OX=9615 GN=ACTB PE=2 SV=3 | 6.13 | 3.45 | 266.80 | 363808552.4 | 21 | 4 | 0 | 13.07 | 375 | 642 |
| >tr\|A0A5F4C0U6\|A0A5F4C0U6_CANLF MLLT1 super elongation complex subunit OS=Canis lupus familiaris OX=9615 GN=MLLT1 PE=4 SV=1 | 0.10 | 0.00 | 138.50 | 313799140.8 | 34 | 2 | 0 | 1.17 | 597 | 1602 |

| | | | | | | | | | | |
|---|---|---|---|---|---|---|---|---|---|---|
| >sp\|Q28895\|NPC2_CANLF NPC intracellular cholesterol transporter 2 OS=Canis lupus familiaris OX=9615 GN=NPC2 PE=2 SV=1 | 11.15 | 4.65 | 591.20 | 305690403.5 | 52 | 3 | 0 | 30.20 | 149 | 153 |
| >sp\|O18840\|ACTB_CANLF Actin, cytoplasmic 1 OS=Canis lupus familiaris OX=9615 GN=ACTB PE=2 SV=3 | 3.20 | 1.90 | 324.10 | 285008321.9 | 19 | 3 | 0 | 7.73 | 375 | 642 |
| >tr\|A0A5F4D9S5\|A0A5F4D9S5_CANLF Hyaluronoglucosaminidase OS=Canis lupus familiaris OX=9615 GN=CEMIP PE=3 SV=1 | 1.01 | 0.65 | 166.50 | 280157362.3 | 19 | 1 | 0 | 0.24 | 1684 | 9775 |
| >sp\|O18840\|ACTB_CANLF Actin, cytoplasmic 1 OS=Canis lupus familiaris OX=9615 GN=ACTB PE=2 SV=3 | 4.32 | 2.89 | 325.00 | 271563599.7 | 18 | 2 | 0 | 5.33 | 375 | 642 |
| >sp\|Q28895\|NPC2_CANLF NPC intracellular cholesterol transporter 2 OS=Canis lupus familiaris OX=9615 GN=NPC2 PE=2 SV=1 | 7.98 | 3.65 | 566.70 | 270657261.6 | 55 | 2 | 0 | 14.77 | 149 | 153 |
| >sp\|P62286\|ASPM_CANLF Abnormal spindle-like microcephaly-associated protein homolog OS=Canis lupus familiaris OX=9615 GN=ASPM PE=2 SV=2 | 1.18 | 1.10 | 94.10 | 258449448.8 | 29 | 2 | 0 | 0.14 | 3469 | 677 |
| >tr\|A0A5F4D9S5\|A0A5F4D9S5_CANLF Hyaluronoglucosaminidase OS=Canis lupus familiaris OX=9615 GN=CEMIP PE=3 SV=1 | 1.25 | 0.91 | 168.80 | 257697074.2 | 18 | 1 | 0 | 0.24 | 1684 | 9775 |
| >sp\|Q6AW47\|EST5A_CANLF Carboxylesterase 5A OS=Canis lupus familiaris OX=9615 GN=CES5A PE=2 SV=1 | 2.79 | 1.73 | 214.80 | 237651160.2 | 22 | 5 | 0 | 6.78 | 575 | 629 |
| >tr\|F1PBU5\|F1PBU5_CANLF Non-specific serine/threonine protein kinase OS=Canis lupus familiaris OX=9615 GN=SMG1 PE=3 SV=3 | 0.68 | 0.33 | 172.00 | 229564678.8 | 17 | 2 | 0 | 0.17 | 3634 | 6898 |
| >sp\|Q28895\|NPC2_CANLF NPC intracellular cholesterol transporter 2 OS=Canis lupus familiaris OX=9615 GN=NPC2 PE=2 SV=1 | 5.58 | 2.67 | 466.20 | 229332681.6 | 35 | 3 | 0 | 30.20 | 149 | 153 |
| >tr\|F1P884\|F1P884_CANLF PiggyBac transposable element derived 5 OS=Canis lupus familiaris OX=9615 GN=PGBD5 PE=4 SV=3 | 0.22 | 0.03 | 193.50 | 225718553.0 | 15 | 1 | 0 | 2.40 | 458 | 28672 |
| >tr\|F1PBU5\|F1PBU5_CANLF Non-specific serine/threonine protein kinase OS=Canis lupus familiaris OX=9615 GN=SMG1 PE=3 SV=3 | 0.93 | 0.35 | 175.80 | 224198462.4 | 16 | 2 | 0 | 0.17 | 3634 | 6898 |
| >sp\|O46607\|GPX5_CANLF Epididymal secretory glutathione peroxidase OS=Canis lupus familiaris OX=9615 GN=GPX5 PE=2 SV=1 | 1.59 | 1.32 | 255.10 | 216045839.2 | 13 | 2 | 0 | 8.60 | 221 | 564 |
| >tr\|A0A5F4BQW4\|A0A5F4BQW4_CANLF Zinc finger protein 654 OS=Canis lupus familiaris OX=9615 GN=ZNF654 PE=4 SV=1 | 0.65 | 0.35 | 184.70 | 215177541.3 | 16 | 1 | 1 | 1.03 | 1170 | 6910 |
| >sp\|O18840\|ACTB_CANLF Actin, cytoplasmic 1 OS=Canis lupus familiaris OX=9615 GN=ACTB PE=2 SV=3 | 3.17 | 2.69 | 347.90 | 203712745.7 | 16 | 2 | 0 | 4.53 | 375 | 642 |
| >tr\|Q9XSV4\|Q9XSV4_CANLF CE10 protein OS=Canis lupus familiaris OX=9615 GN=ce10 PE=2 SV=1 | 3.82 | 3.16 | 309.90 | 197948110.7 | 37 | 2 | 0 | 9.09 | 110 | 41542 |
| >sp\|O18840\|ACTB_CANLF Actin, cytoplasmic 1 OS=Canis lupus familiaris OX=9615 GN=ACTB PE=2 SV=3 | 5.51 | 4.41 | 292.70 | 195437997.0 | 13 | 2 | 0 | 5.33 | 375 | 642 |
| >tr\|A0A5F4BQW4\|A0A5F4BQW4_CANLF Zinc finger protein 654 OS=Canis lupus familiaris OX=9615 GN=ZNF654 PE=4 SV=1 | 0.33 | 0.05 | 182.60 | 195358378.1 | 15 | 1 | 1 | 1.03 | 1170 | 6910 |
| >tr\|A0A5F4BVF3\|A0A5F4BVF3_CANLF Lactotransferrin OS=Canis lupus familiaris OX=9615 GN=LTF PE=3 SV=1 | 4.79 | 2.17 | 217.40 | 189715191.7 | 40 | 6 | 0 | 8.63 | 626 | 32850 |
| >sp\|Q9XS65\|PTGDS_CANLF Prostaglandin-H2 D-isomerase OS=Canis lupus familiaris OX=9615 GN=PTGDS PE=2 SV=1 | 2.51 | 1.33 | 296.30 | 188331634.4 | 17 | 2 | 1 | 10.47 | 191 | 165 |
| >sp\|Q28895\|NPC2_CANLF NPC intracellular cholesterol transporter 2 OS=Canis lupus familiaris OX=9615 GN=NPC2 PE=2 SV=1 | 12.19 | 5.02 | 522.60 | 184771152.5 | 42 | 3 | 0 | 30.20 | 149 | 153 |
| >tr\|Q9XSV4\|Q9XSV4_CANLF CE10 protein OS=Canis lupus familiaris OX=9615 GN=ce10 PE=2 SV=1 | 6.12 | 3.91 | 298.90 | 183203134.3 | 40 | 3 | 0 | 12.73 | 110 | 41542 |

| | | | | | | | | | | |
|---|---|---|---|---|---|---|---|---|---|---|
| >tr\|F1PGF9\|F1PGF9_CANLF Rho guanine nucleotide exchange factor 26 OS=Canis lupus familiaris OX=9615 GN=ARHGEF26 PE=4 SV=3 | 0.39 | 0.25 | 284.80 | 180919513.8 | 8 | 1 | 0 | 0.84 | 594 | 22876 |
| >tr\|Q9XSV4\|Q9XSV4_CANLF CE10 protein OS=Canis lupus familiaris OX=9615 GN=ce10 PE=2 SV=1 | 4.59 | 3.91 | 289.60 | 176281226.2 | 35 | 2 | 0 | 9.09 | 110 | 41542 |
| >sp\|Q28895\|NPC2_CANLF NPC intracellular cholesterol transporter 2 OS=Canis lupus familiaris OX=9615 GN=NPC2 PE=2 SV=1 | 4.36 | 2.00 | 494.20 | 171585154.1 | 31 | 3 | 0 | 30.20 | 149 | 153 |
| >tr\|F1PGF9\|F1PGF9_CANLF Rho guanine nucleotide exchange factor 26 OS=Canis lupus familiaris OX=9615 GN=ARHGEF26 PE=4 SV=3 | 0.29 | 0.17 | 313.90 | 170134699.1 | 7 | 1 | 0 | 0.84 | 594 | 22876 |
| >tr\|Q9XSV4\|Q9XSV4_CANLF CE10 protein OS=Canis lupus familiaris OX=9615 GN=ce10 PE=2 SV=1 | 3.94 | 2.94 | 283.90 | 160368534.8 | 32 | 3 | 0 | 20.91 | 110 | 41542 |
| >tr\|Q9XSV4\|Q9XSV4_CANLF CE10 protein OS=Canis lupus familiaris OX=9615 GN=ce10 PE=2 SV=1 | 2.35 | 1.15 | 235.90 | 154185585.8 | 29 | 3 | 0 | 20.91 | 110 | 41542 |
| >sp\|F1PRN2\|MYO1D_CANLF Unconventional myosin-Id OS=Canis lupus familiaris OX=9615 GN=MYO1D PE=1 SV=2 | 0.65 | 0.24 | 167.80 | 153665307.0 | 13 | 2 | 0 | 0.50 | 1006 | 763 |
| >tr\|A0A5F4D7J3\|A0A5F4D7J3_CANLF Non-specific serine/threonine protein kinase OS=Canis lupus familiaris OX=9615 GN=CDC42BPA PE=3 SV=1 | 1.83 | 1.29 | 257.40 | 150266888.8 | 22 | 2 | 0 | 0.39 | 1794 | 1069 |
| >sp\|O46607\|GPX5_CANLF Epididymal secretory glutathione peroxidase OS=Canis lupus familiaris OX=9615 GN=GPX5 PE=2 SV=1 | 0.95 | 0.87 | 218.70 | 144258937.3 | 6 | 2 | 0 | 8.60 | 221 | 564 |
| >sp\|Q9XS65\|PTGDS_CANLF Prostaglandin-H2 D-isomerase OS=Canis lupus familiaris OX=9615 GN=PTGDS PE=2 SV=1 | 2.96 | 1.57 | 286.80 | 144101989.8 | 12 | 3 | 1 | 10.47 | 191 | 165 |
| >sp\|Q9MZY0\|CP2E1_CANLF Cytochrome P450 2E1 OS=Canis lupus familiaris OX=9615 GN=CYP2E1 PE=2 SV=1 | 0.45 | 0.27 | 113.60 | 144098969.0 | 8 | 2 | 1 | 4.66 | 494 | 522 |
| >sp\|Q6AW47\|EST5A_CANLF Carboxylesterase 5A OS=Canis lupus familiaris OX=9615 GN=CES5A PE=2 SV=1 | 0.75 | 0.51 | 201.10 | 143024166.5 | 12 | 4 | 0 | 7.83 | 575 | 629 |
| >tr\|Q9XSV4\|Q9XSV4_CANLF CE10 protein OS=Canis lupus familiaris OX=9615 GN=ce10 PE=2 SV=1 | 5.15 | 3.09 | 306.70 | 138867227.7 | 28 | 2 | 0 | 9.09 | 110 | 41542 |
| >tr\|J9NYP3\|J9NYP3_CANLF THAP domain containing 7 OS=Canis lupus familiaris OX=9615 GN=THAP7 PE=4 SV=2 | 0.12 | 0.01 | 126.20 | 130894182.5 | 44 | 2 | 1 | 2.43 | 411 | 5194 |
| >tr\|Q9XSV4\|Q9XSV4_CANLF CE10 protein OS=Canis lupus familiaris OX=9615 GN=ce10 PE=2 SV=1 | 4.93 | 3.25 | 278.70 | 130506796.3 | 27 | 2 | 0 | 9.09 | 110 | 41542 |
| >tr\|E2RJR2\|E2RJR2_CANLF NADH:ubiquinone oxidoreductase subunit A9 OS=Canis lupus familiaris OX=9615 GN=NDUFA9 PE=4 SV=2 | 0.16 | 0.04 | 62.00 | 128300823.9 | 7 | 1 | 1 | 5.31 | 377 | 3530 |
| >sp\|Q2PQH8\|GDE_CANLF Glycogen debranching enzyme OS=Canis lupus familiaris OX=9615 GN=AGL PE=2 SV=1 | 0.59 | 0.18 | 124.30 | 127398547.3 | 14 | 2 | 0 | 0.26 | 1533 | 23 |
| >sp\|Q9XS65\|PTGDS_CANLF Prostaglandin-H2 D-isomerase OS=Canis lupus familiaris OX=9615 GN=PTGDS PE=2 SV=1 | 2.33 | 1.02 | 252.50 | 127280671.2 | 24 | 2 | 1 | 10.47 | 191 | 165 |
| >tr\|A0A5F4BU48\|A0A5F4BU48_CANLF Suppression of tumorigenicity 7 OS=Canis lupus familiaris OX=9615 GN=ST7 PE=3 SV=1 | 0.77 | 0.61 | 159.00 | 127117166.5 | 10 | 2 | 0 | 3.91 | 460 | 2504 |
| >sp\|Q9XS65\|PTGDS_CANLF Prostaglandin-H2 D-isomerase OS=Canis lupus familiaris OX=9615 GN=PTGDS PE=2 SV=1 | 2.71 | 1.83 | 322.10 | 123286338.8 | 11 | 2 | 1 | 10.47 | 191 | 165 |
| >tr\|E2R6E0\|E2R6E0_CANLF Lipocln_cytosolic_FA-bd_dom domain-containing protein OS=Canis lupus familiaris OX=9615 GN=LCNL1 PE=3 SV=2 | 3.49 | 2.19 | 207.80 | 122698749.5 | 10 | 4 | 0 | 11.37 | 299 | 1932 |
| >sp\|Q28895\|NPC2_CANLF NPC intracellular cholesterol transporter 2 OS=Canis lupus familiaris OX=9615 GN=NPC2 PE=2 SV=1 | 8.32 | 3.38 | 270.30 | 121461626.9 | 15 | 3 | 0 | 30.20 | 149 | 153 |

| | | | | | | | | | | |
|---|---|---|---|---|---|---|---|---|---|---|
| >sp\|Q9XS65\|PTGDS_CANLF Prostaglandin-H2 D-isomerase OS=Canis lupus familiaris OX=9615 GN=PTGDS PE=2 SV=1 | 1.45 | 1.21 | 330.20 | 121434526.9 | 12 | 2 | 1 | 10.47 | 191 | 165 |
| >sp\|O46607\|GPX5_CANLF Epididymal secretory glutathione peroxidase OS=Canis lupus familiaris OX=9615 GN=GPX5 PE=2 SV=1 | 2.00 | 1.50 | 225.60 | 119482095.6 | 8 | 2 | 0 | 8.60 | 221 | 564 |
| >tr\|E2RRF5\|E2RRF5_CANLF RNA binding motif protein 19 OS=Canis lupus familiaris OX=9615 GN=RBM19 PE=4 SV=3 | 0.10 | 0.00 | 56.10 | 118978307.2 | 49 | 2 | 0 | 0.72 | 970 | 905 |
| >sp\|O18840\|ACTB_CANLF Actin, cytoplasmic 1 OS=Canis lupus familiaris OX=9615 GN=ACTB PE=2 SV=3 | 4.97 | 4.18 | 383.10 | 113426229.3 | 19 | 2 | 0 | 5.33 | 375 | 642 |
| >tr\|F1PHP9\|F1PHP9_CANLF Ectopic P-granules autophagy protein 5 homolog OS=Canis lupus familiaris OX=9615 GN=EPG5 PE=3 SV=3 | 0.25 | 0.09 | 176.50 | 112742791.7 | 9 | 1 | 0 | 0.16 | 2574 | 20660 |
| >tr\|E2RE16\|E2RE16_CANLF Non-specific serine/threonine protein kinase OS=Canis lupus familiaris OX=9615 GN=PAK4 PE=4 SV=1 | 0.65 | 0.02 | 80.40 | 112709410.0 | 43 | 1 | 0 | 0.84 | 592 | 12735 |
| >tr\|E2RCT1\|E2RCT1_CANLF WAP domain-containing protein OS=Canis lupus familiaris OX=9615 PE=4 SV=2 | 1.36 | 0.89 | 222.00 | 112100494.3 | 8 | 2 | 0 | 9.48 | 116 | 21717 |
| >tr\|F1PI09\|F1PI09_CANLF Aldehyde oxidase OS=Canis lupus familiaris OX=9615 GN=AOX2 PE=3 SV=3 | 1.41 | 1.05 | 208.30 | 112062641.8 | 19 | 1 | 0 | 0.67 | 1347 | 21650 |
| >sp\|O18840\|ACTB_CANLF Actin, cytoplasmic 1 OS=Canis lupus familiaris OX=9615 GN=ACTB PE=2 SV=3 | 5.37 | 2.97 | 250.40 | 110965587.9 | 26 | 4 | 0 | 10.40 | 375 | 642 |
| >tr\|E2RCT1\|E2RCT1_CANLF WAP domain-containing protein OS=Canis lupus familiaris OX=9615 PE=4 SV=2 | 1.97 | 1.75 | 278.40 | 109486759.0 | 7 | 2 | 0 | 9.48 | 116 | 21717 |
| >tr\|A0A5F4CAH2\|A0A5F4CAH2_CANLF RNA polymerase II subunit A C-terminal domain phosphatase OS=Canis lupus familiaris OX=9615 GN=CTDP1 PE=4 SV=1 | 0.24 | 0.08 | 132.00 | 107646701.2 | 9 | 1 | 0 | 0.74 | 945 | 14396 |
| >tr\|E2R6E0\|E2R6E0_CANLF Lipocln_cytosolic_FA-bd_dom domain-containing protein OS=Canis lupus familiaris OX=9615 GN=LCNL1 PE=3 SV=2 | 1.62 | 1.50 | 200.90 | 107134375.8 | 7 | 1 | 0 | 3.01 | 299 | 1932 |
| >tr\|F1PI09\|F1PI09_CANLF Aldehyde oxidase OS=Canis lupus familiaris OX=9615 GN=AOX2 PE=3 SV=3 | 1.57 | 1.23 | 204.30 | 106793370.4 | 18 | 1 | 0 | 0.67 | 1347 | 21650 |
| >sp\|Q9GL25\|ESPB1_CANLF Epididymal sperm-binding protein 1 OS=Canis lupus familiaris OX=9615 GN=ELSPBP1 PE=1 SV=1 | 0.82 | 0.68 | 212.10 | 103955444.1 | 8 | 2 | 0 | 11.43 | 245 | 36 |
| >tr\|E2RCT1\|E2RCT1_CANLF WAP domain-containing protein OS=Canis lupus familiaris OX=9615 PE=4 SV=2 | 1.43 | 0.95 | 186.80 | 103331089.4 | 7 | 2 | 0 | 9.48 | 116 | 21717 |
| >tr\|E2R6E0\|E2R6E0_CANLF Lipocln_cytosolic_FA-bd_dom domain-containing protein OS=Canis lupus familiaris OX=9615 GN=LCNL1 PE=3 SV=2 | 2.06 | 1.67 | 172.90 | 102342870.4 | 8 | 3 | 0 | 11.37 | 299 | 1932 |
| >tr\|J9NTK2\|J9NTK2_CANLF J domain-containing protein OS=Canis lupus familiaris OX=9615 GN=DNAJC12 PE=4 SV=2 | 0.10 | 0.00 | 164.10 | 100959245.2 | 6 | 1 | 0 | 4.72 | 106 | 2310 |
| >sp\|P49822\|ALBU_CANLF Albumin OS=Canis lupus familiaris OX=9615 GN=ALB PE=1 SV=3 | 1.43 | 0.65 | 419.20 | 99925622.3 | 8 | 3 | 0 | 6.41 | 608 | 490 |
| >tr\|E2R6E0\|E2R6E0_CANLF Lipocln_cytosolic_FA-bd_dom domain-containing protein OS=Canis lupus familiaris OX=9615 GN=LCNL1 PE=3 SV=2 | 1.53 | 1.41 | 234.60 | 98988824.2 | 7 | 1 | 0 | 3.01 | 299 | 1932 |
| >tr\|A0A5F4BZW4\|A0A5F4BZW4_CANLF Malonyl-CoA decarboxylase OS=Canis lupus familiaris OX=9615 GN=MLYCD PE=4 SV=1 | 0.19 | 0.07 | 193.00 | 98937958.4 | 7 | 1 | 0 | 1.30 | 461 | 4809 |
| >sp\|Q9GL25\|ESPB1_CANLF Epididymal sperm-binding protein 1 OS=Canis lupus familiaris OX=9615 GN=ELSPBP1 PE=1 SV=1 | 2.84 | 1.65 | 213.60 | 96733442.7 | 7 | 2 | 0 | 11.43 | 245 | 36 |
| >tr\|F1PGF9\|F1PGF9_CANLF Rho guanine nucleotide exchange factor 26 OS=Canis lupus familiaris OX=9615 GN=ARHGEF26 PE=4 SV=3 | 2.09 | 2.05 | 312.30 | 95855749.2 | 4 | 2 | 1 | 2.36 | 594 | 22876 |

| | | | | | | | | | | |
|---|---|---|---|---|---|---|---|---|---|---|
| >tr\|Q9XSV4\|Q9XSV4_CANLF CE10 protein OS=Canis lupus familiaris OX=9615 GN=ce10 PE=2 SV=1 | 3.57 | 2.76 | 232.60 | 91654096.5 | 37 | 3 | 0 | 12.73 | 110 | 41542 |
| >tr\|E2RCT1\|E2RCT1_CANLF WAP domain-containing protein OS=Canis lupus familiaris OX=9615 PE=4 SV=2 | 3.05 | 2.11 | 263.60 | 91410460.8 | 6 | 2 | 0 | 9.48 | 116 | 21717 |
| >tr\|J9NTK2\|J9NTK2_CANLF J domain-containing protein OS=Canis lupus familiaris OX=9615 GN=DNAJC12 PE=4 SV=2 | 0.29 | 0.21 | 178.20 | 90115296.5 | 5 | 1 | 0 | 4.72 | 106 | 2310 |
| >sp\|Q5QQ50\|XYLT2_CANLF Xylosyltransferase 2 OS=Canis lupus familiaris OX=9615 GN=XYLT2 PE=2 SV=1 | 0.15 | 0.03 | 205.90 | 90099853.9 | 7 | 2 | 1 | 0.69 | 865 | 388 |
| >tr\|A0A5F4CLI1\|A0A5F4CLI1_CANLF Histone deacetylase 6 OS=Canis lupus familiaris OX=9615 GN=HDAC6 PE=4 SV=1 | 0.82 | 0.72 | 168.10 | 87837816.6 | 6 | 1 | 0 | 0.43 | 1175 | 4057 |
| >tr\|F1PJ71\|F1PJ71_CANLF Glutathione peroxidase OS=Canis lupus familiaris OX=9615 GN=GPX5 PE=3 SV=2 | 0.89 | 0.44 | 229.40 | 87562014.0 | 9 | 5 | 1 | 26.24 | 221 | 19009 |
| >tr\|A0A5F4CPU3\|A0A5F4CPU3_CANLF SEC24 homolog D, COPII coat complex component OS=Canis lupus familiaris OX=9615 GN=SEC24D PE=3 SV=1 | 0.46 | 0.02 | 64.00 | 86457550.0 | 36 | 1 | 0 | 0.53 | 946 | 2125 |
| >tr\|J9NS28\|J9NS28_CANLF RBR-type E3 ubiquitin transferase OS=Canis lupus familiaris OX=9615 GN=ANKIB1 PE=4 SV=2 | 0.56 | 0.50 | 46.20 | 86444910.9 | 4 | 1 | 0 | 2.33 | 988 | 26345 |
| >tr\|A0A5F4C730\|A0A5F4C730_CANLF Semaphorin 4D OS=Canis lupus familiaris OX=9615 GN=SEMA4D PE=3 SV=1 | 0.46 | 0.28 | 217.70 | 86184259.5 | 10 | 1 | 0 | 0.28 | 1067 | 1802 |
| >sp\|F1PRN2\|MYO1D_CANLF Unconventional myosin-Id OS=Canis lupus familiaris OX=9615 GN=MYO1D PE=1 SV=2 | 0.43 | 0.23 | 199.40 | 85900296.0 | 11 | 1 | 0 | 0.30 | 1006 | 763 |
| >tr\|A0A5F4BVF3\|A0A5F4BVF3_CANLF Lactotransferrin OS=Canis lupus familiaris OX=9615 GN=LTF PE=3 SV=1 | 2.97 | 0.77 | 245.20 | 85106957.9 | 21 | 8 | 0 | 12.78 | 626 | 32850 |
| >sp\|F1PRN2\|MYO1D_CANLF Unconventional myosin-Id OS=Canis lupus familiaris OX=9615 GN=MYO1D PE=1 SV=2 | 1.03 | 0.80 | 183.10 | 84640402.1 | 9 | 2 | 0 | 0.50 | 1006 | 763 |
| >tr\|F6XHP7\|F6XHP7_CANLF Centrosomal protein 104 OS=Canis lupus familiaris OX=9615 GN=CEP104 PE=4 SV=1 | 0.18 | 0.08 | 83.10 | 83130079.1 | 6 | 1 | 0 | 0.76 | 925 | 15362 |
| >tr\|A0A5F4D967\|A0A5F4D967_CANLF GLI family zinc finger 1 OS=Canis lupus familiaris OX=9615 GN=GLI1 PE=3 SV=1 | 0.51 | 0.05 | 163.90 | 83105868.0 | 24 | 1 | 0 | 0.70 | 1139 | 11817 |
| >tr\|F1PTD0\|F1PTD0_CANLF F-box and WD repeat domain containing 8 OS=Canis lupus familiaris OX=9615 GN=FBXW8 PE=4 SV=3 | 0.14 | 0.02 | 32.10 | 82206437.4 | 7 | 1 | 1 | 1.83 | 546 | 7542 |
| >tr\|E2RCT1\|E2RCT1_CANLF WAP domain-containing protein OS=Canis lupus familiaris OX=9615 PE=4 SV=2 | 0.52 | 0.43 | 259.80 | 81343342.7 | 6 | 2 | 0 | 9.48 | 116 | 21717 |
| >tr\|Q9XSV4\|Q9XSV4_CANLF CE10 protein OS=Canis lupus familiaris OX=9615 GN=ce10 PE=2 SV=1 | 6.34 | 4.46 | 279.60 | 80851370.9 | 19 | 3 | 0 | 12.73 | 110 | 41542 |
| >tr\|F1P8F3\|F1P8F3_CANLF Utrophin OS=Canis lupus familiaris OX=9615 GN=UTRN PE=4 SV=3 | 0.64 | 0.39 | 134.00 | 80088013.7 | 5 | 2 | 1 | 0.67 | 3421 | 13521 |
| >sp\|Q9GL25\|ESPB1_CANLF Epididymal sperm-binding protein 1 OS=Canis lupus familiaris OX=9615 GN=ELSPBP1 PE=1 SV=1 | 1.36 | 0.99 | 198.60 | 79737602.4 | 8 | 2 | 0 | 11.43 | 245 | 36 |
| >sp\|Q9GL25\|ESPB1_CANLF Epididymal sperm-binding protein 1 OS=Canis lupus familiaris OX=9615 GN=ELSPBP1 PE=1 SV=1 | 1.84 | 1.69 | 210.20 | 79643556.9 | 6 | 2 | 0 | 11.43 | 245 | 36 |
| >sp\|Q2PQH8\|GDE_CANLF Glycogen debranching enzyme OS=Canis lupus familiaris OX=9615 GN=AGL PE=2 SV=1 | 2.49 | 2.28 | 112.90 | 79288050.0 | 5 | 2 | 0 | 0.26 | 1533 | 23 |
| >tr\|F1P8F3\|F1P8F3_CANLF Utrophin OS=Canis lupus familiaris OX=9615 GN=UTRN PE=4 SV=3 | 0.16 | 0.06 | 137.40 | 79124383.3 | 5 | 2 | 1 | 0.67 | 3421 | 13521 |

| | | | | | | | | | | |
|---|---|---|---|---|---|---|---|---|---|---|
| >tr\|A0A5F4CCD0\|A0A5F4CCD0_CANLF Cysteine rich secretory protein 2 OS=Canis lupus familiaris OX=9615 GN=CRISP2 PE=3 SV=1 | 1.47 | 1.37 | 285.30 | 78862207.4 | 7 | 1 | 0 | 2.25 | 311 | 11017 |
| >tr\|A0A5F4C730\|A0A5F4C730_CANLF Semaphorin 4D OS=Canis lupus familiaris OX=9615 GN=SEMA4D PE=3 SV=1 | 0.58 | 0.41 | 221.10 | 78465837.4 | 10 | 1 | 0 | 0.28 | 1067 | 1802 |
| >tr\|J9PAZ6\|J9PAZ6_CANLF Hyperpolarization activated cyclic nucleotide gated potassium channel 4 OS=Canis lupus familiaris OX=9615 GN=HCN4 PE=4 SV=2 | 0.10 | 0.00 | 71.30 | 76259828.2 | 4 | 1 | 1 | 4.91 | 530 | 19838 |
| >sp\|P25473\|CLUS_CANLF Clusterin OS=Canis lupus familiaris OX=9615 GN=CLU PE=2 SV=1 | 0.26 | 0.17 | 214.30 | 76135462.6 | 6 | 2 | 0 | 3.37 | 445 | 725 |
| >tr\|J9P9X5\|J9P9X5_CANLF ST14 transmembrane serine protease matriptase OS=Canis lupus familiaris OX=9615 GN=ST14 PE=4 SV=2 | 0.42 | 0.32 | 201.30 | 75671593.7 | 6 | 1 | 0 | 0.45 | 882 | 2776 |
| >tr\|A0A5F4CQM7\|A0A5F4CQM7_CANLF PX domain-containing protein OS=Canis lupus familiaris OX=9615 PE=4 SV=1 | 0.11 | 0.04 | 143.40 | 75586462.4 | 6 | 1 | 1 | 7.87 | 127 | 39214 |
| >sp\|P62286\|ASPM_CANLF Abnormal spindle-like microcephaly-associated protein homolog OS=Canis lupus familiaris OX=9615 GN=ASPM PE=2 SV=2 | 0.30 | 0.11 | 96.40 | 72175653.0 | 6 | 3 | 0 | 0.23 | 3469 | 677 |
| >sp\|P25473\|CLUS_CANLF Clusterin OS=Canis lupus familiaris OX=9615 GN=CLU PE=2 SV=1 | 0.50 | 0.42 | 242.10 | 70860716.8 | 5 | 1 | 0 | 0.90 | 445 | 725 |
| >tr\|J9NS28\|J9NS28_CANLF RBR-type E3 ubiquitin transferase OS=Canis lupus familiaris OX=9615 GN=ANKIB1 PE=4 SV=2 | 0.10 | 0.00 | 44.40 | 70515348.5 | 3 | 1 | 0 | 2.33 | 988 | 26345 |
| >tr\|A0A5F4D0B3\|A0A5F4D0B3_CANLF Bromodomain containing 1 OS=Canis lupus familiaris OX=9615 GN=BRD1 PE=4 SV=1 | 0.10 | 0.05 | 184.70 | 70399871.5 | 4 | 1 | 0 | 0.81 | 1112 | 1580 |
| >tr\|A0A5F4CCD0\|A0A5F4CCD0_CANLF Cysteine rich secretory protein 2 OS=Canis lupus familiaris OX=9615 GN=CRISP2 PE=3 SV=1 | 2.35 | 2.24 | 286.20 | 68883099.5 | 7 | 2 | 0 | 7.07 | 311 | 11017 |
| >tr\|E2RPK8\|E2RPK8_CANLF Phosphatidylethanolamine binding protein 4 OS=Canis lupus familiaris OX=9615 GN=PEBP4 PE=3 SV=2 | 3.92 | 3.25 | 270.50 | 68457823.8 | 5 | 2 | 0 | 7.29 | 247 | 4725 |
| >tr\|A0A5F4CPY7\|A0A5F4CPY7_CANLF von Willebrand factor A domain containing 5A OS=Canis lupus familiaris OX=9615 GN=VWA5A PE=4 SV=1 | 1.06 | 0.98 | 248.90 | 68042554.9 | 5 | 1 | 0 | 0.75 | 803 | 1309 |
| >tr\|A0A5F4DGF5\|A0A5F4DGF5_CANLF Alkaline phosphatase OS=Canis lupus familiaris OX=9615 GN=ALPL PE=3 SV=1 | 2.27 | 1.13 | 244.60 | 67703427.5 | 8 | 3 | 0 | 5.77 | 572 | 6357 |
| >tr\|E2RRP3\|E2RRP3_CANLF LIM homeobox 5 OS=Canis lupus familiaris OX=9615 GN=LHX5 PE=4 SV=1 | 0.11 | 0.05 | 167.90 | 67584106.6 | 4 | 1 | 0 | 2.24 | 402 | 19607 |
| >tr\|F1P8J6\|F1P8J6_CANLF RNA helicase OS=Canis lupus familiaris OX=9615 GN=DDX55 PE=3 SV=3 | 0.10 | 0.01 | 155.80 | 66489374.3 | 4 | 1 | 0 | 1.58 | 568 | 8934 |
| >tr\|E2RPK8\|E2RPK8_CANLF Phosphatidylethanolamine binding protein 4 OS=Canis lupus familiaris OX=9615 GN=PEBP4 PE=3 SV=2 | 4.64 | 2.38 | 255.40 | 64415870.7 | 5 | 2 | 0 | 7.29 | 247 | 4725 |
| >sp\|P49822\|ALBU_CANLF Albumin OS=Canis lupus familiaris OX=9615 GN=ALB PE=1 SV=3 | 0.52 | 0.39 | 196.70 | 64252726.8 | 6 | 2 | 0 | 4.28 | 608 | 490 |
| >tr\|A0A5F4CQL9\|A0A5F4CQL9_CANLF ATP-dependent RNA helicase DDX1 OS=Canis lupus familiaris OX=9615 GN=DDX1 PE=3 SV=1 | 0.21 | 0.16 | 129.00 | 64002380.6 | 4 | 1 | 0 | 1.15 | 784 | 7666 |
| >tr\|F1Q176\|F1Q176_CANLF Nuclear receptor subfamily 2 group E member 1 OS=Canis lupus familiaris OX=9615 GN=NR2E1 PE=3 SV=3 | 0.30 | 0.22 | 28.10 | 63637974.7 | 5 | 1 | 0 | 5.97 | 385 | 34373 |
| >tr\|F1PIV8\|F1PIV8_CANLF EF-hand domain family member D2 OS=Canis lupus familiaris OX=9615 GN=EFHD2 PE=4 SV=3 | 0.20 | 0.11 | 233.40 | 61863149.6 | 6 | 2 | 0 | 14.79 | 142 | 16068 |
| >tr\|A0A5F4CLI1\|A0A5F4CLI1_CANLF Histone deacetylase 6 OS=Canis lupus familiaris OX=9615 GN=HDAC6 PE=4 SV=1 | 1.00 | 0.94 | 195.20 | 61860410.1 | 4 | 1 | 0 | 0.43 | 1175 | 4057 |

| | | | | | | | | | | |
|---|---|---|---|---|---|---|---|---|---|---|
| >tr\|A0A5F4D8I6\|A0A5F4D8I6_CANLF Phospholipase A2 receptor 1 OS=Canis lupus familiaris OX=9615 GN=PLA2R1 PE=4 SV=1 | 0.71 | 0.39 | 163.90 | 61749741.6 | 17 | 1 | 0 | 0.50 | 1394 | 6796 |
| >sp\|O97758\|ZO1_CANLF Tight junction protein ZO-1 OS=Canis lupus familiaris OX=9615 GN=TJP1 PE=1 SV=1 | 0.42 | 0.20 | 87.00 | 61682721.7 | 5 | 2 | 0 | 0.23 | 1769 | 139 |
| >sp\|P25473\|CLUS_CANLF Clusterin OS=Canis lupus familiaris OX=9615 GN=CLU PE=2 SV=1 | 1.07 | 0.91 | 165.90 | 60288771.1 | 8 | 2 | 0 | 2.25 | 445 | 725 |
| >tr\|A0A5F4D7J3\|A0A5F4D7J3_CANLF Non-specific serine/threonine protein kinase OS=Canis lupus familiaris OX=9615 GN=CDC42BPA PE=3 SV=1 | 1.24 | 1.16 | 245.50 | 60079380.4 | 5 | 1 | 0 | 0.22 | 1794 | 1069 |
| >sp\|O46607\|GPX5_CANLF Epididymal secretory glutathione peroxidase OS=Canis lupus familiaris OX=9615 GN=GPX5 PE=2 SV=1 | 1.17 | 1.00 | 205.10 | 59727087.3 | 3 | 2 | 0 | 8.60 | 221 | 564 |
| >tr\|E2RG75\|E2RG75_CANLF Inactive ribonuclease-like protein 9 OS=Canis lupus familiaris OX=9615 GN=RNASE9 PE=3 SV=2 | 3.63 | 3.52 | 331.60 | 57884252.8 | 8 | 2 | 1 | 8.08 | 198 | 41734 |
| >tr\|A0A5F4C169\|A0A5F4C169_CANLF Dmx like 1 OS=Canis lupus familiaris OX=9615 GN=DMXL1 PE=4 SV=1 | 0.29 | 0.25 | 175.20 | 57461917.9 | 3 | 1 | 0 | 0.14 | 2937 | 1001 |
| >tr\|A0A5F4CZ62\|A0A5F4CZ62_CANLF Dynein cytoplasmic 1 heavy chain 1 OS=Canis lupus familiaris OX=9615 GN=DYNC1H1 PE=3 SV=1 | 0.18 | 0.09 | 212.00 | 57421347.5 | 5 | 2 | 0 | 0.16 | 4329 | 3271 |
| >tr\|A0A5F4DFX0\|A0A5F4DFX0_CANLF Exportin-T OS=Canis lupus familiaris OX=9615 GN=XPOT PE=3 SV=1 | 0.17 | 0.09 | 145.30 | 56971723.2 | 5 | 1 | 0 | 0.75 | 938 | 2329 |
| >sp\|P23685\|NAC1_CANLF Sodium/calcium exchanger 1 OS=Canis lupus familiaris OX=9615 GN=SLC8A1 PE=1 SV=1 | 0.13 | 0.07 | 165.10 | 56287577.8 | 4 | 1 | 0 | 1.03 | 970 | 764 |
| >tr\|E2RG75\|E2RG75_CANLF Inactive ribonuclease-like protein 9 OS=Canis lupus familiaris OX=9615 GN=RNASE9 PE=3 SV=2 | 3.30 | 3.13 | 304.70 | 55207079.5 | 8 | 2 | 1 | 8.08 | 198 | 41734 |
| >tr\|A0A5F4C0S7\|A0A5F4C0S7_CANLF HEAT repeat containing 5A OS=Canis lupus familiaris OX=9615 GN=HEATR5A PE=3 SV=1 | 0.33 | 0.02 | 65.40 | 52598492.9 | 23 | 1 | 0 | 0.25 | 1995 | 1753 |
| >sp\|Q076A6\|MYH1_CANLF Myosin-1 OS=Canis lupus familiaris OX=9615 GN=MYH1 PE=3 SV=2 | 0.10 | 0.02 | 123.30 | 52568652.7 | 5 | 1 | 0 | 0.10 | 1939 | 121 |
| >sp\|P49822\|ALBU_CANLF Albumin OS=Canis lupus familiaris OX=9615 GN=ALB PE=1 SV=3 | 0.16 | 0.06 | 162.50 | 52085471.2 | 5 | 2 | 0 | 4.28 | 608 | 490 |
| >sp\|Q28895\|NPC2_CANLF NPC intracellular cholesterol transporter 2 OS=Canis lupus familiaris OX=9615 GN=NPC2 PE=2 SV=1 | 9.55 | 4.67 | 501.20 | 51800673.6 | 30 | 2 | 0 | 14.77 | 149 | 153 |
| >tr\|A0A5F4CAH2\|A0A5F4CAH2_CANLF RNA polymerase II subunit A C-terminal domain phosphatase OS=Canis lupus familiaris OX=9615 GN=CTDP1 PE=4 SV=1 | 0.16 | 0.10 | 125.90 | 51455409.0 | 4 | 1 | 0 | 0.74 | 945 | 14396 |
| >tr\|A0A5F4DKY4\|A0A5F4DKY4_CANLF Dehydrogenase E1 and transketolase domain containing 1 OS=Canis lupus familiaris OX=9615 GN=DHTKD1 PE=3 SV=1 | 0.10 | 0.05 | 104.80 | 51440914.1 | 3 | 1 | 0 | 2.40 | 834 | 5545 |
| >tr\|A0A5F4CCD0\|A0A5F4CCD0_CANLF Cysteine rich secretory protein 2 OS=Canis lupus familiaris OX=9615 GN=CRISP2 PE=3 SV=1 | 1.87 | 1.79 | 243.20 | 50795729.4 | 5 | 1 | 0 | 2.25 | 311 | 11017 |
| >sp\|Q28894\|WFDC2_CANLF WAP four-disulfide core domain protein 2 OS=Canis lupus familiaris OX=9615 GN=WFDC2 PE=2 SV=1 | 4.54 | 4.38 | 467.20 | 50655447.3 | 9 | 1 | 0 | 6.45 | 124 | 53 |
| >tr\|A0A5F4D8I6\|A0A5F4D8I6_CANLF Phospholipase A2 receptor 1 OS=Canis lupus familiaris OX=9615 GN=PLA2R1 PE=4 SV=1 | 0.34 | 0.06 | 144.40 | 50230749.6 | 15 | 1 | 0 | 0.50 | 1394 | 6796 |
| >sp\|Q5JZQ9\|CLN5_CANLF Ceroid-lipofuscinosis neuronal protein 5 OS=Canis lupus familiaris OX=9615 GN=CLN5 PE=2 SV=1 | 0.90 | 0.86 | 254.10 | 49880822.2 | 3 | 1 | 0 | 1.14 | 350 | 651 |
| >tr\|A0A5F4CGE0\|A0A5F4CGE0_CANLF Ubiquitin protein ligase E3C OS=Canis lupus familiaris OX=9615 GN=UBE3C PE=4 SV=1 | 0.11 | 0.08 | 152.10 | 49667773.2 | 3 | 1 | 0 | 0.97 | 1238 | 9018 |

| | | | | | | | | | | |
|---|---|---|---|---|---|---|---|---|---|---|
| >sp\|P21842\|CMA1_CANLF Chymase OS=Canis lupus familiaris OX=9615 GN=CMA1 PE=1 SV=1 | 0.13 | 0.05 | 94.60 | 49378811.8 | 5 | 1 | 0 | 0.80 | 249 | 34 |
| >sp\|P62286\|ASPM_CANLF Abnormal spindle-like microcephaly-associated protein homolog OS=Canis lupus familiaris OX=9615 GN=ASPM PE=2 SV=2 | 1.91 | 1.04 | 121.50 | 48877259.4 | 7 | 3 | 0 | 0.23 | 3469 | 677 |
| >tr\|E2RE16\|E2RE16_CANLF Non-specific serine/threonine protein kinase OS=Canis lupus familiaris OX=9615 GN=PAK4 PE=4 SV=1 | 0.36 | 0.02 | 100.00 | 48233350.0 | 21 | 1 | 0 | 0.84 | 592 | 12735 |
| >tr\|A0A5F4BQW4\|A0A5F4BQW4_CANLF Zinc finger protein 654 OS=Canis lupus familiaris OX=9615 GN=ZNF654 PE=4 SV=1 | 0.17 | 0.07 | 166.70 | 48155193.5 | 6 | 1 | 1 | 1.03 | 1170 | 6910 |
| >tr\|E2R6E0\|E2R6E0_CANLF Lipocln_cytosolic_FA-bd_dom domain-containing protein OS=Canis lupus familiaris OX=9615 GN=LCNL1 PE=3 SV=2 | 3.20 | 3.14 | 187.20 | 48134410.4 | 4 | 1 | 0 | 3.01 | 299 | 1932 |
| >tr\|F1PJY1\|F1PJY1_CANLF Mannosyl-glycoprotein endo-beta-N-acetylglucosaminidase OS=Canis lupus familiaris OX=9615 GN=ENGASE PE=3 SV=3 | 1.35 | 1.16 | 161.90 | 47342666.3 | 9 | 2 | 1 | 1.74 | 690 | 32761 |
| >tr\|A0A5F4D7Y5\|A0A5F4D7Y5_CANLF Pleckstrin homology, MyTH4 and FERM domain containing H1 OS=Canis lupus familiaris OX=9615 GN=PLEKHH1 PE=4 SV=1 | 0.29 | 0.23 | 232.80 | 46821243.8 | 4 | 1 | 0 | 0.30 | 1342 | 5979 |
| >tr\|A0A5F4D7Y5\|A0A5F4D7Y5_CANLF Pleckstrin homology, MyTH4 and FERM domain containing H1 OS=Canis lupus familiaris OX=9615 GN=PLEKHH1 PE=4 SV=1 | 1.02 | 0.96 | 257.30 | 46707871.0 | 4 | 1 | 0 | 0.30 | 1342 | 5979 |
| >tr\|J9NW72\|J9NW72_CANLF Sperm associated antigen 8 OS=Canis lupus familiaris OX=9615 GN=SPAG8 PE=4 SV=1 | 0.20 | 0.04 | 114.10 | 46404625.9 | 9 | 1 | 0 | 4.07 | 442 | 10371 |
| >tr\|J9P432\|J9P432_CANLF Glutamine--fructose-6-phosphate transaminase (isomerizing) OS=Canis lupus familiaris OX=9615 GN=GFPT1 PE=4 SV=2 | 1.15 | 0.95 | 132.10 | 45591924.9 | 11 | 1 | 0 | 1.18 | 677 | 7191 |
| >sp\|P49822\|ALBU_CANLF Albumin OS=Canis lupus familiaris OX=9615 GN=ALB PE=1 SV=3 | 4.36 | 3.17 | 272.90 | 43519501.1 | 18 | 3 | 0 | 6.41 | 608 | 490 |
| >tr\|F1P6B8\|F1P6B8_CANLF Intraflagellar transport protein 57 homolog OS=Canis lupus familiaris OX=9615 GN=IFT57 PE=3 SV=3 | 0.26 | 0.22 | 124.60 | 43519116.3 | 3 | 1 | 0 | 0.83 | 482 | 4757 |
| >tr\|J9NS29\|J9NS29_CANLF Cystatin domain-containing protein OS=Canis lupus familiaris OX=9615 GN=LOC607874 PE=4 SV=2 | 0.79 | 0.75 | 178.80 | 43209619.1 | 3 | 1 | 0 | 6.39 | 313 | 30016 |
| >tr\|F1PLT8\|F1PLT8_CANLF Sulfhydryl oxidase OS=Canis lupus familiaris OX=9615 GN=QSOX1 PE=3 SV=3 | 1.82 | 1.74 | 132.70 | 42812978.9 | 5 | 1 | 0 | 3.35 | 568 | 33056 |
| >tr\|F1PRI9\|F1PRI9_CANLF Acyltransferase OS=Canis lupus familiaris OX=9615 GN=DGAT2 PE=3 SV=3 | 0.17 | 0.05 | 58.80 | 42206425.2 | 7 | 1 | 1 | 1.89 | 424 | 31675 |
| >tr\|A0A5F4C7E7\|A0A5F4C7E7_CANLF 3-beta-hydroxysterol Delta (14)-reductase OS=Canis lupus familiaris OX=9615 GN=LBR PE=3 SV=1 | 0.10 | 0.05 | 163.20 | 41982111.7 | 3 | 1 | 0 | 1.04 | 576 | 1034 |
| >tr\|A0A5F4D967\|A0A5F4D967_CANLF GLI family zinc finger 1 OS=Canis lupus familiaris OX=9615 GN=GLI1 PE=3 SV=1 | 0.13 | 0.01 | 159.90 | 41711241.5 | 12 | 1 | 0 | 0.70 | 1139 | 11817 |
| >tr\|F1PJY1\|F1PJY1_CANLF Mannosyl-glycoprotein endo-beta-N-acetylglucosaminidase OS=Canis lupus familiaris OX=9615 GN=ENGASE PE=3 SV=3 | 0.48 | 0.18 | 146.50 | 41325052.6 | 10 | 2 | 1 | 1.74 | 690 | 32761 |
| >sp\|P25473\|CLUS_CANLF Clusterin OS=Canis lupus familiaris OX=9615 GN=CLU PE=2 SV=1 | 0.29 | 0.27 | 235.00 | 39800627.0 | 2 | 1 | 0 | 0.90 | 445 | 725 |
| >tr\|A0A5F4CV50\|A0A5F4CV50_CANLF ETS transcription factor ELK4 OS=Canis lupus familiaris OX=9615 GN=ELK4 PE=3 SV=1 | 0.43 | 0.42 | 64.60 | 39553680.0 | 2 | 1 | 1 | 2.50 | 520 | 7938 |
| >tr\|F1PPP9\|F1PPP9_CANLF Family with sequence similarity 135 member A OS=Canis lupus familiaris OX=9615 GN=FAM135A PE=3 SV=3 | 0.21 | 0.09 | 163.00 | 39376421.0 | 8 | 1 | 0 | 1.22 | 1399 | 6815 |
| >tr\|E2R6E0\|E2R6E0_CANLF Lipocln_cytosolic_FA-bd_dom domain-containing protein OS=Canis lupus familiaris OX=9615 GN=LCNL1 PE=3 SV=2 | 3.33 | 3.29 | 212.90 | 39130593.5 | 3 | 1 | 0 | 3.01 | 299 | 1932 |

| | | | | | | | | | | |
|---|---|---|---|---|---|---|---|---|---|---|
| >tr\|A0A5F4CCD0\|A0A5F4CCD0_CANLF Cysteine rich secretory protein 2 OS=Canis lupus familiaris OX=9615 GN=CRISP2 PE=3 SV=1 | 1.27 | 1.21 | 231.00 | 39031198.1 | 4 | 1 | 0 | 2.25 | 311 | 11017 |
| >tr\|A0A5F4DI92\|A0A5F4DI92_CANLF DNA helicase OS=Canis lupus familiaris OX=9615 GN=CHD6 PE=3 SV=1 | 0.10 | 0.06 | 174.60 | 38589390.5 | 3 | 1 | 0 | 0.26 | 2685 | 3470 |
| >tr\|J9P6I3\|J9P6I3_CANLF Chloride intracellular channel protein OS=Canis lupus familiaris OX=9615 GN=CLIC2 PE=3 SV=1 | 0.10 | 0.00 | 170.60 | 38559607.2 | 2 | 1 | 0 | 2.93 | 239 | 40482 |
| >sp\|P62286\|ASPM_CANLF Abnormal spindle-like microcephaly-associated protein homolog OS=Canis lupus familiaris OX=9615 GN=ASPM PE=2 SV=2 | 0.29 | 0.10 | 43.30 | 38400604.6 | 10 | 2 | 0 | 0.23 | 3469 | 677 |
| >tr\|F1PS54\|F1PS54_CANLF MFS domain-containing protein OS=Canis lupus familiaris OX=9615 GN=SLC17A3 PE=4 SV=3 | 0.10 | 0.01 | 42.50 | 38123286.0 | 2 | 1 | 1 | 7.71 | 493 | 988 |
| >tr\|A0A5F4D6L9\|A0A5F4D6L9_CANLF Sacsin molecular chaperone OS=Canis lupus familiaris OX=9615 GN=SACS PE=4 SV=1 | 0.84 | 0.78 | 236.00 | 37550451.5 | 5 | 2 | 0 | 0.18 | 4500 | 1444 |
| >sp\|P25473\|CLUS_CANLF Clusterin OS=Canis lupus familiaris OX=9615 GN=CLU PE=2 SV=1 | 1.71 | 1.69 | 265.40 | 37186402.5 | 2 | 1 | 0 | 0.90 | 445 | 725 |
| >tr\|E2RDH6\|E2RDH6_CANLF Cytoplasmic dynein 2 heavy chain 1 OS=Canis lupus familiaris OX=9615 GN=DYNC2H1 PE=3 SV=3 | 0.16 | 0.03 | 149.60 | 37149940.3 | 10 | 2 | 1 | 0.24 | 4253 | 25056 |
| >tr\|A0A5F4BT89\|A0A5F4BT89_CANLF Olfactory receptor OS=Canis lupus familiaris OX=9615 GN=OR5W6 PE=3 SV=1 | 0.10 | 0.00 | 106.40 | 37127417.2 | 2 | 1 | 1 | 6.95 | 302 | 29923 |
| >tr\|A0A5F4D6L9\|A0A5F4D6L9_CANLF Sacsin molecular chaperone OS=Canis lupus familiaris OX=9615 GN=SACS PE=4 SV=1 | 1.27 | 1.22 | 231.30 | 36901438.8 | 8 | 2 | 0 | 0.18 | 4500 | 1444 |
| >tr\|J9P6I3\|J9P6I3_CANLF Chloride intracellular channel protein OS=Canis lupus familiaris OX=9615 GN=CLIC2 PE=3 SV=1 | 0.10 | 0.00 | 166.30 | 36388172.7 | 2 | 1 | 0 | 2.93 | 239 | 40482 |
| >tr\|A0A5F4DCZ9\|A0A5F4DCZ9_CANLF Kinase D interacting substrate 220 OS=Canis lupus familiaris OX=9615 GN=KIDINS220 PE=4 SV=1 | 0.10 | 0.08 | 140.50 | 36026128.8 | 2 | 1 | 0 | 0.60 | 1678 | 1923 |
| >tr\|F1PPP9\|F1PPP9_CANLF Family with sequence similarity 135 member A OS=Canis lupus familiaris OX=9615 GN=FAM135A PE=3 SV=3 | 1.30 | 0.94 | 141.60 | 35789354.8 | 19 | 1 | 0 | 1.22 | 1399 | 6815 |
| >tr\|J9P432\|J9P432_CANLF Glutamine--fructose-6-phosphate transaminase (isomerizing) OS=Canis lupus familiaris OX=9615 GN=GFPT1 PE=4 SV=2 | 1.13 | 1.01 | 126.60 | 35475474.3 | 7 | 1 | 0 | 1.18 | 677 | 7191 |
| >tr\|J9NS28\|J9NS28_CANLF RBR-type E3 ubiquitin transferase OS=Canis lupus familiaris OX=9615 GN=ANKIB1 PE=4 SV=2 | 0.18 | 0.04 | 34.90 | 35114008.7 | 8 | 1 | 0 | 2.33 | 988 | 26345 |
| >sp\|Q2PQH8\|GDE_CANLF Glycogen debranching enzyme OS=Canis lupus familiaris OX=9615 GN=AGL PE=2 SV=1 | 0.30 | 0.28 | 67.40 | 35106817.3 | 2 | 1 | 0 | 0.13 | 1533 | 23 |
| >tr\|F1PLT8\|F1PLT8_CANLF Sulfhydryl oxidase OS=Canis lupus familiaris OX=9615 GN=QSOX1 PE=3 SV=3 | 0.21 | 0.19 | 105.90 | 34725115.1 | 2 | 1 | 0 | 2.46 | 568 | 33056 |
| >tr\|A0A5F4D7Y5\|A0A5F4D7Y5_CANLF Pleckstrin homology, MyTH4 and FERM domain containing H1 OS=Canis lupus familiaris OX=9615 GN=PLEKHH1 PE=4 SV=1 | 0.82 | 0.78 | 234.90 | 34468734.1 | 3 | 1 | 0 | 0.30 | 1342 | 5979 |
| >tr\|F1PEN8\|F1PEN8_CANLF Carboxylic ester hydrolase OS=Canis lupus familiaris OX=9615 GN=BCHE PE=3 SV=1 | 0.44 | 0.40 | 168.50 | 34444629.9 | 3 | 1 | 0 | 1.33 | 602 | 4179 |
| >sp\|Q28894\|WFDC2_CANLF WAP four-disulfide core domain protein 2 OS=Canis lupus familiaris OX=9615 GN=WFDC2 PE=2 SV=1 | 2.83 | 2.47 | 371.40 | 34328147.1 | 19 | 1 | 0 | 6.45 | 124 | 53 |
| >sp\|Q9GL25\|ESPB1_CANLF Epididymal sperm-binding protein 1 OS=Canis lupus familiaris OX=9615 GN=ELSPBP1 PE=1 SV=1 | 2.32 | 2.28 | 236.50 | 34317958.4 | 3 | 1 | 0 | 6.94 | 245 | 36 |
| >sp\|Q5QQ50\|XYLT2_CANLF Xylosyltransferase 2 OS=Canis lupus familiaris OX=9615 GN=XYLT2 PE=2 SV=1 | 0.26 | 0.24 | 163.50 | 34088881.2 | 2 | 1 | 0 | 0.46 | 865 | 388 |

| | | | | | | | | | | |
|---|---|---|---|---|---|---|---|---|---|---|
| >tr\|J9NZH4\|J9NZH4_CANLF NTR domain-containing protein OS=Canis lupus familiaris OX=9615 GN=LOC102154527 PE=3 SV=2 | 0.56 | 0.48 | 90.30 | 33988855.0 | 3 | 2 | 0 | 16.74 | 221 | 39505 |
| >tr\|F1PS54\|F1PS54_CANLF MFS domain-containing protein OS=Canis lupus familiaris OX=9615 GN=SLC17A3 PE=4 SV=3 | 0.90 | 0.88 | 36.50 | 33351418.1 | 2 | 1 | 1 | 7.71 | 493 | 988 |
| >tr\|A0A5F4D7Y5\|A0A5F4D7Y5_CANLF Pleckstrin homology, MyTH4 and FERM domain containing H1 OS=Canis lupus familiaris OX=9615 GN=PLEKHH1 PE=4 SV=1 | 0.36 | 0.32 | 242.10 | 33189008.8 | 3 | 1 | 0 | 0.30 | 1342 | 5979 |
| >sp\|P25473\|CLUS_CANLF Clusterin OS=Canis lupus familiaris OX=9615 GN=CLU PE=2 SV=1 | 0.15 | 0.07 | 160.30 | 33136343.5 | 5 | 1 | 0 | 2.25 | 445 | 725 |
| >sp\|Q5JZQ9\|CLN5_CANLF Ceroid-lipofuscinosis neuronal protein 5 OS=Canis lupus familiaris OX=9615 GN=CLN5 PE=2 SV=1 | 1.07 | 1.05 | 238.30 | 32945370.0 | 2 | 1 | 0 | 1.14 | 350 | 651 |
| >tr\|F1P8J6\|F1P8J6_CANLF RNA helicase OS=Canis lupus familiaris OX=9615 GN=DDX55 PE=3 SV=3 | 0.10 | 0.00 | 123.50 | 32802576.2 | 2 | 1 | 0 | 1.58 | 568 | 8934 |
| >tr\|A0A5F4CLI1\|A0A5F4CLI1_CANLF Histone deacetylase 6 OS=Canis lupus familiaris OX=9615 GN=HDAC6 PE=4 SV=1 | 0.10 | 0.00 | 149.30 | 32754040.1 | 2 | 1 | 0 | 0.43 | 1175 | 4057 |
| >sp\|Q9GL25\|ESPB1_CANLF Epididymal sperm-binding protein 1 OS=Canis lupus familiaris OX=9615 GN=ELSPBP1 PE=1 SV=1 | 2.60 | 2.45 | 212.30 | 32590498.3 | 3 | 2 | 0 | 11.43 | 245 | 36 |
| >tr\|A0A5F4CU58\|A0A5F4CU58_CANLF Bromodomain containing 9 OS=Canis lupus familiaris OX=9615 GN=BRD9 PE=4 SV=1 | 0.24 | 0.20 | 152.30 | 32426016.6 | 3 | 1 | 0 | 1.10 | 544 | 17873 |
| >tr\|J9P3V5\|J9P3V5_CANLF FAM75 domain-containing protein OS=Canis lupus familiaris OX=9615 PE=4 SV=2 | 0.39 | 0.35 | 134.80 | 32364420.9 | 3 | 1 | 0 | 0.29 | 1370 | 32063 |
| >sp\|Q8WMX5\|S15A1_CANLF Solute carrier family 15 member 1 OS=Canis lupus familiaris OX=9615 GN=SLC15A1 PE=2 SV=2 | 1.31 | 1.26 | 105.20 | 32137387.6 | 3 | 2 | 1 | 1.55 | 708 | 5 |
| >sp\|Q9TU69\|GHR_CANLF Growth hormone receptor OS=Canis lupus familiaris OX=9615 GN=GHR PE=2 SV=1 | 0.10 | 0.04 | 138.30 | 31930926.0 | 2 | 1 | 0 | 1.72 | 638 | 541 |
| >tr\|A0A5F4CGE0\|A0A5F4CGE0_CANLF Ubiquitin protein ligase E3C OS=Canis lupus familiaris OX=9615 GN=UBE3C PE=4 SV=1 | 0.51 | 0.49 | 136.40 | 31871244.2 | 2 | 1 | 0 | 0.97 | 1238 | 9018 |
| >tr\|F1PJY1\|F1PJY1_CANLF Mannosyl-glycoprotein endo-beta-N-acetylglucosaminidase OS=Canis lupus familiaris OX=9615 GN=ENGASE PE=3 SV=3 | 0.62 | 0.52 | 156.90 | 31628699.7 | 5 | 2 | 1 | 1.74 | 690 | 32761 |
| >tr\|A0A5F4BZW4\|A0A5F4BZW4_CANLF Malonyl-CoA decarboxylase OS=Canis lupus familiaris OX=9615 GN=MLYCD PE=4 SV=1 | 0.10 | 0.02 | 174.60 | 31434672.5 | 2 | 1 | 0 | 1.30 | 461 | 4809 |
| >tr\|A0A5F4CCD0\|A0A5F4CCD0_CANLF Cysteine rich secretory protein 2 OS=Canis lupus familiaris OX=9615 GN=CRISP2 PE=3 SV=1 | 0.13 | 0.10 | 229.70 | 30618312.8 | 3 | 1 | 0 | 2.25 | 311 | 11017 |
| >tr\|A0A5F4BP79\|A0A5F4BP79_CANLF Transformation/transcription domain associated protein OS=Canis lupus familiaris OX=9615 GN=TRRAP PE=4 SV=1 | 0.77 | 0.50 | 186.00 | 30589768.9 | 4 | 2 | 0 | 0.37 | 3783 | 1628 |
| >tr\|J9P9K7\|J9P9K7_CANLF Glycylpeptide N-tetradecanoyltransferase OS=Canis lupus familiaris OX=9615 GN=NMT2 PE=3 SV=2 | 0.23 | 0.21 | 45.90 | 30071689.7 | 2 | 1 | 0 | 3.16 | 507 | 19368 |
| >sp\|P21842\|CMA1_CANLF Chymase OS=Canis lupus familiaris OX=9615 GN=CMA1 PE=1 SV=1 | 0.45 | 0.39 | 107.10 | 29552753.0 | 4 | 1 | 0 | 0.80 | 249 | 34 |
| >tr\|E2RCT1\|E2RCT1_CANLF WAP domain-containing protein OS=Canis lupus familiaris OX=9615 PE=4 SV=2 | 3.42 | 1.94 | 256.80 | 29539003.9 | 8 | 2 | 0 | 14.66 | 116 | 21717 |
| >tr\|F1PLT8\|F1PLT8_CANLF Sulfhydryl oxidase OS=Canis lupus familiaris OX=9615 GN=QSOX1 PE=3 SV=3 | 2.19 | 2.13 | 143.20 | 29520274.8 | 4 | 1 | 0 | 3.35 | 568 | 33056 |
| >tr\|J9NS28\|J9NS28_CANLF RBR-type E3 ubiquitin transferase OS=Canis lupus familiaris OX=9615 GN=ANKIB1 PE=4 SV=2 | 0.40 | 0.38 | 36.50 | 29488155.4 | 2 | 1 | 0 | 2.33 | 988 | 26345 |

| | | | | | | | | | | |
|---|---|---|---|---|---|---|---|---|---|---|
| >sp\|Q9TU69\|GHR_CANLF Growth hormone receptor OS=Canis lupus familiaris OX=9615 GN=GHR PE=2 SV=1 | 0.15 | 0.13 | 118.50 | 29480740.7 | 2 | 1 | 0 | 1.72 | 638 | 541 |
| >tr\|A0A5F4D9S5\|A0A5F4D9S5_CANLF Hyaluronoglucosaminidase OS=Canis lupus familiaris OX=9615 GN=CEMIP PE=3 SV=1 | 0.88 | 0.86 | 129.00 | 29240984.6 | 2 | 1 | 0 | 0.24 | 1684 | 9775 |
| >tr\|A0A5F4D9S5\|A0A5F4D9S5_CANLF Hyaluronoglucosaminidase OS=Canis lupus familiaris OX=9615 GN=CEMIP PE=3 SV=1 | 0.39 | 0.37 | 132.10 | 29055546.1 | 2 | 1 | 0 | 0.24 | 1684 | 9775 |
| >tr\|A0A5F4BZW4\|A0A5F4BZW4_CANLF Malonyl-CoA decarboxylase OS=Canis lupus familiaris OX=9615 GN=MLYCD PE=4 SV=1 | 0.10 | 0.08 | 189.40 | 29018094.1 | 2 | 1 | 0 | 1.30 | 461 | 4809 |
| >tr\|E2RRF5\|E2RRF5_CANLF RNA binding motif protein 19 OS=Canis lupus familiaris OX=9615 GN=RBM19 PE=4 SV=3 | 0.10 | 0.00 | 45.30 | 28927092.0 | 4 | 1 | 0 | 0.31 | 970 | 905 |
| >sp\|P52212\|PTHY_CANLF Parathyroid hormone OS=Canis lupus familiaris OX=9615 GN=PTH PE=3 SV=1 | 0.10 | 0.00 | 25.40 | 28598170.4 | 11 | 1 | 0 | 3.48 | 115 | 263 |
| >tr\|A0A5F4D6G2\|A0A5F4D6G2_CANLF SMG7 nonsense mediated mRNA decay factor OS=Canis lupus familiaris OX=9615 GN=SMG7 PE=4 SV=1 | 0.42 | 0.38 | 230.10 | 28574753.1 | 3 | 1 | 0 | 0.43 | 1175 | 2075 |
| >sp\|Q28895\|NPC2_CANLF NPC intracellular cholesterol transporter 2 OS=Canis lupus familiaris OX=9615 GN=NPC2 PE=2 SV=1 | 4.13 | 2.09 | 398.80 | 28420166.8 | 13 | 2 | 0 | 14.77 | 149 | 153 |
| >tr\|J9P3D0\|J9P3D0_CANLF Solute carrier family 4 member 9 OS=Canis lupus familiaris OX=9615 GN=SLC4A9 PE=3 SV=2 | 0.46 | 0.40 | 123.70 | 28122441.8 | 4 | 1 | 0 | 1.12 | 893 | 31921 |
| >tr\|A0A5F4CCD0\|A0A5F4CCD0_CANLF Cysteine rich secretory protein 2 OS=Canis lupus familiaris OX=9615 GN=CRISP2 PE=3 SV=1 | 1.56 | 1.54 | 244.00 | 27758081.1 | 2 | 1 | 0 | 2.25 | 311 | 11017 |
| >tr\|J9P3D0\|J9P3D0_CANLF Solute carrier family 4 member 9 OS=Canis lupus familiaris OX=9615 GN=SLC4A9 PE=3 SV=2 | 1.59 | 1.55 | 90.50 | 27627526.3 | 3 | 1 | 0 | 1.12 | 893 | 31921 |
| >tr\|A0A5F4CXX5\|A0A5F4CXX5_CANLF Carboxypeptidase OS=Canis lupus familiaris OX=9615 GN=CTSA PE=3 SV=1 | 0.11 | 0.09 | 173.80 | 27559167.3 | 2 | 1 | 0 | 2.27 | 485 | 3309 |
| >tr\|F1P6B8\|F1P6B8_CANLF Intraflagellar transport protein 57 homolog OS=Canis lupus familiaris OX=9615 GN=IFT57 PE=3 SV=3 | 0.44 | 0.19 | 116.30 | 27516919.1 | 7 | 2 | 0 | 1.45 | 482 | 4757 |
| >tr\|A0A5F4DGF5\|A0A5F4DGF5_CANLF Alkaline phosphatase OS=Canis lupus familiaris OX=9615 GN=ALPL PE=3 SV=1 | 1.25 | 0.99 | 233.90 | 27462453.1 | 3 | 3 | 0 | 5.77 | 572 | 6357 |
| >tr\|E2RB89\|E2RB89_CANLF Chloride channel accessory 2 OS=Canis lupus familiaris OX=9615 GN=CLCA2 PE=3 SV=2 | 0.15 | 0.09 | 62.70 | 27385999.8 | 4 | 1 | 0 | 1.47 | 951 | 24494 |
| >tr\|F1PPP9\|F1PPP9_CANLF Family with sequence similarity 135 member A OS=Canis lupus familiaris OX=9615 GN=FAM135A PE=3 SV=3 | 0.10 | 0.07 | 145.10 | 27171210.3 | 9 | 1 | 0 | 1.22 | 1399 | 6815 |
| >sp\|P48831\|ZP3_CANLF Zona pellucida sperm-binding protein 3 OS=Canis lupus familiaris OX=9615 GN=ZP3 PE=2 SV=1 | 0.30 | 0.28 | 142.70 | 27165280.4 | 2 | 1 | 0 | 0.70 | 426 | 21 |
| >tr\|E2R6E0\|E2R6E0_CANLF Lipocln_cytosolic_FA-bd_dom domain-containing protein OS=Canis lupus familiaris OX=9615 GN=LCNL1 PE=3 SV=2 | 1.68 | 1.46 | 162.70 | 27033829.4 | 6 | 2 | 0 | 6.35 | 299 | 1932 |
| >tr\|E2RHE2\|E2RHE2_CANLF GTPase, IMAP family member 1 OS=Canis lupus familiaris OX=9615 GN=GIMAP1 PE=3 SV=2 | 0.10 | 0.03 | 19.90 | 26838621.6 | 4 | 2 | 2 | 6.78 | 295 | 36146 |
| >tr\|E2RG75\|E2RG75_CANLF Inactive ribonuclease-like protein 9 OS=Canis lupus familiaris OX=9615 GN=RNASE9 PE=3 SV=2 | 3.00 | 2.85 | 409.50 | 26682678.7 | 8 | 2 | 1 | 8.08 | 198 | 41734 |
| >tr\|E2RBM0\|E2RBM0_CANLF Cleavage and polyadenylation specific factor 4 OS=Canis lupus familiaris OX=9615 GN=CPSF4 PE=4 SV=3 | 0.98 | 0.90 | 91.60 | 26666997.0 | 5 | 1 | 0 | 2.69 | 260 | 1857 |
| >tr\|A0A5F4D6L9\|A0A5F4D6L9_CANLF Sacsin molecular chaperone OS=Canis lupus familiaris OX=9615 GN=SACS PE=4 SV=1 | 0.45 | 0.40 | 239.30 | 26648081.5 | 4 | 1 | 0 | 0.09 | 4500 | 1444 |

| | | | | | | | | | | |
|---|---|---|---|---|---|---|---|---|---|---|
| >tr\|A0A5F4D595\|A0A5F4D595_CANLF Zinc finger protein 483 OS=Canis lupus familiaris OX=9615 GN=ZNF483 PE=4 SV=1 | 0.33 | 0.33 | 70.40 | 26472339.2 | 1 | 1 | 0 | 3.60 | 750 | 3465 |
| >tr\|A0A5K1V0D8\|A0A5K1V0D8_CANLF Sulfatase 2 OS=Canis lupus familiaris OX=9615 GN=SULF2 PE=3 SV=1 | 0.67 | 0.61 | 201.30 | 26246759.4 | 4 | 1 | 0 | 0.35 | 859 | 1192 |
| >sp\|Q6F3J0\|NFKB1_CANLF Nuclear factor NF-kappa-B p105 subunit OS=Canis lupus familiaris OX=9615 GN=NFKB1 PE=2 SV=2 | 0.13 | 0.11 | 150.90 | 26230930.0 | 2 | 1 | 0 | 0.31 | 972 | 124 |
| >tr\|A0A5F4CCD0\|A0A5F4CCD0_CANLF Cysteine rich secretory protein 2 OS=Canis lupus familiaris OX=9615 GN=CRISP2 PE=3 SV=1 | 0.91 | 0.89 | 250.30 | 26101119.6 | 2 | 1 | 0 | 2.25 | 311 | 11017 |
| >tr\|A0A5F4CXX5\|A0A5F4CXX5_CANLF Carboxypeptidase OS=Canis lupus familiaris OX=9615 GN=CTSA PE=3 SV=1 | 0.59 | 0.57 | 156.90 | 25975261.7 | 2 | 1 | 0 | 2.27 | 485 | 3309 |
| >tr\|A0A5F4CGE0\|A0A5F4CGE0_CANLF Ubiquitin protein ligase E3C OS=Canis lupus familiaris OX=9615 GN=UBE3C PE=4 SV=1 | 0.23 | 0.21 | 111.80 | 25339567.3 | 2 | 1 | 0 | 0.97 | 1238 | 9018 |
| >tr\|A0A5F4D7Y5\|A0A5F4D7Y5_CANLF Pleckstrin homology, MyTH4 and FERM domain containing H1 OS=Canis lupus familiaris OX=9615 GN=PLEKHH1 PE=4 SV=1 | 0.25 | 0.23 | 194.00 | 25247667.4 | 2 | 1 | 0 | 0.30 | 1342 | 5979 |
| >sp\|P25473\|CLUS_CANLF Clusterin OS=Canis lupus familiaris OX=9615 GN=CLU PE=2 SV=1 | 0.10 | 0.00 | 145.30 | 25224754.0 | 3 | 1 | 0 | 2.25 | 445 | 725 |
| >tr\|J9NTK2\|J9NTK2_CANLF J domain-containing protein OS=Canis lupus familiaris OX=9615 GN=DNAJC12 PE=4 SV=2 | 0.41 | 0.39 | 177.00 | 25186793.4 | 2 | 1 | 0 | 4.72 | 106 | 2310 |
| >tr\|F1PGK9\|F1PGK9_CANLF ADAM metallopeptidase with thrombospondin type 1 motif 5 OS=Canis lupus familiaris OX=9615 GN=ADAMTS5 PE=4 SV=3 | 0.34 | 0.15 | 105.90 | 23566169.6 | 12 | 1 | 0 | 0.59 | 845 | 11956 |
| >sp\|E2RK33\|GATC_CANLF Glutamyl-tRNA(Gln) amidotransferase subunit C, mitochondrial OS=Canis lupus familiaris OX=9615 GN=GATC PE=3 SV=1 | 0.21 | 0.19 | 180.00 | 23485546.1 | 2 | 1 | 0 | 2.58 | 155 | 39 |
| >tr\|A0A5F4D7Y5\|A0A5F4D7Y5_CANLF Pleckstrin homology, MyTH4 and FERM domain containing H1 OS=Canis lupus familiaris OX=9615 GN=PLEKHH1 PE=4 SV=1 | 0.80 | 0.80 | 217.90 | 22514601.8 | 2 | 1 | 0 | 0.30 | 1342 | 5979 |
| >sp\|P21842\|CMA1_CANLF Chymase OS=Canis lupus familiaris OX=9615 GN=CMA1 PE=1 SV=1 | 0.95 | 0.91 | 100.00 | 22126849.0 | 3 | 1 | 0 | 0.80 | 249 | 34 |
| >sp\|P21842\|CMA1_CANLF Chymase OS=Canis lupus familiaris OX=9615 GN=CMA1 PE=1 SV=1 | 0.16 | 0.12 | 91.90 | 22061926.1 | 3 | 1 | 0 | 0.80 | 249 | 34 |
| >tr\|A0A5F4C9T8\|A0A5F4C9T8_CANLF Inositol polyphosphate-5-phosphatase F OS=Canis lupus familiaris OX=9615 GN=INPP5F PE=4 SV=1 | 0.20 | 0.18 | 28.00 | 21671971.6 | 2 | 1 | 0 | 3.55 | 761 | 6184 |
| >sp\|P62286\|ASPM_CANLF Abnormal spindle-like microcephaly-associated protein homolog OS=Canis lupus familiaris OX=9615 GN=ASPM PE=2 SV=2 | 1.80 | 0.91 | 107.10 | 21549214.0 | 3 | 2 | 0 | 0.17 | 3469 | 677 |
| >tr\|A0A5F4D6G2\|A0A5F4D6G2_CANLF SMG7 nonsense mediated mRNA decay factor OS=Canis lupus familiaris OX=9615 GN=SMG7 PE=4 SV=1 | 0.29 | 0.27 | 235.60 | 21537147.8 | 2 | 1 | 0 | 0.43 | 1175 | 2075 |
| >sp\|Q28895\|NPC2_CANLF NPC intracellular cholesterol transporter 2 OS=Canis lupus familiaris OX=9615 GN=NPC2 PE=2 SV=1 | 1.04 | 0.95 | 288.10 | 21278461.1 | 6 | 1 | 0 | 8.72 | 149 | 153 |
| >tr\|A0A5F4D8I6\|A0A5F4D8I6_CANLF Phospholipase A2 receptor 1 OS=Canis lupus familiaris OX=9615 GN=PLA2R1 PE=4 SV=1 | 0.71 | 0.62 | 146.00 | 21266479.1 | 6 | 1 | 0 | 0.50 | 1394 | 6796 |
| >tr\|F1PB65\|F1PB65_CANLF RAD54 like 2 OS=Canis lupus familiaris OX=9615 GN=RAD54L2 PE=3 SV=2 | 0.78 | 0.78 | 130.30 | 21151961.3 | 1 | 1 | 0 | 0.55 | 1467 | 11575 |
| >tr\|F1PI09\|F1PI09_CANLF Aldehyde oxidase OS=Canis lupus familiaris OX=9615 GN=AOX2 PE=3 SV=3 | 0.31 | 0.25 | 185.10 | 21142095.1 | 4 | 1 | 0 | 0.67 | 1347 | 21650 |
| >tr\|A0A5F4D9L5\|A0A5F4D9L5_CANLF Acid phosphatase 3 OS=Canis lupus familiaris OX=9615 GN=ACP3 PE=4 SV=1 | 1.23 | 1.17 | 148.20 | 21108323.5 | 4 | 1 | 1 | 4.25 | 353 | 972 |

| | | | | | | | | | | |
|---|---|---|---|---|---|---|---|---|---|---|
| >tr\|A0A5F4DHH0\|A0A5F4DHH0_CANLF ATP binding cassette subfamily A member 1 OS=Canis lupus familiaris OX=9615 GN=ABCA1 PE=4 SV=1 | 0.17 | 0.02 | 60.10 | 21091293.3 | 10 | 1 | 0 | 0.23 | 2175 | 3709 |
| >tr\|E2RQR2\|E2RQR2_CANLF Olfactory receptor family 13 subfamily P member 3 OS=Canis lupus familiaris OX=9615 GN=OR13P3 PE=4 SV=2 | 0.37 | 0.37 | 93.00 | 20957177.5 | 1 | 1 | 0 | 6.73 | 312 | 15310 |
| >tr\|A0A5K1V0D8\|A0A5K1V0D8_CANLF Sulfatase 2 OS=Canis lupus familiaris OX=9615 GN=SULF2 PE=3 SV=1 | 0.20 | 0.16 | 192.50 | 20823626.8 | 3 | 1 | 0 | 0.35 | 859 | 1192 |
| >tr\|A0A5F4C9V7\|A0A5F4C9V7_CANLF Roundabout guidance receptor 3 OS=Canis lupus familiaris OX=9615 GN=ROBO3 PE=4 SV=1 | 0.36 | 0.22 | 183.50 | 20693477.0 | 8 | 1 | 0 | 0.63 | 1421 | 3563 |
| >tr\|A0A5F4C0U6\|A0A5F4C0U6_CANLF MLLT1 super elongation complex subunit OS=Canis lupus familiaris OX=9615 GN=MLLT1 PE=4 SV=1 | 0.10 | 0.00 | 71.20 | 20456197.1 | 7 | 1 | 0 | 0.67 | 597 | 1602 |
| >sp\|Q8WN22\|PRKDC_CANLF DNA-dependent protein kinase catalytic subunit OS=Canis lupus familiaris OX=9615 GN=PRKDC PE=2 SV=1 | 1.60 | 1.58 | 107.40 | 20411930.6 | 2 | 1 | 0 | 0.05 | 4144 | 338 |
| >tr\|J9P7R3\|J9P7R3_CANLF Transient receptor potential cation channel subfamily C member 4 associated protein OS=Canis lupus familiaris OX=9615 GN=TRPC4AP PE=4 SV=2 | 0.21 | 0.15 | 134.70 | 19970238.9 | 5 | 1 | 1 | 1.22 | 735 | 19673 |
| >tr\|A0A5F4D595\|A0A5F4D595_CANLF Zinc finger protein 483 OS=Canis lupus familiaris OX=9615 GN=ZNF483 PE=4 SV=1 | 0.39 | 0.39 | 32.70 | 19496810.4 | 1 | 1 | 0 | 3.60 | 750 | 3465 |
| >tr\|F1PRN0\|F1PRN0_CANLF RRM domain-containing protein OS=Canis lupus familiaris OX=9615 GN=LOC492092 PE=4 SV=3 | 0.89 | 0.87 | 157.80 | 19480278.6 | 2 | 1 | 0 | 0.87 | 918 | 37977 |
| >sp\|Q28895\|NPC2_CANLF NPC intracellular cholesterol transporter 2 OS=Canis lupus familiaris OX=9615 GN=NPC2 PE=2 SV=1 | 0.70 | 0.64 | 172.60 | 19345909.6 | 4 | 1 | 0 | 15.44 | 149 | 153 |
| >tr\|J9P432\|J9P432_CANLF Glutamine--fructose-6-phosphate transaminase (isomerizing) OS=Canis lupus familiaris OX=9615 GN=GFPT1 PE=4 SV=2 | 0.34 | 0.28 | 126.80 | 18852000.2 | 4 | 1 | 0 | 1.18 | 677 | 7191 |
| >tr\|J9NYC7\|J9NYC7_CANLF Dynein axonemal heavy chain 12 OS=Canis lupus familiaris OX=9615 GN=DNAH12 PE=3 SV=1 | 0.92 | 0.92 | 168.10 | 18704884.2 | 1 | 1 | 0 | 0.33 | 3960 | 15992 |
| >tr\|J9NTX3\|J9NTX3_CANLF SERTA domain containing 4 OS=Canis lupus familiaris OX=9615 GN=SERTAD4 PE=4 SV=1 | 0.19 | 0.17 | 139.20 | 18564888.4 | 2 | 1 | 0 | 1.40 | 356 | 7323 |
| >tr\|F1PRN0\|F1PRN0_CANLF RRM domain-containing protein OS=Canis lupus familiaris OX=9615 GN=LOC492092 PE=4 SV=3 | 0.72 | 0.70 | 172.00 | 18551823.8 | 2 | 1 | 0 | 0.87 | 918 | 37977 |
| >tr\|A0A5F4DHB2\|A0A5F4DHB2_CANLF Nuclear FMR1 interacting protein 2 OS=Canis lupus familiaris OX=9615 GN=NUFIP2 PE=4 SV=1 | 0.14 | 0.12 | 77.00 | 18405584.9 | 2 | 1 | 1 | 0.97 | 721 | 8839 |
| >tr\|A0A5F4CS87\|A0A5F4CS87_CANLF STE20 related adaptor alpha OS=Canis lupus familiaris OX=9615 GN=STRADA PE=4 SV=1 | 0.41 | 0.41 | 166.90 | 17648561.6 | 1 | 1 | 0 | 2.08 | 336 | 14044 |
| >tr\|F1PIP2\|F1PIP2_CANLF Guanylate cyclase OS=Canis lupus familiaris OX=9615 PE=3 SV=3 | 0.25 | 0.25 | 83.50 | 17641837.7 | 1 | 1 | 0 | 0.83 | 1085 | 12638 |
| >tr\|A0A5F4CNP4\|A0A5F4CNP4_CANLF ADP ribosylation factor GTPase activating protein 3 OS=Canis lupus familiaris OX=9615 GN=ARFGAP3 PE=4 SV=1 | 0.29 | 0.27 | 186.00 | 17577143.1 | 2 | 1 | 0 | 1.12 | 714 | 964 |
| >tr\|A0A5F4D7J3\|A0A5F4D7J3_CANLF Non-specific serine/threonine protein kinase OS=Canis lupus familiaris OX=9615 GN=CDC42BPA PE=3 SV=1 | 2.57 | 2.55 | 250.20 | 17414356.9 | 2 | 1 | 0 | 0.22 | 1794 | 1069 |
| >tr\|J9P0B4\|J9P0B4_CANLF Tudor domain containing 15 OS=Canis lupus familiaris OX=9615 GN=TDRD15 PE=4 SV=2 | 1.16 | 0.91 | 190.80 | 17385108.9 | 4 | 2 | 0 | 1.85 | 2105 | 4188 |
| >sp\|Q8WN22\|PRKDC_CANLF DNA-dependent protein kinase catalytic subunit OS=Canis lupus familiaris OX=9615 GN=PRKDC PE=2 SV=1 | 2.48 | 2.46 | 98.30 | 17372010.5 | 2 | 1 | 0 | 0.05 | 4144 | 338 |
| >tr\|A0A5F4CLI1\|A0A5F4CLI1_CANLF Histone deacetylase 6 OS=Canis lupus familiaris OX=9615 GN=HDAC6 PE=4 SV=1 | 0.82 | 0.82 | 162.50 | 16899203.5 | 1 | 1 | 0 | 0.43 | 1175 | 4057 |

| | | | | | | | | | | |
|---|---|---|---|---|---|---|---|---|---|---|
| >tr\|A0A5F4CS87\|A0A5F4CS87_CANLF STE20 related adaptor alpha OS=Canis lupus familiaris OX=9615 GN=STRADA PE=4 SV=1 | 0.10 | 0.00 | 172.70 | 16743131.7 | 1 | 1 | 0 | 2.08 | 336 | 14044 |
| >tr\|A0A5F4D7J3\|A0A5F4D7J3_CANLF Non-specific serine/threonine protein kinase OS=Canis lupus familiaris OX=9615 GN=CDC42BPA PE=3 SV=1 | 0.82 | 0.80 | 217.80 | 16533068.0 | 2 | 1 | 0 | 0.22 | 1794 | 1069 |
| >tr\|E2R594\|E2R594_CANLF Ring finger protein 167 OS=Canis lupus familiaris OX=9615 GN=RNF167 PE=4 SV=3 | 0.10 | 0.00 | 108.20 | 16461454.3 | 1 | 1 | 1 | 19.23 | 130 | 16405 |
| >tr\|J9NYC7\|J9NYC7_CANLF Dynein axonemal heavy chain 12 OS=Canis lupus familiaris OX=9615 GN=DNAH12 PE=3 SV=1 | 0.10 | 0.04 | 119.90 | 16448646.9 | 1 | 1 | 0 | 0.33 | 3960 | 15992 |
| >tr\|A0A5F4DCA4\|A0A5F4DCA4_CANLF Reverse transcriptase domain-containing protein OS=Canis lupus familiaris OX=9615 PE=4 SV=1 | 0.41 | 0.39 | 159.50 | 16354722.1 | 2 | 1 | 0 | 0.31 | 978 | 860 |
| >tr\|A0A5F4CCE2\|A0A5F4CCE2_CANLF Pappalysin 2 OS=Canis lupus familiaris OX=9615 GN=PAPPA2 PE=3 SV=1 | 0.27 | 0.27 | 180.00 | 16348970.5 | 1 | 1 | 0 | 0.81 | 1722 | 20443 |
| >sp\|Q6AW47\|EST5A_CANLF Carboxylesterase 5A OS=Canis lupus familiaris OX=9615 GN=CES5A PE=2 SV=1 | 0.86 | 0.86 | 186.40 | 16101669.9 | 1 | 1 | 0 | 0.87 | 575 | 629 |
| >tr\|E2QWD0\|E2QWD0_CANLF CAP-Gly domain containing linker protein 1 OS=Canis lupus familiaris OX=9615 GN=CLIP1 PE=4 SV=3 | 0.74 | 0.74 | 143.80 | 16031700.5 | 1 | 1 | 0 | 0.86 | 1403 | 12547 |
| >tr\|A0A5F4BP40\|A0A5F4BP40_CANLF Mitogen-activated protein kinase kinase kinase 1 OS=Canis lupus familiaris OX=9615 GN=MAP3K1 PE=4 SV=1 | 0.21 | 0.21 | 45.60 | 15743730.6 | 1 | 1 | 1 | 1.38 | 1671 | 15991 |
| >tr\|A0A5F4DFX0\|A0A5F4DFX0_CANLF Exportin-T OS=Canis lupus familiaris OX=9615 GN=XPOT PE=3 SV=1 | 0.10 | 0.01 | 115.40 | 15713572.6 | 2 | 1 | 0 | 0.75 | 938 | 2329 |
| >tr\|A0A5F4DFX0\|A0A5F4DFX0_CANLF Exportin-T OS=Canis lupus familiaris OX=9615 GN=XPOT PE=3 SV=1 | 0.10 | 0.00 | 124.60 | 15703857.1 | 2 | 1 | 0 | 0.75 | 938 | 2329 |
| >tr\|A0A5F4DHW1\|A0A5F4DHW1_CANLF Pappalysin 2 OS=Canis lupus familiaris OX=9615 GN=PAPPA2 PE=4 SV=1 | 0.56 | 0.28 | 122.80 | 15694497.4 | 2 | 2 | 0 | 1.79 | 1680 | 22479 |
| >tr\|E2RN16\|E2RN16_CANLF Mitogen-activated protein kinase kinase kinase 2 OS=Canis lupus familiaris OX=9615 GN=MAP3K2 PE=4 SV=2 | 0.20 | 0.20 | 173.50 | 15468692.3 | 1 | 1 | 0 | 0.97 | 620 | 34325 |
| >tr\|A0A5F4CS87\|A0A5F4CS87_CANLF STE20 related adaptor alpha OS=Canis lupus familiaris OX=9615 GN=STRADA PE=4 SV=1 | 0.23 | 0.23 | 165.70 | 15426344.8 | 1 | 1 | 0 | 2.08 | 336 | 14044 |
| >tr\|F1PCU5\|F1PCU5_CANLF Methyltransf_11 domain-containing protein OS=Canis lupus familiaris OX=9615 GN=LOC480074 PE=4 SV=3 | 1.26 | 1.26 | 126.20 | 15398710.0 | 1 | 1 | 0 | 2.86 | 630 | 1484 |
| >tr\|J9NS28\|J9NS28_CANLF RBR-type E3 ubiquitin transferase OS=Canis lupus familiaris OX=9615 GN=ANKIB1 PE=4 SV=2 | 0.13 | 0.07 | 35.60 | 15164556.5 | 4 | 1 | 0 | 2.33 | 988 | 26345 |
| >tr\|F1PBB5\|F1PBB5_CANLF Dynein axonemal heavy chain 3 OS=Canis lupus familiaris OX=9615 GN=DNAH3 PE=3 SV=3 | 0.11 | 0.02 | 19.20 | 15138372.6 | 6 | 1 | 0 | 0.12 | 4054 | 1774 |
| >tr\|J9NS29\|J9NS29_CANLF Cystatin domain-containing protein OS=Canis lupus familiaris OX=9615 GN=LOC607874 PE=4 SV=2 | 0.81 | 0.81 | 183.90 | 15130133.6 | 1 | 1 | 0 | 6.39 | 313 | 30016 |
| >tr\|A0A5F4BZW4\|A0A5F4BZW4_CANLF Malonyl-CoA decarboxylase OS=Canis lupus familiaris OX=9615 GN=MLYCD PE=4 SV=1 | 0.10 | 0.00 | 162.60 | 15074670.8 | 1 | 1 | 0 | 1.30 | 461 | 4809 |
| >tr\|A0A5F4C4Q5\|A0A5F4C4Q5_CANLF ST6 N-acetylgalactosaminide alpha-2,6-sialyltransferase 1 OS=Canis lupus familiaris OX=9615 GN=ST6GALNAC1 PE=3 SV=1 | 0.29 | 0.29 | 63.40 | 15026002.7 | 1 | 1 | 0 | 1.76 | 683 | 1306 |
| >sp\|O46669\|SCNAA_CANLF Sodium channel protein type 10 subunit alpha OS=Canis lupus familiaris OX=9615 GN=SCN10A PE=2 SV=1 | 0.24 | 0.24 | 116.80 | 14939585.4 | 1 | 1 | 0 | 0.15 | 1962 | 90 |
| >tr\|E2RJS0\|E2RJS0_CANLF Adaptor related protein complex 4 subunit epsilon 1 OS=Canis lupus familiaris OX=9615 GN=AP4E1 PE=4 SV=3 | 0.22 | 0.22 | 122.20 | 14847484.3 | 1 | 1 | 0 | 0.53 | 1138 | 37586 |

| | | | | | | | | | | |
|---|---|---|---|---|---|---|---|---|---|---|
| >tr\|F1PQC9\|F1PQC9_CANLF Dynein regulatory complex protein 10 OS=Canis lupus familiaris OX=9615 GN=IQCD PE=3 SV=3 | 0.10 | 0.10 | 183.60 | 14574910.7 | 1 | 1 | 0 | 0.89 | 451 | 1682 |
| >tr\|F1PCU5\|F1PCU5_CANLF Methyltransf_11 domain-containing protein OS=Canis lupus familiaris OX=9615 GN=LOC480074 PE=4 SV=3 | 0.66 | 0.66 | 121.70 | 14555416.2 | 1 | 1 | 0 | 2.86 | 630 | 1484 |
| >tr\|E2RK34\|E2RK34_CANLF Cytochrome P450 family 2 subfamily S member 1 OS=Canis lupus familiaris OX=9615 GN=CYP2S1 PE=3 SV=3 | 0.18 | 0.18 | 124.60 | 14499180.0 | 1 | 1 | 0 | 1.35 | 669 | 7130 |
| >tr\|J9NZH4\|J9NZH4_CANLF NTR domain-containing protein OS=Canis lupus familiaris OX=9615 GN=LOC102154527 PE=3 SV=2 | 0.10 | 0.01 | 116.60 | 14493173.9 | 1 | 1 | 0 | 8.60 | 221 | 39505 |
| >tr\|A0A5F4C7P9\|A0A5F4C7P9_CANLF Beta-2-microglobulin OS=Canis lupus familiaris OX=9615 GN=B2M PE=4 SV=1 | 0.93 | 0.93 | 156.60 | 14403347.3 | 1 | 1 | 0 | 12.15 | 107 | 10611 |
| >tr\|F6XM35\|F6XM35_CANLF HECT-type E3 ubiquitin transferase OS=Canis lupus familiaris OX=9615 GN=HUWE1 PE=4 SV=2 | 0.30 | 0.31 | 114.20 | 14316125.0 | 1 | 1 | 0 | 0.25 | 4399 | 1763 |
| >tr\|J9P2K4\|J9P2K4_CANLF Transmembrane protein 117 OS=Canis lupus familiaris OX=9615 GN=TMEM117 PE=4 SV=2 | 0.25 | 0.25 | 86.90 | 14257134.3 | 1 | 1 | 0 | 2.56 | 508 | 14687 |
| >tr\|J9P9K7\|J9P9K7_CANLF Glycylpeptide N-tetradecanoyltransferase OS=Canis lupus familiaris OX=9615 GN=NMT2 PE=3 SV=2 | 0.45 | 0.45 | 43.70 | 14130899.2 | 1 | 1 | 0 | 3.16 | 507 | 19368 |
| >tr\|Q9XSV4\|Q9XSV4_CANLF CE10 protein OS=Canis lupus familiaris OX=9615 GN=ce10 PE=2 SV=1 | 0.58 | 0.42 | 225.00 | 14071262.5 | 7 | 2 | 0 | 9.09 | 110 | 41542 |
| >tr\|F1PAJ9\|F1PAJ9_CANLF Adenylate cyclase type 5 OS=Canis lupus familiaris OX=9615 GN=ADCY5 PE=3 SV=3 | 1.40 | 1.40 | 111.50 | 13972157.6 | 1 | 1 | 0 | 2.24 | 1027 | 18306 |
| >tr\|F1PFP5\|F1PFP5_CANLF Transporter OS=Canis lupus familiaris OX=9615 GN=LOC100856702 PE=3 SV=2 | 0.10 | 0.10 | 46.90 | 13952552.0 | 1 | 1 | 0 | 2.55 | 705 | 40807 |
| >tr\|E2QWZ4\|E2QWZ4_CANLF Solute carrier family 34 member 3 OS=Canis lupus familiaris OX=9615 GN=SLC34A3 PE=3 SV=3 | 0.18 | 0.18 | 99.70 | 13842171.6 | 1 | 1 | 0 | 2.35 | 255 | 26251 |
| >tr\|A0A5F4D967\|A0A5F4D967_CANLF GLI family zinc finger 1 OS=Canis lupus familiaris OX=9615 GN=GLI1 PE=3 SV=1 | 0.10 | 0.03 | 140.80 | 13768232.8 | 4 | 1 | 0 | 0.70 | 1139 | 11817 |
| >tr\|J9P366\|J9P366_CANLF Ganglioside induced differentiation associated protein 2 OS=Canis lupus familiaris OX=9615 GN=GDAP2 PE=3 SV=2 | 0.15 | 0.15 | 174.30 | 13718512.3 | 1 | 1 | 0 | 2.82 | 497 | 9162 |
| >sp\|E2RDZ6\|SIR5_CANLF NAD-dependent protein deacylase sirtuin-5, mitochondrial OS=Canis lupus familiaris OX=9615 GN=SIRT5 PE=3 SV=1 | 0.21 | 0.19 | 86.80 | 13537439.5 | 2 | 1 | 0 | 0.65 | 310 | 270 |
| >tr\|A0A5F4C7P9\|A0A5F4C7P9_CANLF Beta-2-microglobulin OS=Canis lupus familiaris OX=9615 GN=B2M PE=4 SV=1 | 0.95 | 0.95 | 152.70 | 13362339.2 | 1 | 1 | 0 | 12.15 | 107 | 10611 |
| >tr\|A0A5F4CUE8\|A0A5F4CUE8_CANLF Senataxin OS=Canis lupus familiaris OX=9615 GN=SETX PE=4 SV=1 | 0.27 | 0.25 | 123.70 | 13270568.7 | 2 | 1 | 0 | 0.26 | 2645 | 1796 |
| >tr\|J9NZH4\|J9NZH4_CANLF NTR domain-containing protein OS=Canis lupus familiaris OX=9615 GN=LOC102154527 PE=3 SV=2 | 0.10 | 0.04 | 104.10 | 13235937.8 | 1 | 1 | 0 | 8.60 | 221 | 39505 |
| >tr\|A0A5F4D952\|A0A5F4D952_CANLF FAT atypical cadherin 1 OS=Canis lupus familiaris OX=9615 GN=FAT1 PE=4 SV=1 | 0.29 | 0.30 | 150.90 | 13213103.5 | 1 | 1 | 0 | 0.26 | 4614 | 14224 |
| >tr\|F6PLX8\|F6PLX8_CANLF Beta-defensin OS=Canis lupus familiaris OX=9615 GN=DEFB118 PE=3 SV=1 | 0.47 | 0.45 | 87.30 | 12974492.3 | 2 | 1 | 0 | 8.77 | 171 | 34287 |
| >tr\|F1PLV2\|F1PLV2_CANLF Peptidyl-prolyl cis-trans isomerase OS=Canis lupus familiaris OX=9615 GN=CSNK1G1 PE=3 SV=3 | 0.15 | 0.15 | 162.50 | 12935344.0 | 1 | 1 | 0 | 5.35 | 243 | 4290 |
| >tr\|F1PPP9\|F1PPP9_CANLF Family with sequence similarity 135 member A OS=Canis lupus familiaris OX=9615 GN=FAM135A PE=3 SV=3 | 0.10 | 0.03 | 145.10 | 12729324.1 | 4 | 1 | 0 | 1.22 | 1399 | 6815 |

| | | | | | | | | | | |
|---|---|---|---|---|---|---|---|---|---|---|
| >tr\|J9P3H8\|J9P3H8_CANLF ATM interactor OS=Canis lupus familiaris OX=9615 GN=ATMIN PE=4 SV=2 | 0.13 | 0.02 | 56.20 | 12633306.2 | 7 | 1 | 0 | 0.58 | 863 | 882 |
| >sp\|E2RK33\|GATC_CANLF Glutamyl-tRNA(Gln) amidotransferase subunit C, mitochondrial OS=Canis lupus familiaris OX=9615 GN=GATC PE=3 SV=1 | 0.54 | 0.54 | 168.60 | 12410748.1 | 1 | 1 | 0 | 2.58 | 155 | 39 |
| >tr\|A0A5F4C1S8\|A0A5F4C1S8_CANLF E3 ubiquitin-protein ligase CBL OS=Canis lupus familiaris OX=9615 GN=CBL PE=4 SV=1 | 0.10 | 0.10 | 176.30 | 12232430.2 | 1 | 1 | 0 | 0.39 | 773 | 1308 |
| >tr\|A0A5F4D0U7\|A0A5F4D0U7_CANLF Structural maintenance of chromosomes flexible hinge domain containing 1 OS=Canis lupus familiaris OX=9615 GN=SMCHD1 PE=4 SV=1 | 0.14 | 0.14 | 51.80 | 12112787.3 | 1 | 1 | 1 | 0.62 | 3051 | 9634 |
| >tr\|A0A5F4DC96\|A0A5F4DC96_CANLF Coiled-coil domain containing 178 OS=Canis lupus familiaris OX=9615 GN=CCDC178 PE=4 SV=1 | 0.10 | 0.00 | 19.00 | 11898572.3 | 1 | 1 | 0 | 0.37 | 807 | 32421 |
| >tr\|E2QX33\|E2QX33_CANLF Coiled-coil and C2 domain containing 1A OS=Canis lupus familiaris OX=9615 GN=CC2D1A PE=3 SV=1 | 0.19 | 0.17 | 144.50 | 11795640.6 | 2 | 1 | 1 | 0.84 | 951 | 10961 |
| >tr\|A0A5F4BTW9\|A0A5F4BTW9_CANLF Adhesion G protein-coupled receptor L2 OS=Canis lupus familiaris OX=9615 GN=ADGRL2 PE=4 SV=1 | 0.32 | 0.32 | 144.80 | 11648843.7 | 1 | 1 | 0 | 0.47 | 1474 | 2238 |
| >tr\|F1PJY1\|F1PJY1_CANLF Mannosyl-glycoprotein endo-beta-N-acetylglucosaminidase OS=Canis lupus familiaris OX=9615 GN=ENGASE PE=3 SV=3 | 0.10 | 0.00 | 183.00 | 11493761.4 | 5 | 2 | 2 | 1.74 | 690 | 32761 |
| >tr\|J9P3R7\|J9P3R7_CANLF SCO-spondin OS=Canis lupus familiaris OX=9615 GN=SSPO PE=3 SV=2 | 0.10 | 0.00 | 126.10 | 11392323.2 | 5 | 1 | 1 | 0.23 | 5112 | 24898 |
| >tr\|A0A5F4BU36\|A0A5F4BU36_CANLF Titin OS=Canis lupus familiaris OX=9615 GN=TTN PE=3 SV=1 | 0.15 | 0.15 | 107.90 | 11162151.6 | 2 | 1 | 0 | 0.06 | 27097 | 33785 |
| >tr\|A0A5F4C9F7\|A0A5F4C9F7_CANLF Activating signal cointegrator 1 complex subunit 3 OS=Canis lupus familiaris OX=9615 GN=ASCC3 PE=3 SV=1 | 0.21 | 0.21 | 199.70 | 11045232.0 | 1 | 1 | 0 | 0.60 | 2004 | 5494 |
| >tr\|E2R079\|E2R079_CANLF Serpin family B member 2 OS=Canis lupus familiaris OX=9615 GN=SERPINB2 PE=3 SV=2 | 0.33 | 0.31 | 87.60 | 10936664.5 | 2 | 1 | 0 | 1.92 | 416 | 21385 |
| >tr\|F1P6B8\|F1P6B8_CANLF Intraflagellar transport protein 57 homolog OS=Canis lupus familiaris OX=9615 GN=IFT57 PE=3 SV=3 | 0.24 | 0.24 | 229.70 | 10821822.7 | 1 | 1 | 0 | 0.83 | 482 | 4757 |
| >tr\|A0A5F4DKE8\|A0A5F4DKE8_CANLF Suppression of tumorigenicity 7 like OS=Canis lupus familiaris OX=9615 GN=ST7L PE=3 SV=1 | 0.18 | 0.18 | 133.40 | 10820650.2 | 1 | 1 | 0 | 0.78 | 644 | 5269 |
| >tr\|H9GWY3\|H9GWY3_CANLF Inter-alpha-trypsin inhibitor heavy chain 4 OS=Canis lupus familiaris OX=9615 GN=ITIH4 PE=3 SV=3 | 1.09 | 1.07 | 294.40 | 10490136.4 | 2 | 1 | 0 | 0.54 | 925 | 1304 |
| >tr\|A0A5F4BQA2\|A0A5F4BQA2_CANLF Dermatan sulfate epimerase OS=Canis lupus familiaris OX=9615 GN=DSE PE=3 SV=1 | 0.10 | 0.00 | 84.90 | 10466782.3 | 1 | 1 | 0 | 2.60 | 308 | 8924 |
| >tr\|F1PGK9\|F1PGK9_CANLF ADAM metallopeptidase with thrombospondin type 1 motif 5 OS=Canis lupus familiaris OX=9615 GN=ADAMTS5 PE=4 SV=3 | 0.10 | 0.02 | 32.80 | 10448788.8 | 5 | 1 | 0 | 0.59 | 845 | 11956 |
| >sp\|Q076A6\|MYH1_CANLF Myosin-1 OS=Canis lupus familiaris OX=9615 GN=MYH1 PE=3 SV=2 | 0.21 | 0.21 | 136.00 | 10211826.4 | 1 | 1 | 0 | 0.15 | 1939 | 121 |
| >tr\|F1Q030\|F1Q030_CANLF Ring finger protein 208 OS=Canis lupus familiaris OX=9615 GN=RNF208 PE=4 SV=3 | 0.17 | 0.18 | 30.00 | 9850180.4 | 1 | 1 | 1 | 9.81 | 265 | 27522 |
| >tr\|J9PB30\|J9PB30_CANLF THAP domain-containing protein 1 OS=Canis lupus familiaris OX=9615 GN=THAP1 PE=3 SV=2 | 0.14 | 0.14 | 72.80 | 8958205.0 | 1 | 1 | 1 | 2.44 | 246 | 11070 |
| >tr\|F1PFZ5\|F1PFZ5_CANLF Milk fat globule EGF and factor V/VIII domain containing OS=Canis lupus familiaris OX=9615 GN=MFGE8 PE=4 SV=3 | 0.46 | 0.46 | 194.00 | 8758445.0 | 1 | 1 | 0 | 1.87 | 428 | 7079 |
| >tr\|J9P7Y2\|J9P7Y2_CANLF Angiotensin-converting enzyme OS=Canis lupus familiaris OX=9615 GN=ACE2 PE=3 SV=1 | 0.15 | 0.15 | 42.60 | 8676308.0 | 2 | 1 | 0 | 1.62 | 804 | 1041 |

| | | | | | | | | | | |
|---|---|---|---|---|---|---|---|---|---|---|
| >tr\|E2QWB2\|E2QWB2_CANLF Plastin 1 OS=Canis lupus familiaris OX=9615 GN=PLS1 PE=4 SV=2 | 0.41 | 0.39 | 134.20 | 8639749.5 | 2 | 1 | 0 | 1.91 | 629 | 25458 |
| >tr\|J9P912\|J9P912_CANLF GRB10 interacting GYF protein 1 OS=Canis lupus familiaris OX=9615 GN=GIGYF1 PE=4 SV=2 | 0.30 | 0.30 | 5.70 | 8496941.6 | 1 | 1 | 0 | 4.39 | 683 | 18238 |
| >tr\|E2RHG5\|E2RHG5_CANLF Nudix hydrolase 3 OS=Canis lupus familiaris OX=9615 GN=NUDT3 PE=4 SV=1 | 0.30 | 0.28 | 113.10 | 8358541.2 | 2 | 1 | 0 | 6.40 | 172 | 21894 |
| >tr\|E2RG75\|E2RG75_CANLF Inactive ribonuclease-like protein 9 OS=Canis lupus familiaris OX=9615 GN=RNASE9 PE=3 SV=2 | 2.28 | 2.20 | 387.10 | 8288276.3 | 5 | 1 | 1 | 4.55 | 198 | 41734 |
| >tr\|F1PFZ5\|F1PFZ5_CANLF Milk fat globule EGF and factor V/VIII domain containing OS=Canis lupus familiaris OX=9615 GN=MFGE8 PE=4 SV=3 | 0.21 | 0.21 | 193.10 | 8276897.2 | 1 | 1 | 0 | 1.87 | 428 | 7079 |
| >tr\|A0A5F4CMF9\|A0A5F4CMF9_CANLF Cilia and flagella associated protein 65 OS=Canis lupus familiaris OX=9615 GN=CFAP65 PE=4 SV=1 | 1.07 | 1.08 | 174.60 | 8276036.7 | 1 | 1 | 0 | 0.17 | 1789 | 4175 |
| >tr\|F1PBU5\|F1PBU5_CANLF Non-specific serine/threonine protein kinase OS=Canis lupus familiaris OX=9615 GN=SMG1 PE=3 SV=3 | 0.83 | 0.83 | 173.30 | 8268862.3 | 1 | 1 | 0 | 0.08 | 3634 | 6898 |
| >tr\|F1PJY1\|F1PJY1_CANLF Mannosyl-glycoprotein endo-beta-N-acetylglucosaminidase OS=Canis lupus familiaris OX=9615 GN=ENGASE PE=3 SV=3 | 1.08 | 0.96 | 178.40 | 8211977.1 | 6 | 2 | 1 | 1.74 | 690 | 32761 |
| >tr\|F1PBU5\|F1PBU5_CANLF Non-specific serine/threonine protein kinase OS=Canis lupus familiaris OX=9615 GN=SMG1 PE=3 SV=3 | 1.22 | 1.22 | 163.80 | 8188124.9 | 1 | 1 | 0 | 0.08 | 3634 | 6898 |
| >tr\|F1PHQ0\|F1PHQ0_CANLF Clathrin heavy chain OS=Canis lupus familiaris OX=9615 GN=CLTC PE=3 SV=3 | 0.10 | 0.10 | 117.90 | 8063892.1 | 1 | 1 | 1 | 0.65 | 1682 | 24646 |
| >sp\|Q5I2M8\|TLR9_CANLF Toll-like receptor 9 OS=Canis lupus familiaris OX=9615 GN=TLR9 PE=2 SV=1 | 0.23 | 0.21 | 133.70 | 8030072.0 | 2 | 1 | 0 | 1.16 | 1032 | 382 |
| >tr\|F1PQC9\|F1PQC9_CANLF Dynein regulatory complex protein 10 OS=Canis lupus familiaris OX=9615 GN=IQCD PE=3 SV=3 | 0.22 | 0.23 | 193.30 | 7995980.7 | 1 | 1 | 0 | 0.89 | 451 | 1682 |
| >tr\|F1P8K0\|F1P8K0_CANLF Extended synaptotagmin 2 OS=Canis lupus familiaris OX=9615 GN=ESYT2 PE=3 SV=3 | 0.17 | 0.17 | 85.20 | 7967931.3 | 1 | 1 | 0 | 1.74 | 804 | 19801 |
| >tr\|E2RG75\|E2RG75_CANLF Inactive ribonuclease-like protein 9 OS=Canis lupus familiaris OX=9615 GN=RNASE9 PE=3 SV=2 | 0.92 | 0.88 | 399.90 | 7883394.3 | 3 | 1 | 1 | 4.55 | 198 | 41734 |
| >tr\|E2RN56\|E2RN56_CANLF Zinc finger CCCH-type containing 13 OS=Canis lupus familiaris OX=9615 GN=ZC3H13 PE=4 SV=3 | 1.22 | 1.22 | 220.00 | 7605712.3 | 1 | 1 | 0 | 0.24 | 1660 | 1446 |
| >tr\|F1Q4I7\|F1Q4I7_CANLF Beta-1,4-N-acetylgalactosaminyltransferase OS=Canis lupus familiaris OX=9615 GN=B4GALNT3 PE=3 SV=3 | 0.21 | 0.21 | 232.10 | 7598279.6 | 1 | 1 | 0 | 0.40 | 1003 | 6004 |
| >tr\|A0A5F4DCA4\|A0A5F4DCA4_CANLF Reverse transcriptase domain-containing protein OS=Canis lupus familiaris OX=9615 PE=4 SV=1 | 1.22 | 1.22 | 155.50 | 7526476.4 | 1 | 1 | 0 | 0.31 | 978 | 860 |
| >tr\|A0A5F4DCA4\|A0A5F4DCA4_CANLF Reverse transcriptase domain-containing protein OS=Canis lupus familiaris OX=9615 PE=4 SV=1 | 0.10 | 0.02 | 159.50 | 7392861.2 | 1 | 1 | 0 | 0.31 | 978 | 860 |
| >tr\|A0A5F4C9V7\|A0A5F4C9V7_CANLF Roundabout guidance receptor 3 OS=Canis lupus familiaris OX=9615 GN=ROBO3 PE=4 SV=1 | 0.31 | 0.31 | 144.50 | 7349836.4 | 1 | 1 | 0 | 0.63 | 1421 | 3563 |
| >tr\|E2RPU3\|E2RPU3_CANLF Ubiquitination factor E4A OS=Canis lupus familiaris OX=9615 GN=UBE4A PE=3 SV=2 | 0.21 | 0.19 | 70.70 | 7331525.3 | 2 | 1 | 0 | 1.28 | 1094 | 4207 |
| >tr\|A0A5F4D7Y5\|A0A5F4D7Y5_CANLF Pleckstrin homology, MyTH4 and FERM domain containing H1 OS=Canis lupus familiaris OX=9615 GN=PLEKHH1 PE=4 SV=1 | 0.26 | 0.22 | 86.50 | 7286984.7 | 3 | 1 | 0 | 0.30 | 1342 | 5979 |
| >tr\|E2RG75\|E2RG75_CANLF Inactive ribonuclease-like protein 9 OS=Canis lupus familiaris OX=9615 GN=RNASE9 PE=3 SV=2 | 0.89 | 0.85 | 397.80 | 7231364.2 | 3 | 1 | 1 | 4.55 | 198 | 41734 |

| | | | | | | | | | | |
|---|---|---|---|---|---|---|---|---|---|---|
| >tr\|E2RF02\|E2RF02_CANLF Ubiquitin protein ligase E3 component n-recognin 4 OS=Canis lupus familiaris OX=9615 GN=UBR4 PE=3 SV=2 | 0.16 | 0.15 | 113.10 | 7013691.8 | 2 | 1 | 0 | 0.19 | 5192 | 7776 |
| >tr\|J9P3H8\|J9P3H8_CANLF ATM interactor OS=Canis lupus familiaris OX=9615 GN=ATMIN PE=4 SV=2 | 0.22 | 0.15 | 39.10 | 6930331.9 | 5 | 1 | 0 | 0.58 | 863 | 882 |
| >tr\|J9NS29\|J9NS29_CANLF Cystatin domain-containing protein OS=Canis lupus familiaris OX=9615 GN=LOC607874 PE=4 SV=2 | 1.13 | 0.91 | 75.00 | 6825633.6 | 2 | 2 | 0 | 9.27 | 313 | 30016 |
| >sp\|Q75ZY9\|MET_CANLF Hepatocyte growth factor receptor OS=Canis lupus familiaris OX=9615 GN=MET PE=1 SV=1 | 0.39 | 0.39 | 157.00 | 6825360.6 | 1 | 1 | 0 | 0.22 | 1382 | 161 |
| >tr\|F1PPP9\|F1PPP9_CANLF Family with sequence similarity 135 member A OS=Canis lupus familiaris OX=9615 GN=FAM135A PE=3 SV=3 | 1.26 | 1.18 | 119.50 | 6795483.0 | 5 | 1 | 0 | 1.22 | 1399 | 6815 |
| >tr\|J9NSS6\|J9NSS6_CANLF DNA helicase OS=Canis lupus familiaris OX=9615 GN=CHD2 PE=4 SV=2 | 0.22 | 0.15 | 73.20 | 6784136.8 | 5 | 1 | 0 | 0.28 | 1780 | 1264 |
| >tr\|F1Q1J0\|F1Q1J0_CANLF DIX domain containing 1 OS=Canis lupus familiaris OX=9615 GN=DIXDC1 PE=4 SV=2 | 0.23 | 0.23 | 207.60 | 6680988.4 | 1 | 1 | 0 | 0.73 | 683 | 3850 |
| >sp\|Q28894\|WFDC2_CANLF WAP four-disulfide core domain protein 2 OS=Canis lupus familiaris OX=9615 GN=WFDC2 PE=2 SV=1 | 2.84 | 2.78 | 406.40 | 6491878.1 | 4 | 1 | 0 | 6.45 | 124 | 53 |
| >tr\|J9P2T7\|J9P2T7_CANLF 26S proteasome non-ATPase regulatory subunit 5 OS=Canis lupus familiaris OX=9615 PE=4 SV=1 | 1.54 | 1.54 | 116.00 | 5896750.8 | 1 | 1 | 0 | 1.95 | 461 | 23870 |
| >tr\|J9P432\|J9P432_CANLF Glutamine--fructose-6-phosphate transaminase (isomerizing) OS=Canis lupus familiaris OX=9615 GN=GFPT1 PE=4 SV=2 | 0.33 | 0.29 | 58.20 | 5884831.8 | 3 | 1 | 0 | 1.18 | 677 | 7191 |
| >tr\|E2RIV7\|E2RIV7_CANLF Syntrophin alpha 1 OS=Canis lupus familiaris OX=9615 GN=SNTA1 PE=3 SV=3 | 0.10 | 0.00 | 41.50 | 5638575.8 | 3 | 1 | 1 | 1.03 | 486 | 34454 |
| >sp\|Q9XSU7\|RL27_CANLF 60S ribosomal protein L27 OS=Canis lupus familiaris OX=9615 GN=RPL27 PE=2 SV=3 | 0.10 | 0.02 | 35.00 | 5589067.7 | 4 | 1 | 0 | 3.68 | 136 | 314 |
| >sp\|Q9GL25\|ESPB1_CANLF Epididymal sperm-binding protein 1 OS=Canis lupus familiaris OX=9615 GN=ELSPBP1 PE=1 SV=1 | 0.22 | 0.20 | 108.30 | 5246003.2 | 2 | 1 | 1 | 3.67 | 245 | 36 |
| >tr\|A0A5F4DHT9\|A0A5F4DHT9_CANLF Neurobeachin like 1 OS=Canis lupus familiaris OX=9615 GN=NBEAL1 PE=3 SV=1 | 0.21 | 0.19 | 6.40 | 5245571.7 | 2 | 1 | 0 | 0.23 | 2653 | 3732 |
| >tr\|J9P2T7\|J9P2T7_CANLF 26S proteasome non-ATPase regulatory subunit 5 OS=Canis lupus familiaris OX=9615 PE=4 SV=1 | 1.19 | 1.19 | 122.00 | 5155796.4 | 1 | 1 | 0 | 1.95 | 461 | 23870 |
| >tr\|J9P432\|J9P432_CANLF Glutamine--fructose-6-phosphate transaminase (isomerizing) OS=Canis lupus familiaris OX=9615 GN=GFPT1 PE=4 SV=2 | 1.19 | 1.19 | 116.50 | 4938685.4 | 1 | 1 | 0 | 1.18 | 677 | 7191 |
| >sp\|Q05052\|OST48_CANLF Dolichyl-diphosphooligosaccharide--protein glycosyltransferase 48 kDa subunit OS=Canis lupus familiaris OX=9615 GN=DDOST PE=1 SV=1 | 0.91 | 0.89 | 72.10 | 4899209.3 | 2 | 1 | 0 | 2.47 | 445 | 254 |
| >tr\|E2R868\|E2R868_CANLF [histone H4]-N-methyl-L-lysine20 N-methyltransferase KMT5B OS=Canis lupus familiaris OX=9615 GN=KMT5B PE=4 SV=3 | 0.10 | 0.02 | 46.30 | 4869575.4 | 2 | 2 | 1 | 1.13 | 885 | 7704 |
| >tr\|E2RG75\|E2RG75_CANLF Inactive ribonuclease-like protein 9 OS=Canis lupus familiaris OX=9615 GN=RNASE9 PE=3 SV=2 | 2.63 | 2.61 | 295.20 | 4845940.0 | 2 | 1 | 1 | 4.55 | 198 | 41734 |
| >tr\|A0A5F4DGF5\|A0A5F4DGF5_CANLF Alkaline phosphatase OS=Canis lupus familiaris OX=9615 GN=ALPL PE=3 SV=1 | 2.05 | 2.03 | 266.90 | 4834747.3 | 2 | 1 | 0 | 2.45 | 572 | 6357 |
| >tr\|A0A5F4DDV5\|A0A5F4DDV5_CANLF SMG5 nonsense mediated mRNA decay factor OS=Canis lupus familiaris OX=9615 GN=SMG5 PE=4 SV=1 | 0.18 | 0.18 | 51.60 | 4803540.3 | 1 | 1 | 0 | 0.76 | 1046 | 7629 |
| >tr\|E2R264\|E2R264_CANLF HAUS augmin like complex subunit 8 OS=Canis lupus familiaris OX=9615 GN=HAUS8 PE=4 SV=2 | 0.30 | 0.30 | 96.80 | 4797075.3 | 1 | 1 | 0 | 2.93 | 376 | 14074 |

| | | | | | | | | | | |
|---|---|---|---|---|---|---|---|---|---|---|
| >tr\|F1P8S5\|F1P8S5_CANLF Retrotransposon Gag like 1 OS=Canis lupus familiaris OX=9615 GN=RTL1 PE=4 SV=2 | 0.23 | 0.23 | 64.40 | 4773031.4 | 1 | 1 | 0 | 0.74 | 1347 | 39404 |
| >tr\|J9P432\|J9P432_CANLF Glutamine--fructose-6-phosphate transaminase (isomerizing) OS=Canis lupus familiaris OX=9615 GN=GFPT1 PE=4 SV=2 | 0.92 | 0.92 | 59.60 | 4750361.1 | 1 | 1 | 0 | 1.18 | 677 | 7191 |
| >tr\|A0A5F4D8I6\|A0A5F4D8I6_CANLF Phospholipase A2 receptor 1 OS=Canis lupus familiaris OX=9615 GN=PLA2R1 PE=4 SV=1 | 0.28 | 0.28 | 129.70 | 4718805.4 | 1 | 1 | 1 | 0.50 | 1394 | 6796 |
| >sp\|Q5JZQ9\|CLN5_CANLF Ceroid-lipofuscinosis neuronal protein 5 OS=Canis lupus familiaris OX=9615 GN=CLN5 PE=2 SV=1 | 1.08 | 1.08 | 105.00 | 4604410.2 | 1 | 1 | 0 | 1.14 | 350 | 651 |
| >tr\|F1PBJ1\|F1PBJ1_CANLF Methylcytosine dioxygenase TET OS=Canis lupus familiaris OX=9615 GN=TET3 PE=3 SV=2 | 0.10 | 0.02 | 69.90 | 4573564.7 | 2 | 1 | 0 | 0.28 | 1795 | 1529 |
| >tr\|F1PYS8\|F1PYS8_CANLF Olfactory receptor OS=Canis lupus familiaris OX=9615 GN=OR5P6 PE=3 SV=3 | 0.19 | 0.19 | 65.00 | 4442480.3 | 1 | 1 | 1 | 6.69 | 314 | 20945 |
| >sp\|P23685\|NAC1_CANLF Sodium/calcium exchanger 1 OS=Canis lupus familiaris OX=9615 GN=SLC8A1 PE=1 SV=1 | 0.26 | 0.27 | 73.90 | 4423626.8 | 1 | 1 | 0 | 1.03 | 970 | 764 |
| >tr\|F1P8S0\|F1P8S0_CANLF GPRIN family member 3 OS=Canis lupus familiaris OX=9615 GN=GPRIN3 PE=4 SV=2 | 0.39 | 0.40 | 55.70 | 4261456.3 | 1 | 1 | 0 | 1.42 | 776 | 30471 |
| >sp\|P25473\|CLUS_CANLF Clusterin OS=Canis lupus familiaris OX=9615 GN=CLU PE=2 SV=1 | 0.15 | 0.15 | 102.50 | 4189861.4 | 1 | 1 | 0 | 0.90 | 445 | 725 |
| >tr\|E2RIN4\|E2RIN4_CANLF Endoribonuclease Dicer OS=Canis lupus familiaris OX=9615 GN=DICER1 PE=3 SV=2 | 0.22 | 0.22 | 42.30 | 4165387.4 | 1 | 1 | 0 | 0.52 | 1923 | 3285 |
| >tr\|E2RH82\|E2RH82_CANLF DRY_EERY domain-containing protein OS=Canis lupus familiaris OX=9615 GN=CLASRP PE=4 SV=3 | 0.88 | 0.88 | 102.20 | 4162716.4 | 1 | 1 | 0 | 0.91 | 656 | 2531 |
| >tr\|A0A5F4D0F5\|A0A5F4D0F5_CANLF Ring finger protein 24 OS=Canis lupus familiaris OX=9615 GN=RNF24 PE=4 SV=1 | 0.39 | 0.39 | 74.50 | 4122591.0 | 1 | 1 | 0 | 7.64 | 144 | 7273 |
| >tr\|E2RSI6\|E2RSI6_CANLF Ezrin OS=Canis lupus familiaris OX=9615 GN=EZR PE=4 SV=1 | 0.41 | 0.41 | 111.40 | 3975779.6 | 1 | 1 | 0 | 1.54 | 586 | 15650 |
| >tr\|A0A5F4CLN6\|A0A5F4CLN6_CANLF DLG associated protein 4 OS=Canis lupus familiaris OX=9615 GN=DLGAP4 PE=3 SV=1 | 0.27 | 0.27 | 35.40 | 3800979.2 | 1 | 1 | 0 | 3.04 | 461 | 7940 |
| >tr\|J9P2T7\|J9P2T7_CANLF 26S proteasome non-ATPase regulatory subunit 5 OS=Canis lupus familiaris OX=9615 PE=4 SV=1 | 0.24 | 0.24 | 105.80 | 3783122.6 | 1 | 1 | 0 | 1.95 | 461 | 23870 |
| >tr\|E2RM62\|E2RM62_CANLF FAT atypical cadherin 2 OS=Canis lupus familiaris OX=9615 GN=FAT2 PE=4 SV=1 | 0.23 | 0.23 | 65.00 | 3771599.7 | 1 | 1 | 0 | 0.34 | 4354 | 8419 |
| >tr\|J9P883\|J9P883_CANLF Structural maintenance of chromosomes protein 5 OS=Canis lupus familiaris OX=9615 GN=SMC5 PE=3 SV=1 | 0.23 | 0.23 | 118.70 | 3688528.9 | 1 | 1 | 0 | 0.63 | 1106 | 5004 |
| >tr\|E2RTL2\|E2RTL2_CANLF Tubulin tyrosine ligase like 6 OS=Canis lupus familiaris OX=9615 GN=TTLL6 PE=4 SV=3 | 0.38 | 0.39 | 46.60 | 3677553.7 | 1 | 1 | 0 | 0.60 | 827 | 2703 |
| >tr\|F1PB88\|F1PB88_CANLF Nik related kinase OS=Canis lupus familiaris OX=9615 GN=NRK PE=4 SV=3 | 0.92 | 0.93 | 93.00 | 3617672.0 | 1 | 1 | 0 | 0.46 | 1522 | 3609 |
| >tr\|F1Q3K9\|F1Q3K9_CANLF Activator of transcription and developmental regulator AUTS2 OS=Canis lupus familiaris OX=9615 GN=AUTS2 PE=4 SV=3 | 0.15 | 0.15 | 102.80 | 3578840.3 | 1 | 1 | 0 | 0.87 | 1029 | 9473 |
| >tr\|J9P2T7\|J9P2T7_CANLF 26S proteasome non-ATPase regulatory subunit 5 OS=Canis lupus familiaris OX=9615 PE=4 SV=1 | 0.45 | 0.45 | 95.10 | 3485271.8 | 1 | 1 | 0 | 1.95 | 461 | 23870 |
| >tr\|E2RG75\|E2RG75_CANLF Inactive ribonuclease-like protein 9 OS=Canis lupus familiaris OX=9615 GN=RNASE9 PE=3 SV=2 | 1.85 | 1.81 | 251.00 | 3411153.5 | 3 | 1 | 1 | 4.55 | 198 | 41734 |
| >tr\|A0A5F4C745\|A0A5F4C745_CANLF HECT-type E3 ubiquitin transferase OS=Canis lupus familiaris OX=9615 GN=HERC2 PE=4 SV=1 | 0.10 | 0.02 | 47.00 | 3331439.9 | 2 | 1 | 0 | 0.10 | 4862 | 967 |

| | | | | | | | | | | |
|---|---|---|---|---|---|---|---|---|---|---|
| >tr\|F1P719\|F1P719_CANLF EH domain containing 4 OS=Canis lupus familiaris OX=9615 GN=EHD4 PE=4 SV=2 | 0.96 | 0.96 | 41.20 | 3331432.8 | 1 | 1 | 0 | 1.85 | 541 | 8517 |
| >tr\|E2R1K2\|E2R1K2_CANLF RAB, member RAS oncogene family like 6 OS=Canis lupus familiaris OX=9615 GN=RABL6 PE=4 SV=1 | 0.10 | 0.00 | 11.30 | 3314552.9 | 4 | 1 | 0 | 0.70 | 715 | 6632 |
| >tr\|A0A5F4C2W9\|A0A5F4C2W9_CANLF Methyl-CpG binding domain protein 1 OS=Canis lupus familiaris OX=9615 GN=MBD1 PE=4 SV=1 | 0.26 | 0.26 | 114.50 | 3212872.2 | 1 | 1 | 0 | 1.71 | 703 | 14519 |
| >tr\|A0A5F4C3H5\|A0A5F4C3H5_CANLF Purine rich element binding protein G OS=Canis lupus familiaris OX=9615 GN=PURG PE=3 SV=1 | 0.15 | 0.15 | 90.00 | 3150858.2 | 1 | 1 | 0 | 2.82 | 319 | 1070 |
| >tr\|J9P6W8\|J9P6W8_CANLF Chromobox 8 OS=Canis lupus familiaris OX=9615 GN=CBX8 PE=4 SV=2 | 0.21 | 0.22 | 29.90 | 2783344.6 | 1 | 1 | 1 | 2.99 | 368 | 19169 |
| >tr\|H9GWH3\|H9GWH3_CANLF LRRC37AB_C domain-containing protein OS=Canis lupus familiaris OX=9615 GN=LOC491436 PE=4 SV=3 | 0.24 | 0.24 | 137.30 | 2675078.4 | 1 | 1 | 0 | 0.75 | 796 | 5712 |
| >tr\|J9P2D3\|J9P2D3_CANLF Alpha kinase 3 OS=Canis lupus familiaris OX=9615 GN=ALPK3 PE=4 SV=2 | 0.15 | 0.15 | 106.30 | 2518644.1 | 1 | 1 | 0 | 0.50 | 1616 | 5104 |
| >tr\|J9NUT9\|J9NUT9_CANLF Pericentrin OS=Canis lupus familiaris OX=9615 GN=PCNT PE=4 SV=2 | 0.13 | 0.14 | 162.10 | 2414689.7 | 1 | 1 | 0 | 0.19 | 3208 | 18609 |
| >tr\|A0A5F4CLU1\|A0A5F4CLU1_CANLF Superoxide dismutase [Cu-Zn] OS=Canis lupus familiaris OX=9615 GN=SOD1 PE=3 SV=1 | 0.40 | 0.40 | 79.20 | 2410644.2 | 1 | 1 | 0 | 7.09 | 141 | 25417 |
| >tr\|E2RG75\|E2RG75_CANLF Inactive ribonuclease-like protein 9 OS=Canis lupus familiaris OX=9615 GN=RNASE9 PE=3 SV=2 | 0.66 | 0.66 | 301.50 | 2181445.8 | 1 | 1 | 1 | 4.55 | 198 | 41734 |
| >tr\|E2R868\|E2R868_CANLF [histone H4]-N-methyl-L-lysine20 N-methyltransferase KMT5B OS=Canis lupus familiaris OX=9615 GN=KMT5B PE=4 SV=3 | 0.78 | 0.78 | 51.80 | 2150072.2 | 1 | 1 | 0 | 0.56 | 885 | 7704 |
| >tr\|A0A5F4C2A7\|A0A5F4C2A7_CANLF SECIS binding protein 2 OS=Canis lupus familiaris OX=9615 GN=SECISBP2 PE=4 SV=1 | 0.34 | 0.34 | 163.50 | 2081362.1 | 1 | 1 | 0 | 1.39 | 863 | 11966 |
| >tr\|A0A5F4CGA0\|A0A5F4CGA0_CANLF IRG-type G domain-containing protein OS=Canis lupus familiaris OX=9615 GN=LOC481472 PE=3 SV=1 | 0.18 | 0.18 | 108.40 | 2002421.3 | 1 | 1 | 0 | 1.23 | 408 | 33545 |
| >tr\|J9NSS6\|J9NSS6_CANLF DNA helicase OS=Canis lupus familiaris OX=9615 GN=CHD2 PE=4 SV=2 | 0.10 | 0.02 | 30.80 | 1774003.3 | 2 | 1 | 0 | 0.28 | 1780 | 1264 |
| >tr\|A0A5F4BZ61\|A0A5F4BZ61_CANLF G_PROTEIN_RECEP_F1_2 domain-containing protein OS=Canis lupus familiaris OX=9615 GN=OR5D13 PE=4 SV=1 | 0.10 | 0.02 | 26.40 | 1711126.5 | 1 | 1 | 0 | 1.70 | 294 | 37537 |
| >tr\|Q9XSV4\|Q9XSV4_CANLF CE10 protein OS=Canis lupus familiaris OX=9615 GN=ce10 PE=2 SV=1 | 4.38 | 3.15 | 337.30 | 1595297.1 | 18 | 2 | 0 | 9.09 | 110 | 41542 |
| >tr\|F1PI09\|F1PI09_CANLF Aldehyde oxidase OS=Canis lupus familiaris OX=9615 GN=AOX2 PE=3 SV=3 | 0.21 | 0.21 | 163.90 | 1245918.8 | 1 | 1 | 0 | 0.67 | 1347 | 21650 |
| >tr\|F1PR54\|F1PR54_CANLF Lactotransferrin OS=Canis lupus familiaris OX=9615 GN=LTF PE=3 SV=1 | 4.15 | 3.57 | 370.70 | 1178722.5 | 12 | 2 | 0 | 3.95 | 708 | 40436 |
| >sp\|O18840\|ACTB_CANLF Actin, cytoplasmic 1 OS=Canis lupus familiaris OX=9615 GN=ACTB PE=2 SV=3 | 4.24 | 1.67 | 271.40 | 1168988.9 | 7 | 3 | 0 | 12.00 | 375 | 642 |
| >sp\|Q28279\|CNGA1_CANLF cGMP-gated cation channel alpha-1 OS=Canis lupus familiaris OX=9615 GN=CNGA1 PE=2 SV=1 | 0.10 | 0.09 | 138.90 | 1020267.9 | 1 | 1 | 0 | 0.58 | 691 | 455 |
| >tr\|F1PKS8\|F1PKS8_CANLF Anoctamin OS=Canis lupus familiaris OX=9615 GN=VWF PE=3 SV=3 | 0.36 | 0.34 | 129.60 | 1005732.9 | 2 | 1 | 1 | 1.61 | 992 | 42310 |
| >tr\|A0A5F4CYU0\|A0A5F4CYU0_CANLF RAS and EF-hand domain containing OS=Canis lupus familiaris OX=9615 GN=RASEF PE=4 SV=1 | 0.15 | 0.15 | 49.40 | 992527.8 | 1 | 1 | 0 | 0.50 | 795 | 864 |

| | | | | | | | | | | |
|---|---|---|---|---|---|---|---|---|---|---|
| >tr\|E2RCT1\|E2RCT1_CANLF WAP domain-containing protein OS=Canis lupus familiaris OX=9615 PE=4 SV=2 | 3.13 | 2.03 | 198.50 | 789288.7 | 8 | 2 | 0 | 14.66 | 116 | 21717 |
| >tr\|F1PUY9\|F1PUY9_CANLF Mucin 4, cell surface associated OS=Canis lupus familiaris OX=9615 GN=MUC4 PE=4 SV=3 | 0.15 | 0.15 | 19.20 | 662103.3 | 1 | 1 | 0 | 1.77 | 1132 | 28376 |
| >sp\|Q9XSU7\|RL27_CANLF 60S ribosomal protein L27 OS=Canis lupus familiaris OX=9615 GN=RPL27 PE=2 SV=3 | 0.10 | 0.02 | 8.60 | 658805.3 | 1 | 1 | 0 | 3.68 | 136 | 314 |
| >sp\|Q9XS65\|PTGDS_CANLF Prostaglandin-H2 D-isomerase OS=Canis lupus familiaris OX=9615 GN=PTGDS PE=2 SV=1 | 0.99 | 0.75 | 209.10 | 503395.2 | 5 | 2 | 1 | 16.23 | 191 | 165 |
| >tr\|A0A5F4D304\|A0A5F4D304_CANLF Ring finger protein 112 OS=Canis lupus familiaris OX=9615 GN=RNF112 PE=3 SV=1 | 0.23 | 0.23 | 37.20 | 426238.2 | 1 | 1 | 0 | 1.49 | 871 | 5762 |
| >tr\|A0A5F4C5N3\|A0A5F4C5N3_CANLF Signal induced proliferation associated 1 like 2 OS=Canis lupus familiaris OX=9615 GN=SIPA1L2 PE=4 SV=1 | 0.27 | 0.25 | 171.50 | 421468.7 | 2 | 1 | 0 | 0.24 | 1676 | 5347 |
| >tr\|E2R6E0\|E2R6E0_CANLF Lipocln_cytosolic_FA-bd_dom domain-containing protein OS=Canis lupus familiaris OX=9615 GN=LCNL1 PE=3 SV=2 | 3.36 | 2.32 | 138.90 | 389301.4 | 5 | 2 | 0 | 6.35 | 299 | 1932 |
| >tr\|F1P6L3\|F1P6L3_CANLF Junctional cadherin 5 associated OS=Canis lupus familiaris OX=9615 GN=JCAD PE=4 SV=2 | 0.51 | 0.51 | 87.60 | 335206.3 | 1 | 1 | 1 | 0.66 | 1364 | 44204 |
| >tr\|E2R203\|E2R203_CANLF Ring finger protein 216 OS=Canis lupus familiaris OX=9615 GN=RNF216 PE=4 SV=2 | 0.27 | 0.27 | 8.20 | 318557.4 | 1 | 1 | 0 | 1.29 | 927 | 32362 |
| >tr\|F1P985\|F1P985_CANLF Tumor protein p53 binding protein 2 OS=Canis lupus familiaris OX=9615 GN=TP53BP2 PE=4 SV=3 | 0.10 | 0.10 | 38.90 | 203003.6 | 1 | 1 | 1 | 0.75 | 1200 | 2639 |
| >tr\|A0A5F4CCD0\|A0A5F4CCD0_CANLF Cysteine rich secretory protein 2 OS=Canis lupus familiaris OX=9615 GN=CRISP2 PE=3 SV=1 | 0.72 | 0.70 | 82.30 | 176461.5 | 2 | 1 | 0 | 4.82 | 311 | 11017 |
| >tr\|F1Q013\|F1Q013_CANLF Glucoside xylosyltransferase 2 OS=Canis lupus familiaris OX=9615 GN=GXYLT2 PE=4 SV=3 | 0.17 | 0.15 | 26.50 | 169490.2 | 2 | 1 | 1 | 4.07 | 442 | 27211 |
| >tr\|F1PS03\|F1PS03_CANLF 1-phosphatidylinositol 4,5-bisphosphate phosphodiesterase gamma OS=Canis lupus familiaris OX=9615 GN=PLCG1 PE=4 SV=2 | 0.60 | 0.60 | 36.90 | 111264.3 | 1 | 1 | 0 | 1.23 | 1217 | 6493 |
| >tr\|F1PL65\|F1PL65_CANLF KIAA1217 OS=Canis lupus familiaris OX=9615 GN=KIAA1217 PE=4 SV=3 | 0.14 | 0.15 | 104.70 | 109957.5 | 1 | 1 | 1 | 0.80 | 1997 | 3670 |
| >tr\|A0A5F4CHU9\|A0A5F4CHU9_CANLF E1A binding protein p400 OS=Canis lupus familiaris OX=9615 GN=EP400 PE=4 SV=1 | 0.18 | 0.18 | 25.80 | 96280.8 | 1 | 1 | 1 | 0.70 | 3002 | 5590 |
| >tr\|A0A5F4C9W6\|A0A5F4C9W6_CANLF Armadillo like helical domain containing 3 OS=Canis lupus familiaris OX=9615 GN=ARMH3 PE=4 SV=1 | 0.32 | 0.32 | 19.10 | 92607.9 | 1 | 1 | 1 | 2.30 | 697 | 22893 |
| >tr\|A0A5F4BP01\|A0A5F4BP01_CANLF Cyclin dependent kinase like 5 OS=Canis lupus familiaris OX=9615 GN=CDKL5 PE=4 SV=1 | 0.14 | 0.15 | 25.70 | 90145.4 | 1 | 1 | 1 | 1.60 | 1001 | 930 |
| >tr\|A0A5F4CB26\|A0A5F4CB26_CANLF F-box protein 33 OS=Canis lupus familiaris OX=9615 GN=FBXO33 PE=4 SV=1 | 0.34 | 0.34 | 64.20 | 84564.9 | 1 | 1 | 0 | 1.90 | 525 | 9458 |
| >sp\|P49822\|ALBU_CANLF Albumin OS=Canis lupus familiaris OX=9615 GN=ALB PE=1 SV=3 | 1.14 | 1.14 | 20.90 | 84546.2 | 1 | 1 | 0 | 2.80 | 608 | 490 |
| >tr\|A0A5F4CZX8\|A0A5F4CZX8_CANLF Exocyst complex component 7 OS=Canis lupus familiaris OX=9615 GN=EXOC7 PE=3 SV=1 | 0.31 | 0.31 | 8.90 | 80200.2 | 1 | 1 | 0 | 1.69 | 890 | 9605 |
| >tr\|F1PJP1\|F1PJP1_CANLF Dynein axonemal heavy chain 11 OS=Canis lupus familiaris OX=9615 GN=DNAH11 PE=3 SV=3 | 0.30 | 0.30 | 17.70 | 79011.7 | 1 | 1 | 0 | 0.35 | 4519 | 42705 |
| >tr\|A0A5F4D7B4\|A0A5F4D7B4_CANLF SUI1 domain-containing protein OS=Canis lupus familiaris OX=9615 PE=3 SV=1 | 0.30 | 0.30 | 5.50 | 78605.4 | 1 | 1 | 0 | 10.91 | 165 | 38637 |

| | | | | | | | | | | |
|---|---|---|---|---|---|---|---|---|---|---|
| >tr\|E2RNJ7\|E2RNJ7_CANLF ETS variant transcription factor 6 OS=Canis lupus familiaris OX=9615 GN=ETV6 PE=3 SV=2 | 0.38 | 0.38 | 66.40 | 76486.0 | 1 | 1 | 0 | 1.55 | 452 | 3147 |
| >tr\|E2RI36\|E2RI36_CANLF Nuclear transcription factor, X-box binding 1 OS=Canis lupus familiaris OX=9615 GN=NFX1 PE=3 SV=3 | 1.28 | 1.28 | 109.40 | 76468.1 | 1 | 1 | 0 | 0.89 | 1118 | 13894 |
| >tr\|E2RDC9\|E2RDC9_CANLF Family with sequence similarity 53 member A OS=Canis lupus familiaris OX=9615 GN=FAM53A PE=3 SV=2 | 0.32 | 0.32 | 92.50 | 76412.4 | 1 | 1 | 0 | 3.50 | 371 | 11515 |
| >tr\|A0A5F4DLH0\|A0A5F4DLH0_CANLF Cyclin-C OS=Canis lupus familiaris OX=9615 GN=CCNC PE=3 SV=1 | 0.32 | 0.32 | 22.30 | 76145.2 | 1 | 1 | 0 | 4.43 | 271 | 17674 |
| >sp\|Q28895\|NPC2_CANLF NPC intracellular cholesterol transporter 2 OS=Canis lupus familiaris OX=9615 GN=NPC2 PE=2 SV=1 | 1.06 | 1.06 | 76.10 | 75676.8 | 1 | 1 | 0 | 6.04 | 149 | 153 |
| >tr\|A0A5F4BZB8\|A0A5F4BZB8_CANLF Supervillin OS=Canis lupus familiaris OX=9615 GN=SVIL PE=3 SV=1 | 0.59 | 0.60 | 15.70 | 75563.4 | 1 | 1 | 0 | 0.77 | 2215 | 2770 |
| >tr\|A0A5F4CIF3\|A0A5F4CIF3_CANLF Calcium binding protein 2 OS=Canis lupus familiaris OX=9615 GN=CABP2 PE=4 SV=1 | 0.34 | 0.34 | 22.00 | 75349.7 | 1 | 1 | 0 | 9.82 | 163 | 11050 |
| >tr\|E2R9F0\|E2R9F0_CANLF Tyrosine kinase non receptor 1 OS=Canis lupus familiaris OX=9615 GN=TNK1 PE=4 SV=3 | 0.82 | 0.82 | 28.50 | 73631.9 | 1 | 1 | 1 | 1.81 | 663 | 37868 |
| >tr\|A0A5F4C148\|A0A5F4C148_CANLF Centromere protein J OS=Canis lupus familiaris OX=9615 GN=CENPJ PE=3 SV=1 | 1.01 | 1.01 | 77.90 | 72116.8 | 1 | 1 | 0 | 0.78 | 1274 | 15824 |
| >tr\|A0A5F4D513\|A0A5F4D513_CANLF Contactin 4 OS=Canis lupus familiaris OX=9615 GN=CNTN4 PE=4 SV=1 | 0.13 | 0.13 | 25.20 | 71734.9 | 1 | 1 | 0 | 0.62 | 973 | 7826 |
| >tr\|F1P9Y3\|F1P9Y3_CANLF Complex I-30kD OS=Canis lupus familiaris OX=9615 GN=NDUFS3 PE=3 SV=2 | 0.15 | 0.15 | 23.80 | 62079.0 | 1 | 1 | 0 | 4.04 | 322 | 18622 |
