# Supplementary material for: Age-Dependent Variations in Functional Quality and Proteomic Characteristics of Canine (Canis lupus familiaris) Epididymal Spermatozoa
Source: Int J Mol Sci. 2022 Aug 15;23(16):9143. doi: 10.3390/ijms23169143 (PMC9409041; doi:10.3390/ijms23169143)
Supplement: Supplementary file 1 [file ijms-23-09143-s001.zip › Supplementary Table S3.pdf]

Table S3 Supplementary. Proteins of Group 3 (78 to 132 months old) dog (*Canis lupus familiaris*) epididymal spermatozoa evaluated by mass spectrometry (NanoUPLC-Q-TOF/MS).

Description	|Log Prob|	Best |Log Prob|	Best score	Total Intensity	# of spectra	# of unique peptides	# of mod peptides	Coverage %	# AA's in protein	Protein DB number
>tr|A0A5F4BVF3|A0A5F4BVF3_CANLF Lactotransferrin OS=Canis lupus familiaris OX=9615 GN=LTF PE=3 SV=1	8.24	2.47	289.50	708660872.9	51	8	0	7.99	626	32850
>tr|A0A5F4BVF3|A0A5F4BVF3_CANLF Lactotransferrin OS=Canis lupus familiaris OX=9615 GN=LTF PE=3 SV=1	16.39	4.76	391.20	692545448.9	87	13	0	17.25	626	32850
>tr|F1PR54|F1PR54_CANLF Lactotransferrin OS=Canis lupus familiaris OX=9615 GN=LTF PE=3 SV=1	20.54	4.06	423.00	652964790.4	92	17	1	22.46	708	40436
>sp|P62286|ASPM_CANLF Abnormal spindle-like microcephaly-associated protein homolog OS=Canis lupus familiaris OX=9615 GN=ASPM PE=2 SV=2	0.25	0.15	86.60	605260377.9	67	3	0	0.29	3469	677
>sp|Q697L1|TRPV1_CANLF Transient receptor potential cation channel subfamily V member 1 OS=Canis lupus familiaris OX=9615 GN=TRPV1 PE=2 SV=1	2.18	0.16	115.70	587367198.5	118	3	2	0.71	840	297
>sp|Q697L1|TRPV1_CANLF Transient receptor potential cation channel subfamily V member 1 OS=Canis lupus familiaris OX=9615 GN=TRPV1 PE=2 SV=1	1.94	0.10	118.50	581120430.2	112	3	2	0.71	840	297
>tr|F1PR54|F1PR54_CANLF Lactotransferrin OS=Canis lupus familiaris OX=9615 GN=LTF PE=3 SV=1	23.56	4.15	274.10	575486227.0	90	14	1	22.03	708	40436
>tr|F1PJ71|F1PJ71_CANLF Glutathione peroxidase OS=Canis lupus familiaris OX=9615 GN=GPX5 PE=3 SV=2	11.93	3.79	293.40	558589605.0	68	8	1	30.32	221	19009
>sp|E2RKA8|RL32_CANLF 60S ribosomal protein L32 OS=Canis lupus familiaris OX=9615 GN=RPL32 PE=1 SV=1	3.52	0.59	116.90	555722532.7	133	2	0	4.44	135	275
>tr|A0A5F4BVF3|A0A5F4BVF3_CANLF Lactotransferrin OS=Canis lupus familiaris OX=9615 GN=LTF PE=3 SV=1	17.44	2.38	424.60	448583852.7	100	16	2	24.76	626	32850
>sp|Q9XS65|PTGDS_CANLF Prostaglandin-H2 D-isomerase OS=Canis lupus familiaris OX=9615 GN=PTGDS PE=2 SV=1	5.28	2.20	294.90	434245415.3	55	6	1	24.08	191	165
>tr|F1PJ71|F1PJ71_CANLF Glutathione peroxidase OS=Canis lupus familiaris OX=9615 GN=GPX5 PE=3 SV=2	13.01	2.06	361.60	429116073.1	65	8	1	31.67	221	19009
>sp|Q9XS65|PTGDS_CANLF Prostaglandin-H2 D-isomerase OS=Canis lupus familiaris OX=9615 GN=PTGDS PE=2 SV=1	6.06	1.48	395.20	359426817.2	53	7	1	18.85	191	165
>sp|P62286|ASPM_CANLF Abnormal spindle-like microcephaly-associated protein homolog OS=Canis lupus familiaris OX=9615 GN=ASPM PE=2 SV=2	2.34	0.21	103.00	349492154.5	94	3	0	0.23	3469	677
>sp|P62286|ASPM_CANLF Abnormal spindle-like microcephaly-associated protein homolog OS=Canis lupus familiaris OX=9615 GN=ASPM PE=2 SV=2	1.76	0.33	125.40	349134726.5	60	3	0	0.23	3469	677
>sp|Q9XS65|PTGDS_CANLF Prostaglandin-H2 D-isomerase OS=Canis lupus familiaris OX=9615 GN=PTGDS PE=2 SV=1	6.02	2.27	354.50	329937435.5	35	3	1	10.47	191	165
>sp|E2RKA8|RL32_CANLF 60S ribosomal protein L32 OS=Canis lupus familiaris OX=9615 GN=RPL32 PE=1 SV=1	0.65	0.02	136.10	305681280.0	55	2	0	4.44	135	275
>tr|F1PR54|F1PR54_CANLF Lactotransferrin OS=Canis lupus familiaris OX=9615 GN=LTF PE=3 SV=1	18.69	3.07	397.90	294005519.4	75	15	0	18.22	708	40436
>sp|P62286|ASPM_CANLF Abnormal spindle-like microcephaly-associated protein homolog OS=Canis lupus familiaris OX=9615 GN=ASPM PE=2 SV=2	1.96	0.14	85.20	287427350.8	90	3	0	0.32	3469	677
>sp|O18840|ACTB_CANLF Actin, cytoplasmic 1 OS=Canis lupus familiaris OX=9615 GN=ACTB PE=2 SV=3	4.71	3.57	281.80	273998734.5	19	2	0	5.33	375	642

>tr|A0A5F4DD58|A0A5F4DD58_CANLF Phosphoinositide phospholipase C OS=Canis lupus familiaris OX=9615 GN=PLCD3 PE=4 SV=1	0.51	0.31	230.40	232039410.9	140	3	0	1.48	741	3088
>sp|P25473|CLUS_CANLF Clusterin OS=Canis lupus familiaris OX=9615 GN=CLU PE=2 SV=1	5.63	2.22	328.30	211355223.5	32	7	0	8.99	445	725
>tr|A0A5F4BVF3|A0A5F4BVF3_CANLF Lactotransferrin OS=Canis lupus familiaris OX=9615 GN=LTF PE=3 SV=1	9.40	3.45	337.00	208889865.0	38	8	0	9.42	626	32850
>sp|O18840|ACTB_CANLF Actin, cytoplasmic 1 OS=Canis lupus familiaris OX=9615 GN=ACTB PE=2 SV=3	6.01	3.34	280.70	198640885.7	18	2	0	5.33	375	642
>sp|P49822|ALBU_CANLF Albumin OS=Canis lupus familiaris OX=9615 GN=ALB PE=1 SV=3	3.86	2.52	211.10	188982271.2	22	4	0	8.39	608	490
>sp|O18840|ACTB_CANLF Actin, cytoplasmic 1 OS=Canis lupus familiaris OX=9615 GN=ACTB PE=2 SV=3	3.48	1.23	219.10	186364574.1	17	3	0	10.13	375	642
>sp|O18840|ACTB_CANLF Actin, cytoplasmic 1 OS=Canis lupus familiaris OX=9615 GN=ACTB PE=2 SV=3	6.39	3.83	322.20	168494180.5	21	5	0	8.27	375	642
>sp|Q9XS65|PTGDS_CANLF Prostaglandin-H2 D-isomerase OS=Canis lupus familiaris OX=9615 GN=PTGDS PE=2 SV=1	3.01	1.11	351.10	167606083.6	35	3	1	13.61	191	165
>sp|O18840|ACTB_CANLF Actin, cytoplasmic 1 OS=Canis lupus familiaris OX=9615 GN=ACTB PE=2 SV=3	9.22	4.49	350.60	152859001.6	24	5	0	13.07	375	642
>tr|A0A5F4C1S8|A0A5F4C1S8_CANLF E3 ubiquitin-protein ligase CBL OS=Canis lupus familiaris OX=9615 GN=CBL PE=4 SV=1	0.50	0.08	153.60	151951512.7	21	2	0	0.91	773	1308
>tr|A0A5F4C1S8|A0A5F4C1S8_CANLF E3 ubiquitin-protein ligase CBL OS=Canis lupus familiaris OX=9615 GN=CBL PE=4 SV=1	0.88	0.46	223.10	148111446.0	22	1	0	0.52	773	1308
>tr|A0A5F4D6L9|A0A5F4D6L9_CANLF Sacsin molecular chaperone OS=Canis lupus familiaris OX=9615 GN=SACS PE=4 SV=1	0.81	0.54	194.60	140131501.7	44	3	0	0.27	4500	1444
>sp|O18840|ACTB_CANLF Actin, cytoplasmic 1 OS=Canis lupus familiaris OX=9615 GN=ACTB PE=2 SV=3	4.27	3.70	334.80	137279040.9	13	2	0	4.53	375	642
>tr|A0A5F4CEM0|A0A5F4CEM0_CANLF Proline rich coiled-coil 2B OS=Canis lupus familiaris OX=9615 GN=PRRC2B PE=4 SV=1	0.16	0.02	127.90	135370939.7	13	1	1	0.58	2235	37089
>sp|O18840|ACTB_CANLF Actin, cytoplasmic 1 OS=Canis lupus familiaris OX=9615 GN=ACTB PE=2 SV=3	3.08	2.45	414.20	133874317.1	14	2	0	4.53	375	642
>sp|P49822|ALBU_CANLF Albumin OS=Canis lupus familiaris OX=9615 GN=ALB PE=1 SV=3	4.18	1.15	322.70	123903818.7	21	7	0	15.13	608	490
>tr|Q9XSV4|Q9XSV4_CANLF CE10 protein OS=Canis lupus familiaris OX=9615 GN=ce10 PE=2 SV=1	3.28	2.61	253.30	117020949.8	19	3	0	14.55	110	41542
>sp|P49822|ALBU_CANLF Albumin OS=Canis lupus familiaris OX=9615 GN=ALB PE=1 SV=3	3.82	0.88	276.20	114292000.1	19	5	1	8.39	608	490
>tr|E2RE16|E2RE16_CANLF Non-specific serine/threonine protein kinase OS=Canis lupus familiaris OX=9615 GN=PAK4 PE=4 SV=1	0.61	0.02	86.70	112965242.4	57	1	0	0.84	592	12735
>sp|O18840|ACTB_CANLF Actin, cytoplasmic 1 OS=Canis lupus familiaris OX=9615 GN=ACTB PE=2 SV=3	5.33	3.46	321.20	111249282.4	15	3	0	5.60	375	642
>sp|O18840|ACTB_CANLF Actin, cytoplasmic 1 OS=Canis lupus familiaris OX=9615 GN=ACTB PE=2 SV=3	7.26	4.16	385.40	110173530.3	15	4	0	8.27	375	642
>tr|F1PR54|F1PR54_CANLF Lactotransferrin OS=Canis lupus familiaris OX=9615 GN=LTF PE=3 SV=1	18.92	3.64	313.90	109770181.5	47	12	0	15.96	708	40436

>tr|A0A5F4BVF3|A0A5F4BVF3_CANLF Lactotransferrin OS=Canis lupus familiaris OX=9615 GN=LTF PE=3 SV=1	4.24	2.10	303.40	109258034.8	21	5	0	5.91	626	32850
>tr|E2RN16|E2RN16_CANLF Mitogen-activated protein kinase kinase kinase 2 OS=Canis lupus familiaris OX=9615 GN=MAP3K2 PE=4 SV=2	0.26	0.10	219.10	106763900.6	10	1	0	0.97	620	34325
>sp|P49822|ALBU_CANLF Albumin OS=Canis lupus familiaris OX=9615 GN=ALB PE=1 SV=3	4.45	2.36	295.00	93470727.3	17	6	1	10.86	608	490
>tr|A0A5F4BVF3|A0A5F4BVF3_CANLF Lactotransferrin OS=Canis lupus familiaris OX=9615 GN=LTF PE=3 SV=1	4.84	2.92	299.40	87110930.6	17	3	0	3.99	626	32850
>tr|A0A5F4BVF3|A0A5F4BVF3_CANLF Lactotransferrin OS=Canis lupus familiaris OX=9615 GN=LTF PE=3 SV=1	4.71	2.62	306.30	83811883.9	18	4	0	5.75	626	32850
>tr|A0A5F4CAH2|A0A5F4CAH2_CANLF RNA polymerase II subunit A C-terminal domain phosphatase OS=Canis lupus familiaris OX=9615 GN=CTDP1 PE=4 SV=1	0.53	0.35	137.30	83769899.9	10	1	0	0.74	945	14396
>tr|A0A5F4DKE8|A0A5F4DKE8_CANLF Suppression of tumorigenicity 7 like OS=Canis lupus familiaris OX=9615 GN=ST7L PE=3 SV=1	1.33	0.86	202.40	83267598.1	18	2	0	1.24	644	5269
>sp|P25473|CLUS_CANLF Clusterin OS=Canis lupus familiaris OX=9615 GN=CLU PE=2 SV=1	1.04	0.89	275.80	80533012.8	10	3	0	3.37	445	725
>tr|A0A5F4CAH2|A0A5F4CAH2_CANLF RNA polymerase II subunit A C-terminal domain phosphatase OS=Canis lupus familiaris OX=9615 GN=CTDP1 PE=4 SV=1	0.29	0.13	133.60	76625383.7	9	1	0	0.74	945	14396
>tr|Q9XSV4|Q9XSV4_CANLF CE10 protein OS=Canis lupus familiaris OX=9615 GN=ce10 PE=2 SV=1	7.57	3.06	363.80	74770867.6	31	4	0	23.64	110	41542
>tr|F1PGK9|F1PGK9_CANLF ADAM metallopeptidase with thrombospondin type 1 motif 5 OS=Canis lupus familiaris OX=9615 GN=ADAMTS5 PE=4 SV=3	0.29	0.02	62.80	71960179.4	31	1	0	0.59	845	11956
>tr|E2RRP1|E2RRP1_CANLF Butyryl-CoA dehydrogenase OS=Canis lupus familiaris OX=9615 GN=IVD PE=3 SV=2	0.20	0.12	103.90	69416871.5	5	1	0	3.99	426	10739
>sp|P49822|ALBU_CANLF Albumin OS=Canis lupus familiaris OX=9615 GN=ALB PE=1 SV=3	4.75	2.74	244.10	68239829.4	10	4	1	6.91	608	490
>tr|J9NS28|J9NS28_CANLF RBR-type E3 ubiquitin transferase OS=Canis lupus familiaris OX=9615 GN=ANKIB1 PE=4 SV=2	0.42	0.36	78.40	68169466.9	4	1	0	2.33	988	26345
>tr|F1PGK9|F1PGK9_CANLF ADAM metallopeptidase with thrombospondin type 1 motif 5 OS=Canis lupus familiaris OX=9615 GN=ADAMTS5 PE=4 SV=3	0.23	0.02	35.60	65563484.4	32	1	0	0.59	845	11956
>tr|J9NTK2|J9NTK2_CANLF J domain-containing protein OS=Canis lupus familiaris OX=9615 GN=DNAJC12 PE=4 SV=2	0.46	0.36	169.00	65402747.5	6	1	0	4.72	106	2310
>tr|A0A5F4DKE8|A0A5F4DKE8_CANLF Suppression of tumorigenicity 7 like OS=Canis lupus familiaris OX=9615 GN=ST7L PE=3 SV=1	1.98	0.87	205.70	64765730.2	15	2	0	1.24	644	5269
>tr|A0A5K1V0D8|A0A5K1V0D8_CANLF Sulfatase 2 OS=Canis lupus familiaris OX=9615 GN=SULF2 PE=3 SV=1	0.10	0.02	189.60	64093192.6	11	1	0	0.35	859	1192
>tr|F1PBJ1|F1PBJ1_CANLF Methylcytosine dioxygenase TET OS=Canis lupus familiaris OX=9615 GN=TET3 PE=3 SV=2	0.40	0.10	44.80	62836488.3	16	1	0	0.28	1795	1529
>tr|Q9XSV4|Q9XSV4_CANLF CE10 protein OS=Canis lupus familiaris OX=9615 GN=ce10 PE=2 SV=1	1.02	0.88	266.60	62465982.2	10	1	0	9.09	110	41542
>tr|E2RG76|E2RG76_CANLF Inactive ribonuclease-like protein 10 OS=Canis lupus familiaris OX=9615 GN=RNASE10 PE=3 SV=2	0.22	0.10	202.90	59813431.0	8	1	0	3.70	297	8212
>tr|E2RE16|E2RE16_CANLF Non-specific serine/threonine protein kinase OS=Canis lupus familiaris OX=9615 GN=PAK4 PE=4 SV=1	0.18	0.02	57.60	59242568.1	23	1	0	0.84	592	12735

>sp|P49822|ALBU_CANLF Albumin OS=Canis lupus familiaris OX=9615 GN=ALB PE=1 SV=3	0.52	0.44	162.20	58078598.7	6	2	1	2.30	608	490
>tr|J9NYC7|J9NYC7_CANLF Dynein axonemal heavy chain 12 OS=Canis lupus familiaris OX=9615 GN=DNAH12 PE=3 SV=1	0.41	0.36	179.30	56593223.2	4	1	0	0.33	3960	15992
>sp|Q28895|NPC2_CANLF NPC intracellular cholesterol transporter 2 OS=Canis lupus familiaris OX=9615 GN=NPC2 PE=2 SV=1	3.96	1.84	408.10	56541705.1	29	4	0	36.91	149	153
>tr|E2R868|E2R868_CANLF [histone H4]-N-methyl-L-lysine20 N-methyltransferase KMT5B OS=Canis lupus familiaris OX=9615 GN=KMT5B PE=4 SV=3	0.25	0.23	35.30	55400080.5	10	3	2	1.81	885	7704
>sp|Q28895|NPC2_CANLF NPC intracellular cholesterol transporter 2 OS=Canis lupus familiaris OX=9615 GN=NPC2 PE=2 SV=1	6.13	3.42	364.80	54117000.6	13	2	0	24.16	149	153
>tr|Q9XSV4|Q9XSV4_CANLF CE10 protein OS=Canis lupus familiaris OX=9615 GN=ce10 PE=2 SV=1	5.17	4.02	301.80	53971451.9	25	3	0	12.73	110	41542
>tr|Q9XSV4|Q9XSV4_CANLF CE10 protein OS=Canis lupus familiaris OX=9615 GN=ce10 PE=2 SV=1	3.86	2.31	328.50	53745445.6	27	3	0	12.73	110	41542
>tr|A0A5F4C0S7|A0A5F4C0S7_CANLF HEAT repeat containing 5A OS=Canis lupus familiaris OX=9615 GN=HEATR5A PE=3 SV=1	0.31	0.13	55.20	51558300.2	17	1	0	0.25	1995	1753
>tr|A0A5F4C9T7|A0A5F4C9T7_CANLF Telomerase associated protein 1 OS=Canis lupus familiaris OX=9615 GN=TEP1 PE=4 SV=1	0.10	0.00	32.60	50450702.5	3	1	0	0.16	2507	891
>tr|J9NYC7|J9NYC7_CANLF Dynein axonemal heavy chain 12 OS=Canis lupus familiaris OX=9615 GN=DNAH12 PE=3 SV=1	1.46	1.42	123.50	45928018.2	3	1	0	0.33	3960	15992
>sp|P62286|ASPM_CANLF Abnormal spindle-like microcephaly-associated protein homolog OS=Canis lupus familiaris OX=9615 GN=ASPM PE=2 SV=2	0.33	0.13	114.80	45431255.6	17	2	0	0.23	3469	677
>tr|E2R824|E2R824_CANLF Zinc finger protein 518B OS=Canis lupus familiaris OX=9615 GN=ZNF518B PE=4 SV=3	0.15	0.02	115.70	45048357.4	41	2	1	1.30	1000	24468
>tr|A0A5F4C618|A0A5F4C618_CANLF Basonuclin 1 OS=Canis lupus familiaris OX=9615 GN=BNC1 PE=4 SV=1	0.38	0.34	104.80	44256001.8	3	1	0	1.66	965	2022
>tr|E2QXA7|E2QXA7_CANLF LETM1 domain containing 1 OS=Canis lupus familiaris OX=9615 GN=LETMD1 PE=4 SV=2	0.94	0.90	129.60	43986142.8	3	1	1	2.95	373	9332
>sp|E2RKA8|RL32_CANLF 60S ribosomal protein L32 OS=Canis lupus familiaris OX=9615 GN=RPL32 PE=1 SV=1	0.29	0.03	110.20	41596467.8	17	1	0	2.22	135	275
>tr|A0A5F4DMP2|A0A5F4DMP2_CANLF Heterogeneous nuclear ribonucleoprotein M OS=Canis lupus familiaris OX=9615 GN=HNRNPM PE=4 SV=1	0.53	0.39	102.80	41232650.6	8	1	0	0.55	731	1228
>sp|Q9GL25|ESPB1_CANLF Epididymal sperm-binding protein 1 OS=Canis lupus familiaris OX=9615 GN=ELSPBP1 PE=1 SV=1	0.44	0.38	200.00	41095353.0	4	1	0	6.94	245	36
>sp|Q9GL25|ESPB1_CANLF Epididymal sperm-binding protein 1 OS=Canis lupus familiaris OX=9615 GN=ELSPBP1 PE=1 SV=1	1.26	1.20	106.20	40690435.7	4	1	0	6.94	245	36
>tr|A0A5F4D9Z7|A0A5F4D9Z7_CANLF Zinc finger FYVE-type containing 19 OS=Canis lupus familiaris OX=9615 GN=ZFYVE19 PE=4 SV=1	0.12	0.12	96.80	40635378.9	2	1	0	1.53	392	13875
>tr|E2RG75|E2RG75_CANLF Inactive ribonuclease-like protein 9 OS=Canis lupus familiaris OX=9615 GN=RNASE9 PE=3 SV=2	6.87	3.34	433.40	40242533.6	9	3	1	8.08	198	41734
>tr|A0A5K1V5T8|A0A5K1V5T8_CANLF Zinc finger and BTB domain containing 49 OS=Canis lupus familiaris OX=9615 GN=ZBTB49 PE=4 SV=1	0.25	0.21	68.40	40135537.3	3	1	0	1.47	543	5803
>tr|A0A5K1V0D8|A0A5K1V0D8_CANLF Sulfatase 2 OS=Canis lupus familiaris OX=9615 GN=SULF2 PE=3 SV=1	0.16	0.02	128.40	40089978.0	9	1	0	0.35	859	1192

>tr|J9NZH4|J9NZH4_CANLF NTR domain-containing protein OS=Canis lupus familiaris OX=9615 GN=LOC102154527 PE=3 SV=2	1.29	1.23	133.40	39923173.6	4	1	0	8.60	221	39505
>tr|A0A5F4CPE1|A0A5F4CPE1_CANLF Major facilitator superfamily domain containing 14B OS=Canis lupus familiaris OX=9615 GN=MFSD14B PE=4 SV=1	0.10	0.00	83.60	38928765.7	2	1	0	3.71	485	28321
>tr|Q9XSV4|Q9XSV4_CANLF CE10 protein OS=Canis lupus familiaris OX=9615 GN=ce10 PE=2 SV=1	3.86	1.63	281.40	38452000.8	16	3	0	14.55	110	41542
>tr|A0A5F4D7J3|A0A5F4D7J3_CANLF Non-specific serine/threonine protein kinase OS=Canis lupus familiaris OX=9615 GN=CDC42BPA PE=3 SV=1	1.12	1.03	283.40	37440878.0	6	1	0	0.22	1794	1069
>tr|E2RMC9|E2RMC9_CANLF DUF4515 domain-containing protein OS=Canis lupus familiaris OX=9615 GN=LOC100688167 PE=4 SV=3	0.15	0.04	182.00	36108884.8	11	2	0	2.08	432	29175
>tr|F1PB65|F1PB65_CANLF RAD54 like 2 OS=Canis lupus familiaris OX=9615 GN=RAD54L2 PE=3 SV=2	0.30	0.28	158.60	35733148.2	2	1	0	0.55	1467	11575
>tr|J9NS29|J9NS29_CANLF Cystatin domain-containing protein OS=Canis lupus familiaris OX=9615 GN=LOC607874 PE=4 SV=2	0.46	0.46	202.40	35486277.3	2	1	0	6.39	313	30016
>tr|E2RRE4|E2RRE4_CANLF Netrin 5 OS=Canis lupus familiaris OX=9615 GN=NTN5 PE=4 SV=3	0.25	0.13	133.90	35168475.2	7	1	0	1.02	489	13410
>tr|A0A5F4CQY7|A0A5F4CQY7_CANLF Neuregulin 2 OS=Canis lupus familiaris OX=9615 GN=NRG2 PE=4 SV=1	0.26	0.02	39.20	35061327.2	19	1	0	0.62	802	13353
>tr|F1PGF9|F1PGF9_CANLF Rho guanine nucleotide exchange factor 26 OS=Canis lupus familiaris OX=9615 GN=ARHGEF26 PE=4 SV=3	0.74	0.70	304.40	34821887.7	3	1	0	0.84	594	22876
>tr|J9NTK2|J9NTK2_CANLF J domain-containing protein OS=Canis lupus familiaris OX=9615 GN=DNAJC12 PE=4 SV=2	0.16	0.12	138.00	34327155.4	3	1	0	4.72	106	2310
>tr|E2QW13|E2QW13_CANLF Inhibin subunit beta A OS=Canis lupus familiaris OX=9615 GN=INHBA PE=3 SV=1	0.54	0.48	70.50	34234131.7	4	1	1	2.59	424	5794
>tr|J9NTK2|J9NTK2_CANLF J domain-containing protein OS=Canis lupus familiaris OX=9615 GN=DNAJC12 PE=4 SV=2	0.10	0.00	104.30	33689209.6	2	1	0	4.72	106	2310
>tr|A0A5F4D1D2|A0A5F4D1D2_CANLF Protein-tyrosine-phosphatase OS=Canis lupus familiaris OX=9615 GN=PTPRD PE=3 SV=1	2.53	2.52	63.00	33485086.8	2	1	0	0.60	1828	2208
>tr|F1PGF9|F1PGF9_CANLF Rho guanine nucleotide exchange factor 26 OS=Canis lupus familiaris OX=9615 GN=ARHGEF26 PE=4 SV=3	1.34	1.28	294.10	33161554.5	4	1	0	0.84	594	22876
>sp|Q8WN22|PRKDC_CANLF DNA-dependent protein kinase catalytic subunit OS=Canis lupus familiaris OX=9615 GN=PRKDC PE=2 SV=1	1.90	1.18	185.40	33005844.1	6	3	0	0.22	4144	338
>sp|Q9GL25|ESPB1_CANLF Epididymal sperm-binding protein 1 OS=Canis lupus familiaris OX=9615 GN=ELSPBP1 PE=1 SV=1	3.55	2.26	137.40	32822569.4	5	2	0	11.43	245	36
>tr|F1Q432|F1Q432_CANLF Tenascin XB OS=Canis lupus familiaris OX=9615 GN=TNXB PE=3 SV=3	0.53	0.49	279.50	32357737.3	3	1	0	0.13	3966	30371
>tr|J9P487|J9P487_CANLF Serine/threonine-protein phosphatase 2A 55 kDa regulatory subunit B OS=Canis lupus familiaris OX=9615 GN=PPP2R2D PE=3 SV=2	0.26	0.24	15.80	32338413.4	2	1	0	7.96	427	12816
>tr|F6UZ16|F6UZ16_CANLF Zinc finger BED-type containing 9 OS=Canis lupus familiaris OX=9615 GN=ZBED9 PE=4 SV=2	0.21	0.17	23.90	32090101.7	3	2	2	1.51	1327	42064
>tr|A0A5F4CS27|A0A5F4CS27_CANLF Abhydrolase domain containing 14B OS=Canis lupus familiaris OX=9615 GN=ABHD14B PE=4 SV=1	0.10	0.00	126.30	31942504.9	2	1	0	2.56	234	21786
>tr|F1PFZ5|F1PFZ5_CANLF Milk fat globule EGF and factor V/VIII domain containing OS=Canis lupus familiaris OX=9615 GN=MFGE8 PE=4 SV=3	0.49	0.43	157.40	31552216.5	4	1	0	1.87	428	7079

>tr|Q9XSV4|Q9XSV4_CANLF CE10 protein OS=Canis lupus familiaris OX=9615 GN=ce10 PE=2 SV=1	3.25	1.57	293.60	31290614.7	14	3	0	14.55	110	41542
>tr|A0A5F4DBL7|A0A5F4DBL7_CANLF LDL receptor related protein 1 OS=Canis lupus familiaris OX=9615 GN=LRP1 PE=3 SV=1	0.15	0.13	118.90	31037917.9	2	1	0	0.34	4133	14011
>tr|J9NS29|J9NS29_CANLF Cystatin domain-containing protein OS=Canis lupus familiaris OX=9615 GN=LOC607874 PE=4 SV=2	0.32	0.28	111.90	30625104.5	3	1	0	4.79	313	30016
>tr|A0A5F4CCD0|A0A5F4CCD0_CANLF Cysteine rich secretory protein 2 OS=Canis lupus familiaris OX=9615 GN=CRISP2 PE=3 SV=1	2.90	1.75	273.80	30582918.9	7	2	0	7.07	311	11017
>tr|J9P4U7|J9P4U7_CANLF Alpha-1,6-mannosyl-glycoprotein 6-beta-N-acetylglucosaminyltransferase OS=Canis lupus familiaris OX=9615 GN=MGAT5B PE=3 SV=2	0.17	0.13	65.90	30557673.3	3	1	1	1.39	792	13595
>tr|A0A5F4CCD0|A0A5F4CCD0_CANLF Cysteine rich secretory protein 2 OS=Canis lupus familiaris OX=9615 GN=CRISP2 PE=3 SV=1	2.13	2.05	287.00	30267844.4	5	1	0	2.25	311	11017
>tr|E2RIH1|E2RIH1_CANLF DOP1 leucine zipper like protein B OS=Canis lupus familiaris OX=9615 GN=DOP1B PE=3 SV=3	0.81	0.56	37.60	30256843.0	9	2	0	1.42	2259	7751
>tr|E2R6E0|E2R6E0_CANLF Lipocln_cytosolic_FA-bd_dom domain-containing protein OS=Canis lupus familiaris OX=9615 GN=LCNL1 PE=3 SV=2	3.03	2.97	193.10	30089137.0	4	1	0	3.01	299	1932
>tr|E2RRP3|E2RRP3_CANLF LIM homeobox 5 OS=Canis lupus familiaris OX=9615 GN=LHX5 PE=4 SV=1	0.25	0.23	161.80	30034913.6	2	1	0	2.24	402	19607
>tr|A0A5F4CPU3|A0A5F4CPU3_CANLF SEC24 homolog D, COPII coat complex component OS=Canis lupus familiaris OX=9615 GN=SEC24D PE=3 SV=1	0.20	0.13	34.30	29986999.0	10	1	0	0.53	946	2125
>tr|J9P3H8|J9P3H8_CANLF ATM interactor OS=Canis lupus familiaris OX=9615 GN=ATMIN PE=4 SV=2	0.20	0.10	44.00	28658261.0	11	1	0	0.58	863	882
>tr|F1PGF9|F1PGF9_CANLF Rho guanine nucleotide exchange factor 26 OS=Canis lupus familiaris OX=9615 GN=ARHGEF26 PE=4 SV=3	1.28	1.22	309.00	28259485.7	4	1	0	0.84	594	22876
>sp|Q9GL25|ESPB1_CANLF Epididymal sperm-binding protein 1 OS=Canis lupus familiaris OX=9615 GN=ELSPBP1 PE=1 SV=1	0.73	0.65	192.50	27935980.9	5	1	0	6.94	245	36
>tr|A0A5F4DHH0|A0A5F4DHH0_CANLF ATP binding cassette subfamily A member 1 OS=Canis lupus familiaris OX=9615 GN=ABCA1 PE=4 SV=1	0.28	0.02	23.80	27929620.9	14	1	0	0.23	2175	3709
>tr|E2RCT1|E2RCT1_CANLF WAP domain-containing protein OS=Canis lupus familiaris OX=9615 PE=4 SV=2	0.62	0.28	173.60	27296972.7	6	3	0	24.14	116	21717
>tr|A0A5F4BZ12|A0A5F4BZ12_CANLF Importin 11 OS=Canis lupus familiaris OX=9615 GN=IPO11 PE=4 SV=1	1.21	1.17	268.90	26980253.9	3	1	0	0.72	968	20170
>sp|P01002|IPSG_CANLF Double-headed protease inhibitor, submandibular gland OS=Canis lupus familiaris OX=9615 PE=1 SV=1	2.02	1.96	210.90	26854855.2	4	1	0	7.83	115	434
>tr|A0A5F4C730|A0A5F4C730_CANLF Semaphorin 4D OS=Canis lupus familiaris OX=9615 GN=SEMA4D PE=3 SV=1	0.35	0.31	202.40	26436220.2	3	1	0	0.28	1067	1802
>tr|A0A5F4D3J6|A0A5F4D3J6_CANLF ATP binding cassette subfamily A member 12 OS=Canis lupus familiaris OX=9615 GN=ABCA12 PE=4 SV=1	0.44	0.34	106.30	25381153.8	6	1	1	0.31	2614	2871
>sp|O46607|GPX5_CANLF Epididymal secretory glutathione peroxidase OS=Canis lupus familiaris OX=9615 GN=GPX5 PE=2 SV=1	0.41	0.25	218.20	24941059.5	3	2	0	8.60	221	564
>sp|Q28895|NPC2_CANLF NPC intracellular cholesterol transporter 2 OS=Canis lupus familiaris OX=9615 GN=NPC2 PE=2 SV=1	2.86	2.84	294.90	24871865.9	2	1	0	15.44	149	153
>tr|A0A5F4CSE7|A0A5F4CSE7_CANLF TYR_PHOSPHATASE_2 domain-containing protein OS=Canis lupus familiaris OX=9615 GN=PTP4A1 PE=4 SV=1	0.10	0.00	63.40	24837297.8	2	1	1	5.20	173	25182

>tr|F1PFZ5|F1PFZ5_CANLF Milk fat globule EGF and factor V/VIII domain containing OS=Canis lupus familiaris OX=9615 GN=MFGE8 PE=4 SV=3	2.29	2.25	240.70	24688641.3	3	1	0	1.87	428	7079
>tr|E2R6E0|E2R6E0_CANLF Lipocln_cytosolic_FA-bd_dom domain-containing protein OS=Canis lupus familiaris OX=9615 GN=LCNL1 PE=3 SV=2	1.42	1.36	188.70	24616683.7	4	1	0	3.01	299	1932
>sp|F1PRN2|MYO1D_CANLF Unconventional myosin-Id OS=Canis lupus familiaris OX=9615 GN=MYO1D PE=1 SV=2	0.37	0.21	206.80	24612720.7	9	1	0	0.30	1006	763
>tr|Q9XSV4|Q9XSV4_CANLF CE10 protein OS=Canis lupus familiaris OX=9615 GN=ce10 PE=2 SV=1	2.59	1.77	249.10	24116860.5	12	3	0	14.55	110	41542
>tr|A0A5F4DM58|A0A5F4DM58_CANLF Zinc finger MYM-type containing 2 OS=Canis lupus familiaris OX=9615 GN=ZMYM2 PE=4 SV=1	0.13	0.09	98.20	23746174.5	3	1	1	0.85	1295	4956
>tr|A0A5F4D3J6|A0A5F4D3J6_CANLF ATP binding cassette subfamily A member 12 OS=Canis lupus familiaris OX=9615 GN=ABCA12 PE=4 SV=1	0.32	0.22	118.20	23735100.0	6	1	1	0.31	2614	2871
>tr|E2RG75|E2RG75_CANLF Inactive ribonuclease-like protein 9 OS=Canis lupus familiaris OX=9615 GN=RNASE9 PE=3 SV=2	8.73	4.37	420.10	23576522.8	6	3	1	8.08	198	41734
>tr|F1PS80|F1PS80_CANLF Protein phosphatase 4 regulatory subunit 1 OS=Canis lupus familiaris OX=9615 GN=PPP4R1 PE=4 SV=3	0.11	0.07	50.30	23137086.9	3	1	0	0.56	887	2666
>sp|O46607|GPX5_CANLF Epididymal secretory glutathione peroxidase OS=Canis lupus familiaris OX=9615 GN=GPX5 PE=2 SV=1	1.43	1.10	171.40	23094427.1	2	2	0	8.60	221	564
>tr|A0A5F4CQY7|A0A5F4CQY7_CANLF Neuregulin 2 OS=Canis lupus familiaris OX=9615 GN=NRG2 PE=4 SV=1	0.10	0.02	85.40	23042448.7	35	1	0	0.62	802	13353
>sp|Q28895|NPC2_CANLF NPC intracellular cholesterol transporter 2 OS=Canis lupus familiaris OX=9615 GN=NPC2 PE=2 SV=1	6.55	2.18	337.10	22906105.3	14	4	0	38.93	149	153
>tr|A0A5K1V0D8|A0A5K1V0D8_CANLF Sulfatase 2 OS=Canis lupus familiaris OX=9615 GN=SULF2 PE=3 SV=1	0.66	0.56	184.50	22817089.7	6	1	0	0.35	859	1192
>tr|A0A5F4DHD3|A0A5F4DHD3_CANLF Sorbin and SH3 domain containing 2 OS=Canis lupus familiaris OX=9615 GN=SORBS2 PE=4 SV=1	0.17	0.13	9.40	22743143.6	3	1	0	0.41	1219	1177
>sp|O97758|ZO1_CANLF Tight junction protein ZO-1 OS=Canis lupus familiaris OX=9615 GN=TJP1 PE=1 SV=1	0.17	0.13	9.40	22743143.6	3	1	0	0.28	1769	139
>tr|A0A5F4BT89|A0A5F4BT89_CANLF Olfactory receptor OS=Canis lupus familiaris OX=9615 GN=OR5W6 PE=3 SV=1	0.10	0.00	24.70	22103820.5	1	1	1	6.95	302	29923
>tr|E2R4A0|E2R4A0_CANLF Actin-like protein 7B OS=Canis lupus familiaris OX=9615 GN=ACTL7B PE=3 SV=2	0.15	0.13	160.30	21951113.9	2	1	1	2.42	454	3066
>tr|A0A5F4DGS3|A0A5F4DGS3_CANLF Polypeptide N-acetylgalactosaminyltransferase OS=Canis lupus familiaris OX=9615 GN=GALNT18 PE=3 SV=1	0.35	0.33	101.50	21316920.7	2	1	0	0.99	607	14798
>sp|P01002|IPSG_CANLF Double-headed protease inhibitor, submandibular gland OS=Canis lupus familiaris OX=9615 PE=1 SV=1	0.27	0.24	182.90	21013934.2	3	1	0	7.83	115	434
>tr|E2RA00|E2RA00_CANLF Leucine rich repeat containing 8 VRAC subunit D OS=Canis lupus familiaris OX=9615 GN=LRRC8D PE=3 SV=1	0.10	0.00	41.00	20869413.5	1	1	1	1.98	858	42697
>tr|F1PRL1|F1PRL1_CANLF Dppa2_A domain-containing protein OS=Canis lupus familiaris OX=9615 PE=4 SV=2	0.27	0.21	38.30	20672121.5	4	1	0	2.79	179	17222
>tr|Q9XSV4|Q9XSV4_CANLF CE10 protein OS=Canis lupus familiaris OX=9615 GN=ce10 PE=2 SV=1	0.75	0.52	222.10	20375401.0	6	3	0	14.55	110	41542
>tr|E2RR09|E2RR09_CANLF Sodium channel protein OS=Canis lupus familiaris OX=9615 GN=SCN4A PE=3 SV=2	0.33	0.29	106.10	20341988.1	3	1	1	0.33	1837	22889

>tr|J9P3H8|J9P3H8_CANLF ATM interactor OS=Canis lupus familiaris OX=9615 GN=ATMIN PE=4 SV=2	0.48	0.02	40.60	20200389.4	24	1	0	0.58	863	882
>tr|A0A5F4D9S5|A0A5F4D9S5_CANLF Hyaluronoglucosaminidase OS=Canis lupus familiaris OX=9615 GN=CEMIP PE=3 SV=1	1.23	1.19	167.40	20127352.9	3	1	0	0.24	1684	9775
>tr|J9NS29|J9NS29_CANLF Cystatin domain-containing protein OS=Canis lupus familiaris OX=9615 GN=LOC607874 PE=4 SV=2	1.18	1.16	190.10	20017522.7	2	1	0	6.39	313	30016
>tr|A0A5F4D7Y5|A0A5F4D7Y5_CANLF Pleckstrin homology, MyTH4 and FERM domain containing H1 OS=Canis lupus familiaris OX=9615 GN=PLEKHH1 PE=4 SV=1	0.96	0.93	218.90	19898063.0	3	1	0	0.30	1342	5979
>tr|F1PQM0|F1PQM0_CANLF BAH domain and coiled-coil containing 1 OS=Canis lupus familiaris OX=9615 GN=BAHCC1 PE=4 SV=3	0.61	0.57	154.00	19044310.8	3	1	1	0.43	2565	10908
>tr|A0A5F4CDG4|A0A5F4CDG4_CANLF Protein phosphatase 1 regulatory subunit 32 OS=Canis lupus familiaris OX=9615 GN=PPP1R32 PE=4 SV=1	0.51	0.51	82.60	19036886.0	1	1	1	2.56	429	3606
>tr|A0A5F4BQ68|A0A5F4BQ68_CANLF Interferon-induced GTP-binding protein Mx1 OS=Canis lupus familiaris OX=9615 GN=MX2 PE=3 SV=1	0.10	0.06	102.50	19008940.4	3	2	2	2.47	729	13816
>tr|A0A5F4DKY4|A0A5F4DKY4_CANLF Dehydrogenase E1 and transketolase domain containing 1 OS=Canis lupus familiaris OX=9615 GN=DHTKD1 PE=3 SV=1	0.18	0.14	81.00	18683652.4	3	1	0	2.40	834	5545
>tr|F1Q432|F1Q432_CANLF Tenascin XB OS=Canis lupus familiaris OX=9615 GN=TNXB PE=3 SV=3	0.34	0.32	188.40	18566850.4	2	1	0	0.13	3966	30371
>sp|P23685|NAC1_CANLF Sodium/calcium exchanger 1 OS=Canis lupus familiaris OX=9615 GN=SLC8A1 PE=1 SV=1	0.25	0.21	133.30	18288613.1	3	1	0	1.03	970	764
>tr|A0A5F4CGE7|A0A5F4CGE7_CANLF C3 and PZP like alpha-2-macroglobulin domain containing 8 OS=Canis lupus familiaris OX=9615 GN=CPAMD8 PE=3 SV=1	0.35	0.33	19.40	18272240.6	2	1	1	1.07	1773	5057
>tr|E2RTL2|E2RTL2_CANLF Tubulin tyrosine ligase like 6 OS=Canis lupus familiaris OX=9615 GN=TTLL6 PE=4 SV=3	0.25	0.21	35.60	18218161.4	3	1	0	0.60	827	2703
>sp|A2IBY8|MIP_CANLF Lens fiber major intrinsic protein OS=Canis lupus familiaris OX=9615 GN=MIP PE=2 SV=1	0.64	0.55	81.00	18104535.5	6	1	0	1.90	263	112
>tr|E2RJF6|E2RJF6_CANLF Mortality factor 4 like 1 OS=Canis lupus familiaris OX=9615 GN=MORF4L1 PE=4 SV=3	0.15	0.13	56.00	18084682.8	2	1	0	4.56	439	18584
>tr|E2R186|E2R186_CANLF Fibroblast growth factor receptor OS=Canis lupus familiaris OX=9615 GN=FGFR1 PE=3 SV=3	0.10	0.02	74.70	17701554.6	10	1	0	0.59	853	9797
>tr|J9NZJ2|J9NZJ2_CANLF Protein Wnt OS=Canis lupus familiaris OX=9615 GN=WNT6 PE=3 SV=2	0.23	0.21	214.90	17561941.3	2	1	0	1.47	339	11134
>tr|A0A5F4CYC6|A0A5F4CYC6_CANLF Leucine zipper protein 1 OS=Canis lupus familiaris OX=9615 GN=LUZP1 PE=4 SV=1	0.21	0.17	20.40	17508851.3	3	1	0	0.56	1074	30023
>tr|A0A5F4CS87|A0A5F4CS87_CANLF STE20 related adaptor alpha OS=Canis lupus familiaris OX=9615 GN=STRADA PE=4 SV=1	0.29	0.29	138.70	16951984.1	1	1	0	2.08	336	14044
>tr|E2R0S2|E2R0S2_CANLF [Histone H3]-trimethyl-L-lysine(4) demethylase OS=Canis lupus familiaris OX=9615 GN=KDM5C PE=3 SV=3	0.54	0.53	105.70	16949610.2	2	1	0	0.46	1523	25191
>tr|A0A5F4CKF6|A0A5F4CKF6_CANLF [Heparan sulfate]-glucosamine N-sulfotransferase OS=Canis lupus familiaris OX=9615 GN=NDST3 PE=3 SV=1	0.10	0.07	26.60	16917822.0	2	1	0	1.49	872	7557
>tr|F1PB68|F1PB68_CANLF Olfactomedin 4 OS=Canis lupus familiaris OX=9615 GN=OLFM4 PE=4 SV=3	0.23	0.19	92.40	16620344.0	3	1	0	4.40	477	17246
>tr|F1P6D8|F1P6D8_CANLF Dynein axonemal heavy chain 5 OS=Canis lupus familiaris OX=9615 GN=DNAH5 PE=3 SV=3	0.10	0.00	105.40	16532748.4	1	1	0	0.19	4620	37739

>tr|A0A5F4DJ37|A0A5F4DJ37_CANLF KRAB domain-containing protein OS=Canis lupus familiaris OX=9615 PE=4 SV=1	0.14	0.14	112.00	16515676.6	1	1	0	19.64	56	37151
>tr|F1PPP9|F1PPP9_CANLF Family with sequence similarity 135 member A OS=Canis lupus familiaris OX=9615 GN=FAM135A PE=3 SV=3	0.33	0.10	169.80	16443805.1	16	1	0	1.22	1399	6815
>tr|E2R4W0|E2R4W0_CANLF Anaphase-promoting complex subunit 11 OS=Canis lupus familiaris OX=9615 GN=ANAPC11 PE=3 SV=2	0.25	0.23	158.90	16436251.7	2	1	0	1.75	458	23846
>tr|A0A5F4D9S5|A0A5F4D9S5_CANLF Hyaluronoglucosaminidase OS=Canis lupus familiaris OX=9615 GN=CEMIP PE=3 SV=1	0.97	0.95	162.80	15884679.8	2	1	0	0.24	1684	9775
>tr|F1PL54|F1PL54_CANLF Olfactory receptor OS=Canis lupus familiaris OX=9615 GN=OR52E2B PE=3 SV=2	0.10	0.03	78.80	15785972.3	2	1	1	2.25	311	5515
>tr|E2R0G7|E2R0G7_CANLF DNA ligase OS=Canis lupus familiaris OX=9615 GN=LIG4 PE=3 SV=1	0.14	0.10	45.30	15723919.3	3	1	0	0.55	911	29979
>tr|A0A5F4CCD0|A0A5F4CCD0_CANLF Cysteine rich secretory protein 2 OS=Canis lupus familiaris OX=9615 GN=CRISP2 PE=3 SV=1	0.67	0.63	190.00	15676538.4	3	1	0	2.25	311	11017
>tr|F1PQU5|F1PQU5_CANLF Synaptic vesicle glycoprotein 2C OS=Canis lupus familiaris OX=9615 GN=SV2C PE=3 SV=2	0.60	0.58	75.90	15675333.5	2	1	0	2.76	724	12575
>tr|E2RNZ9|E2RNZ9_CANLF Tudor domain containing 9 OS=Canis lupus familiaris OX=9615 GN=TDRD9 PE=3 SV=3	0.19	0.17	21.10	15671460.2	2	1	0	0.59	1014	6293
>tr|F1PQN7|F1PQN7_CANLF Ankyrin repeat and BTB domain containing 2 OS=Canis lupus familiaris OX=9615 GN=ABTB2 PE=4 SV=2	0.18	0.16	133.20	15514641.0	2	1	0	0.67	1188	42888
>tr|F1PJY1|F1PJY1_CANLF Mannosyl-glycoprotein endo-beta-N-acetylglucosaminidase OS=Canis lupus familiaris OX=9615 GN=ENGASE PE=3 SV=3	0.70	0.25	176.20	15431288.8	16	2	1	1.74	690	32761
>tr|E2RRE4|E2RRE4_CANLF Netrin 5 OS=Canis lupus familiaris OX=9615 GN=NTN5 PE=4 SV=3	0.13	0.09	110.10	14965330.4	3	1	0	1.02	489	13410
>tr|Q9XSV4|Q9XSV4_CANLF CE10 protein OS=Canis lupus familiaris OX=9615 GN=ce10 PE=2 SV=1	1.24	0.87	210.70	14879128.5	7	2	0	11.82	110	41542
>tr|F1PQU5|F1PQU5_CANLF Synaptic vesicle glycoprotein 2C OS=Canis lupus familiaris OX=9615 GN=SV2C PE=3 SV=2	0.59	0.57	90.90	14800364.2	2	1	0	2.76	724	12575
>tr|F1PFP6|F1PFP6_CANLF Matrix metallopeptidase 12 OS=Canis lupus familiaris OX=9615 GN=MMP12 PE=3 SV=3	0.37	0.37	80.20	14780824.2	1	1	0	2.44	491	24324
>tr|A0A5F4CU85|A0A5F4CU85_CANLF Leucyl-tRNA synthetase OS=Canis lupus familiaris OX=9615 GN=LARS1 PE=3 SV=1	0.13	0.13	47.70	14489099.0	1	1	0	1.01	1187	2068
>tr|A0A5F4D7Y5|A0A5F4D7Y5_CANLF Pleckstrin homology, MyTH4 and FERM domain containing H1 OS=Canis lupus familiaris OX=9615 GN=PLEKHH1 PE=4 SV=1	0.54	0.50	180.10	14432697.4	3	1	0	0.30	1342	5979
>tr|A0A5F4D9Z7|A0A5F4D9Z7_CANLF Zinc finger FYVE-type containing 19 OS=Canis lupus familiaris OX=9615 GN=ZFYVE19 PE=4 SV=1	0.19	0.17	100.30	14347306.2	2	1	0	1.53	392	13875
>sp|P21842|CMA1_CANLF Chymase OS=Canis lupus familiaris OX=9615 GN=CMA1 PE=1 SV=1	0.45	0.41	92.60	14291715.1	3	1	0	0.80	249	34
>tr|E2RJR2|E2RJR2_CANLF NADH:ubiquinone oxidoreductase subunit A9 OS=Canis lupus familiaris OX=9615 GN=NDUFA9 PE=4 SV=2	0.10	0.02	31.90	14129329.5	3	1	1	5.31	377	3530
>tr|E2RJR2|E2RJR2_CANLF NADH:ubiquinone oxidoreductase subunit A9 OS=Canis lupus familiaris OX=9615 GN=NDUFA9 PE=4 SV=2	0.20	0.20	5.90	13993368.2	1	1	1	5.31	377	3530
>tr|E2R967|E2R967_CANLF Tectonin beta-propeller repeat-containing protein 1 OS=Canis lupus familiaris OX=9615 GN=TECPR1 PE=3 SV=2	0.24	0.24	115.20	13869261.3	1	1	0	1.00	1303	18227

>tr|J9NTK2|J9NTK2_CANLF J domain-containing protein OS=Canis lupus familiaris OX=9615 GN=DNAJC12 PE=4 SV=2	0.23	0.21	160.30	13652893.2	2	1	0	4.72	106	2310
>tr|A0A5F4CF57|A0A5F4CF57_CANLF WD repeat domain 90 OS=Canis lupus familiaris OX=9615 GN=WDR90 PE=4 SV=1	0.10	0.02	116.40	13631870.4	1	1	0	0.26	2322	3876
>tr|A0A5F4DFX0|A0A5F4DFX0_CANLF Exportin-T OS=Canis lupus familiaris OX=9615 GN=XPOT PE=3 SV=1	0.26	0.18	141.70	13567998.2	5	1	0	0.75	938	2329
>sp|Q28895|NPC2_CANLF NPC intracellular cholesterol transporter 2 OS=Canis lupus familiaris OX=9615 GN=NPC2 PE=2 SV=1	6.65	6.35	427.00	12973006.0	16	1	0	15.44	149	153
>sp|P49822|ALBU_CANLF Albumin OS=Canis lupus familiaris OX=9615 GN=ALB PE=1 SV=3	9.81	3.92	287.80	12969291.6	13	6	1	11.51	608	490
>tr|F1P8J6|F1P8J6_CANLF RNA helicase OS=Canis lupus familiaris OX=9615 GN=DDX55 PE=3 SV=3	0.35	0.35	138.80	12899574.5	1	1	0	1.58	568	8934
>tr|J9NS29|J9NS29_CANLF Cystatin domain-containing protein OS=Canis lupus familiaris OX=9615 GN=LOC607874 PE=4 SV=2	0.85	0.76	194.70	12812353.1	2	2	0	5.11	313	30016
>tr|A0A5F4C9T7|A0A5F4C9T7_CANLF Telomerase associated protein 1 OS=Canis lupus familiaris OX=9615 GN=TEP1 PE=4 SV=1	0.61	0.57	214.40	12659890.7	3	1	0	0.16	2507	891
>sp|Q28346|RL4_CANLF 60S ribosomal protein L4 OS=Canis lupus familiaris OX=9615 GN=RPL4 PE=1 SV=2	0.35	0.31	47.90	12659890.7	3	1	0	0.48	421	168
>tr|A0A5F4CCD0|A0A5F4CCD0_CANLF Cysteine rich secretory protein 2 OS=Canis lupus familiaris OX=9615 GN=CRISP2 PE=3 SV=1	0.63	0.61	84.30	12651840.7	2	1	0	4.82	311	11017
>tr|F1PCU5|F1PCU5_CANLF Methyltransf_11 domain-containing protein OS=Canis lupus familiaris OX=9615 GN=LOC480074 PE=4 SV=3	0.21	0.21	51.60	12622318.7	1	1	0	2.86	630	1484
>tr|E2RF35|E2RF35_CANLF Calcium-transporting ATPase OS=Canis lupus familiaris OX=9615 GN=ATP2B2 PE=3 SV=2	0.60	0.58	13.10	12411099.2	2	1	0	2.49	1243	27151
>tr|A0A5F4BT89|A0A5F4BT89_CANLF Olfactory receptor OS=Canis lupus familiaris OX=9615 GN=OR5W6 PE=3 SV=1	0.10	0.01	91.80	12092954.6	2	1	1	6.95	302	29923
>tr|F1PFP6|F1PFP6_CANLF Matrix metallopeptidase 12 OS=Canis lupus familiaris OX=9615 GN=MMP12 PE=3 SV=3	0.30	0.28	63.70	12011017.0	2	1	0	2.44	491	24324
>tr|A0A5F4C4H6|A0A5F4C4H6_CANLF Biliverdin reductase A OS=Canis lupus familiaris OX=9615 GN=BLVRA PE=4 SV=1	0.10	0.07	106.70	11996867.4	2	1	0	3.49	401	15382
>tr|A0A5F4CRH0|A0A5F4CRH0_CANLF Ceruloplasmin OS=Canis lupus familiaris OX=9615 GN=CP PE=3 SV=1	0.36	0.34	128.00	11920452.2	2	1	0	1.41	1063	2330
>tr|J9P2D3|J9P2D3_CANLF Alpha kinase 3 OS=Canis lupus familiaris OX=9615 GN=ALPK3 PE=4 SV=2	0.32	0.24	190.30	11794790.9	5	1	0	0.31	1616	5104
>tr|A0A5F4CLI1|A0A5F4CLI1_CANLF Histone deacetylase 6 OS=Canis lupus familiaris OX=9615 GN=HDAC6 PE=4 SV=1	0.47	0.47	177.60	11764224.7	1	1	0	0.43	1175	4057
>tr|A0A5F4CXX5|A0A5F4CXX5_CANLF Carboxypeptidase OS=Canis lupus familiaris OX=9615 GN=CTSA PE=3 SV=1	0.36	0.36	133.00	11745399.0	1	1	0	2.27	485	3309
>tr|F1PBJ1|F1PBJ1_CANLF Methylcytosine dioxygenase TET OS=Canis lupus familiaris OX=9615 GN=TET3 PE=3 SV=2	0.10	0.02	66.10	11476539.9	4	1	0	0.28	1795	1529
>tr|J9NS29|J9NS29_CANLF Cystatin domain-containing protein OS=Canis lupus familiaris OX=9615 GN=LOC607874 PE=4 SV=2	0.35	0.33	146.80	11441647.2	2	1	0	6.39	313	30016
>tr|F1PHA9|F1PHA9_CANLF Motile sperm domain containing 2 OS=Canis lupus familiaris OX=9615 GN=MOSPD2 PE=4 SV=3	0.33	0.31	158.30	11391947.3	2	1	0	0.97	518	10201

>tr|F1PHV9|F1PHV9_CANLF Tetratricopeptide repeat domain 28 OS=Canis lupus familiaris OX=9615 GN=TTC28 PE=4 SV=2	1.17	1.17	51.50	11325246.8	1	1	0	0.77	2469	14555
>tr|A0A5F4D6G2|A0A5F4D6G2_CANLF SMG7 nonsense mediated mRNA decay factor OS=Canis lupus familiaris OX=9615 GN=SMG7 PE=4 SV=1	0.73	0.71	153.60	11313777.9	2	1	0	0.43	1175	2075
>tr|E2R3X1|E2R3X1_CANLF Rho GTPase activating protein 10 OS=Canis lupus familiaris OX=9615 GN=ARHGAP10 PE=4 SV=3	0.35	0.35	86.10	11254295.2	1	1	1	0.80	754	33789
>tr|J9P758|J9P758_CANLF Sorcin OS=Canis lupus familiaris OX=9615 GN=SRI PE=4 SV=2	0.57	0.57	155.50	11097658.5	1	1	0	2.30	348	7242
>tr|J9P5T2|J9P5T2_CANLF Non-specific serine/threonine protein kinase OS=Canis lupus familiaris OX=9615 GN=WNK3 PE=4 SV=2	0.10	0.01	55.10	11038696.0	4	1	0	0.22	2294	5229
>tr|A0A5F4DCA4|A0A5F4DCA4_CANLF Reverse transcriptase domain-containing protein OS=Canis lupus familiaris OX=9615 PE=4 SV=1	0.36	0.34	171.20	10943713.1	2	1	0	0.31	978	860
>tr|A0A5F4CGE0|A0A5F4CGE0_CANLF Ubiquitin protein ligase E3C OS=Canis lupus familiaris OX=9615 GN=UBE3C PE=4 SV=1	0.68	0.68	164.60	10766689.9	1	1	0	0.97	1238	9018
>tr|J9NT31|J9NT31_CANLF Thymocyte selection associated family member 2 OS=Canis lupus familiaris OX=9615 GN=THEMIS2 PE=3 SV=1	0.17	0.03	26.60	10738555.4	8	1	0	0.78	642	9120
>tr|E2RTL2|E2RTL2_CANLF Tubulin tyrosine ligase like 6 OS=Canis lupus familiaris OX=9615 GN=TTLL6 PE=4 SV=3	0.14	0.12	52.50	10731031.9	2	1	0	0.60	827	2703
>tr|A0A5F4CAH2|A0A5F4CAH2_CANLF RNA polymerase II subunit A C-terminal domain phosphatase OS=Canis lupus familiaris OX=9615 GN=CTDP1 PE=4 SV=1	0.19	0.19	101.50	10684469.1	1	1	0	0.74	945	14396
>sp|Q5TJE1|DAXX_CANLF Death domain-associated protein 6 OS=Canis lupus familiaris OX=9615 GN=DAXX PE=3 SV=1	0.46	0.44	155.70	10620387.5	2	1	0	0.41	737	429
>tr|F1PCL0|F1PCL0_CANLF DNL-type zinc finger OS=Canis lupus familiaris OX=9615 GN=DNLZ PE=4 SV=2	0.10	0.05	104.90	10584036.3	2	2	2	5.26	171	2400
>tr|I6MS72|I6MS72_CANLF Early lactation protein OS=Canis lupus familiaris OX=9615 GN=ELP PE=2 SV=1	0.13	0.13	34.40	10550443.2	1	1	0	21.00	100	41650
>tr|F1PS80|F1PS80_CANLF Protein phosphatase 4 regulatory subunit 1 OS=Canis lupus familiaris OX=9615 GN=PPP4R1 PE=4 SV=3	0.37	0.36	51.30	10433783.3	3	1	0	0.56	887	2666
>tr|A0A5F4D6G2|A0A5F4D6G2_CANLF SMG7 nonsense mediated mRNA decay factor OS=Canis lupus familiaris OX=9615 GN=SMG7 PE=4 SV=1	0.43	0.41	251.70	10417728.2	2	1	0	0.43	1175	2075
>tr|A0A5F4CGE0|A0A5F4CGE0_CANLF Ubiquitin protein ligase E3C OS=Canis lupus familiaris OX=9615 GN=UBE3C PE=4 SV=1	0.37	0.37	169.40	10413794.3	1	1	0	0.97	1238	9018
>tr|A0A5K1V0D8|A0A5K1V0D8_CANLF Sulfatase 2 OS=Canis lupus familiaris OX=9615 GN=SULF2 PE=3 SV=1	0.36	0.32	191.40	10406737.4	3	1	0	0.35	859	1192
>tr|A0A5F4DCA4|A0A5F4DCA4_CANLF Reverse transcriptase domain-containing protein OS=Canis lupus familiaris OX=9615 PE=4 SV=1	0.32	0.30	125.50	10372354.2	2	1	0	0.31	978	860
>sp|Q9XT60|SRY_CANLF Sex-determining region Y protein OS=Canis lupus familiaris OX=9615 GN=SRY PE=3 SV=1	0.17	0.03	27.80	10356106.0	8	1	0	2.27	220	347
>tr|J9NS29|J9NS29_CANLF Cystatin domain-containing protein OS=Canis lupus familiaris OX=9615 GN=LOC607874 PE=4 SV=2	0.80	0.78	178.20	10239116.2	2	1	0	6.39	313	30016
>tr|J9NT31|J9NT31_CANLF Thymocyte selection associated family member 2 OS=Canis lupus familiaris OX=9615 GN=THEMIS2 PE=3 SV=1	0.53	0.39	42.50	10171395.0	8	1	0	0.78	642	9120
>tr|A0A5F4C7W7|A0A5F4C7W7_CANLF Phosphatidylinositol-4,5-bisphosphate 3-kinase OS=Canis lupus familiaris OX=9615 GN=PIK3CD PE=3 SV=1	0.20	0.20	106.70	10088526.5	2	1	0	1.52	1051	18533
>tr|A0A5F4D7J3|A0A5F4D7J3_CANLF Non-specific serine/threonine protein kinase OS=Canis lupus familiaris OX=9615 GN=CDC42BPA PE=3 SV=1	1.04	1.04	189.50	10078853.4	2	2	1	0.39	1794	1069

>tr|F1Q2F6|F1Q2F6_CANLF 3-hydroxyacyl-[acyl-carrier-protein] dehydratase OS=Canis lupus familiaris OX=9615 GN=FASN PE=4 SV=3	0.80	0.78	173.90	10047512.1	2	1	0	0.40	2478	22936
>tr|A0A5F4DCA4|A0A5F4DCA4_CANLF Reverse transcriptase domain-containing protein OS=Canis lupus familiaris OX=9615 PE=4 SV=1	0.86	0.84	161.30	10047150.0	2	1	0	0.31	978	860
>tr|F6Y091|F6Y091_CANLF Olfactory receptor OS=Canis lupus familiaris OX=9615 GN=OR13C2 PE=3 SV=1	0.10	0.01	31.10	10029599.6	8	1	1	1.26	318	2117
>tr|F1PXW1|F1PXW1_CANLF Proteasome activator subunit 4 OS=Canis lupus familiaris OX=9615 GN=PSME4 PE=3 SV=3	1.02	1.02	164.60	9951003.8	2	2	1	0.74	1762	4363
>sp|P21842|CMA1_CANLF Chymase OS=Canis lupus familiaris OX=9615 GN=CMA1 PE=1 SV=1	1.17	1.15	100.40	9570435.8	2	1	0	0.80	249	34
>tr|A0A5F4D6L9|A0A5F4D6L9_CANLF Sacsin molecular chaperone OS=Canis lupus familiaris OX=9615 GN=SACS PE=4 SV=1	0.84	0.82	206.60	9407695.2	2	1	0	0.09	4500	1444
>tr|F1PLT8|F1PLT8_CANLF Sulfhydryl oxidase OS=Canis lupus familiaris OX=9615 GN=QSOX1 PE=3 SV=3	0.15	0.13	141.50	9369012.1	2	1	0	2.46	568	33056
>tr|Q9XSV4|Q9XSV4_CANLF CE10 protein OS=Canis lupus familiaris OX=9615 GN=ce10 PE=2 SV=1	2.13	1.12	244.50	9261502.1	20	2	0	9.09	110	41542
>tr|A0A5F4C7X6|A0A5F4C7X6_CANLF Nucleoporin 58 OS=Canis lupus familiaris OX=9615 GN=NUP58 PE=4 SV=1	0.10	0.09	34.10	9211896.3	1	1	1	3.12	544	8520
>tr|E2RJF6|E2RJF6_CANLF Mortality factor 4 like 1 OS=Canis lupus familiaris OX=9615 GN=MORF4L1 PE=4 SV=3	0.19	0.19	26.30	9211896.3	1	1	0	4.56	439	18584
>sp|Q9XSU7|RL27_CANLF 60S ribosomal protein L27 OS=Canis lupus familiaris OX=9615 GN=RPL27 PE=2 SV=3	0.10	0.03	46.70	9007219.1	10	1	0	3.68	136	314
>tr|F1PR47|F1PR47_CANLF DNA replication licensing factor MCM2 OS=Canis lupus familiaris OX=9615 GN=MCM2 PE=3 SV=3	0.28	0.24	196.50	8440132.5	3	1	0	0.57	881	9370
>tr|A0A5F4CCY0|A0A5F4CCY0_CANLF Programmed cell death 11 OS=Canis lupus familiaris OX=9615 GN=PDCD11 PE=4 SV=1	0.90	0.88	222.40	8439515.1	2	1	0	0.22	1829	1406
>tr|F1PJY1|F1PJY1_CANLF Mannosyl-glycoprotein endo-beta-N-acetylglucosaminidase OS=Canis lupus familiaris OX=9615 GN=ENGASE PE=3 SV=3	1.58	0.84	181.80	8387507.9	9	3	2	1.74	690	32761
>tr|F1PR54|F1PR54_CANLF Lactotransferrin OS=Canis lupus familiaris OX=9615 GN=LTF PE=3 SV=1	5.16	2.71	274.00	8022453.1	17	3	0	5.08	708	40436
>sp|F1PRN2|MYO1D_CANLF Unconventional myosin-Id OS=Canis lupus familiaris OX=9615 GN=MYO1D PE=1 SV=2	0.69	0.63	117.8	7978788.7	4	1	0	0.3	1006	763
>tr|J9P432|J9P432_CANLF Glutamine--fructose-6-phosphate transaminase (isomerizing) OS=Canis lupus familiaris OX=9615 GN=GFPT1 PE=4 SV=2	0.79	0.73	107.30	7976954.0	4	1	0	1.18	677	7191
>tr|A0A5F4D7Y5|A0A5F4D7Y5_CANLF Pleckstrin homology, MyTH4 and FERM domain containing H1 OS=Canis lupus familiaris OX=9615 GN=PLEKHH1 PE=4 SV=1	0.35	0.35	212.90	7955527.8	1	1	0	0.30	1342	5979
>tr|E2R6E0|E2R6E0_CANLF Lipocln_cytosolic_FA-bd_dom domain-containing protein OS=Canis lupus familiaris OX=9615 GN=LCNL1 PE=3 SV=2	2.12	2.12	199.40	7947178.4	1	1	0	3.68	299	1932
>tr|A0A5F4DDV9|A0A5F4DDV9_CANLF Actin alpha 2, smooth muscle OS=Canis lupus familiaris OX=9615 GN=ACTA2 PE=3 SV=1	0.55	0.55	103.00	7906270.6	1	1	1	10.16	374	9804
>tr|A0A5F4D7J3|A0A5F4D7J3_CANLF Non-specific serine/threonine protein kinase OS=Canis lupus familiaris OX=9615 GN=CDC42BPA PE=3 SV=1	0.10	0.02	61.10	7884672.7	3	1	1	0.17	1794	1069
>sp|Q9XSU7|RL27_CANLF 60S ribosomal protein L27 OS=Canis lupus familiaris OX=9615 GN=RPL27 PE=2 SV=3	0.10	0.00	6.40	7845848.0	3	1	0	3.68	136	314

>tr|A0A5F4D8I6|A0A5F4D8I6_CANLF Phospholipase A2 receptor 1 OS=Canis lupus familiaris OX=9615 GN=PLA2R1 PE=4 SV=1	0.73	0.71	131.10	7673585.8	2	1	0	0.50	1394	6796
>tr|F6V8I0|F6V8I0_CANLF Melanophilin OS=Canis lupus familiaris OX=9615 GN=MLPH PE=4 SV=2	0.11	0.09	148.90	7616201.3	2	1	0	1.46	549	10015
>tr|F1PB68|F1PB68_CANLF Olfactomedin 4 OS=Canis lupus familiaris OX=9615 GN=OLFM4 PE=4 SV=3	0.47	0.47	24.80	7551009.1	1	1	0	4.40	477	17246
>tr|F1PED7|F1PED7_CANLF Poly [ADP-ribose] polymerase OS=Canis lupus familiaris OX=9615 GN=PARP3 PE=4 SV=2	0.59	0.59	115.10	7523536.3	1	1	0	2.07	531	21143
>tr|E2RE16|E2RE16_CANLF Non-specific serine/threonine protein kinase OS=Canis lupus familiaris OX=9615 GN=PAK4 PE=4 SV=1	0.16	0.02	18.10	7522447.4	8	1	0	0.84	592	12735
>tr|J9NZJ2|J9NZJ2_CANLF Protein Wnt OS=Canis lupus familiaris OX=9615 GN=WNT6 PE=3 SV=2	0.85	0.85	268.20	7516265.4	1	1	0	1.47	339	11134
>tr|F1P9S9|F1P9S9_CANLF MutS homolog 3 OS=Canis lupus familiaris OX=9615 GN=MSH3 PE=3 SV=3	0.22	0.22	118.10	7514485.2	1	1	0	1.04	1058	29932
>tr|F1PPP9|F1PPP9_CANLF Family with sequence similarity 135 member A OS=Canis lupus familiaris OX=9615 GN=FAM135A PE=3 SV=3	0.37	0.27	121.60	7426745.5	6	1	0	1.22	1399	6815
>tr|A0A5F4D8I6|A0A5F4D8I6_CANLF Phospholipase A2 receptor 1 OS=Canis lupus familiaris OX=9615 GN=PLA2R1 PE=4 SV=1	0.15	0.13	110.40	7385920.1	2	1	0	0.50	1394	6796
>tr|F1PL54|F1PL54_CANLF Olfactory receptor OS=Canis lupus familiaris OX=9615 GN=OR52E2B PE=3 SV=2	0.11	0.11	115.20	7290701.6	1	1	1	2.25	311	5515
>tr|A0A5F4D9R8|A0A5F4D9R8_CANLF Transcription factor CP2 OS=Canis lupus familiaris OX=9615 GN=TFCP2 PE=3 SV=1	0.90	0.90	31.50	7139238.5	1	1	0	3.15	508	12674
>tr|E2QWH7|E2QWH7_CANLF Coiled-coil domain containing 127 OS=Canis lupus familiaris OX=9615 GN=CCDC127 PE=4 SV=3	0.10	0.09	66.80	7137123.7	1	1	0	3.34	479	28521
>tr|A0A5F4CXA7|A0A5F4CXA7_CANLF Maestro heat like repeat family member 9 OS=Canis lupus familiaris OX=9615 GN=MROH9 PE=4 SV=1	0.10	0.08	25.70	6962114.9	1	1	0	2.40	791	40674
>tr|A0A5F4CXA7|A0A5F4CXA7_CANLF Maestro heat like repeat family member 9 OS=Canis lupus familiaris OX=9615 GN=MROH9 PE=4 SV=1	0.33	0.33	10.80	6936616.2	1	1	0	2.40	791	40674
>tr|A0A5F4D6L9|A0A5F4D6L9_CANLF Sacsin molecular chaperone OS=Canis lupus familiaris OX=9615 GN=SACS PE=4 SV=1	0.26	0.22	231.10	6907424.1	3	1	0	0.09	4500	1444
>sp|F1PZV2|MFS12_CANLF Major facilitator superfamily domain-containing protein 12 OS=Canis lupus familiaris OX=9615 GN=MFSD12 PE=1 SV=3	0.15	0.15	14.80	6879602.3	1	1	1	5.23	478	230
>tr|A0A5F4C8H3|A0A5F4C8H3_CANLF Glycerol-3-phosphate acyltransferase 1, mitochondrial OS=Canis lupus familiaris OX=9615 GN=GPAM PE=3 SV=1	0.79	0.76	106.90	6793061.6	2	2	1	2.63	837	11048
>tr|A0A5F4DFY1|A0A5F4DFY1_CANLF SHH signaling and ciliogenesis regulator SDCCAG8 OS=Canis lupus familiaris OX=9615 GN=SDCCAG8 PE=4 SV=1	0.14	0.08	14.80	6735716.6	4	1	1	0.59	673	17765
>tr|E2RRE4|E2RRE4_CANLF Netrin 5 OS=Canis lupus familiaris OX=9615 GN=NTN5 PE=4 SV=3	0.16	0.14	125.30	6676765.0	2	1	0	1.02	489	13410
>sp|F1PRN2|MYO1D_CANLF Unconventional myosin-Id OS=Canis lupus familiaris OX=9615 GN=MYO1D PE=1 SV=2	0.61	0.58	156.40	6493781.8	3	1	0	0.30	1006	763
>tr|J9P432|J9P432_CANLF Glutamine--fructose-6-phosphate transaminase (isomerizing) OS=Canis lupus familiaris OX=9615 GN=GFPT1 PE=4 SV=2	0.23	0.23	53.50	6457411.5	1	1	0	1.18	677	7191
>tr|F1PSL8|F1PSL8_CANLF Matrix metallopeptidase 11 OS=Canis lupus familiaris OX=9615 GN=MMP11 PE=3 SV=3	0.39	0.37	65.90	6400593.3	2	1	1	1.86	485	3125

>tr|E2RA54|E2RA54_CANLF Bromodomain and WD repeat domain containing 3 OS=Canis lupus familiaris OX=9615 GN=BRWD3 PE=4 SV=3	0.10	0.02	48.20	6352116.0	2	1	0	0.29	1750	4294
>tr|F1PFP6|F1PFP6_CANLF Matrix metallopeptidase 12 OS=Canis lupus familiaris OX=9615 GN=MMP12 PE=3 SV=3	0.10	0.02	95.60	6344627.9	1	1	0	2.44	491	24324
>tr|J9NTK2|J9NTK2_CANLF J domain-containing protein OS=Canis lupus familiaris OX=9615 GN=DNAJC12 PE=4 SV=2	0.10	0.02	142.00	6310985.1	1	1	0	4.72	106	2310
>tr|J9P9K7|J9P9K7_CANLF Glycylpeptide N-tetradecanoyltransferase OS=Canis lupus familiaris OX=9615 GN=NMT2 PE=3 SV=2	0.32	0.32	59.00	6229770.5	1	1	0	3.16	507	19368
>tr|A0A5F4CR67|A0A5F4CR67_CANLF Methyl-CpG-binding protein 2 OS=Canis lupus familiaris OX=9615 GN=MECP2 PE=4 SV=1	0.10	0.09	126.10	6188853.7	1	1	1	2.41	498	1334
>tr|J9NZH4|J9NZH4_CANLF NTR domain-containing protein OS=Canis lupus familiaris OX=9615 GN=LOC102154527 PE=3 SV=2	0.10	0.10	53.10	6165021.7	1	1	0	8.60	221	39505
>tr|F6XAZ8|F6XAZ8_CANLF Dispatched RND transporter family member 1 OS=Canis lupus familiaris OX=9615 GN=DISP1 PE=4 SV=2	0.10	0.06	57.90	6150747.1	1	1	1	1.01	1485	5621
>sp|O18840|ACTB_CANLF Actin, cytoplasmic 1 OS=Canis lupus familiaris OX=9615 GN=ACTB PE=2 SV=3	0.30	0.24	150.00	6127412.6	4	1	0	2.93	375	642
>tr|A0A5F4CP99|A0A5F4CP99_CANLF CD109 molecule OS=Canis lupus familiaris OX=9615 GN=CD109 PE=3 SV=1	0.57	0.57	50.60	6058965.5	1	1	0	1.84	1520	4665
>sp|Q6UR05|MRP1_CANLF Multidrug resistance-associated protein 1 OS=Canis lupus familiaris OX=9615 GN=ABCC1 PE=1 SV=1	0.24	0.20	115.80	6036770.1	3	1	0	0.13	1531	259
>tr|F1PY73|F1PY73_CANLF SMG6 nonsense mediated mRNA decay factor OS=Canis lupus familiaris OX=9615 GN=SMG6 PE=4 SV=2	0.34	0.34	82.70	6024611.4	1	1	0	1.42	1412	40090
>tr|A0A5F4CJ52|A0A5F4CJ52_CANLF Beta-galactosidase OS=Canis lupus familiaris OX=9615 GN=GLB1 PE=3 SV=1	0.36	0.36	104.40	5983799.0	1	1	0	0.53	946	3892
>tr|A0A5F4CKD5|A0A5F4CKD5_CANLF Polypeptide N-acetylgalactosaminyltransferase OS=Canis lupus familiaris OX=9615 GN=GALNT6 PE=3 SV=1	0.69	0.69	115.90	5950260.1	1	1	0	1.71	644	1617
>sp|Q9XSU7|RL27_CANLF 60S ribosomal protein L27 OS=Canis lupus familiaris OX=9615 GN=RPL27 PE=2 SV=3	0.12	0.02	16.60	5923851.1	6	1	0	3.68	136	314
>sp|P50996|ATP4A_CANLF Potassium-transporting ATPase alpha chain 1 OS=Canis lupus familiaris OX=9615 GN=ATP4A PE=2 SV=3	0.55	0.55	38.40	5902147.9	1	1	1	2.90	1034	672
>tr|F1PQM0|F1PQM0_CANLF BAH domain and coiled-coil containing 1 OS=Canis lupus familiaris OX=9615 GN=BAHCC1 PE=4 SV=3	0.63	0.63	124.40	5873788.6	1	1	1	0.43	2565	10908
>tr|J9NS29|J9NS29_CANLF Cystatin domain-containing protein OS=Canis lupus familiaris OX=9615 GN=LOC607874 PE=4 SV=2	0.21	0.21	111.00	5867607.9	1	1	0	6.39	313	30016
>tr|A0A5F4CCD0|A0A5F4CCD0_CANLF Cysteine rich secretory protein 2 OS=Canis lupus familiaris OX=9615 GN=CRISP2 PE=3 SV=1	0.24	0.20	127.90	5859721.0	3	1	0	4.82	311	11017
>sp|O97758|ZO1_CANLF Tight junction protein ZO-1 OS=Canis lupus familiaris OX=9615 GN=TJP1 PE=1 SV=1	0.71	0.71	7.50	5843550.4	1	1	0	0.28	1769	139
>tr|A0A5F4C9R3|A0A5F4C9R3_CANLF Ubiquitin protein ligase E3 component n-recognin 5 OS=Canis lupus familiaris OX=9615 GN=UBR5 PE=4 SV=1	0.70	0.71	150.00	5770903.4	1	1	0	0.18	2714	3284
>tr|F6XN72|F6XN72_CANLF Leucine rich repeat containing 71 OS=Canis lupus familiaris OX=9615 GN=LRRC71 PE=4 SV=1	0.10	0.06	105.20	5739592.9	1	1	1	1.96	560	34087
>tr|F1PFZ5|F1PFZ5_CANLF Milk fat globule EGF and factor V/VIII domain containing OS=Canis lupus familiaris OX=9615 GN=MFGE8 PE=4 SV=3	1.84	1.84	231.40	5675948.1	1	1	0	1.87	428	7079

>tr|A0A5F4D7J3|A0A5F4D7J3_CANLF Non-specific serine/threonine protein kinase OS=Canis lupus familiaris OX=9615 GN=CDC42BPA PE=3 SV=1	0.85	0.85	235.30	5675005.7	1	1	0	0.22	1794	1069
>tr|J9P539|J9P539_CANLF Telomeric repeat-binding factor OS=Canis lupus familiaris OX=9615 GN=TERF1 PE=4 SV=2	0.10	0.07	98.60	5673217.6	1	1	1	2.66	413	7133
>tr|A0A5F4BRN6|A0A5F4BRN6_CANLF Class II major histocompatibility complex transactivator OS=Canis lupus familiaris OX=9615 GN=CIITA PE=4 SV=1	0.60	0.60	179.40	5590030.8	1	1	0	0.34	1191	5452
>tr|F1P9L4|F1P9L4_CANLF Dual-specificity kinase OS=Canis lupus familiaris OX=9615 GN=DYRK1A PE=3 SV=3	0.44	0.44	88.90	5578141.7	1	1	0	0.52	762	4349
>tr|F1PG90|F1PG90_CANLF Leucine rich repeat LGI family member 3 OS=Canis lupus familiaris OX=9615 GN=LGI3 PE=4 SV=3	0.10	0.07	74.00	5562585.2	1	1	0	0.73	548	1276
>tr|J9NS29|J9NS29_CANLF Cystatin domain-containing protein OS=Canis lupus familiaris OX=9615 GN=LOC607874 PE=4 SV=2	0.68	0.68	183.50	5535283.6	1	1	0	6.39	313	30016
>tr|A0A5F4D9S5|A0A5F4D9S5_CANLF Hyaluronoglucosaminidase OS=Canis lupus familiaris OX=9615 GN=CEMIP PE=3 SV=1	0.44	0.42	140.80	5517350.8	2	1	0	0.24	1684	9775
>tr|F1PM73|F1PM73_CANLF Palmitoyltransferase OS=Canis lupus familiaris OX=9615 GN=ZDHHC23 PE=3 SV=3	0.19	0.19	36.60	5484916.3	1	1	0	3.98	427	22460
>tr|J9P822|J9P822_CANLF TPR_REGION domain-containing protein OS=Canis lupus familiaris OX=9615 GN=TTC16 PE=4 SV=2	0.58	0.58	83.60	5461242.9	1	1	0	1.73	866	2823
>tr|E2RCF4|E2RCF4_CANLF Replication factor C subunit 5 OS=Canis lupus familiaris OX=9615 GN=RFC5 PE=3 SV=1	0.40	0.40	17.20	5429132.6	1	1	0	5.60	339	16271
>tr|A0A5F4CU74|A0A5F4CU74_CANLF FYVE, RhoGEF and PH domain containing 6 OS=Canis lupus familiaris OX=9615 GN=FGD6 PE=4 SV=1	0.10	0.05	74.00	5395228.4	1	1	1	1.67	1320	14282
>tr|A0A5F4DFC0|A0A5F4DFC0_CANLF Caseinolytic mitochondrial matrix peptidase chaperone subunit X OS=Canis lupus familiaris OX=9615 GN=CLPX PE=4 SV=1	0.10	0.02	7.20	5350398.5	1	1	1	2.58	776	5729
>tr|F1PYS8|F1PYS8_CANLF Olfactory receptor OS=Canis lupus familiaris OX=9615 GN=OR5P6 PE=3 SV=3	0.16	0.16	13.50	5338911.3	1	1	1	6.69	314	20945
>tr|A0A5F4DKM7|A0A5F4DKM7_CANLF Terminal uridylyl transferase 4 OS=Canis lupus familiaris OX=9615 GN=TUT4 PE=4 SV=1	0.47	0.47	120.60	5294282.5	1	1	0	0.19	1611	941
>tr|F1PFZ5|F1PFZ5_CANLF Milk fat globule EGF and factor V/VIII domain containing OS=Canis lupus familiaris OX=9615 GN=MFGE8 PE=4 SV=3	1.48	1.48	191.60	5263001.6	1	1	0	1.87	428	7079
>tr|E2RN65|E2RN65_CANLF Phosphatidylinositol-glycan biosynthesis class W protein OS=Canis lupus familiaris OX=9615 GN=PIGW PE=3 SV=2	0.39	0.37	116.20	5261859.4	2	1	0	1.38	509	21598
>tr|J9NZH4|J9NZH4_CANLF NTR domain-containing protein OS=Canis lupus familiaris OX=9615 GN=LOC102154527 PE=3 SV=2	0.13	0.13	47.70	5248951.3	1	1	0	8.60	221	39505
>tr|A0A5F4CCY0|A0A5F4CCY0_CANLF Programmed cell death 11 OS=Canis lupus familiaris OX=9615 GN=PDCD11 PE=4 SV=1	0.94	0.95	217.30	5211573.7	1	1	0	0.22	1829	1406
>sp|Q8WN22|PRKDC_CANLF DNA-dependent protein kinase catalytic subunit OS=Canis lupus familiaris OX=9615 GN=PRKDC PE=2 SV=1	0.16	0.16	127.20	5112820.5	1	1	0	0.10	4144	338
>tr|E2RSI6|E2RSI6_CANLF Ezrin OS=Canis lupus familiaris OX=9615 GN=EZR PE=4 SV=1	0.61	0.61	131.80	4896880.6	1	1	0	1.19	586	15650
>tr|A0A5F4CUE8|A0A5F4CUE8_CANLF Senataxin OS=Canis lupus familiaris OX=9615 GN=SETX PE=4 SV=1	0.39	0.37	110.60	4835244.7	2	1	0	0.26	2645	1796
>tr|J9P3R7|J9P3R7_CANLF SCO-spondin OS=Canis lupus familiaris OX=9615 GN=SSPO PE=3 SV=2	0.20	0.13	152.20	4819541.2	5	1	1	0.23	5112	24898
>sp|Q8WMX5|S15A1_CANLF Solute carrier family 15 member 1 OS=Canis lupus familiaris OX=9615 GN=SLC15A1 PE=2 SV=2	0.27	0.25	54.40	4818120.4	2	1	1	1.27	708	5

>tr|E2R824|E2R824_CANLF Zinc finger protein 518B OS=Canis lupus familiaris OX=9615 GN=ZNF518B PE=4 SV=3	0.14	0.14	92.90	4810757.6	1	1	0	0.80	1000	24468
>tr|A0A5F4D4W8|A0A5F4D4W8_CANLF Dedicator of cytokinesis 1 OS=Canis lupus familiaris OX=9615 GN=DOCK1 PE=3 SV=1	0.10	0.09	69.40	4734497.8	1	1	1	1.02	1855	11311
>sp|P49822|ALBU_CANLF Albumin OS=Canis lupus familiaris OX=9615 GN=ALB PE=1 SV=3	0.10	0.09	60.00	4726404.3	1	1	1	2.14	608	490
>tr|J9P3J0|J9P3J0_CANLF R3H domain and coiled-coil containing 1 OS=Canis lupus familiaris OX=9615 GN=R3HCC1 PE=4 SV=1	0.23	0.23	99.90	4705074.6	1	1	0	1.09	458	1410
>tr|A0A5F4CJ52|A0A5F4CJ52_CANLF Beta-galactosidase OS=Canis lupus familiaris OX=9615 GN=GLB1 PE=3 SV=1	0.23	0.23	99.90	4705074.6	1	1	0	0.53	946	3892
>tr|A0A5F4BSI9|A0A5F4BSI9_CANLF Centrosomal protein 350 OS=Canis lupus familiaris OX=9615 GN=CEP350 PE=4 SV=1	0.10	0.04	158.90	4672931.6	1	1	0	0.32	3113	15196
>tr|F1PHA9|F1PHA9_CANLF Motile sperm domain containing 2 OS=Canis lupus familiaris OX=9615 GN=MOSPD2 PE=4 SV=3	0.57	0.57	148.20	4672931.6	1	1	0	0.97	518	10201
>tr|A0A5F4DFU6|A0A5F4DFU6_CANLF Acetyl-CoA acyltransferase 1 OS=Canis lupus familiaris OX=9615 GN=ACAA1 PE=3 SV=1	0.13	0.11	217.70	4646285.0	2	1	0	0.97	513	2936
>tr|A0A5F4C0S7|A0A5F4C0S7_CANLF HEAT repeat containing 5A OS=Canis lupus familiaris OX=9615 GN=HEATR5A PE=3 SV=1	0.10	0.00	51.60	4620329.6	3	1	0	0.25	1995	1753
>tr|F1PZN6|F1PZN6_CANLF Olfactory receptor OS=Canis lupus familiaris OX=9615 PE=3 SV=3	0.13	0.13	48.60	4598782.0	1	1	1	6.77	310	37737
>tr|A0A5F4C8H3|A0A5F4C8H3_CANLF Glycerol-3-phosphate acyltransferase 1, mitochondrial OS=Canis lupus familiaris OX=9615 GN=GPAM PE=3 SV=1	0.87	0.87	121.50	4596836.8	1	1	0	1.31	837	11048
>tr|A0A5F4CLI1|A0A5F4CLI1_CANLF Histone deacetylase 6 OS=Canis lupus familiaris OX=9615 GN=HDAC6 PE=4 SV=1	0.63	0.63	250.70	4526077.8	1	1	0	0.43	1175	4057
>tr|F1P721|F1P721_CANLF Kinase suppressor of ras 2 OS=Canis lupus familiaris OX=9615 GN=KSR2 PE=4 SV=3	0.21	0.21	17.90	4523411.4	1	1	0	1.37	950	2276
>tr|F1PHA9|F1PHA9_CANLF Motile sperm domain containing 2 OS=Canis lupus familiaris OX=9615 GN=MOSPD2 PE=4 SV=3	0.33	0.33	152.10	4470267.0	1	1	0	0.97	518	10201
>tr|A0A5F4BX19|A0A5F4BX19_CANLF Multidrug and toxin extrusion protein OS=Canis lupus familiaris OX=9615 GN=ALDH3A2 PE=3 SV=1	0.56	0.56	137.70	4451339.3	1	1	0	0.61	983	8236
>tr|F6XN72|F6XN72_CANLF Leucine rich repeat containing 71 OS=Canis lupus familiaris OX=9615 GN=LRRC71 PE=4 SV=1	0.15	0.15	97.40	4431542.6	1	1	1	1.96	560	34087
>tr|A0A5F4CUE8|A0A5F4CUE8_CANLF Senataxin OS=Canis lupus familiaris OX=9615 GN=SETX PE=4 SV=1	0.23	0.21	120.30	4424101.7	2	1	0	0.26	2645	1796
>tr|A0A5F4CXV0|A0A5F4CXV0_CANLF Mahogunin ring finger 1 OS=Canis lupus familiaris OX=9615 GN=MGRN1 PE=4 SV=1	0.31	0.31	84.00	4399504.7	1	1	0	2.19	549	4641
>tr|A0A5F4CJI0|A0A5F4CJI0_CANLF Folate_rec domain-containing protein OS=Canis lupus familiaris OX=9615 GN=FOLR1 PE=3 SV=1	0.36	0.36	192.90	4394554.1	1	1	0	3.14	255	10075
>sp|P21842|CMA1_CANLF Chymase OS=Canis lupus familiaris OX=9615 GN=CMA1 PE=1 SV=1	0.16	0.16	84.50	4287964.4	1	1	0	0.80	249	34
>tr|F1PWR1|F1PWR1_CANLF Insulin like growth factor 2 receptor OS=Canis lupus familiaris OX=9615 GN=IGF2R PE=4 SV=3	0.10	0.03	105.30	4278844.4	1	1	1	0.85	2474	1073
>tr|E2R5W0|E2R5W0_CANLF Myosin binding protein C2 OS=Canis lupus familiaris OX=9615 GN=MYBPC2 PE=3 SV=2	1.15	1.15	88.50	4231783.1	1	1	0	4.15	265	19744

>tr|A0A5F4D9S5|A0A5F4D9S5_CANLF Hyaluronoglucosaminidase OS=Canis lupus familiaris OX=9615 GN=CEMIP PE=3 SV=1	0.16	0.16	103.40	4195688.3	1	1	0	0.24	1684	9775
>tr|F1PQ45|F1PQ45_CANLF Integrin subunit alpha 11 OS=Canis lupus familiaris OX=9615 GN=ITGA11 PE=3 SV=3	0.12	0.12	68.80	4113875.1	1	1	1	1.52	1183	26268
>sp|E2RED8|AP4M1_CANLF AP-4 complex subunit mu-1 OS=Canis lupus familiaris OX=9615 GN=AP4M1 PE=3 SV=2	0.91	0.91	135.10	3990557.9	1	1	0	0.66	452	634
>tr|E2RHZ3|E2RHZ3_CANLF DEAH-box helicase 37 OS=Canis lupus familiaris OX=9615 GN=DHX37 PE=4 SV=1	0.40	0.40	90.40	3802195.2	1	1	1	0.52	1149	44834
>tr|A0A5F4CJI0|A0A5F4CJI0_CANLF Folate_rec domain-containing protein OS=Canis lupus familiaris OX=9615 GN=FOLR1 PE=3 SV=1	1.25	1.25	197.40	3770823.4	1	1	0	3.14	255	10075
>sp|Q9XS65|PTGDS_CANLF Prostaglandin-H2 D-isomerase OS=Canis lupus familiaris OX=9615 GN=PTGDS PE=2 SV=1	1.04	0.72	234.20	3595633.3	7	2	1	10.47	191	165
>tr|E2RN65|E2RN65_CANLF Phosphatidylinositol-glycan biosynthesis class W protein OS=Canis lupus familiaris OX=9615 GN=PIGW PE=3 SV=2	0.62	0.62	121.00	3567338.7	1	1	0	1.38	509	21598
>tr|A0A5F4DFX0|A0A5F4DFX0_CANLF Exportin-T OS=Canis lupus familiaris OX=9615 GN=XPOT PE=3 SV=1	0.10	0.01	115.10	3423369.5	2	1	0	0.75	938	2329
>sp|Q697L1|TRPV1_CANLF Transient receptor potential cation channel subfamily V member 1 OS=Canis lupus familiaris OX=9615 GN=TRPV1 PE=2 SV=1	0.33	0.31	73.20	3403778.4	2	1	1	0.36	840	297
>sp|F1PRN2|MYO1D_CANLF Unconventional myosin-Id OS=Canis lupus familiaris OX=9615 GN=MYO1D PE=1 SV=2	0.65	0.65	112.00	3386784.9	1	1	0	0.30	1006	763
>sp|P23685|NAC1_CANLF Sodium/calcium exchanger 1 OS=Canis lupus familiaris OX=9615 GN=SLC8A1 PE=1 SV=1	0.10	0.05	98.60	3254155.3	1	1	0	1.03	970	764
>sp|F1PRN2|MYO1D_CANLF Unconventional myosin-Id OS=Canis lupus familiaris OX=9615 GN=MYO1D PE=1 SV=2	0.41	0.41	113.90	3245197.0	1	1	0	0.30	1006	763
>tr|A0A5F4CPU3|A0A5F4CPU3_CANLF SEC24 homolog D, COPII coat complex component OS=Canis lupus familiaris OX=9615 GN=SEC24D PE=3 SV=1	0.10	0.00	21.20	3207395.2	2	1	0	0.53	946	2125
>sp|B8K1W2|ABCBB_CANLF Bile salt export pump OS=Canis lupus familiaris OX=9615 GN=Abcb11e PE=1 SV=1	0.87	0.87	216.70	3092899.8	1	1	0	0.30	1325	527
>tr|A0A5F4CQP5|A0A5F4CQP5_CANLF Coiled-coil and C2 domain containing 2A OS=Canis lupus familiaris OX=9615 GN=CC2D2A PE=4 SV=1	0.33	0.33	43.60	3043436.9	1	1	1	0.43	1638	11842
>tr|J9P432|J9P432_CANLF Glutamine--fructose-6-phosphate transaminase (isomerizing) OS=Canis lupus familiaris OX=9615 GN=GFPT1 PE=4 SV=2	1.06	1.06	74.60	3036486.9	1	1	0	1.18	677	7191
>tr|E2RIV7|E2RIV7_CANLF Syntrophin alpha 1 OS=Canis lupus familiaris OX=9615 GN=SNTA1 PE=3 SV=3	0.10	0.00	33.90	2913593.7	2	1	1	1.03	486	34454
>tr|A0A5F4D9S5|A0A5F4D9S5_CANLF Hyaluronoglucosaminidase OS=Canis lupus familiaris OX=9615 GN=CEMIP PE=3 SV=1	0.38	0.39	142.90	2908687.7	1	1	0	0.24	1684	9775
>tr|A0A5F4BZ61|A0A5F4BZ61_CANLF G_PROTEIN_RECEP_F1_2 domain-containing protein OS=Canis lupus familiaris OX=9615 GN=OR5D13 PE=4 SV=1	0.57	0.57	54.20	2896904.8	1	1	0	1.70	294	37537
>tr|E2R868|E2R868_CANLF [histone H4]-N-methyl-L-lysine20 N-methyltransferase KMT5B OS=Canis lupus familiaris OX=9615 GN=KMT5B PE=4 SV=3	0.10	0.00	42.50	2848313.2	2	1	0	0.56	885	7704
>sp|O18840|ACTB_CANLF Actin, cytoplasmic 1 OS=Canis lupus familiaris OX=9615 GN=ACTB PE=2 SV=3	0.98	0.94	302.60	2782134.0	3	1	0	2.93	375	642
>sp|E2RED8|AP4M1_CANLF AP-4 complex subunit mu-1 OS=Canis lupus familiaris OX=9615 GN=AP4M1 PE=3 SV=2	1.18	1.18	148.60	2687637.1	1	1	0	0.66	452	634

>tr|F6XBJ5|F6XBJ5_CANLF Integrator complex subunit 1 OS=Canis lupus familiaris OX=9615 GN=INTS1 PE=4 SV=1	0.68	0.66	75.30	2672468.8	2	1	0	0.73	2188	9613
>tr|F1PKS8|F1PKS8_CANLF Anoctamin OS=Canis lupus familiaris OX=9615 GN=VWF PE=3 SV=3	0.12	0.02	133.60	2612261.7	6	1	1	1.61	992	42310
>tr|E2RS58|E2RS58_CANLF Solute carrier family 35 member B1 OS=Canis lupus familiaris OX=9615 GN=SLC35B1 PE=3 SV=2	0.10	0.05	64.70	2610967.5	1	1	1	0.83	360	7421
>tr|A0A5F4CEY4|A0A5F4CEY4_CANLF ELL associated factor 2 OS=Canis lupus familiaris OX=9615 GN=EAF2 PE=3 SV=1	0.11	0.09	8.40	2527130.3	2	1	0	0.74	543	6686
>tr|A0A5F4BY19|A0A5F4BY19_CANLF RELT like 1 OS=Canis lupus familiaris OX=9615 GN=RELL1 PE=3 SV=1	0.20	0.18	158.50	2270381.7	2	1	1	4.65	258	11917
>sp|Q9XS65|PTGDS_CANLF Prostaglandin-H2 D-isomerase OS=Canis lupus familiaris OX=9615 GN=PTGDS PE=2 SV=1	1.62	1.44	252.20	2203885.9	11	2	1	10.47	191	165
>tr|A0A5F4CTR9|A0A5F4CTR9_CANLF Semaphorin 3A OS=Canis lupus familiaris OX=9615 GN=SEMA3A PE=3 SV=1	0.22	0.22	5.90	1961165.6	1	1	0	0.68	732	2791
>tr|F1PJ45|F1PJ45_CANLF Adhesion G protein-coupled receptor L3 OS=Canis lupus familiaris OX=9615 GN=ADGRL3 PE=4 SV=2	0.22	0.22	5.90	1961165.6	1	1	0	0.33	1528	2066
>tr|F1PPQ1|F1PPQ1_CANLF Schlafen like 1 OS=Canis lupus familiaris OX=9615 GN=SLFNL1 PE=4 SV=3	0.10	0.01	67.40	1816832.0	1	1	0	2.31	347	24972
>sp|Q30DN6|KDM5D_CANLF Lysine-specific demethylase 5D OS=Canis lupus familiaris OX=9615 GN=KDM5D PE=2 SV=1	0.35	0.35	93.40	1805107.1	1	1	0	0.13	1545	213
>tr|A0A5F4DIL6|A0A5F4DIL6_CANLF Dpy-19 like C-mannosyltransferase 3 OS=Canis lupus familiaris OX=9615 GN=DPY19L3 PE=3 SV=1	0.30	0.28	117.00	1796079.9	2	1	1	1.75	742	42539
>tr|A0A5F4D3R3|A0A5F4D3R3_CANLF Mitogen-activated protein kinase kinase kinase 4 OS=Canis lupus familiaris OX=9615 GN=MAP3K4 PE=4 SV=1	0.29	0.29	79.30	1732983.2	1	1	0	0.55	1630	2223
>tr|F1PPP9|F1PPP9_CANLF Family with sequence similarity 135 member A OS=Canis lupus familiaris OX=9615 GN=FAM135A PE=3 SV=3	0.20	0.18	105.20	1727197.3	2	1	0	1.22	1399	6815
>tr|E2RQX2|E2RQX2_CANLF Ubiquitin specific peptidase 53 OS=Canis lupus familiaris OX=9615 GN=USP53 PE=4 SV=2	0.65	0.65	43.50	1688846.7	1	1	1	1.35	1112	11373
>tr|A0A5F4D6Q9|A0A5F4D6Q9_CANLF Sialic acid binding Ig like lectin 1 OS=Canis lupus familiaris OX=9615 GN=SIGLEC1 PE=4 SV=1	0.38	0.38	76.90	1673862.5	1	1	1	0.99	1719	15622
>tr|F1PPP9|F1PPP9_CANLF Family with sequence similarity 135 member A OS=Canis lupus familiaris OX=9615 GN=FAM135A PE=3 SV=3	0.14	0.14	93.40	1627559.1	1	1	0	1.22	1399	6815
>tr|F1PC83|F1PC83_CANLF ADAM metallopeptidase with thrombospondin type 1 motif 18 OS=Canis lupus familiaris OX=9615 GN=ADAMTS18 PE=4 SV=3	0.24	0.24	110.70	1624420.2	1	1	1	0.34	1191	4997
>tr|E2RTH4|E2RTH4_CANLF Dendrin OS=Canis lupus familiaris OX=9615 GN=DDN PE=4 SV=2	0.65	0.65	133.70	1515276.4	1	1	1	2.84	704	20318
>tr|F1PEJ3|F1PEJ3_CANLF WD_REPEATS_REGION domain-containing protein OS=Canis lupus familiaris OX=9615 GN=WDR97 PE=4 SV=3	0.14	0.14	26.20	1446384.1	1	1	0	0.34	1467	13780
>sp|A2IBY8|MIP_CANLF Lens fiber major intrinsic protein OS=Canis lupus familiaris OX=9615 GN=MIP PE=2 SV=1	0.10	0.00	34.40	1437915.6	1	1	0	1.90	263	112
>tr|F1PI09|F1PI09_CANLF Aldehyde oxidase OS=Canis lupus familiaris OX=9615 GN=AOX2 PE=3 SV=3	0.37	0.36	180.30	1428729.9	2	1	0	0.67	1347	21650
>sp|Q9XS65|PTGDS_CANLF Prostaglandin-H2 D-isomerase OS=Canis lupus familiaris OX=9615 GN=PTGDS PE=2 SV=1	0.49	0.41	222.20	1260896.8	5	1	1	7.33	191	165

>sp|Q28895|NPC2_CANLF NPC intracellular cholesterol transporter 2 OS=Canis lupus familiaris OX=9615 GN=NPC2 PE=2 SV=1	0.96	0.65	128.50	1220988.5	3	2	0	14.77	149	153
>sp|O46607|GPX5_CANLF Epididymal secretory glutathione peroxidase OS=Canis lupus familiaris OX=9615 GN=GPX5 PE=2 SV=1	3.59	1.94	157.90	1119106.1	8	3	0	11.76	221	564
>sp|Q28895|NPC2_CANLF NPC intracellular cholesterol transporter 2 OS=Canis lupus familiaris OX=9615 GN=NPC2 PE=2 SV=1	1.37	1.05	146.20	1099642.5	2	2	0	14.77	149	153
>tr|F1PJP1|F1PJP1_CANLF Dynein axonemal heavy chain 11 OS=Canis lupus familiaris OX=9615 GN=DNAH11 PE=3 SV=3	0.65	0.65	62.90	1056658.7	1	1	0	0.35	4519	42705
>tr|F1PHN5|F1PHN5_CANLF G protein-coupled receptor class C group 5 member A OS=Canis lupus familiaris OX=9615 GN=GPRC5A PE=4 SV=2	0.90	0.90	12.20	1034092.2	1	1	0	3.64	357	3905
>tr|J9NSS6|J9NSS6_CANLF DNA helicase OS=Canis lupus familiaris OX=9615 GN=CHD2 PE=4 SV=2	0.14	0.10	57.50	1031603.0	3	1	0	0.28	1780	1264
>tr|J9P0B4|J9P0B4_CANLF Tudor domain containing 15 OS=Canis lupus familiaris OX=9615 GN=TDRD15 PE=4 SV=2	0.67	0.65	153.10	1026905.3	2	1	0	0.57	2105	4188
>sp|P23685|NAC1_CANLF Sodium/calcium exchanger 1 OS=Canis lupus familiaris OX=9615 GN=SLC8A1 PE=1 SV=1	0.37	0.37	153.60	982205.0	1	1	0	1.03	970	764
>sp|Q9GL25|ESPB1_CANLF Epididymal sperm-binding protein 1 OS=Canis lupus familiaris OX=9615 GN=ELSPBP1 PE=1 SV=1	1.64	1.60	214.70	944907.5	3	1	0	4.49	245	36
>tr|F1PJY1|F1PJY1_CANLF Mannosyl-glycoprotein endo-beta-N-acetylglucosaminidase OS=Canis lupus familiaris OX=9615 GN=ENGASE PE=3 SV=3	0.30	0.30	129.10	909314.2	1	1	1	1.74	690	32761
>sp|Q2Q421|AGRE2_CANLF Adhesion G protein-coupled receptor E2 OS=Canis lupus familiaris OX=9615 GN=ADGRE2 PE=2 SV=2	0.67	0.67	12.90	896724.6	1	1	0	0.48	830	472
>tr|F6UNY1|F6UNY1_CANLF Neuropilin OS=Canis lupus familiaris OX=9615 GN=NRP1 PE=3 SV=2	0.67	0.67	12.90	896724.6	1	1	0	0.45	890	1315
>tr|A0A5F4D844|A0A5F4D844_CANLF Adhesion G protein-coupled receptor E5 OS=Canis lupus familiaris OX=9615 GN=ADGRE5 PE=4 SV=1	0.18	0.12	19.40	876804.0	4	1	0	0.48	836	1078
>tr|A0A5F4DB52|A0A5F4DB52_CANLF Neuropilin OS=Canis lupus familiaris OX=9615 GN=NRP1 PE=3 SV=1	0.18	0.12	19.40	876804.0	4	1	0	0.47	856	4302
>sp|Q6AW47|EST5A_CANLF Carboxylesterase 5A OS=Canis lupus familiaris OX=9615 GN=CES5A PE=2 SV=1	0.85	0.79	188.90	825726.6	4	1	0	1.22	575	629
>tr|A0A5F4CCD0|A0A5F4CCD0_CANLF Cysteine rich secretory protein 2 OS=Canis lupus familiaris OX=9615 GN=CRISP2 PE=3 SV=1	1.67	1.67	175.00	703679.8	1	1	0	4.82	311	11017
>tr|E2R9Z9|E2R9Z9_CANLF Leucine rich repeat containing 8 VRAC subunit C OS=Canis lupus familiaris OX=9615 GN=LRRC8C PE=3 SV=1	0.12	0.12	65.70	651475.4	1	1	0	0.62	803	9247
>sp|Q9GL25|ESPB1_CANLF Epididymal sperm-binding protein 1 OS=Canis lupus familiaris OX=9615 GN=ELSPBP1 PE=1 SV=1	1.06	1.02	238.00	613431.8	3	1	0	4.49	245	36
>tr|A0A5F4CPU3|A0A5F4CPU3_CANLF SEC24 homolog D, COPII coat complex component OS=Canis lupus familiaris OX=9615 GN=SEC24D PE=3 SV=1	0.58	0.58	7.90	555937.6	1	1	0	0.53	946	2125
>tr|A0A5F4C0S7|A0A5F4C0S7_CANLF HEAT repeat containing 5A OS=Canis lupus familiaris OX=9615 GN=HEATR5A PE=3 SV=1	0.58	0.58	7.90	555937.6	1	1	0	0.25	1995	1753
>tr|A0A5F4C5M9|A0A5F4C5M9_CANLF Contactin associated protein 1 OS=Canis lupus familiaris OX=9615 GN=CNTNAP1 PE=3 SV=1	0.81	0.60	154.40	544591.4	4	2	0	0.65	1388	10827
>tr|J9NYC7|J9NYC7_CANLF Dynein axonemal heavy chain 12 OS=Canis lupus familiaris OX=9615 GN=DNAH12 PE=3 SV=1	0.35	0.32	134.30	503314.2	3	1	0	0.33	3960	15992

>tr|F1PCZ0|F1PCZ0_CANLF Septin OS=Canis lupus familiaris OX=9615 GN=SEPTIN5 PE=3 SV=2	0.65	0.65	38.00	486065.2	1	1	0	4.23	378	3212
>tr|Q9XSV4|Q9XSV4_CANLF CE10 protein OS=Canis lupus familiaris OX=9615 GN=ce10 PE=2 SV=1	1.95	1.93	149.90	439663.0	3	2	0	9.09	110	41542
>sp|P79149|PININ_CANLF Pinin OS=Canis lupus familiaris OX=9615 GN=PNN PE=2 SV=3	0.37	0.37	64.10	428019.6	1	1	0	0.65	773	156
>sp|E2QRY6|NNRE_CANLF NAD(P)H-hydrate epimerase OS=Canis lupus familiaris OX=9615 GN=NAXE PE=3 SV=1	0.19	0.17	109.00	357197.7	2	1	0	1.74	288	159
>tr|A0A5F4BSK0|A0A5F4BSK0_CANLF RUN and SH3 domain containing 2 OS=Canis lupus familiaris OX=9615 GN=RUSC2 PE=4 SV=1	0.54	0.54	47.70	291337.0	1	1	0	0.28	1450	24598
>tr|A0A5F4C5M9|A0A5F4C5M9_CANLF Contactin associated protein 1 OS=Canis lupus familiaris OX=9615 GN=CNTNAP1 PE=3 SV=1	0.31	0.31	44.00	288154.4	1	1	0	0.36	1388	10827
>tr|A0A5F4CIJ4|A0A5F4CIJ4_CANLF Transmembrane protein 63A OS=Canis lupus familiaris OX=9615 GN=TMEM63A PE=3 SV=1	0.40	0.40	115.40	264293.0	1	1	0	0.76	789	13092
>tr|J9P821|J9P821_CANLF A-kinase anchoring protein 3 OS=Canis lupus familiaris OX=9615 GN=AKAP3 PE=3 SV=2	0.65	0.65	18.30	241661.7	1	1	0	2.03	787	7082
>tr|A0A5F4DHH0|A0A5F4DHH0_CANLF ATP binding cassette subfamily A member 1 OS=Canis lupus familiaris OX=9615 GN=ABCA1 PE=4 SV=1	0.38	0.38	10.10	241479.4	1	1	1	0.51	2175	3709
>tr|E2RP25|E2RP25_CANLF Tyrosine-protein kinase OS=Canis lupus familiaris OX=9615 GN=ITK PE=3 SV=2	0.24	0.24	7.90	238020.7	1	1	0	0.97	620	41809
>tr|E2RG75|E2RG75_CANLF Inactive ribonuclease-like protein 9 OS=Canis lupus familiaris OX=9615 GN=RNASE9 PE=3 SV=2	0.79	0.79	238.60	169807.0	1	1	0	4.55	198	41734
>tr|E2QX33|E2QX33_CANLF Coiled-coil and C2 domain containing 1A OS=Canis lupus familiaris OX=9615 GN=CC2D1A PE=3 SV=1	0.14	0.14	19.40	161331.3	1	1	0	0.53	951	10961
>tr|E2R4U2|E2R4U2_CANLF Proline rich 35 OS=Canis lupus familiaris OX=9615 GN=PRR35 PE=4 SV=2	0.29	0.30	93.90	158230.7	1	1	0	1.49	536	44008
>sp|Q28895|NPC2_CANLF NPC intracellular cholesterol transporter 2 OS=Canis lupus familiaris OX=9615 GN=NPC2 PE=2 SV=1	1.35	1.35	162.30	152904.1	1	1	0	6.04	149	153
>tr|F6UP22|F6UP22_CANLF Metallophosphoesterase domain containing 1 OS=Canis lupus familiaris OX=9615 GN=MPPED1 PE=4 SV=1	0.20	0.20	15.60	141792.6	1	1	0	3.34	359	4733
>tr|E2R179|E2R179_CANLF Coiled-coil domain containing 30 OS=Canis lupus familiaris OX=9615 GN=CCDC30 PE=4 SV=3	0.29	0.29	23.60	141016.9	1	1	0	1.41	709	15846
>tr|A0A5F4DDH9|A0A5F4DDH9_CANLF Saccharopine dehydrogenase (putative) OS=Canis lupus familiaris OX=9615 GN=SCCPDH PE=4 SV=1	0.27	0.27	61.70	102848.6	1	1	0	3.09	421	6763
